# Supplementary material for: Safety, immunogenicity and efficacy of the self-amplifying mRNA ARCT-154 COVID-19 vaccine: pooled phase 1, 2, 3a and 3b randomized, controlled trials
Source: Nat Commun. 2024 May 14;15:4081. doi: 10.1038/s41467-024-47905-1 (PMC11094049; doi:10.1038/s41467-024-47905-1)
Supplement: Supplementary file 1 — Supplementary Information [file 41467_2024_47905_MOESM1_ESM.pdf]

**Supplementary Appendix**

**Supplementary table 1 –** Study sites page 2

**Supplementary table 2 –** Exclusion criteria page 3

**Supplementary table 3 –** Primary COVID-19 case definitions page 5

**Supplementary figure 1 –** Days on which solicited local and systemic AEs occurred page 7

**Supplementary table 4 –** All reported COVID-19 cases page 8

**Supplementary figure 2 –** Kaplan-Meier curves of COVID-19 cases from Day 1 page 9

**Supplementary table 5 –** Identified SARS-CoV-2 variants in COVID-19 case in phase 3b page 10

**Supplementary table 6 –** VE endpoints against Delta (B.1.617.2) variant in phase 3b page 11

**Supplementary table 7 –** VE endpoints in phases 1, 2 and 3a combined page 11

**Study protocol**

**Statistical Analysis Plan**

| **Supplementary table 1 – study sites** | | |
|---|---|---|
| **Site no.** | **Institute** | **Location** |
| 1 | Clinical Pharmacology Center – Hanoi Medical University | Hanoi |
| 2 | Yên Phong District Medical Center | Bắc Ninh |
| 3 | Vĩnh Long District Medical Center | Tỉnh Vĩnh Long |
| 4 | Long Hồ District Medical Center – Tỉnh Vĩnh Long | Tỉnh Vĩnh Long |
| 5 | Cái Bè District Medical Center | Tỉnh Tiền Giang |
| 6 | Chợ Gạo District Medical Center | Tỉnh Tiền Giang |
| 7 | Cao Lãnh District Medical Center | Tỉnh Đồng Tháp |
| 8 | Lấp Vò District Medical Center | Tỉnh Đồng Tháp |
| 9 | Văn Lâm District Medical Center | Hưng Yên Province |
| 10 | Văn Giang District Medical Center | Hưng Yên Province |
| 11 | Phù Cừ District Medical Center | Hưng Yên Province |
| 12 | Tiên Lữ District Medical Center | Hưng Yên Province |
| 13 | Kim Động District Medical Center | Hưng Yên Province |
| 14 | Ân Thi District Medical Center | Hưng Yên Province |
| 15 | Khoái Châu District Medical Center | Hưng Yên Province |
| 16 | Hưng Yên District Medical Center | Hưng Yên Province |

**Supplementary table 2:** Exclusion Criteria

Individuals meeting any of the following criteria were excluded from the study:

**Medical Conditions**

1. Significant infection or other acute illness, including body temperature >100.4°F (>38.0°C) on the day prior to or Day 1. Participants meeting this criterion could be rescheduled within the relevant window periods (Section 5.2.1). Afebrile participants with minor illnesses could be enrolled at the discretion of the investigator.
2. Pregnant or breastfeeding.
3. Known history of COVID-19 (nucleocapsid positive test is not exclusionary) or positive nasal swab SARS-CoV-2 by RT-PCR test. Note: if obtained within 5 days prior to Screening period, there was no need to repeat RT-PCR at Screening.
4. Close contact with a person known to be SARS-CoV-2 positive or with a clinical diagnosis of COVID-19 within 7 days prior to enrolment. Participants meeting this criterion who remained asymptomatic for 7 days could be rescheduled for enrolment within the relevant window periods (Section 5.2.1).
5. Known history of anaphylaxis, urticaria, or other significant adverse reaction to the vaccine or its excipients as listed in the Investigator's Brochure.
6. Known history of anaphylaxis to other vaccines.
7. Bleeding disorder considered a contraindication to intramuscular (IM) injection or phlebotomy.
8. Immunosuppressive or immunodeficient state, asplenia, recurrent severe infections, or known to be HIV positive.
9. An underlying clinically significant acute or chronic medical condition or physical examination findings for which, in the opinion of the investigator, participation would not be in the best interest of the participant (e.g., compromise the well-being) or that could prevent, limit, or confound the protocol-specified assessments.

**Prior/Concomitant Therapy**

10. Had previously received investigational or approved MERS-CoV, SARS-CoV, SARS-CoV-2 vaccines or who had plans to receive off-study COVID-19 vaccines.
11. Had received a live replicating vaccine within 28 days prior to each study vaccination or a licensed inactivated or non-replicating vaccine within 14 days prior to first study vaccination.
12. Had received treatment with immunosuppressive therapy, including cytotoxic agents or systemic corticosteroids, e.g., for cancer or an autoimmune disease, within 6 months prior to Screening, or planned receipt throughout the study. If systemic corticosteroids had been administered short term (<14 days) for treatment of an acute illness, participants should not be enrolled into the study until corticosteroid therapy had been discontinued for at least 28 days prior to first study vaccine administration. Inhaled/nebulised, intra-articular, intrabursal, or topical (skin or eyes) corticosteroids are permitted.
13. Had received systemic immunoglobulins or blood products within 3 months prior to first study vaccine administration or planned to receive such products during the study.

**Other Exclusions**

14. Demonstrated inability to comply with the study procedures.
15. Investigator site staff members, employees of the Sponsor or the CRO directly involved in the conduct of the study, or site staff members otherwise supervised by the investigator, or immediate family members of any of the previously mentioned individuals.

**Additional Exclusion Criteria for Phase 1 Participants Only**

16. Individuals ≥ 60 years of age.
17. Individuals with clinically significant abnormalities in medical history or physical examination, including but not limited to, the following:
    a) Respiratory disease (e.g., chronic obstructive pulmonary disease [COPD], asthma) requiring daily medications or oxygen currently or any treatment of respiratory disease exacerbations (e.g., COPD or asthma exacerbation) warranting hospitalization or an emergency room visit or supplemental oxygen in the last 5 years.
    b) Significant cardiovascular disease (e.g., congestive heart failure, cardiomyopathy, ischemic heart disease) or history of myocarditis or pericarditis as an adult.
    c) Neurological or neurodevelopmental conditions (e.g., migraines, epilepsy, stroke, seizures in the last 3 years, encephalopathy, focal neurologic deficits, Guillain-Barré syndrome, encephalomyelitis or transverse myelitis).
    d) Significant hematologic abnormalities (e.g., sickle cell disease, beta thalassemia, coagulation disorders).
    e) Major surgery within the past 6 months.
    f) Individuals with a history of chronic liver disease.
18. Individuals with the following abnormal Screening laboratory results:
    a) Positive nasal swab SARS-CoV-2 by RT-PCR test. Note: if obtained within 5 days prior to Screening period, do not need to repeat RT-PCR at Screening.
    b) Alanine aminotransferase (ALT), aspartate aminotransferase (AST), gamma-glutamyl transferase (GGT), total bilirubin, or alkaline phosphatase >1.5 upper limit of normal.
    c) Haemoglobin <9.5 g/dL for females and <10.5 g/dL for males.
    d) Platelet count <100 × 109/L.
    e) Other clinically significant abnormal (according to the judgment of the Investigator) Screening laboratory values.
    f) Individuals with Type 2 diabetes and Screening haemoglobin A1c >8.0%.
    g) Hepatitis B core antigen (HbsAg), Hepatitis C virus (HCV) antibody or HIV positive.

| **Supplementary table 3:** Primary COVID-19 case definitions based on FDA recommendation * | | | |
|---|---|---|---|
| **Case definition** | **Laboratory finding** | **Clinical status** | |
| | | **Symptoms** | **Other clinical parameters** |
| Uninfected | No +ve SARS-CoV-2 test | None | None relevant |
| Asymptomatic SARS-CoV-2 infection | +ve SARS-CoV-2 test | None | None relevant |
| Protocol-defined COVID-19 | +ve SARS-CoV-2 test | At least one of the following that is a NEW or WORSENING finding:<br>• Fever or chills<br>• Cough<br>• Shortness of breath or difficulty breathing<br>• Fatigue<br>• Muscle or body aches<br>• Headache<br>• New loss of taste or smell<br>• Sore throat<br>• Congestion or runny nose<br>• Nausea or vomiting<br>• Diarrhea | None relevant |
| Atypical COVID-19 | +ve SARS-CoV-2 test | Clinical findings suggestive of COVID-19 but not included above | None relevant |
| Severe COVID-19 | +ve SARS-CoV-2 test | As above for protocol-defined COVID-19 | Any of the following<br>• Clinical signs at rest indicative of severe systemic illness<br>○ Respiratory rate ≥ 30 per min<br>○ Heart rate ≥ 125 per min<br>○ $SpO_2$ ≤ 93% on room air at sea level or $PO_2/FiO_2$ < 300 mm Hg |

| | | | |
|---|---|---|---|
| | | | • Respiratory failure (needing high flow oxygen, noninvasive ventilation, mechanical ventilation or ECMO)<br>• Evidence of shock<br>  ○ SBP < 90 mm Hg, or<br>  ○ DBP < 60 mm Hg, or<br>  ○ requiring vasopressors<br>• Significant acute renal, hepatic, or neurologic dysfunction<br>• Admission to an ICU<br>• Death |

* Department of Health and Human Services (DHHS), Food and Drug Administration, Center for Biologics Evaluation and Research (US). Guidance Document. Development and Licensure of Vaccines to Prevent COVID-19. 2020. https://www.fda.gov/regulatory-information/search-fda-guidance-documents/development-and-licensure-vaccines-prevent-covid-19. Accessed 10 Mar 2021.

**Supplementary figure 1:**
Days post-vaccination on which local reactions and systemic adverse events were reported.

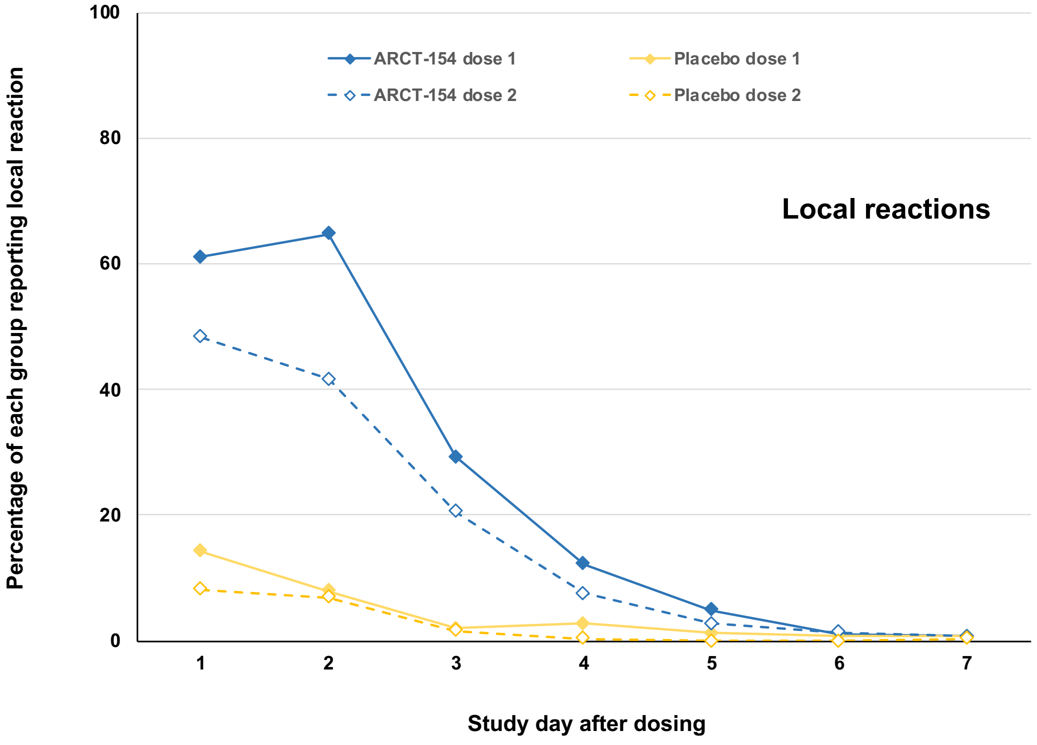


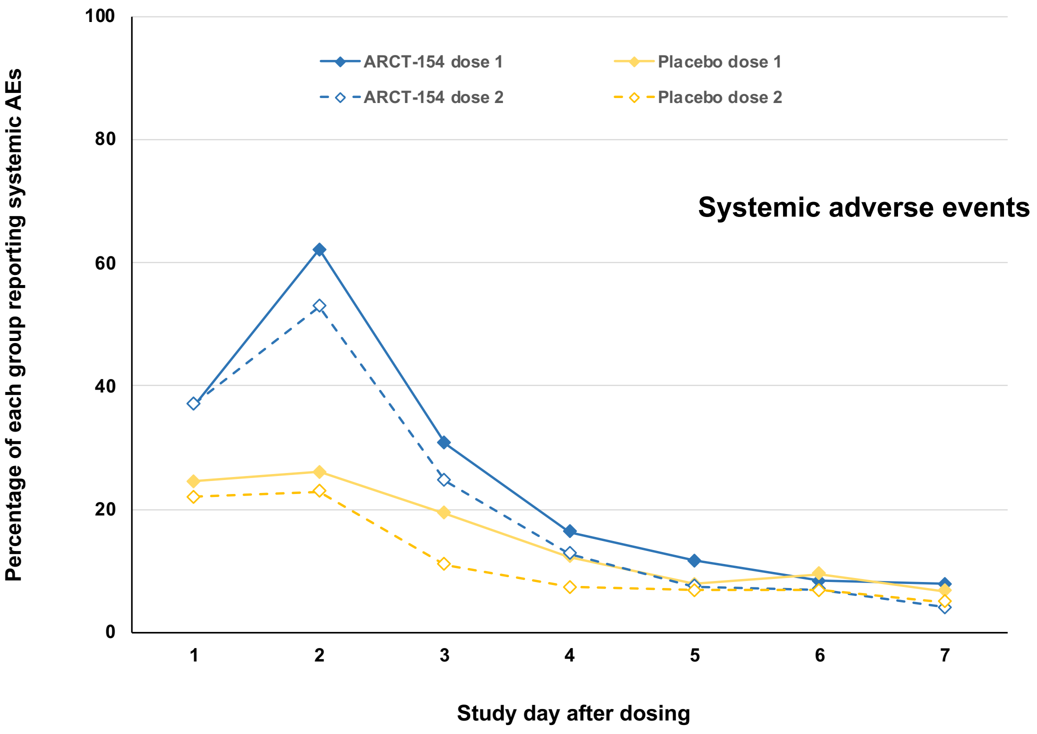

| **Supplementary table 4:** All reported COVID-19 cases | | | | |
|---|---|---|---|---|
| **Analysis Period** | **Number of Events** | **Phase 1/2/3a** | **Phase 3b** | **Total** |
| **Day 1 to Day 35** | Any protocol-confirmed COVID-19 | 1 | 44 | 45 |
| | Severe COVID-19 | 0 | 2 | 2 |
| | Death attributed to COVID-19 | 0 | 0 | 0 |
| **Day 36 to Day 92** | Any protocol-confirmed COVID-19 | 40 | 651 | 691 |
| | Severe COVID-19 | 2 | 44 | 46 |
| | Death attributed to COVID-19 | 0 | 10 | 10 |
| **Any** | Any protocol-confirmed COVID-19 | 50 | 734 | 784 |
| | Severe COVID-19 | 2 | 48 | 50 |
| | Death attributed to COVID-19 | 0 | 10 | 10 |
| | Asymptomatic SARS-CoV-2 infection | 30 | 102 | 132 |
| | Total | 80 | 836 | 916 |

**Supplementary figure 2:**
Cumulative incidence of COVID-19 of any severity (panel A), and severe COVID-19 (panel B) in vaccine and placebo from Day 1.

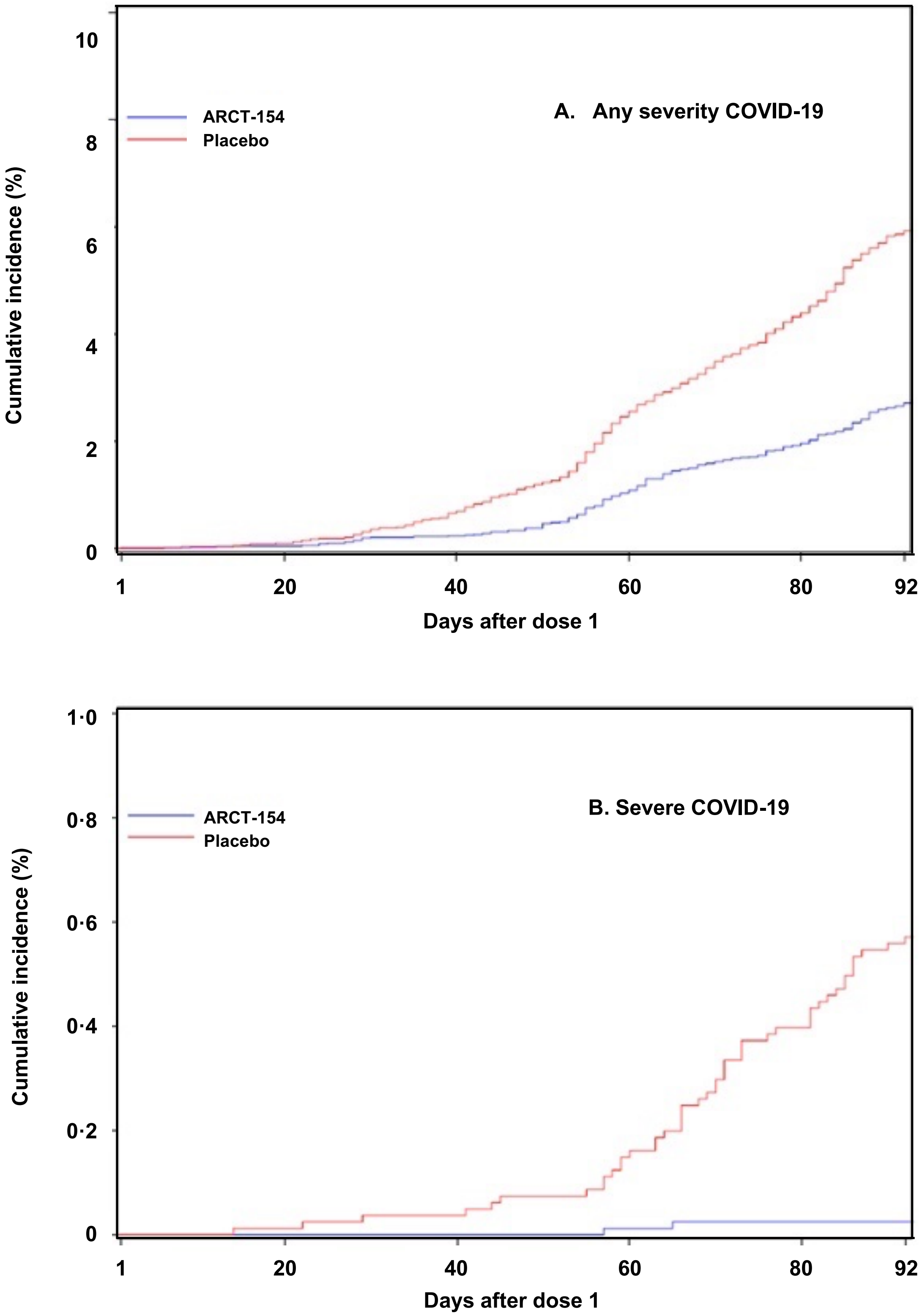

| **Supplementary table 5:** Identification of SARS-CoV-2 variants in virologically-confirmed, protocol-defined COVID-19 cases occurring between Days 36 and 92 in Phase 3b (mITT set) | | | |
|---|---|---|---|
| | **Total (N = 16100)** | **ARCT-154 (N = 8056)** | **Placebo (N = 8044)** |
| **Variant** | n (%) | n | n |
| **Alpha (B.1.1.7)** | 2 (0.3) | 0 | 2 |
| **Beta (B.1.351)** | 1 (0.2) | 0 | 1 |
| **Delta (B.1.617.2)** | 477 (74.2) * | 164 | 313 |
| **Omicron (B.1.1.529)** | 2 (0.3) | 1 | 1 |
| **Not determined** | 55 (8.6) * | 14 | 41 |
| **Missed** | 106 (16.5) | 21 | 85 |
| **Overall** | 643 (100) | 200 | 443 |

* Two Delta (B.1.617.2) cases and one case with an undetermined variant were censored due to receipt of a non-study COVID-19 vaccine before the onset of the episode.

| **Supplementary table 6:** Key secondary vaccine efficacy (VE) endpoints in phase 3b (mITT) against the Delta (B.1.617.2) SARS-CoV-2 variant | | | | | | | |
|---|---|---|---|---|---|---|---|
| | Total no. of persons | Cumulative follow-up in person-yrs | No. with event | Total no. of persons | Cumulative follow-up in person-yrs | No. with event | **Vaccine efficacy** (95% confidence intervals) |
| | | **ARCT-154** | | | **Placebo** | | |
| **Primary VE endpoints in the mITT population against Delta (B.1.617.2) variant SARS-CoV-2** | | | | | | | |
| **Any severity** protocol-confirmed COVID-19 (Days 36–92) | 7787 | 1131.7 | **164** | 7723 | 1100.6 | **311** | **49.8%** (39.3–58.4) |
| **Severe** protocol-confirmed COVID-19 (Days 36–92) | 7787 | 1148.2 | **1** | 7723 | 1134.8 | **17** | **94.3%** (57.4–99.2) |

| **Supplementary table 7:** Key secondary vaccine efficacy (VE) endpoints in the cumulative phase 1, 2 and 3a study population | | | | | | | |
|---|---|---|---|---|---|---|---|
| | Total no. of persons | Cumulative follow-up in person-yrs | No. with event | Total no. of persons | Cumulative follow-up in person-yrs | No. with event | **Vaccine efficacy** (95% confidence intervals) |
| | | **ARCT-154** | | | **Placebo** | | |
| **VE endpoints in the phase 1, 2 and 3a mITT population** | | | | | | | |
| **Any severity** protocol-confirmed COVID-19 (Days 36–92) | 729 | 120.8 | **23** | 244 | 39.6 | **17** | **56.3%** (18.2–76.7) |
| **VE endpoints in the phase 1, 2 and 3a ITT population** | | | | | | | |
| **Any severity** protocol-confirmed COVID-19 (Days 1–92) | 749 | 195.5 | **23** | 252 | 64.6 | **18** | **58.9%** (23.8–77.8) |

# A Randomized, Observer-Blind, Controlled Study to Assess the Safety, Immunogenicity and Efficacy of the SARS-CoV-2 Self-Amplifying RNA Vaccine ARCT-154 in Adults

## PROTOCOL NO. ARCT-154-01

| | |
|---|---|
| **Sponsor:** | Vinbiocare Biotechnology Joint Stock Company<br>Techno Park building, Vinhomes Ocean Park Residential Area, Da Ton ward, Gia Lam District,<br>Hanoi, Vietnam |
| **Version of Protocol:** | 9.0 |
| **Date of Protocol:** | 07 February 2022 |
| **Previous Version:** | 8.0 |
| **Investigational Product(s):** | ARCT-154 |
| **Study Phase:** | 1/2/3 |

**Confidentiality Statement**

All financial and nonfinancial support for this study will be provided by Vinbiocare Biotechnology Joint Stock Company (the Sponsor). This document contains information that is confidential, proprietary, and/or under the control of the Sponsor. The information is only to be used in connection with authorized clinical studies of the investigational drug described in the protocol provided herewith. You may disclose the contents of this document while acting in the capacity of an investigator, potential investigator, or consultant to study personnel under your supervision and/or to an applicable Institutional Review Board or Independent Ethics Committee who need to know the contents for the aforementioned purpose and who have been advised of the confidential nature of the document. You may not disclose any of the information to others without written authorization from the Sponsor except to the extent necessary to obtain informed consent from those persons to whom the drug may be administered.

# SPONSOR'S APPROVAL

| | |
|---|---|
| **Title** | A Randomized, Observer-Blind, Controlled Study to Assess the Safety, Immunogenicity and Efficacy of the SARS-CoV-2 Self-Amplifying RNA Vaccine ARCT-154 in Adults<br>The ARCT-154 Self-Amplifying RNA Vaccine Study |
| **Protocol Number** | ARCT-154-01 |
| **Version Number** | 9.0 |
| **Version Date** | 07 February 2022 |
| **Amendment** | Amendment 8 |

The design of this study as outlined by this protocol has been reviewed and approved by the Sponsor's responsible personnel.

Protocol accepted and approved on behalf of Sponsor by:

______________________________ ______________________________

**Signature** **Date**

[redacted] [redacted]

**Name** **Title**

VINBIOCARE BIOTECHNOLOGY JOINT STOCK COMPANY
Techno Park building, Vinhomes Ocean Park Residential Area, Da Ton ward, Gia Lam District,
Hanoi, Vietnam

**Institution**

# TABLE OF CONTENTS

SPONSOR'S APPROVAL ... 2
TABLE OF CONTENTS ... 3
LIST OF TABLES ... 9
LIST OF FIGURES ... 10
LIST OF APPENDICES ... 10
LIST OF ABBREVIATIONS ... 11
1 SYNOPSIS ... 13
1.1 Study Schematic ... 36
2 INTRODUCTION ... 38
2.1 Background ... 38
2.2 Current Therapies for COVID-19 ... 38
2.3 Therapeutic Rationale for ARCT-154 in Prevention of COVID-19 ... 39
2.4 Mechanism of Action ... 40
2.5 Nonclinical Experience with ARCT-154 ... 41
2.6 Clinical Trial Experience with the LUNAR COVID-19 Vaccines (ARCT-021 and ARCT-154) ... 42
2.7 Rationale for Dose Regimen Selection ... 44
2.8 Risk: Benefit Assessment ... 44
2.8.1 Potential Risks ... 44
2.8.2 Potential Benefits ... 46
2.8.3 Overall Risk: Benefit Assessment ... 46
3 OBJECTIVES AND ENDPOINTS ... 47
3.1 Phase 1/2/3a Substudy ... 47
3.1.1 Phase 1/2/3a Substudy Primary Objectives and Endpoints ... 47
3.1.2 Phase 1/2/3a Substudy Secondary Objectives and Endpoints ... 49
3.1.3 Phase 1/2/3a Substudy Exploratory Objectives and Endpoints ... 49
3.2 Phase 3b ... 52
3.2.1 Phase 3b Primary Objectives and Endpoints ... 52
3.2.2 Phase 3b Secondary Objectives and Endpoints ... 52
3.2.3 Phase 3b Exploratory Objectives and Endpoints ... 53

3.3 Phase 3c Substudy ..... 54
3.3.1 Phase 3c Substudy Primary Objectives and Endpoints ..... 54
3.3.2 Phase 3c Substudy Secondary Objectives and Endpoints ..... 55
3.3.3 Phase 3c Substudy Exploratory Objectives and Endpoints ..... 57
3.4 Pooled Objectives and Endpoints ..... 59
3.4.1 Pooled Primary Objectives and Endpoints ..... 59
3.4.2 Pooled Secondary Objectives and Endpoints ..... 60
3.4.3 Pooled Exploratory Objectives and Endpoints ..... 60
4 INVESTIGATIONAL PLAN ..... 62
4.1 Number of Participants ..... 62
4.2 Number of Sites ..... 62
4.3 Duration of Participation ..... 62
4.4 Study Design ..... 62
4.5 Study Assessments and Procedures ..... 66
4.5.1 Informed Consent ..... 66
4.5.2 Randomization and Enrollment ..... 66
4.5.3 Visits ..... 67
4.5.3.1 Screening Visit (Up to 10 days prior to Day 1) ..... 69
4.5.3.2 Vaccine Dose Administration Visits ..... 69
4.5.3.3 Follow-up Visit on Day 8 and 36 (Phase 1 only) ..... 71
4.5.3.4 Follow-up Visit on Day 57 ..... 71
4.5.3.5 Follow-up Visit on Day 211 (Phase 3c-1 only) ..... 71
4.5.3.6 Weekly and Monthly Study Calls ..... 71
4.5.3.7 Unscheduled Visits ..... 72
4.5.3.8 Final Visit/Early Termination Visit ..... 74
4.5.4 End of Study ..... 74
4.5.5 Additional Procedural Specifics ..... 74
4.5.5.1 Diary Use to Collect Solicited Adverse Events ..... 74
4.5.5.2 Collection of Unsolicited Adverse Events and COVID-19 Exposure/Symptoms ..... 75
4.5.5.3 Blood and SARS-CoV-2 Sampling ..... 75
4.5.5.4 Telephone Calls ..... 78

4.5.5.5 Physical Examinations ..... 79
4.5.5.6 Height and Weight ..... 79
4.5.5.7 Vital Signs, Body Temperature, and Pulse Oximetry ..... 79
4.5.5.8 Pregnancy Testing ..... 79
4.5.5.9 Medical History ..... 80
5 PARTICIPANT SELECTION AND WITHDRAWAL CRITERIA ..... 81
5.1 Selection of Study Participants ..... 81
5.1.1 Inclusion Criteria ..... 81
5.1.2 Exclusion Criteria ..... 81
5.1.3 Determination of "At Risk" of Severe COVID-19 ..... 83
5.1.4 Screen Failures ..... 84
5.1.5 Switchover/Further Study Vaccine Eligibility Criteria ..... 84
5.2 Delaying or Discontinuing Study Vaccination and Participant Withdrawal From the Study ..... 85
5.2.1 Delay in Study Vaccination ..... 85
5.2.2 Discontinuation of Study Vaccine (Participant) ..... 86
5.2.3 Withdrawal/Discontinuation from the Study ..... 86
5.2.4 Discontinuation of Study Dosing (Study-wide) ..... 88
5.3 Lost to Follow-Up ..... 88
5.4 Replacement of Participants ..... 88
6 STUDY TREATMENTS ..... 89
6.1 Method of Assigning Participants to Study Groups ..... 89
6.2 Study Vaccine Manufacturer ..... 89
6.3 Study Vaccine, Dosage, and Route of Administration ..... 89
6.4 Management of Clinical Supplies ..... 90
6.4.1 Investigational Product Packaging and Storage ..... 90
6.4.2 Clinical Supplies for Reconstitution and Administration of Study Vaccine ..... 91
6.4.3 Study Vaccine Accountability ..... 91
6.4.4 Other Supplies for Participant Use ..... 91
6.5 Overdose Management ..... 91
6.6 Blinding ..... 92
6.6.1 Breaking the Blind ..... 93

6.7 Compliance With Study Treatment ... 93
6.8 Concomitant Therapies and Procedures ... 93
6.8.1 Concomitant Therapy ... 93
6.8.1.1 Concomitant Procedures ... 94
6.9 Dose Modification ... 94
6.10 Intervention After the End of the Study ... 94
7 STUDY ASSESSMENTS ... 95
7.1 Efficacy Assessments ... 95
7.1.1 Evaluation of Participants With Suspected COVID-19 ... 95
7.1.1.1 Severe COVID-19 ... 95
7.1.1.2 Asymptomatic COVID-19 ... 95
7.1.1.3 Atypical COVID-19 ... 96
7.1.1.4 Death Attributed to COVID-19 ... 96
7.2 Immunogenicity Assessments ... 96
7.3 Reactogenicity ... 98
7.4 Safety Assessments ... 99
7.4.1 Adverse Events ... 99
7.4.1.1 Definitions ... 99
7.4.1.2 Eliciting and Documenting Adverse Events ... 102
7.4.1.3 Reporting Adverse Events ... 104
7.4.2 Physical Examinations ... 106
7.4.3 Vital Signs, Body Temperature, and Pulse Oximetry ... 106
7.5 Pregnancy ... 106
8 STATISTICAL CONSIDERATIONS ... 107
8.1 Overall Endpoint Testing Strategy ... 107
8.2 Analysis Sets ... 108
8.3 Statistical Considerations for Phase 3 ... 110
8.3.1 Estimands and Intercurrent Events for Phase 1/2/3a, Phase 3b and Phase 3c ... 112
8.4 Statistical Hypothesis ... 117
8.4.1 Phase 1/2/3a Substudy Statistical Hypothesis ... 117
8.4.2 Phase 3b Statistical Hypothesis ... 117

8.4.3 Phase 3c Substudy Statistical Hypotheses ... 118
8.4.4 Pooled Analyses Statistical Hypotheses ... 119
8.5 Sample Size Determination ... 119
8.5.1 Sample Size for Phase 1/2/3a Substudy ... 119
8.5.2 Sample Size for Phase 3b ... 119
8.5.3 Sample Size for Phase 3c Substudy ... 120
8.6 Description of Additional Subgroups to Be Analyzed ... 120
8.7 Statistical Analysis Methodology ... 121
8.7.1 General Considerations ... 121
8.7.1.1 General Considerations for Phase 1/2/3a Substudy ... 121
8.7.1.2 General Considerations for Phase 3b ... 121
8.7.1.3 General Considerations for Phase 3c Substudy ... 122
8.7.2 Overview of Statistical Methods: Estimation of Estimands and Sensitivity Analyses ... 122
8.7.3 Analysis of Primary Endpoints for Phase 1/2/3a, Phase 3b and Phase 3c ... 127
8.7.3.1 Main Estimation of Primary Immunogenicity Endpoint for Phase 1/2/3a Substudy (Estimand 1) ... 127
8.7.3.2 Main Estimation of Primary Safety Endpoint for Phase 1/2/3a, 3b, and 3c (Estimand 3 & 4) ... 128
8.7.3.3 Supportive Analysis of Primary Safety Endpoints for Phase 1/2/3a, 3b, and 3c (Estimand 3 & 4) ... 128
8.7.3.4 Main Estimation of Phase 3b Primary Efficacy Endpoint (Estimand 2) ... 128
8.7.4 Analysis of Secondary Endpoints for Phase 1/2/3a, Phase 3b and Phase 3c ... 129
8.7.4.1 Main Estimation of Secondary Immunogenicity Endpoints for Phase 1/2/3a and Phase 3c Substudies ... 129
8.7.4.2 Main Estimation of Secondary Efficacy Endpoints for Phase 3b ... 129
8.7.5 Analysis of Pooled Efficacy and Safety Endpoints ... 129
8.7.6 Analyses of Other Exploratory Endpoints ... 129
8.7.7 Other Analyses ... 129
8.7.8 Analyses of Immunogenicity, Safety and Efficacy Data Post Day 92 (Phase 1//2/3a, Phase 3b and Pooled Cohorts) ... 129
8.8 Handling of Missing Data ... 130
8.9 Primary and Interim Analyses Timepoints ... 130

8.9.1 Phase 1/2/3a Substudy ... 130
8.9.2 Phase 3b ... 130
8.9.3 Phase 3c Substudy ... 130
8.10 Monitoring Committees ... 131
8.10.1 Blinded Safety Review Committee ... 131
8.10.2 Blinded Independent Adjudication Committee ... 131
8.10.3 Unblinded Independent Data and Safety Monitoring Board ... 131
9 DATA QUALITY ASSURANCE ... 132
9.1 Data Management ... 132
9.2 Data Disclosure ... 132
10 ETHICS ... 133
10.1 Independent Ethics Committee or Institutional Review Board ... 133
10.2 Ethical Conduct of the Study ... 133
10.3 Participant Information and Consent ... 133
11 INVESTIGATOR'S OBLIGATIONS ... 135
11.1 Confidentiality ... 135
11.2 Data Protection ... 135
11.3 Financial Disclosure and Obligations ... 136
11.4 Investigator Documentation ... 136
11.5 Study Conduct ... 136
11.6 Adverse Events and Study Report Requirements ... 136
11.7 Investigator's Final Report ... 136
11.8 Records Retention ... 136
12 STUDY MANAGEMENT ... 137
12.1 Monitoring ... 137
12.1.1 Monitoring of the Study ... 137
12.1.2 Inspection of Records ... 137
12.2 Management of Protocol Amendments and Deviations ... 137
12.2.1 Modification of the Protocol ... 137
12.2.2 Protocol Deviations ... 137
12.3 Study Termination ... 138

12.4 Final Report ... 138
13 REFERENCES ... 139
14 APPENDICES ... 147
Protocol Amendment 1 (Version 2.0, 27 July 2021): Summary of Major Changes ... 171
Protocol Amendment 2 (Version 3.0, 01 August 2021): Summary of Major Changes ... 173
Protocol Amendment 3 (Version 4.0, 09 August 2021): Summary of Major Changes ... 176
Protocol Amendment 4 (Version 5.0, 24 August 2021): Summary of Major Changes ... 178
Protocol Amendment 5 (Version 6.0, 28 October 2021): Summary of Major Changes ... 180
Protocol Amendment 5 (Version 6.0, 28 October 2021): Summary of Major Changes ... 182
Protocol Amendment 6 (Version 7.0, 28 October 2021): Summary of Major Changes ... 184
Protocol Amendment 7 (Version 8.0, 14 December 2021): Summary of Major Changes ... 186
Protocol Amendment 8 (Version 9.0, XX February 2022): Summary of Major Changes ... 190

# LIST OF TABLES

Table 1 Risk Minimization Measures Included in Clinical Trials of LUNAR COVID-19 Vaccines ... 45
Table 2 Phase 1/2/3a Substudy Primary Objectives and Endpoints ... 48
Table 3 Phase 1/2/3a Secondary Objectives and Endpoints ... 49
Table 4 Phase 1/2/3a Exploratory Objectives and Endpoints ... 50
Table 5 Phase 3b Primary Objectives and Endpoints ... 52
Table 6 Phase 3b Secondary Objectives and Endpoints ... 52
Table 7 Phase 3b Exploratory Objectives and Endpoints ... 53
Table 8 Phase 3c Substudy Primary Objectives and Endpoints ... 55
Table 9 Phase 3c Substudy Secondary Objectives and Endpoints ... 55
Table 10 Phase 3c Substudy Exploratory Objectives and Endpoints ... 57
Table 11 Pooled Primary Objectives and Endpoints ... 59
Table 12 Pooled Secondary Objectives and Endpoints ... 60
Table 13 Pooled Exploratory Objectives and Endpoints ... 60
Table 14 Intercurrent Event Types ... 112
Table 15 Estimands for Phase 1/2/3a Substudy and Phase 3c Substudy Primary, Secondary and Exploratory Immunogenicity Endpoints ... 112
Table 16 Estimands for Primary, Secondary and Exploratory Efficacy Analyses ... 114

Table 17 Estimands for Primary Safety Analyses for Phase 1/2/3a, Phase 3b, Phase 3c and Pooled Cohorts ........ 115
Table 18 Summary of Statistical Methods, Including Sensitivity Analyses ........ 123
Table 19 Toxicity Grading for Solicited Adverse Events ........ 167

# LIST OF FIGURES

Figure 1 Study Schematic ........ 37
Figure 2 sVNT Antibody Responses to ARCT-154 in Non-Human Primates ........ 41
Figure 3 Pseudoneutralization Data from NHPs After Two Doses of ARCT-154 Vaccine ........ 42

# LIST OF APPENDICES

Appendix 1: Schedule of Assessments ........ 148
Appendix 2: Case Definitions of COVID-19 ........ 160
Appendix 3: Contraceptive Guidance ........ 164
Appendix 4: Toxicity Grading Scales ........ 167
Appendix 5: At-risk Medical Conditions ........ 169
Appendix 6: Protocol Amendments ........ 171

# LIST OF ABBREVIATIONS

| Abbreviation | Definition |
|---|---|
| Ab | antibody |
| AC | Adjudication Committee |
| ACE-2 | angiotensin-converting enzyme 2 |
| AE | adverse event |
| AESI | adverse event of special interest |
| ARDS | acute respiratory distress syndrome |
| ART | antiretroviral therapy |
| BAb | binding antibody |
| ChAdOx1 | Astra Zeneca COVID_19 vaccine ChAdOx1 nCoV-19 used as comparator for Phase 3c |
| CFR | US Code of Federal Regulations |
| CI | confidence interval |
| CLIA | Clinical Laboratory Improvement Amendment |
| COVID-19 | Coronavirus disease 2019 |
| CRO | clinical research organization |
| CSR | clinical study report |
| DSMB | Data and Safety Monitoring Board |
| eCRF | electronic case report form |
| EDC | electronic data capture |
| eDiary | electronic diary |
| EOS | end of study |
| ET | early termination |
| FDA | US Food and Drug Administration |
| FSH | follicle-stimulating hormone |
| GCP | Good Clinical Practice |
| HCP | healthcare provider |
| HIV | human immunodeficiency virus |
| HR | hazard ratio |
| HRT | hormone replacement therapy |
| IAS | immunogenicity analysis set |
| IB | Investigator’s Brochure |
| IcEv | intercurrent event |
| ICF | informed consent form |
| ICH | International Council for Harmonisation |
| ICS | intracellular cytokine staining |
| IEC | Independent Ethics Committee |
| IM | intramuscular |

| **Abbreviation** | **Definition** |
|---|---|
| IRB | Institutional Review Board |
| IRT | interactive response technology |
| ISO | International Organization for Standardization |
| LNP | lipid nanoparticle |
| MAAE | medically attended adverse event |
| MedDRA | Medical Dictionary for Regulatory Activities |
| MERS | Middle East respiratory syndrome |
| mITT | modified intent-to-treat |
| mRNA | messenger ribonucleic acid |
| MOH | Ministry of Health |
| NAAT | nucleic acid amplification–based test |
| NAb | neutralizing antibody |
| NHP | non-human primate |
| nsP | nonstructural protein |
| PCR | polymerase chain reaction |
| PP | per protocol |
| PRNT50 | plaque reduction neutralization test at 50% reduction |
| RAS | reactogenicity analysis set |
| RBD | receptor binding domain |
| RNA | ribonucleic acid |
| RT-PCR | reverse transcriptase-polymerase chain reaction |
| S glycoprotein | spike glycoprotein |
| SAE | serious adverse event |
| SAP | statistical analysis plan |
| SARS-CoV-2 | severe acute respiratory syndrome coronavirus 2 |
| SAS | safety analysis set |
| S.C | subcutaneous |
| $SC^{154}$ | proportion of ARCT-154 participants that seroconvert |
| $SC^{ChAdOx1}$ | proportion of ChAdOx1participants that seroconvert |
| $SC^{Placebo}$ | proportion of placebo participants that seroconvert |
| SRC | Safety Review Committee |
| SUSAR | suspected unexpected serious adverse reaction |
| sVNT | surrogate virus neutralization |
| Tfh | T follicular helper |
| VAERD | vaccine-associated enhanced respiratory disease |
| VE | vaccine efficacy |
| VOC | variants of concern |
| VSV | vesicular stomatitis virus |
| WHO | World Health Organization |

# 1 SYNOPSIS

| | |
|---|---|
| **Title** | A Randomized, Observer-Blind, Controlled Study to Assess the Safety, Immunogenicity and Efficacy of the SARS-CoV-2 Self-Amplifying RNA Vaccine ARCT-154 in Adults |
| **Short Title** | The ARCT-154 Self-Amplifying RNA Vaccine Study |
| **Phase** | 1/2/3 |
| **Study Design** | This is a Phase 1/2/3, randomized, controlled, observer-blind study designed to evaluate the safety, immunogenicity and efficacy of ARCT-154 in adult participants to be enrolled in Vietnam. This study design has been updated to include a cohort (Phase 3c) to evaluate the immunogenicity, safety, and efficacy of ARCT-154 versus a comparator COVID-19 vaccine.<br><br>The study is divided into five parts: Phase 1, Phase 2, Phase 3a, Phase 3b and Phase 3c.<br><br>Participants in Phase 1 and 3b will be randomly assigned to a study group that will receive up to 2 vaccination series (4 doses of study vaccine total). Each vaccination series comprises two vaccinations at 28-day intervals: an initial vaccination series with vaccinations on Day 1 and Day 29 and an additional two-dose vaccination series with the opposite vaccine on Day 92 and Day 120, which is at the 2-month timepoint after the second dose (referred to as the “Switchover” vaccination series throughout the protocol). Each participant in Phase 1 and 3b is planned to receive a two-dose vaccination series of ARCT-154 at a dose of 5 µg and a two-dose vaccination series of placebo (saline). Therefore, the study group that initially received two doses of ARCT-154 will receive two doses of placebo on Switchover and study group that initially received placebo will receive two doses of ARCT-154 5 µg on Switchover (Figure 1).<br><br>Participants in Phase 2 and 3a will be randomly assigned to a study group that will receive two vaccinations with either ARCT-154 or placebo at a 28-day interval with vaccinations on Day 1 and Day 29. With protocol version 8.0, participants that received ARCT-154 in the initial two-dose vaccination series will be further randomized to receive either ARCT-154 or placebo (in a 3:1 ratio) at Day 92 (referred to as “Further Study Vaccine” throughout the protocol) followed by placebo at Day 120. Participants that received Placebo in the initial vaccination series will undergo switch over to receive ARCT-154 at Day 92 and Day 120.<br><br>Participants in Phase 3c will be randomized to receive either ARCT-154 or Astra Zeneca COVID-19 vaccine (ChAdOx1 nCoV-19; referred to as ChAdOx1from here on) which has received authorization for use in Vietnam. Participants in Phase 3c will receive a 2-dose priming vaccination series of either ARCT-154 5 µg or ChAdOx1, with vaccinations being administered on Day 1 and Day 29. Because all participants in Phase 3c will receive an active vaccine, no switchover will occur for these participants.<br><br>Phase 1 is a sentinel cohort for assessment of safety. 100 healthy participants will be randomly assigned 3:1 to receive ARCT-154 or placebo (75:25) for the initial series of vaccinations. The Safety Review Committee (SRC) and Vietnam Ministry of Health (MOH) will independently review available blinded safety data for Phase 1 participants through at least 7 days post-second vaccination (Day 36) and, if study vaccination is judged to be |

| | |
|---|---|
| | adequately tolerated by SRC and the MOH, the study will initiate simultaneous enrollment of both Phase 2 and Phase 3a.<br><br>In Phase 2, which expands the safety and immunogenicity characterization of the study vaccine in a larger group of participants, 300 participants will be randomly assigned 3:1 to receive ARCT-154 or placebo (225:75) for the initial series of vaccinations.<br><br>In Phase 3a, which expands the enrollment further to include 600 participants who will be randomly assigned 3:1 to receive ARCT-154 or placebo (450:150) for the initial series of vaccinations. After completion of enrollment of Phases 2 and 3a, the SRC and MOH will independently review available blinded safety data through at least 7 days (Day 8) post-first vaccination and, if study vaccination is judged to be adequately tolerated by SRC and the MOH, the study will initiate enrollment of Phase 3b. The second dose of study vaccine will not be administered to participants in Phase 3b until the SRC has reviewed the Phases 2 and 3a safety data through 7 days post-second vaccination (Day 36) and judged that study vaccine is adequately tolerated. Data from participants in Phase 1, Phase 2, and Phase 3a will be pooled for a primary analysis of immunogenicity at Day 57. Data from this analysis will be submitted to the Vietnamese Ministry of Health for evaluation for potential emergency use authorization (EUA). Data from Phase 2 and 3a will also be pooled to allow comparison of immunogenicity following the 3rd dose (immune responses at Day 120 with that after the 2nd dose (immune responses at Day 57).<br><br>In Phase 3b, approximately 16,000 participants will be randomly assigned 1:1 to receive ARCT-154 or placebo for the initial series of vaccinations. Phase 3b constitutes the Exploratory Efficacy Subgroup. Data from this subgroup will support regulatory filings along with immunogenicity data from Phase 1/2/3a and 3c. Participants in this Phase 3b portion of the study will contribute substantially to the safety analyses of ARCT-154.<br><br>Phase 3c will enroll approximately 2,400 participants randomized 1:1 to receive ARCT-154 or ChAdOx1. A subset of these enrolled participants (the first 1,500 enrolled in Phase 3c) will form an Immunogenicity Noninferiority Subgroup (Phase 3c-1). Day 57 data from 800 participants in this Subgroup will be used to establish the noninferiority of immunogenicity of ARCT-154 vaccine as compared to ChAdOx1, a licensed comparator vaccine. These data, along with an analysis of safety from participants exposed to ARCT-154 and followed for a median period of 2 months (60 days) may be submitted to health authorities for evaluation for a Marketing Authorization or EUA dependent on jurisdiction.<br><br>Phase 1 participants must be <60 years of age. Phase 2, 3a, 3b and Phase 3c participants will include elderly (≥60 years) participants as well. Phase 3b will enrich for participants at higher risk for COVID-19 by attempting to enroll individuals considered at high risk due to their workplace environment or living conditions.<br><br>For Phase 2, 3a, 3b and Phase 3c, prior to randomization, participants will be stratified by age (< 60 or ≥ 60 years of age) and for participants < 60 years of age by risk of severe COVID-19. Participants will be defined as "at risk" if they are 60 years of age or older OR have medical history described as putting the individual at risk or possibly at risk of severe coronavirus disease 2019 (COVID-19) (Appendix 5). As the eligibility criteria for Phase 1 |

| | |
|---|---|
| | exclude participants ≥ 60 years of age or otherwise at risk for severe COVID-19, stratification will not occur for Phase 1.<br><br>Participants in all study phases will undergo informed consent and screening procedures prior to enrollment. After enrollment, participants will be randomized to groups. Study vaccines will be given in a supervised medical setting. All participants will be evaluated for reactogenicity (solicited adverse events) and reporting of unsolicited adverse events and concomitant medications through a series of clinic visits and phone calls. Depending on the study phase, blood sampling for immunogenicity and testing for COVID-19 may be performed. See Appendix 1 for an overview of study visits and assessments for all phases of the study.<br><br>For participants in Phase 1/2/3a/3b, at the Day 92 visit and prior to receipt of further study vaccine, participants will be evaluated by the Investigator according to Further Study Vaccine Eligibility criteria (Section 5.1.5). Any participant who does not meet these criteria will not receive further vaccinations with study vaccine and will be asked to continue in the study through Final Visit (Day 394).<br><br>For participants that receive further doses of study vaccine at Day 92 and Day 120, this will occur in a blinded fashion in that participants and blinded site and Sponsor personnel will remain blinded to treatment assignment until End of Study (EOS).<br><br>Participants who choose to receive vaccination with off-study COVID-19 vaccine will be encouraged to remain in the study, but those that choose to leave the study to pursue alternate vaccination will not be unblinded to study treatment unless there is an unexpected post study SAE attributed as related to study vaccine by the study investigator.<br><br>Data will be collected through a combination of clinic visits and use of electronic diaries (and paper diaries in circumstances where electronic diaries are not feasible) to capture solicited adverse events and to prompt for symptoms of COVID-19 and risk of SARS-CoV-2 exposure, other adverse events, and concomitant medications. All participants enrolled in Phase 1 and Phase 2 must have access to the eDiary to facilitate rapid review of safety data by the SRC.<br><br>Exposure to SARS-CoV-2 is defined as a participant who has had close contact (within 6 feet [2 meters] to a person with virologically confirmed COVID-19 during the active transmission period.<br><br>The active transmission period is:<br>1) for a symptomatic COVID-19 case, 3 days before the first symptom or<br>2) for an asymptomatic COVID-19 case, from 14 days prior to the positive COVID-19 test result.<br><br>The close contact definition applies regardless of whether the person with COVID-19 or the contact was wearing a mask or whether the contact was wearing respiratory personal protective equipment. Individuals with suspected COVID-19 symptoms and/or exposures will be evaluated, ideally within 72 hours to determine if there is a potential COVID-19 case. Participants may also be seen at unscheduled clinic visits if there are any safety issues that an investigator wishes to evaluate. |

| | |
|---|---|
| | Participant data from each of the study phases may be unblinded prior to study completion to support regulatory filings. When these unblindings occur, unblinded data will only be provided to Sponsor or CRO employees not involved in the subsequent conduct of the study; the specific timings of these unblindings are defined in Section 8.9. Individual participants, blinded study site staff and Sponsor team members involved in overseeing the conduct of the study will remain blinded until the end of study for all Phases (Phase 1, 2 and 3a/b/c). Sponsor staff and other persons that will become unblinded at the various data analysis time points will be specified prior to unblinding occurring.<br><br>Phase 1, 2, 3a and 3c-1 participants will be consented to have blood collected more frequently for assessment of SARS-CoV-2 neutralizing antibody responses and binding antibody responses to the SARS-CoV-2 spike protein. The study will also include collection of blood samples in all participants for potential future exploratory testing of potential correlates of protection and vaccine responses to SARS-CoV-2 variants.<br><br>An independent Data and Safety Monitoring Board (DSMB) will perform ongoing review of blinded and unblinded data, including both safety and confirmed cases of COVID-19 at scheduled data review meetings. At each meeting, the DSMB will review the available data and make recommendations to the Sponsor to continue, modify, or discontinue study enrollment (if the study is enrolling). In addition to the ongoing review of safety data, the DSMB will also review available severe COVID-19 case data to determine the risk of Vaccine-Associated Enhanced Respiratory Disease (VAERD). The DSMB will be constituted prior to commencement of Phase 3b.<br><br>An independent blinded adjudication committee will adjudicate all suspected COVID-19 cases to determine if they meet the endpoint requirements (Appendix 2). |
| **Rationale** | In January 2020, a novel variant of coronavirus was identified as the cause of an outbreak of severe pneumonia in China. This virus, SARS-CoV-2, was identified as the causative agent of a broad clinical spectrum of disease (from asymptomatic infection to severe or fatal cases of COVID-19). Over 188 million cases of COVID-19 have been confirmed worldwide and 4.1 million people have died as of July 2021, and the number of cases continues to grow in most countries. While the global incidence of COVID-19 appears to be slowing and some vaccines to prevent COVID-19 have been approved by health authorities of several countries in recent months, the number of cases remains unacceptably high in most countries, and there are significant supply limitations for these vaccines. More global access to vaccines to prevent COVID-19 is clearly needed.<br><br>Arcturus Therapeutics, Inc (Arcturus) has developed ARCT-154, a self-replicating ribonucleic acid (RNA) vaccine intended for the prevention of COVID-19 caused by SARS-CoV-2. RNA included in this study vaccine promotes the synthesis of the SARS-CoV-2 Spike (S) glycoprotein, which is the component of SARS-CoV-2 that allows for attachment of the virus to host cell angiotensin-converting enzyme 2 receptors.<br><br>The active pharmaceutical ingredient in ARCT-154, mRNA-2105, consists of a series of ribonucleotides encoding the replicase and an RNA sequence encoding the SARS-CoV-2 S-glycoprotein containing the D614G variant |

| | |
|---|---|
| | mutation. By promoting expression of this viral S protein in vaccinated individuals, ARCT-154 is intended to promote immune responses to recognize and prevent SARS-CoV-2 infection. This study will evaluate the safety, reactogenicity, immunogenicity, immunogenicity noninferiority (compared to ChAdOx1) and (exploratory) efficacy of the ARCT-154 vaccine in the prevention of COVID-19 in adults, including those adults at increased risk of severe COVID-19. |
| **Target Population** | Eligible participants will be adults without a history of COVID-19 infection, immunosuppression, or receipt of another COVID-19 vaccine. Phase 3b and 3c will be enriched for participants at higher risk for acquiring COVID-19 infection. |
| **Number of Participants** | Total of approximately 19,400:<br>• Phase 1: 100 (75 ARCT-154:25 placebo per study group)<br>• Phase 2: approximately 300 (225 ARCT-154:75 placebo per study group)<br>• Phase 3a: approximately 600 (450 ARCT-154:150 placebo per study group)<br>• Phase 3b: approximately 16,000 (8,000 per study group)<br>• Phase 3c: approximately 2,400 (1,200 per study group)<br>o The first approximately 1,500 participants will be enrolled into Phase 3c-1, the group that will have blood drawn for immunogenicity assessments. Data from 800 participants in this subgroup will be used to establish the noninferiority of immunogenicity of ARCT-154 vaccine as compared to ChAdOx1<br>o The remaining ~900 participants will form Phase 3c-2 |
| **Duration of Participation** | The expected duration of participation for an individual participant (including the Screening period) is approximately 14 months, 12 months after completion of the initial two-dose vaccination series. |
| **Number of Sites** | Approximately 20 sites in Vietnam |
| **Inclusion Criteria** | Each participant must meet all the following criteria to be enrolled in this study:<br>**Consent and Compliance**<br>1. Individuals or legally authorized representatives must freely provide consent to study participation.<br>2. Individuals must agree to comply with all study visits and procedures (including blood and nasal swab sampling, Diary completion, receipt of telephone calls from the site, willingness to be available for Unscheduled clinic visits).<br>3. Individuals in Phase 1 and Phase 2 must have access to and be capable of using an eDiary.<br>4. Individuals of childbearing potential who are sexually active must be willing to adhere to contraceptive requirements (Appendix 3).<br>**Type of Participant**<br>5. Adult males or females of at least 18 years of age at the time of signing of the informed consent.<br>6. Individuals considered at risk for COVID-19 due to work or living environment in the opinion of the Investigator. |

| | |
|---|---|
| **Exclusion Criteria** | Individuals meeting any of the following criteria will be excluded from the study:<br>**Medical Conditions**<br>1. Significant infection or other acute illness, including body temperature >100.4°F (>38.0°C) on the day prior to or Day 1. Participants meeting this criterion may be rescheduled within the relevant window periods. Afebrile participants with minor illnesses can be enrolled at the discretion of the investigator.<br>2. Pregnant or breastfeeding.<br>3. Known history of COVID-19 (nucleocapsid positive test is not exclusionary) or positive nasal swab SARS-CoV-2 by RT-PCR. Note: if obtained within 5 days prior to Screening period, do not need to repeat RT-PCR at Screening.<br>4. Close contact with a person known to be SARS-CoV-2 positive or with a clinical diagnosis of COVID-19 within 7 days prior to enrollment. Participants meeting this criterion who remain asymptomatic for 7 days may be rescheduled for enrollment within the relevant windows.<br>5. Known history of anaphylaxis, urticaria, or other significant adverse reaction to the vaccine or its excipients. See the Investigator's Brochure (IB) for list of vaccine components.<br>6. Known history of anaphylaxis to other vaccines.<br>7. Bleeding disorder considered a contraindication to intramuscular (IM) injection or phlebotomy.<br>8. Immunosuppressive or immunodeficient state, asplenia, recurrent severe infections, or known to be HIV positive.<br>9. An underlying clinically significant acute or chronic medical condition or physical examination findings for which, in the opinion of the investigator, participation would not be in the best interest of the participant (eg, compromise the well-being) or that could prevent, limit, or confound the protocol-specified assessments.<br>**Prior/Concomitant Therapy**<br>10. Has previously received investigational or approved MERS-CoV, SARS-CoV, SARS-CoV-2 vaccines or who have plans to receive off-study COVID-19 vaccines.<br>11. Has received a live replicating vaccine within 28 days prior to each study vaccination or a licensed inactivated or non-replicating vaccine within 14 days prior to first study vaccination.<br>12. Has received treatment with immunosuppressive therapy, including cytotoxic agents or systemic corticosteroids, eg, for cancer or an autoimmune disease, within 6 months prior to Screening, or planned receipt throughout the study. If systemic corticosteroids have been administered short term (<14 days) for treatment of an acute illness, participants should not be enrolled into the study until corticosteroid therapy has been discontinued for at least 28 days prior to first study vaccine administration. Inhaled/nebulized, intra-articular, intrabursal, or topical (skin or eyes) corticosteroids are permitted. |

| | |
|---|---|
| | 13. Has received systemic immunoglobulins or blood products within 3 months prior to first study vaccine administration or plans to receive such products during the study.<br>**Other Exclusions**<br>14. Demonstrated inability to comply with the study procedures.<br>15. Investigator site staff members, employees of the Sponsor or the CRO directly involved in the conduct of the study, or site staff members otherwise supervised by the investigator, or immediate family members of any of the previously mentioned individuals.<br><br>**Additional Exclusion Criteria for Phase 1 Participants Only**<br>16. Individuals ≥ 60 years of age.<br>17. Individuals with clinically significant abnormalities in medical history or physical examination, including but not limited to, the following:<br>a) Respiratory disease (e.g., chronic obstructive pulmonary disease [COPD], asthma) requiring daily medications or oxygen currently or any treatment of respiratory disease exacerbations (e.g., COPD or asthma exacerbation) warranting hospitalization or an emergency room visit or supplemental oxygen in the last 5 years.<br>b) Significant cardiovascular disease (e.g., congestive heart failure, cardiomyopathy, ischemic heart disease) or history of myocarditis or pericarditis as an adult.<br>c) Neurological or neurodevelopmental conditions (e.g., migraines, epilepsy, stroke, seizures in the last 3 years, encephalopathy, focal neurologic deficits, Guillain-Barré syndrome, encephalomyelitis or transverse myelitis).<br>d) Significant hematologic abnormalities (e.g., sickle cell disease, beta thalassemia, coagulation disorders).<br>e) Major surgery within the past 6 months.<br>f) Individuals with a history of chronic liver disease.<br>18. Individuals with the following abnormal Screening laboratory results:<br>a) Positive nasal swab SARS-CoV-2 by RT-PCR test. Note: if obtained within 5 days prior to Screening period, do not need to repeat RT-PCR at Screening.<br>b) Alanine aminotransferase (ALT), aspartate aminotransferase (AST), gamma-glutamyl transferase (GGT), total bilirubin, or alkaline phosphatase >1.5 upper limit of normal.<br>c) Hemoglobin <9.5 g/dL for females and <10.5 g/dL for males.<br>d) Platelet count $<100 \times 10^9$/L.<br>e) Other clinically significant abnormal (according to the judgment of the Investigator) Screening laboratory values.<br>f) Individuals with Type 2 diabetes and Screening hemoglobin A1c >8.0%. |

| | |
|---|---|
| | g) Hepatitis B core antigen (HbsAg), Hepatitis C virus (HCV) antibody or human immunodeficiency virus (HIV) positive.<br><br>**Additional Exclusion Criteria for Phase 3c Participants Only**<br>19. No contraindications (as specified in the prescribing information) to receiving the ChAdOx1 vaccine. |
| **Intervention** | <u>For Cohorts 1/2/3a/3b</u><br>Test: ARCT-154<br>Reference: Placebo<br><br><u>For Cohort 3c</u><br>Test: ARCT-154<br>Reference: ChAdOx1 |
| **Independent Data Committees** | • Unblinded Data and Safety Monitoring Board<br>• Blinded Adjudication Committee |
| **Stopping Rules** | All cases of severe COVID-19, SAEs determined to be related to study vaccine, or deaths determined to be related to study vaccine will be referred to the DSMB (Section 8.10.3) in an expedited fashion.<br>In the event of death determined to be related to study vaccine, further vaccination will be paused until the relevant safety data has been reviewed by DSMB.<br>The DSMB, based on their review of the blinded or unblinded safety data (including assessment for potential VAERD) will recommend whether the study should terminate, proceed unmodified, or proceed but with changes to the protocol. |
| **Analysis Sets** | The following analysis sets are defined for the statistical analyses:<br>• **Randomized Set (RS)**: Includes all participants who are randomly assigned in the study regardless of the participants' vaccination status in the study. Participants will be analyzed according to the vaccine to which the participant was randomly assigned. RS are defined for each Phase of the study as follows:<br>  o Phase 1/2/3a RS: this pools data from Phase 1, 2 and 3a participants<br>  o Phase 3b RS<br>  o Phase 3c RS<br>  o Pooled RS: this pools data from all Phases of the study<br>• **Intent-to-Treat (ITT) Analysis Set**: Includes all participants who receive any dose of study vaccine (ARCT-154 or placebo or ChAdOx1). Participants will be analyzed according to the vaccine to which the participant was randomly assigned. ITT are defined for each Phase of the study as follows:<br>  o Phase 1 ITT<br>  o Phase 2/3a ITT: this pools data from Phase 2 and 3a participants<br>  o Phase 1/2/3a ITT: this pools data from Phase 1, 2 and 3a participants and will include evaluations up to Day 92 only<br>  o Phase 3b ITT |

| | |
|---|---|
| | <ul><li>Phase 3c ITT</li><li>Pooled ITT: this pools data from all Phases of the study and will include evaluations up to Day 92 only</li></ul>• **Safety Analysis Set (SAS)**: Includes all participants who receive any dose of study vaccine (ARCT-154 or placebo or ChAdOx1). Participants will be analyzed according to the vaccine received. SAS analysis sets are defined for each Phase of the study as follows:<ul><li>Phase 1 SAS</li><li>Phase 2/3a SAS: this pools data from Phase 2 and Phase 3a participants</li><li>Phase 1/2/3a SAS: this pools data from Phase 1, Phase 2 and Phase 3a participants (up to Day 92 only)</li><li>Phase 3b SAS</li><li>Phase 3c SAS</li><li>Pooled SAS: this pools data from Phase 1/2/3a/3b for ARCT-154 and placebo, respectively. It will include evaluations up to Day 92 only</li></ul>• **Reactogenicity Analysis Set (RAS)**: Includes all participants who receive any dose of study vaccine (ARCT-154 or placebo or ChAdOx1) and provide at least 1 reactogenicity diary report. Participants will be analyzed according to the vaccine received. Reactogenicity analysis sets are defined for each Phase of the study as follows:<ul><li>Phase 1 RAS</li><li>Phase 2/3a RAS: this pools data from Phase 2 and Phase 3a participants</li><li>Phase 1/2/3a RAS: this pools data from Phase 1, Phase 2 and Phase 3a participants (up to pre-dose Day 92 only)</li><li>Phase 3b RAS</li><li>Phase 3c RAS</li><li>Pooled RAS: this pools data from Phase 1/2/3a/3b for ARCT-154 and placebo, respectively. It will include evaluations up to Day 92 only</li></ul>• **Modified Intent-to-Treat (mITT) Analysis Set**: Includes all participants who received all protocol required doses of study vaccine (ARCT-154 or placebo or ChAdOx1) up to the evaluation timepoint concerned, and who have no evidence of SARS-CoV-2 infection on Day 1 or up to 7 days after the 2nd study vaccination. The mITT analysis set will be analyzed according to vaccine assigned. mITT sets are defined for the following Phases of the study:<ul><li>Phase 3b mITT</li><li>Phase 3c mITT</li><li>Pooled mITT: this pools data from Phases 1/2/3a/3b and will include evaluations up to Day 92 only</li></ul>• **Per-Protocol (PP) Analysis Set**: Includes all eligible randomized participants who received all protocol-required doses of study vaccine (ARCT-154 or placebo or ChAdOx1) up to the evaluation timepoint concerned and within the protocol predefined window, |

| | |
|---|---|
| | and who have no major protocol deviations expected to affect efficacy, immunogenicity, safety or reactogenicity assessments as determined by the Sponsor Medical Monitor or designee in a blinded manner.<br>• The PP analysis set will be analyzed according to which study vaccine was received in the event there is a discrepancy. PP analysis sets are defined for the following Phases of the study:<br>o Phase 1 PP set: this excludes any participant that has evidence of SARS-CoV-2 infection at baseline or prior to the analysis time point concerned (IcEv3) or that receives an off-study COVID-19 vaccine prior to the analysis time point concerned (IcEv2).<br>o Phase 2/3a PP set: this pools data from Phase 2 and Phase 3a participants and excludes any participant that has evidence of SARS-CoV-2 infection at baseline or prior to the analysis time point concerned (IcEv3) or that receives an off-study COVID-19 vaccine prior to the analysis time point concerned (IcEv2).<br>o Phase 1/2/3a PP set: this pools data from Phase 1, 2 and Phase 3a participants and excludes any participant that has evidence of SARS-CoV-2 infection at baseline or prior to the analysis time point concerned (IcEv3) or that receives an off-study COVID-19 vaccine prior to the analysis time point concerned (IcEv2). This set will include evaluations of immunogenicity up to Day 92 only<br>o Phase 3b PP set: this excludes any participant with evidence of SARS-CoV-2 infection on Day 1 or up to 7 days after the 2$^{nd}$ study vaccination (IcEv3) or that receives an off-study COVID-19 vaccine prior to Day 92 (IcEv2)<br>o Phase 3c-1 PP set: this includes just the participants enrolled in the Phase 3c-1 subset of Phase 3c but excludes any participant that has evidence of SARS-CoV-2 infection at baseline or prior to the analysis time point concerned (IcEv3) or that receives an off-study COVID-19 vaccine prior to the analysis time point concerned (IcEv2)<br>o Phase 3c PP set: this includes all of Phase 3c but excludes any participant that has evidence of SARS-CoV-2 infection at baseline or prior to the analysis time point concerned (IcEv3) or that receives an off-study COVID-19 vaccine prior to the analysis time point concerned (IcEv2)<br>o Pooled PP set: this pools data from Phase 1/2/3a/3b and excludes any participant with evidence of SARS-CoV-2 infection on Day 1 or up to 7 days after the 2$^{nd}$ study vaccination (IcEv3) or that receives an off-study COVID-19 vaccine prior to Day 92 (IcEv2). This will include evaluations up to Day 92 only<br>• **Immunogenicity Analysis Set (IAS)**: Includes all participants who received all protocol required doses of study vaccine (ARCT-154 or placebo or ChAdOx1) up to the evaluation timepoint concerned, |

| | |
|---|---|
| | who have no evidence of prior SARS-CoV-2 infection at Day 1 (IcEv3; Section 8.3.1) and who have at least 1 valid post-vaccination immunogenicity assay result. Data at timepoints following evidence of a SARS-CoV-2 infection (IcEv3), use of immune-modifying drugs, blood products or immunogloblins (IcEv5) or non-study COVID-19 vaccines (IcEv2), or protocol deviations (eg, time windows for doses and blood draws for the time period summarized) that may impact immunogenicity as determined by the Sponsor Medical Monitor or designee in a blinded manner will be excluded. All participants in the IAS will be analyzed according to which study vaccine was received. IAS are defined for the following Phases of the study:<br>o Phase 1 IAS<br>o Phase 2/3a IAS: this pools data from participants in Phase 2 and 3a<br>o Phase 1/2/3a IAS: this pools data from Phase 1, 2 and 3a participants and will include evaluations of immunogenicity up to Day 92 only<br>o Phase 3b IAS<br>o Phase 3c-1 IAS |

**Objectives and Endpoints**

In interpreting the objectives and endpoints for this study, the following considerations will apply:

- The overall primary efficacy endpoint for the study is vaccine efficacy as evaluated in the Phase 3b Modified Intent to Treat (mITT) population
- The overall primary safety endpoints are those evaluated in the Phase 3b Safety Analysis Set (SAS) and Phase 3b Reactogenicity Analysis Set (RAS)
- Phase 1/2/3a and subgroups thereof constitute a substudy with separate objectives and endpoints. As these constitute a substudy, and there is no evaluation of efficacy for this substudy, no adjustment of type 1 error is applied to the overall efficacy primary endpoint
- The Pooled efficacy endpoint is only evaluated as exploratory, so no adjustment of type 1 error is applied to the overall efficacy primary endpoint
- Phase 3c and subgroups thereof also constitute a substudy with separate objectives and endpoints. As these constitute a substudy, and efficacy for this study is only evaluated as exploratory, no adjustment of type 1 error is applied to the overall efficacy primary endpoint.

**Phases 1, 2 and 3a Substudy Objectives and Endpoints**

Immunogenicity data from participants in Phases 1, 2 and 3a will be pooled for analysis up to Day 92. With implementation of protocol version 8.0, participants in Phase 2 and 3a that received ARCT-154 in the initial vaccination series are further randomized to receive a 3rd vaccination of either ARCT-154 or placebo at Day 92 followed by placebo at Day 120 in order to compare immunogenicity after 3 injections of ARCT-154 with that after 2 injections. Therefore, after Day 92 only data from Phase 2/3a will be pooled and will be displayed separately from participants in Phase 1.

Safety data from participants in Phase 2/3a will be pooled for all time points and will be displayed separately to safety data from Phase 1.

| **Phase 1/2/3a Primary Objectives** | **Endpoint Description** |
|---|---|
| 1. To assess the safety and reactogenicity of ARCT-154 compared to placebo | Safety will be evaluated in all participants in Phase 1 and Phase 2/3a Safety Analysis Sets (SAS, Section 8.2) and will be summarized for each vaccination as number and percentage of participants with: |

| | |
|---|---|
| | • Any unsolicited AE starting within 28 days after each study vaccine administration, summarized by severity and relationship to study vaccine.<br>• Any medically attended adverse event (MAAE), serious adverse event (SAE), or AE leading to discontinuation/withdrawal through Final Visit/Early Termination (ET) summarized by relationship to study vaccine.<br>Reactogenicity will be evaluated in participants in the Phase 1 and Phase 2/3a Reactogenicity Analysis Sets (RAS) and will be summarized following each vaccination on Day 1 and Day 29 only in Phase 1 and Day 1, Day 29, and Day 92 in Phase 2/3a as the number and percentage of participants with:<br>• Any solicited local or systemic AE starting within 7 days after each study vaccine administration by toxicity grade.<br>Additional safety analyses in the Phase 1/2/3a SAS may also be performed as exploratory. |
| 2. To assess the neutralizing antibody (NAb) responses to ARCT-154 by surrogate virus neutralization test at Day 57 | Neutralizing antibody (NAb) responses by surrogate virus neutralization test (sVNT) will be evaluated at Day 1 (baseline) and Day 57 for assessment of seroconversion in all participants in the Phase 1/2/3a Immunogenicity Analysis Set (IAS, Section 8.2).<br>• The endpoint is defined as the proportion of participants in each study vaccine group that demonstrate seroconversion (defined as: 4-fold increase in antibody concentration from baseline).<br>Data will be summarized according to use of international reference standards, if available. |
| **Phase 1/2/3a Secondary Objectives** | **Endpoint Description** |
| 1. To assess the neutralizing and IgG binding antibody responses to ARCT-154 over time | Immunogenicity will be evaluated at the following time points:<br>• Phase 1 IAS: Day 1 (baseline), Day 29, Day 57, Day 92, Day 394.<br>• Phase 2/3a IAS: Day 1 (baseline), Day 29, Day 57, Day 92, Day 120, Day 394<br>• Phase 1/2/3a IAS: Day 1 (baseline), Day 29, Day 57, Day 92<br>The following will be evaluated:<br>• Neutralizing antibody (NAb) by surrogate virus neutralization test (sVNT) and IgG antibody binding the full-length SARS-CoV-2 spike protein (binding antibody [BAb]) responses will be evaluated; assessments for both BAb and NAb will include:<br>○ Antibody level: GMC (BAb and NAb)<br>○ Increase in antibody levels from baseline (GMFR or other measure, as appropriate for the assay concerned)<br>○ Proportion of participants in each study vaccine group with seroconversion at each timepoint. Note: NAb responses at Day 57 evaluated as primary.<br>Data will be summarized according to use of international reference standards, if available.<br>Full details will be provided in the statistical analysis plan. |
| 2. To assess early neutralizing antibody responses using a live virus assay | NAb responses by plaque reduction neutralization test at 50% reduction (PRNT50) will be evaluated in the subset of the Phase 1/2 IAS (Section 8.2) |

| | |
|---|---|
| | comprising all participants enrolled in Phase 1 and the first 50 evaluable participants in Phase 2 at Day 1 (baseline), Day 29 and Day 57.<br>The following endpoints will be assessed:<br>• Geometric mean antibody concentration (GMC)<br>• Increase in antibody levels from baseline (GMFR)<br>• Proportion of participants in each study vaccine group with seroconversion. |
| **Phase 1/2/3a Exploratory Objectives** | **Endpoint Description** |
| 1. To compare the humoral immune responses of ARCT-154 to those of patients recovering from COVID-19 | Day 57 GMT/GMC results following vaccination with ARCT-154 may be compared with the test results for convalescent sera from COVID-19 patients measured on the same assay for the subset of the Phase 1/2/3a IAS comprising all participants enrolled in Phase 1 and the first 50 evaluable participants in Phase 2. The following assays will be used:<br>• PRNT50<br>• MNT<br>Immune responses may also be assessed at additional time points and/or in additional participants in Phase 2 and Phase 3a. If performed, this will be described in the statistical analysis plan (SAP).<br>For the MNT assay, data will be summarized according to use of international reference standards, if available |
| 2. To evaluate the efficacy of ARCT-154 versus placebo for the prevention of virologically confirmed COVID-19 | Efficacy may be evaluated in participants in the Phase 1/2/3a Modified Intent to Treat (mITT) set (Section 8.2). The endpoint is as follows:<br>• The first occurrence of confirmed, protocol-defined (Appendix 2) COVID-19 with onset between Day 36 and Day 92 inclusive.<br>Efficacy may also be evaluated in participants that have received any dose of study vaccine in the first vaccination series, with no evidence of infection prior to vaccination. The endpoint is as follows:<br>• The first occurrence of confirmed, protocol-defined (Appendix 2) COVID-19 with onset at any time after the first study vaccination and up to Day 92. |
| 3. To assess the neutralizing antibody immune responses to ARCT-154 to SARS-CoV-2 variants of concern/variants of interest following 2 and 3 vaccinations with ARCT-154 | Blood samples from post-vaccination timepoints may be evaluated for immune responses to SARS-CoV-2 variants of concern/variants of interest for the following groups/time points:<br>• The subset of the Phase 1/2/3a IAS comprising all participants enrolled in Phase 1 and the first 50 participants in Phase 2 at Day 1 (baseline) and Day 57.<br>• The subset of the Phase 2/3a IAS comprising the first 150 participants that received ARCT-154 in the initial vaccination series in Phase 2; evaluated at Day 1 (baseline), Day 57 and Day 120.<br>The following assays may be used:<br>• PRNT50<br>• sVNT<br>• MNT |
| 4. To assess neutralizing antibody responses using a pseudovirus | NAb responses by microneutralization test (MNT) will be evaluated for the following groups/time points: |

| | |
|---|---|
| microneutralization test (MNT) following 2 (Day 57) and 3 (Day 120) vaccinations with ARCT-154 | • The subset of the Phase 1/2/3a IAS comprising all participants enrolled in Phase 1 and the first 50 evaluable participants in Phase 2 at Day 1 (baseline) and Day 57.<br>• The subset of the Phase 2/3a IAS comprising the first 150 participants that received ARCT-154 in the initial vaccination series in Phase 2; evaluated at Day 1 (baseline), Day 57 and Day 120.<br>The following endpoints will be assessed:<br>o Geometric mean antibody concentration (GMC)<br>o Increase in antibody levels from baseline (GMFR)<br>o Proportion of participants in each study vaccine group with seroconversion<br>Data will be summarized according to use of international reference standards, if available. |
| 5. To assess the neutralizing antibody (NAb) responses by surrogate virus neutralization test to ARCT-154 following 2 (Day 57) and 3 (Day 120) vaccinations with ARCT-154 in Phase 2/3a Participants | Neutralizing antibody (NAb) responses by surrogate virus neutralization test (sVNT) will be evaluated at Day 1 (baseline), Day 57 and Day 120 for assessment of seroconversion in all participants in the Phase 2/3a IAS that received ARCT-154 in the initial vaccination series<br>• The endpoint is defined as the proportion of participants that demonstrate seroconversion (defined as: 4-fold increase in antibody concentration from baseline).<br>Data will be summarized according to use of international reference standards, if available. |
| 6. To assess the IgG binding antibody responses to ARCT-154 following 2 (Day 57) and 3 (Day 120) vaccinations with ARCT-154 in Phase 2/3a participants | IgG antibody binding the full-length SARS-CoV-2 spike protein (binding antibody [BAb]) responses will be evaluated at Day 1 (baseline), Day 57 and Day 120 in all participants in the Phase 2/3a IAS that received ARCT-154 in the initial vaccination series. The following endpoints will be assessed:<br>• Geometric mean antibody concentration (GMC)<br>• Increase in antibody levels from baseline (GMFR)<br>• Proportion of participants in each study vaccine group with seroconversion<br>Data will be summarized according to use of international reference standards, if available. |

| **Phase 3b Objectives and Endpoints** | |
|---|---|
| **Phase 3b Primary Objectives** | **Endpoint Description** |
| 1. To assess the safety and reactogenicity of ARCT-154 compared to placebo | Safety will be evaluated in all participants in the Phase 3b SAS and will be summarized for each vaccination as number and percentage of participants with:<br>• Any unsolicited AE starting within 28 days after each study vaccine administration, summarized by severity and relationship to study vaccine.<br>• Any medically attended adverse event (MAAE), serious adverse event (SAE), or AE leading to discontinuation/withdrawal through Final Visit/Early Termination (ET) summarized by relationship to study vaccine.<br>Reactogenicity will be evaluated in participants in the Phase 3b RAS and will be summarized following vaccinations on Day 1 and Day 29 only as the number and percentage of participants with: |

| | |
|---|---|
| | • Any solicited local or systemic AE starting within 7 days after each study vaccine administration by toxicity grade. |
| 2. To evaluate the efficacy of ARCT-154 and placebo for the prevention of virologically confirmed COVID-19 | Efficacy will be evaluated in all participants in the Phase 3b mITT. The endpoint to be evaluated is as follows:<br>• The first occurrence of confirmed, protocol-defined (Appendix 2) COVID-19 with onset between Day 36 and Day 92 inclusive. |
| **Phase 3b Secondary Objectives** | **Endpoint Description** |
| 1. To evaluate the efficacy of ARCT-154 and placebo for the prevention of virologically confirmed severe COVID-19 | Efficacy will be evaluated in all participants in the Phase 3b mITT. The endpoint to be evaluated is as follows:<br>• The first occurrence of confirmed, protocol-defined (Appendix 2) severe COVID-19 with onset between Day 36 and Day 92 inclusive. |
| 2. To evaluate the efficacy of ARCT-154 and placebo for the prevention of virologically confirmed COVID-19 at any time after first vaccination | Efficacy will be evaluated in all participants in the Phase 3b mITT that have received any dose of study vaccine in the first vaccination series, with no evidence of infection prior to vaccination. The endpoint is as follows:<br>• The first occurrence of confirmed, protocol-defined (Appendix 2) COVID-19 with onset at any time after the first study vaccination and up to Day 92 inclusive. |
| 3. To evaluate the efficacy of ARCT-154 and placebo for the prevention of death due to COVID-19 | Efficacy will be evaluated in all participants in the Phase 3b mITT. The endpoint is as follows:<br>• The occurrence of death attributed to COVID-19 disease occurring between Day 36 and Day 92 inclusive. |
| 4. To evaluate the efficacy of ARCT-154 and placebo for the prevention of virologically confirmed COVID-19 regardless of baseline status for evidence of prior SARS-CoV-2 infection | Efficacy will be evaluated in all participants in the Phase 3b mITT regardless of baseline status for evidence of prior SARS-CoV-2 infection. The endpoint to be evaluated is as follows:<br>• The first occurrence of confirmed, protocol-defined (Appendix 2) COVID-19 with onset between Day 36 and Day 92 inclusive. |
| **Phase 3b Exploratory Objectives** | **Endpoint Description** |
| 1. To evaluate the efficacy of ARCT-154 and placebo for the prevention of virologically confirmed severe COVID-19 at any time after first vaccination | Efficacy may be evaluated in all participants in the Phase 3b mITT who have received any dose of study vaccine in the first vaccination series, with no evidence of infection prior to vaccination. The endpoint is as follows:<br>• The first occurrence of confirmed, protocol-defined (Appendix 2) severe COVID-19 with onset at any time after the first study vaccination and up to Day 92. |
| 2. To evaluate the efficacy of ARCT-154 and placebo for the prevention of death due to COVID-19 at any time after first vaccination | Efficacy may be evaluated in all participants in the Phase 3b mITT that have received any dose of study vaccine in the first vaccination series. The endpoint is as follows:<br>• The occurrence of death attributed to COVID-19 occurring at any time after the first study vaccination and up to Day 92. |

| **Phase 3b Exploratory Objectives** | **Endpoint Description** |
|---|---|
| 3. To evaluate the efficacy of ARCT-154 and placebo for the prevention of virologically confirmed COVID-19 at any time after first vaccination regardless of baseline status for evidence of prior SARS-CoV-2 infection | Efficacy may be evaluated in all participants in the Phase 3b mITT that have received any dose of study vaccine in the first vaccination series, regardless of baseline status for evidence of prior SARS-CoV-2 infection. The endpoints are as follows:<br>• The first occurrence of confirmed, protocol-defined (Appendix 2) COVID-19 with onset at any time after the first study vaccination and up to Day 92. |
| 4. To evaluate the efficacy of ARCT-154 and placebo for the prevention of virologically confirmed SARS-CoV-2 by specific strain | Efficacy will be evaluated in participants in Phase 3b mITT. The endpoint to be evaluated is as follows:<br>• The first occurrence of confirmed, protocol-defined (Appendix 2) COVID-19 for all participants infected with the same strain of SARS-CoV-2, with onset between Day 36 and Day 92 inclusive.<br>A second nasal swab specimen collected contemporaneously in participants under evaluation for COVID-19 will be retained for possible future testing to identify the SARS-CoV-2 strain detected in the COVID-19 case. |
| 5. To evaluate the efficacy of ARCT-154 and placebo for the prevention of virologically confirmed severe SARS-CoV-2 by specific strain | Efficacy will be evaluated in participants in Phase 3b mITT. The endpoint to be evaluated is as follows:<br>• The first occurrence of confirmed, protocol-defined (Appendix 2) severe COVID-19 for all participants infected with the same strain of SARS-CoV-2, with onset between Day 36 and Day 92 inclusive.<br>A second nasal swab specimen collected contemporaneously in participants under evaluation for COVID-19 will be retained for possible future testing to identify the SARS-CoV-2 strain detected in the COVID-19 case. |
| 6. To evaluate an immune correlate associated with reduced risk of COVID-19 | • Blood samples drawn at Baseline and Day 57 in all participants will be held for potential future testing for evaluation of a correlate of protection. |

| **Phase 3c Substudy Objectives and Endpoints** | |
|---|---|
| The first approximately 1,500 participants in Phase 3c will be enrolled into Phase 3c-1, the group that will have blood drawn for immunogenicity assessments. Data from 800 participants in this subgroup will be used to establish the noninferiority of immunogenicity of ARCT-154 vaccine as compared to ChAdOx1. Samples from the remaining 700 participants in Phase 3c-1 will be retained for potential future immunological testing, for example, against newly emerging variants of concern. Safety evaluations will be based on all participants in Phase 3c. | |
| **Phase 3c Primary Objectives** | **Endpoint Description** |
| 1. To assess the safety and reactogenicity of ARCT-154 and ChAdOx1 | Safety will be evaluated in all participants in the Phase 3c SAS and will be summarized for each vaccination as number and percentage of participants with:<br>• Any unsolicited AE starting within 28 days after each study vaccine administration, summarized by severity and relationship to study vaccine.<br>• Any medically attended adverse event (MAAE), serious adverse event (SAE), or AE leading to discontinuation/withdrawal through Final Visit/Early Termination (ET) summarized by relationship to study vaccine.<br>Reactogenicity will be evaluated in participants in the Phase 3c RAS and will be summarized following vaccinations on Day 1 and Day 29 only as the number and percentage of participants with:<br>• Any solicited local or systemic AE starting within 7 days after each study vaccine administration by toxicity grade. |
| 2. To evaluate noninferiority of neutralizing antibody geometric mean concentration for ARCT-154 versus ChAdOx1 at Day 57 | Neutralizing antibody (NAb) responses by pseudovirus microneutralization test (MNT) will be evaluated at Day 57 for assessment of geometric mean concentration in all participants in the Phase 3c-1 IAS.<br>• The endpoint is the geometric mean ratio of the surrogate virus neutralization test for ARCT-154 and ChAdOx1 at Day 57<br>Data will be summarized according to use of international reference standards, if available.<br>Noninferiority will be concluded if the lower bound of the 95% confidence interval for the geometric mean ratio of ARCT-154 / ChAdOx1 >0.67. |
| **Phase 3c Secondary Objectives** | **Endpoint Description** |
| 1. To evaluate noninferiority of IgG binding antibody geometric mean concentration for ARCT-154 versus ChAdOx1 at Day 57 | IgG antibody binding the full-length SARS-CoV-2 spike protein (binding antibody [BAb]) responses will be evaluated Day 57 for assessment of geometric mean concentration in all participants in the Phase 3c-1 IAS.<br>• The endpoint is the geometric mean ratio of BAb for ARCT- 154 and ChAdOx1 at Day 57<br>Data will be summarized according to use of international reference standards, if available<br>Noninferiority will be concluded if the lower bound of the 95% confidence interval for the geometric mean ratio of ARCT-154 / ChAdOx1 >0.67. |
| 2. To evaluate noninferiority of neutralizing antibody seroconversion rate for | Neutralizing antibody (NAb) responses by pseudovirus microneutralization test (MNT) will be evaluated at Day 1 (baseline) and Day 57 for assessment of seroconversion in all participants in the Phase 3c-1 IAS. |

| | |
|---|---|
| ARCT-154 versus ChAdOx1 at Day 57 | • The endpoint is seroconversion rate (defined as: 4-fold increase in titer from baseline) for ARCT-154 and ChAdOx1 at Day 57<br>Data will be summarized according to use of international reference standards, if available<br>Noninferiority will be concluded if the lower bound of the 95% confidence interval of the difference between neutralizing antibody seroconversion rates (ARCT-154 group minus ChAdOx1 nCoV-1 group) excludes the non-inferiority margin of -10%. |
| 3. To evaluate noninferiority of IgG binding antibody seroconversion rate for ARCT-154 versus ChAdOx1 at Day 57 | IgG antibody binding the full-length SARS-CoV-2 spike protein (BAb) responses will be evaluated at Day 1 (baseline) and Day 57 for assessment of seroconversion in all participants in the Phase 3c-1 IAS.<br>• The endpoint is seroconversion rate (defined as: 4-fold increase in titer from baseline) for ARCT-154 and ChAdOx1 at Day 57<br>Data will be summarized according to use of international reference standards, if available<br>Noninferiority will be concluded if the lower bound of the 95% confidence interval of the difference between binding antibody seroconversion rates (ARCT-154 group minus ChAdOx1 nCoV-1 group) excludes the non-inferiority margin of -10%. |
| 4. To evaluate superiority of neutralizing antibody geometric mean concentration for ARCT-154 versus ChAdOx1 at Day 57 | Neutralizing antibody (NAb) responses by pseudovirus microneutralization test (MNT) will be evaluated at Day 57 for assessment of geometric mean concentration in all participants in the Phase 3c-1 IAS.<br>• The endpoint is the geometric mean ratio of the MNT for ARCT-154 and ChAdOx1 at Day 57<br>Data will be summarized according to use of international reference standards, if available.<br>Superiority will be concluded if the lower bound of the 95% confidence interval for the geometric mean ratio of ARCT-154 / ChAdOx1 >1.0. |
| 5. To evaluate superiority of IgG binding antibody geometric mean concentration for ARCT-154 versus ChAdOx1 at Day 57 | IgG antibody binding the full-length SARS-CoV-2 spike protein (BAb) responses will be evaluated at Day 57 for assessment of geometric mean concentration in all participants in the Phase 3c-1 IAS.<br>• The endpoint is the geometric mean ratio of BAb for ARCT- 154 and ChAdOx1 at Day 57<br>Data will be summarized according to use of international reference standards, if available.<br>Superiority will be concluded if the lower bound of the 95% confidence interval for the geometric mean concentration ratio of ARCT-154 / ChAdOx1 >1.0. |
| 6. To assess the neutralizing and IgG binding antibody immune responses for ARCT-154 and ChAdOx1 over time | Neutralizing antibody (NAb) by pseudovirus microneutralization test (MNT) and IgG antibody binding the full-length SARS-CoV-2 spike protein (BAb) responses will be evaluated at: Day 1 (baseline), Day 29, Day 57 and Day 211 for all participants in the Phase 3c-1 IAS. Assessments for both BAb and NAb will include:<br>• Antibody level: GMC<br>• Increase in antibody levels from baseline (GMFR or other measure, as appropriate for the assay concerned)<br>• Geometric mean ratio (GMR)<br>• Proportion of participants in each study vaccine group with seroconversion at each timepoint |

| | |
|---|---|
| | Samples will also be collected at Day 394 and may be evaluated in the same assays.<br><br>Note: NAb and BAb responses that have been evaluated as other primary and secondary endpoints will be included in this endpoint for completeness of data presentation across time points. Full details will be provided in the statistical analysis plan. |
| **Phase 3c Exploratory Objectives** | **Endpoint Description** |
| 1. To evaluate the efficacy of ARCT-154 and ChAdOx1 nCoV-1 for the prevention of virologically confirmed COVID-19 | Efficacy will be evaluated in all participants in the Phase 3c mITT. The endpoint to be evaluated is as follows:<br>• The first occurrence of confirmed, protocol-defined (Appendix 2) COVID-19 with onset after Day 35.<br>Efficacy may also be evaluated in participants that have received any dose of study vaccine in the first vaccination series, with no evidence of infection prior to vaccination. The endpoint is as follows:<br>• The first occurrence of confirmed, protocol-defined (Appendix 2) COVID-19 with onset at any time after the first study vaccination. |
| 2. To evaluate the efficacy of ARCT-154 and ChAdOx1 nCoV-1 for the prevention of virologically confirmed severe COVID-19 | Efficacy will be evaluated in all participants in the Phase 3c mITT. The endpoint to be evaluated is as follows:<br>• The first occurrence of confirmed, protocol-defined (Appendix 2) severe COVID-19 with onset after Day 35.<br>Efficacy may also be evaluated in participants that have received any dose of study vaccine in the first vaccination series, with no evidence of infection prior to vaccination. The endpoint is as follows:<br>• The first occurrence of confirmed, protocol-defined (Appendix 2) severe COVID-19 with onset at any time after the first study vaccination. |
| 3. To evaluate the efficacy of ARCT-154 and ChAdOx1 nCoV-1 for the prevention of death due to COVID-19 | Efficacy will be evaluated in all participants in the Phase 3c mITT. The endpoint to be evaluated is as follows:<br>• The occurrence of death attributed to COVID-19 disease occurring after Day 35.<br>Efficacy may also be evaluated in participants that have received any dose of study vaccine in the first vaccination series, with no evidence of infection prior to vaccination. The endpoint to be evaluated is as follows:<br>• The occurrence of death attributed to COVID-19 disease occurring at any time after the first study vaccination. |
| 4. To evaluate the efficacy of ARCT-154 and ChAdOx1 for the prevention of virologically confirmed COVID-19 regardless of baseline status for evidence of prior SARS-CoV-2 infection | Efficacy will be evaluated in all participants in the Phase 3c mITT regardless of baseline status for evidence of prior SARS-CoV-2 infection. The endpoint to be evaluated is as follows:<br>• The first occurrence of confirmed, protocol-defined (Appendix 2) COVID-19 with onset after Day 35.<br>Efficacy may also be evaluated in participants that have received any dose of study vaccine in the first vaccination series. The endpoint is as follows:<br>• The first occurrence of confirmed, protocol-defined (Appendix 2) COVID-19 with onset at any time after the first study vaccination. |

| | |
|---|---|
| 5. To evaluate the neutralizing antibody responses of ARCT-154 and ChAdOx1 using a surrogate virus neutralization test | Neutralizing antibody (NAb) responses by surrogate virus neutralization test (sVNT) may be evaluated at individual time points. The following endpoints may be assessed:<br>• Geometric mean antibody concentration (GMC)<br>• Increase in antibody levels from baseline (GMFR)<br>• Proportion of participants in each study vaccine group with seroconversion.<br>Data will be summarized according to use of international reference standards, if available. |
| 6. To evaluate the neutralizing antibody responses of ARCT-154 and ChAdOx1 to SARS-CoV-2 variants of concern/variants of interest | Neutralizing antibody responses to SARS-CoV-2 variants of concern may be evaluated at individual time points in participants in the Phase 3c-1 IAS using a pseudovirus microneutralization test (MNT) and/or surrogate virus neutralization test (sVNT) for assessment of:<br>• Antibody level: GMC<br>• Increase in antibody levels from baseline (GMFR or other measure, as appropriate for the assay concerned)<br>• Geometric mean ratio (GMR)<br>• Proportion of participants in each study vaccine group with seroconversion at each timepoint.<br>The following assays may be used:<br>• PRNT50<br>• sVNT<br>• MNT |

| **Pooled Objectives and Endpoints** | |
|---|---|
| **Pooled Primary Objectives** | **Endpoint Description** |
| 1. To assess the safety and reactogenicity of ARCT-154 and comparator vaccines (placebo, ChAdOx1 nCoV-1) | Safety data will be evaluated in the Pooled SAS. Data and will be summarized for each vaccination as number and percentage of participants with:<br>• Any unsolicited AE starting within 28 days after each study vaccine administration, summarized by severity and relationship to study vaccine.<br>• Any medically attended adverse event (MAAE), serious adverse event (SAE), or AE leading to discontinuation/withdrawal through Final Visit/Early Termination (ET) summarized by relationship to study vaccine.<br>Reactogenicity will be evaluated in the Pooled RAS and will be summarized following vaccinations on Day 1 and Day 29 only as the number and percentage of participants with:<br>• Any solicited local or systemic AE starting within 7 days after each study vaccine administration by toxicity grade. |
| 2. To evaluate the efficacy of ARCT-154 and placebo for the prevention of virologically confirmed COVID-19 | Efficacy will be evaluated in all participants in Pooled mITT. The endpoint to be evaluated is as follows:<br>• The first occurrence of confirmed, protocol-defined (Appendix 2) COVID-19 with onset between Day 36 and Day 92 inclusive. |

| Pooled Secondary Objectives | Endpoint Description |
|---|---|
| 1. To evaluate the efficacy of ARCT-154 and placebo for the prevention of virologically confirmed severe COVID-19 | Efficacy will be evaluated in all participants in the Pooled mITT. The endpoint to be evaluated is as follows:<br>• The first occurrence of confirmed, protocol-defined (Appendix 2) severe COVID-19 with onset between Day 36 and Day 92 inclusive.<br>• Severity as scored using the FDA criteria will be the principal analysis. Severity using the WHO criteria will be performed as a sensitivity analysis |
| 2. To evaluate the efficacy of ARCT-154 and placebo for the prevention of virologically confirmed COVID-19 at any time after first vaccination | Efficacy will be evaluated in participants that have received any dose of study vaccine in the first vaccination series, with no evidence of infection prior to vaccination. The endpoint is as follows:<br>The first occurrence of confirmed, protocol-defined (Appendix 2) COVID-19 with onset at any time after the first study vaccination and up to Day 92 inclusive. |
| 3. To evaluate the efficacy of ARCT-154 and placebo for the prevention of death due to COVID-19 | Efficacy will be evaluated in all participants in the Pooled mITT The endpoint is as follows:<br>• The occurrence of death attributed to COVID-19 with onset between Day 36 and Day 92 inclusive. |
| 4. To evaluate the efficacy of ARCT-154 and placebo for the prevention of virologically confirmed COVID-19 regardless of baseline status for evidence of prior SARS-CoV-2 infection | Efficacy will be evaluated in all participants in the Pooled mITT regardless of baseline status for evidence of prior SARS-CoV-2 infection. The endpoint to be evaluated is as follows:<br>• The first occurrence of confirmed, protocol-defined (Appendix 2) COVID-19 with onset between Day 36 and Day 92 inclusive. |
| **Pooled Exploratory Objectives** | **Endpoint Description** |
| 1. To evaluate the efficacy of ARCT-154 and placebo for the prevention of virologically confirmed severe COVID-19 at any time after first vaccination | Efficacy may be evaluated in all participants in the Pooled mITT that have received any dose of study vaccine in the first vaccination series, with no evidence of infection prior to vaccination. The endpoint is as follows:<br>• The first occurrence of confirmed, protocol-defined (Appendix 2) severe COVID-19 with onset at any time after the first study vaccination and up to Day 92. |
| 2. To evaluate the efficacy of ARCT-154 and placebo for the prevention of death due to COVID-19 at any time after first vaccination | Efficacy may be evaluated in all participants in the Pooled mITT that have received any dose of study vaccine in the first vaccination series. The endpoint is as follows:<br>• The occurrence of death attributed to COVID-19 disease occurring at any time after the first study vaccination and up to Day 92. |

| | |
|---|---|
| 3. To evaluate the efficacy of ARCT-154 and placebo for the prevention of virologically confirmed COVID-19 at any time after first vaccination regardless of baseline status for evidence of prior SARS-CoV-2 infection | Efficacy may be evaluated in all participants in the Pooled mITT that have received any dose of study vaccine in the first vaccination series. The endpoints are as follows:<br>• The first occurrence of confirmed, protocol-defined (Appendix 2) COVID-19 with onset at any time after the first study vaccination and up to Day 92. |
| 4. To evaluate the efficacy of ARCT-154 and placebo for the prevention of virologically confirmed SARS-CoV-2 by specific strain | Efficacy will be evaluated in participants in Pooled mITT. The endpoint to be evaluated is as follows:<br>• The first occurrence of confirmed, protocol-defined (Appendix 2) COVID-19 for all participants infected with the same strain of SARS-CoV-2, with onset between Day 36 and Day 92 inclusive.<br>A second nasal swab specimen collected contemporaneously in participants under evaluation for COVID-19 will be retained for possible future testing to identify the SARS-CoV-2 strain detected in the COVID-19 case. |
| 5. To evaluate the efficacy of ARCT-154 and placebo for the prevention of virologically confirmed severe SARS-CoV-2 by specific strain | Efficacy will be evaluated in participants in Pooled mITT. The endpoint to be evaluated is as follows:<br>• The first occurrence of confirmed, protocol-defined (Appendix 2) severe COVID-19 for all participants infected with the same strain of SARS-CoV-2, with onset between Day 36 and Day 92 inclusive.<br>A second nasal swab specimen collected contemporaneously in participants under evaluation for COVID-19 will be retained for possible future testing to identify the SARS-CoV-2 strain detected in the COVID-19 case. |
| 6. To evaluate for an immune correlate associated with reduced risk of COVID-19 | Blood samples drawn at Baseline and Day 57 in all participants will be held for potential future testing for evaluation of a correlate of protection. |

| | |
|---|---|
| **Statistical Considerations** | The design of the study is structured to maintain the integrity of the safety, efficacy and immunogenicity noninferiority endpoints for Phase 3b and 3c such that these data remain blinded to the blinded Sponsor employees and CRO until the analysis of time points for these endpoints whilst earlier unblinding of Phases 1/2/3a participants for analysis of Phase 1/2/3a immunogenicity and safety may occur earlier to inform filing for EUA.<br>**Phase 3b Primary Analyses**<br>The primary Phase 3b safety endpoints will be evaluated in the Phase 3b Safety Analysis Set and Phase 3b Reactogenicity Analysis Set (Section 8.2). With a sample size ~16,000 participants randomized and with approximately 8,000 participants randomized in the study to receive ARCT-154 for the primary safety analysis, if the incidence rate of an adverse event is 0.1%, the probability to detect one event in 8,000 vaccinated participants is >99.9%, based upon the following formula:<br>$p = 1 - (1 - R)^N$ |

| | |
|---|---|
| | where R = incidence rate and N = sample size<br><br>The primary statistical analyses for vaccine efficacy (VE) will be performed using virologically confirmed COVID-19 cases as determined in a blinded fashion by an Independent Adjudication Committee according to definitions provided in Appendix 2. This analysis of VE will be based on the Phase 3b mITT analysis set (Section 8.2). Sensitivity analyses will be performed in the Phase 3b PP set and the Pooled mITT set.<br>The null hypothesis ($H0^{Efficacy}$) is as follows: $H0^{Efficacy}$: VE ≤ 30%<br>VE is estimated as 1-HR, where HR is the hazard ratio. The primary efficacy objective will be met if the lower limit of the 95% confidence interval (CI) for VE exceeds 30%.<br>Under the assumptions of proportional hazards over time and a 50% reduction in hazard rate (50% VE) and with 1:1 randomization of ARCT-154 and placebo, a total of 372 confirmed COVID-19 cases will provide approximately 90% power to reject the null hypothesis (H0: VE ≤ 30%), using a log-rank test statistic with a 1-sided false positive error rate of 0.025.<br><br>**Phase 1/2/3a Substudy**<br><br>Data from Phases 1, 2 and 3a will be pooled for the analyses of immunogenicity and Safety up to Day 92. The total sample size for Phases 1, 2 and 3a is primarily driven by the size of safety database required at the time of potential EUA application. Safety analyses will be performed in the Phase 1/2/3a SAS and Phase 1/2/3a RAS (Section 8.2). With approximately 750 participants randomized in Phases 1/2/3a to receive ARCT-154 for the Phase 1/2/3a primary safety analysis, if the incidence rate of an adverse event is 1.0%, the probability to detect one event in 750 vaccinated participants is >99%, based upon the following formula:<br>$p = 1 - (1 - R)^{N}$<br>where R = incidence rate and N = sample size<br><br>The primary immunogenicity endpoint of Phase 1/2/3a will be evaluated in participants in the Phase 1/2/3a Immunogenicity Analysis Set (Section 8.2). It is defined as the proportion of participants in each study vaccine group that demonstrate seroconversion (defined as: 4-fold increase in titer from baseline) by surrogate virus neutralization assay at Day 57. Sensitivity analyses will be performed in the Phase 1/2/3a per protocol (PP) set.<br><br>The null hypothesis ($H0^{Immunogenicity}$) is that the lower bound of the 95% CI for ARCT-154 seroconversion rate ($SC^{154}$) is ≤ seroconversion rate for placebo ($SC^{Placebo}$); i.e., $H0^{Immunogenicity}$: lower bound 95% CI for $SC^{154} \leq SC^{Placebo}$. A sample size of 750 and 250 participants in the ARCT-154 and placebo groups respectively will provide greater than 90% power to exclude the null hypothesis with a 1-sided type 1 error rate of 0.025 assuming that 10% of participants are excluded from the analysis (for example, due to baseline seropositivity), and that $SC^{154}$ >50% and $SC^{Placebo}$ ≤10%.<br><br>**Phase 3c Substudy**<br>The primary safety endpoints of Phase 3c will be conducted in the Phase 3c Safety Analysis Set and Phase 3c Reactogenicity Analysis Set (Section 8.2). With a sample size of approximately 2,400 participants randomized and with approximately 1,200 participants randomized in the study to receive ARCT-154 or ChAdOx1 for the safety analysis, based upon the following |

| | |
|---|---|
| | formula, if the incidence rate of an adverse event is 0.1%, the probability to detect one event in 1,200 vaccinated participants is 69.9% and, if the incidence rate is 1.0%, the probability is more than 99%:<br>$$p = 1 - (1 - R)^N$$<br>where R = incidence rate and N = sample size<br><br>The primary immunogenicity noninferiority endpoint for Phase 3c is the ratio of geometric mean concentrations (geometric mean ratio or GMR) for neutralizing antibody (using the pseudovirus microneutralization assay [MNT]) concentration and will be evaluated in participants in the Phase 3c-1 Immunogenicity Analysis Set (Section 8.2). Sensitivity analyses will be performed in the Phase 3c PP set. International units (i.e., the WHO/NIBSC international reference standard) will be used if available.<br><br>The null hypothesis ($H0^{GMR}$) for the primary endpoint is as follows:<br>$H0^{GMR}$: R ≤ NIM<br><br>Where R is the ratio of GMC (i.e., GMC for ARCT-154 arm / GMC for the ChAdOx1 arm) and NIM is the acceptable noninferiority margin (0.67). The null hypothesis will be rejected if the lower bound of the 95% confidence interval for GMR is > 0.67.<br><br>Assuming a GMR of ≥0.9 and a coefficient of variation of variation of 4.6, a sample size of 800 participants in Phase 3c-1 will provide greater than 90% power to exclude a non-inferiority boundary of 0.67 based on the use of a one-sided test at the alpha=0.025 level of significance, accounting for a dropout rate of approximately 20-25% due to the national COVID-19 vaccination campaign in Vietnam.<br><br>A sequential/hierarchical testing procedure will be used to control type 1 error rate over the Phase 3c primary endpoint and the secondary endpoints such that the secondary immunogenicity endpoints will only be tested if the null hypothesis is rejected for the primary endpoint and Phase 3c secondary endpoints numbers 1 through 5 will be tested sequentially using the same hierarchical test procedure with each endpoint only being tested if the null hypothesis is rejected for the preceding endpoint.<br><br>**Missing Data**<br><br>How missing data points will be handled will be described in the SAP. |

## 1.1 Study Schematic

The study schematic is presented in Figure 1.

**Figure 1 Study Schematic**

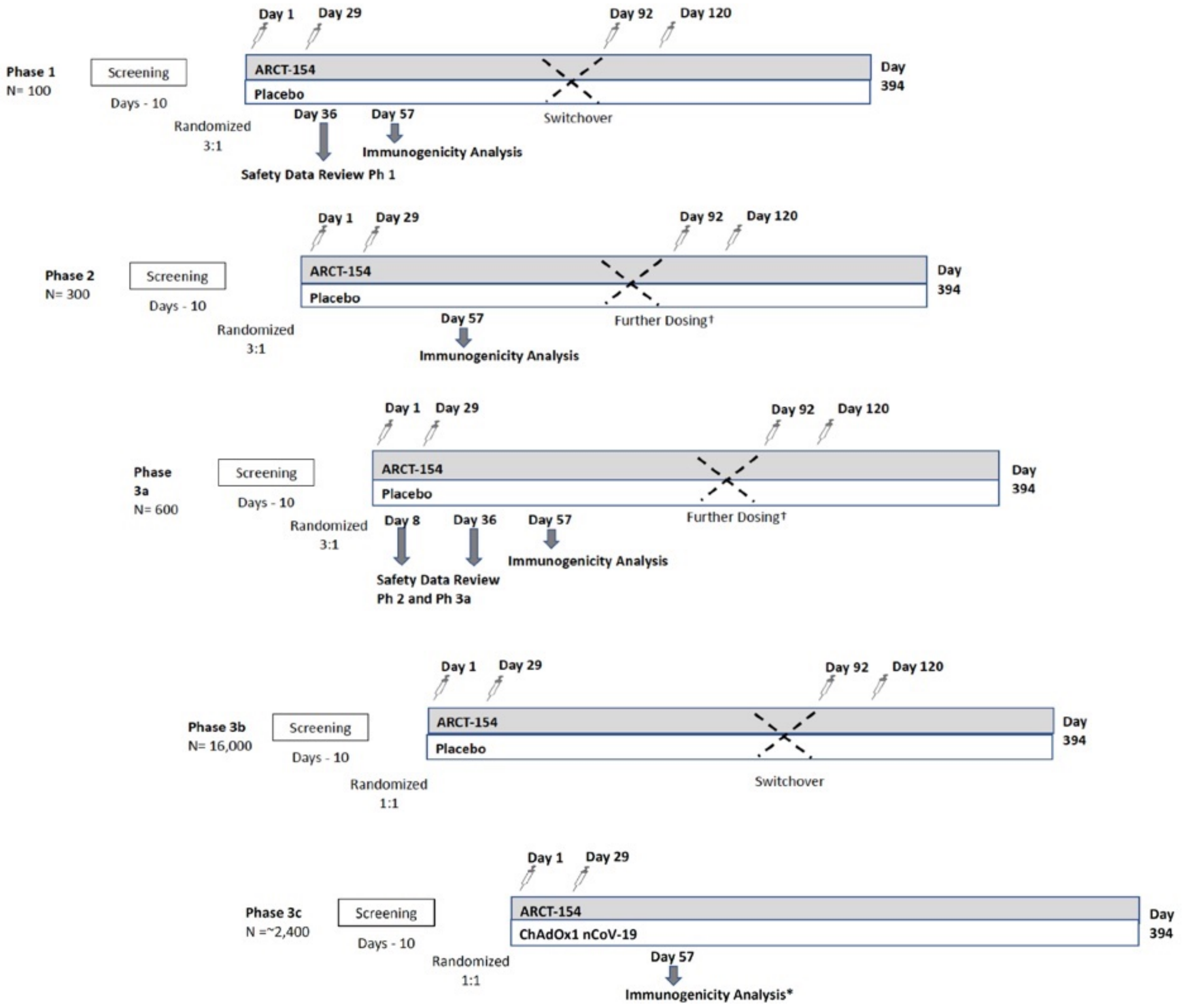


†Participants in Phase 2 and 3a that are randomized to receive ARCT-154 as the initial vaccination series will be further randomized to receive either ARCT-154 or placebo (in a 3:1 ratio) at Day 92 followed by placebo at Day 120. Phase 2 and 3a participants that received placebo initially and Phase 1 and 3b participants switch over to the opposite vaccine at Day 92 and will receive this vaccine at both Day 92 and Day 120.
*Immunogenicity analysis will involve approximately 800 participants enrolled in Phase 3c-1.

# 2 INTRODUCTION

## 2.1 Background

In January 2020, a novel variant of coronavirus was identified as the cause of an outbreak of severe pneumonia in China. This virus, severe acute respiratory syndrome coronavirus 2 (SARS-CoV-2), was identified as the causative agent of a broad clinical spectrum of disease.

Both symptomatic and asymptomatic infection in humans is caused by the SARS-CoV-2 virus, which is a positive strand ribonucleic acid (RNA) virus. A serious manifestation of the infection is a viral pneumonia that can progress to acute respiratory distress syndrome (ARDS), respiratory failure, and death. Clinical symptoms relating to other organ systems have also been described (Zhang 2020; Zaim 2020). Risk factors for more severe disease include age, male sex, pregnancy, ethnic minority status, and comorbidity, particularly chronic obstructive pulmonary disease, cancer, heart conditions (congestive heart failure, pulmonary hypertension, and coronary artery disease), immunocompromise, chronic renal failure, Type 2 diabetes, and obesity (CDC 2020b; De Lusignan 2020; Williamson 2020; Wu 2021; Yang 2020; Li 2020; Zhou 2020; Grasselli 2020). Patients with severe lung disease require ventilatory support and mortality rates as high as 25% to >90% have been reported in these patients (Zhou 2020; Richardson 2020; Auld 2020; Phua 2020), although more recently mortality rates may be reducing as more is known about how best to manage severe disease with treatments such as recently authorized targeted monoclonal therapies (etsevimab, casirivimab, and imdevimab) and application of other therapeutic agents (remdesivir, tocolizumab, baricitinib, and corticosteroids) (NIH 2021).

Over 188 million cases of coronavirus disease 2019 (COVID-19) have been confirmed worldwide and 4.1 million people have died as of July 2021 (WHO 2021), and the number of cases continues to grow in most countries.

While the global incidence of COVID-19 appears to be slowing and some vaccines to prevent COVID-19 have been approved by health authorities of several countries in recent months, the number of cases remains unacceptably high in most countries, and there are significant supply limitations for these vaccines. More global access to vaccines to prevent COVID-19 is clearly needed.

## 2.2 Current Therapies for COVID-19

COVID-19 is a complicated disease and optimal management is still evolving. As such, a comprehensive discussion is beyond the scope of this document. However, a number of treatment guidelines exist (WHO 2020; NIH 2020; Bhimraj 2020). Treatment of less severe disease involves isolation and largely symptom management. Severe disease requires supplemental oxygen ± mechanical ventilation, prevention of thromboembolic complications, and treatment of secondary infection. Progress has been made in the development and emergency use authorization of targeted monoclonal antibodies directed against SARS-CoV-2 (NIH 2021); however, the clinical application of these monoclonal antibodies is limited to non-hospitalized patients with mild to moderate symptoms and high risk of progression to severe COVID-19 disease (NIH 2021; FDA 2021a; FDA 2021b). Application of -anti-inflammatory agents, such as

steroids, Interleukin-6 inhibitors, and Janus kinase inhibitors, have improved outcomes in hospitalized patients (NIH 2021).

A critical component to addressing morbidity and mortality due to SARS-CoV-2 is the development of vaccines to prevent the spread of infection. In late 2020 and in early 2021, multiple companies have shared the results of pivotal trials examining vaccine candidates intended to prevent COVID-19 disease. This has led to the emergency use authorization approvals of several inactivated SARS-CoV-2 viral (Global Regulatory Partners 2021; Reuters 2021a; WashingtonPost 2020), SARS-CoV-2 peptide (Turkmenistan Today 2021), SARS-CoV-2 receptor binding domain (RBD) dimer (RBD dimer), adenoviral vector (EMA 2021a; EMA 2021b; Reuters 2021b; Burki 2020), and non-self-replicating messenger RNA (mRNA) vaccines (FDA 2020b; FDA 2020c; EMA 2021c). While each of these vaccines has demonstrated promising early vaccine efficacy and these vaccines have been used in mass vaccination efforts, there remains a significant ongoing circulation of SARS-CoV-2, and vaccine rollout campaigns will take months or more across the globe. In this current situation of ongoing infection, lack of herd immunity, and incomplete vaccine rollout, there exists a new risk of evolutionary selection pressure on SARS-CoV-2.

Ongoing global demand for vaccines is being fueled by the concern that the continued circulation of SARS-CoV-2 has led to the development of variants, such as D614G, and may ultimately become endemic. With these additional challenges in mind, it will be necessary to characterize vaccine durability of response as well as immunogenicity against homologous and heterologous SARS-CoV-2 strains, and to continue mass vaccinations to limit morbidity and mortality associated with SARS-CoV-2 (Sah 2021).

## 2.3 Therapeutic Rationale for ARCT-154 in Prevention of COVID-19

SARS-CoV-2 is a novel virus belonging to the β-coronavirus genus. Coronavirus host cell infection is mediated by the attachment of the transmembrane spike (S) glycoprotein to host cell receptors and subsequent fusion with host cell membranes. The S glycoprotein forms homotrimers protruding from the viral surface (Tortorici 2019) and can be divided into 4 subdomains; S1, S2, transmembrane, and internal domain or endodomain. The S1 domain contains the RBD, which allows SARS-CoV-2 to directly bind to the peptidase domain of the angiotensin-converting enzyme 2 (ACE-2) receptor expressed on epithelial cells in the lungs, heart, kidneys, and gastrointestinal tract. Hence, antibodies to the S glycoprotein, especially the RBD, should block viral entry into cells expressing the ACE-2 receptor and thereby prevent infection.

Two conventional mRNA vaccines have established marked vaccine efficacy, and the data from the pivotal trials establishing vaccine efficacy has led to the emergency use authorization and/or conditional marketing authorizations of these vaccines (see FDA 2020b; FDA 2020c; EMA 2020; EMA 2021a). However, the ability of post-vaccination serum from these vaccines to neutralize the B.1.351 and D614G variants is reduced (Zhou 2021; Wu 2021; Zou 2021), resulting in concerns that they may not provide such effective protection against this variant. Evaluation of additional mRNA vaccines, the pursuit of an immune correlate of protection, and characterization of the vaccine efficacy of these other mRNA vaccines against variants of concern remain ongoing at the time of this protocol finalization.

## 2.4 Mechanism of Action

Arcturus Therapeutics has developed a series of vaccines intended for the prevention of COVID-19 disease based on its mRNA technology. Two of these vaccines are referred to as ARCT-154 (the vaccine that will be used in this study) and ARCT-021 (a vaccine that is currently under evaluation in other clinical studies).

ARCT-021 and ARCT-154 each contain Arcturus' proprietary self-transcribing and -replicating RNA (STARR™) technology, an RNA replicon construct based on the alphavirus, Venezuelan equine encephalitis virus (VEEV). The replicon for ARCT-021 (mRNA-2002) consists of a replicase gene and an RNA sequence encoding the Wuhan strain SARS-CoV-2 S glycoprotein. The replicon for ARCT-154 (mRNA-2105) consists of a replicase gene and an RNA sequence encoding the SARS-CoV-2 S glycoprotein containing the D614G variant mutation. The final drug product for both vaccines includes an RNA replicon formulated with Arcturus' proprietary lipid nanoparticle (LNP) technology (LUNAR®), including 4 different lipid nanoparticles. More detail relating to these vaccines is included in the Investigator's Brochure (IB).

Alphaviruses are enveloped viruses with a positive strand RNA genome. Upon infection, the genomic RNA serves as a template for translation of 4 viral nonstructural proteins that form replicase complexes. These complexes synthesize viral genomic and subgenomic RNA, the latter of which serves as a template for translation of viral structural proteins, which then assembles with genomic RNA into new infectious viral particles. By replacing the RNA coding for structural proteins with RNA coding for a protein antigen of interest (in this case the SARS-CoV-2 D614G S glycoprotein), the self-replicating machinery of the alphavirus can be used to generate sustained expression of the antigen, making such alphavirus replicon constructs an attractive tool for vaccines. These replicon RNAs, which do not encode the compliment of structural genes necessary for assembly of virus particles and do not contain reverse transcriptase that converts the RNA genome into complementary deoxyribonucleic acid (DNA), replicate exclusively in the cytoplasm and cannot introduce their genetic material into the cellular genome. A more detailed description of the mechanism of mRNA amplification and the biochemical functions for each of the nonstructural proteins is described in Rupp 2015.

On entry into the cytoplasm, the replicase gene, encoding the 4 nonstructural proteins (nsPs) (nsP1 to nsP4), is translated from mRNA-2105 producing only the replicase proteins as a single polyprotein. The RNA-dependent RNA polymerase, nsP4, is released from the polyprotein and, in combination with the remaining nsP123 polyprotein, transcribes the complementary RNA strand of the entire mRNA-2105, including the SARS-CoV-2 S glycoprotein RNA and poly A tail. The remaining polyprotein is processed into nsP1, nsP2, and nsP3 and, in combination with nsP4, transcribes only the 5'-G-methyl capped S glycoprotein mRNA from the 3'-5' complementary strand of mRNA-2105. The multiple copies of S glycoprotein mRNA transcript are then translated to produce full-length S glycoprotein, which is the vaccine antigen. Cleavage of the nsP123 polyprotein into its component nsPs terminates transcription of the complementary RNA (Rupp 2015). Remaining complementary RNA and S glycoprotein mRNA is then degraded by intracellular nucleases thereby terminating production of the antigen.

During the process of self-replication, a double-stranded RNA intermediate is produced that has immunostimulatory properties activating the innate immune system, ultimately enhancing the adaptive immune response to the expressed S glycoprotein and thereby behaving as an adjuvant.

At the injection site, the LNP-formulated RNA is taken up by antigen-presenting cells and myocytes, the former of which traffic to regional lymph nodes where they present the vaccine antigen to CD4+ T cells and CD8+ T cells, resulting in their activation. Antigen can be detected in regional lymph nodes within hours of injection (Liang 2017; Lindsay 2019). Interleukin-2 from activated CD4+ T cells is subsequently important in the terminal differentiation of the activated, antigen-specific CD8+ T cells (Zhang 2011). Although the immunological events leading to antibody responses elicited by RNA vaccines have yet to be fully elucidated, T follicular helper (Tfh) cells, which are a subset of CD4+ cells, are required to develop germinal center responses and drive immunoglobulin class switch, affinity maturation, and long-term B cell memory (Pardi 2018) and strong induction of antigen-specific Tfh cells has been demonstrated following vaccination with mRNA vaccines (Lindgren 2017).

## 2.5 Nonclinical Experience with ARCT-154

Nonclinical studies have characterized the protective effects of vaccination with ARCT-021 and the immunogenicity of vaccination with ARCT-021 and ARCT-154 in mouse and non-human primate (NHP) models. For full details, please see the IB.

Immunogenicity data from NHP support that two doses of ARCT-154 lead to high antibody titers. As shown below, surrogate virus neutralization (sVNT) Ab responses for four strains of SARS-CoV-2, including variants of concern, exceed 87% in all four SARS-CoV-2 strains evaluated (Figure 2), and pseudovirus neutralization titers confirm robust responses to VOC, including the delta variant (Figure 3).

**Figure 2 sVNT Antibody Responses to ARCT-154 in Non-Human Primates**

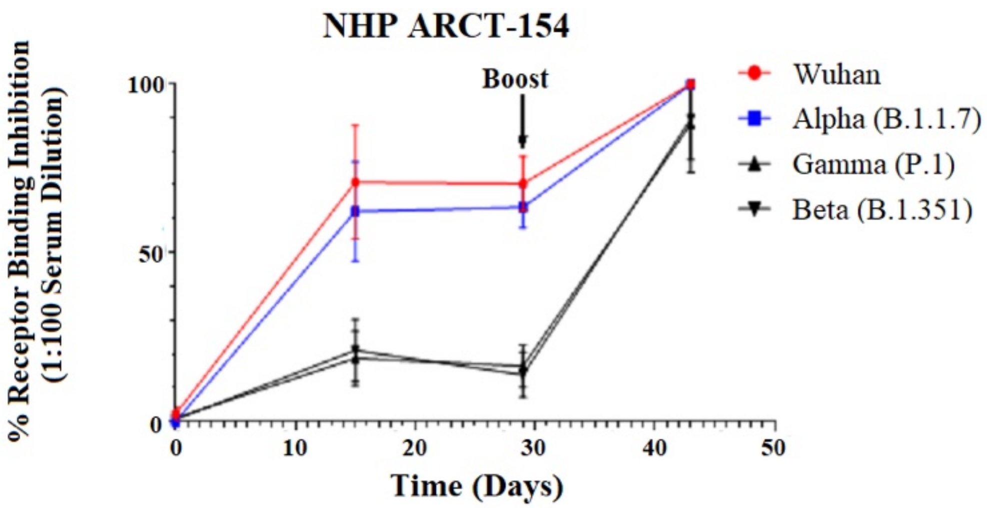

**Figure 3 Pseudoneutralization Data from NHPs After Two Doses of ARCT-154 Vaccine**

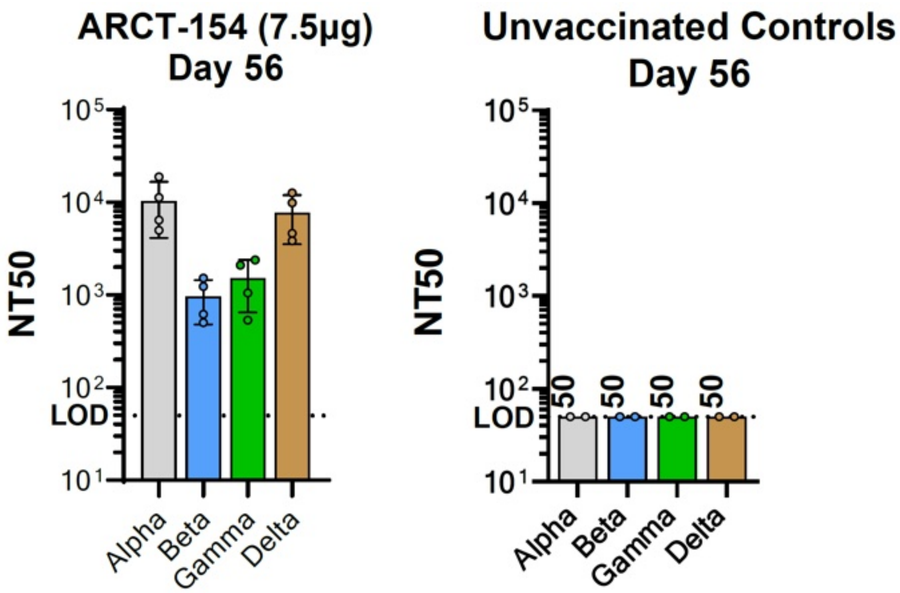


Results from the Saphire laboratory (La Jolla Institute for Immunology) using VSV pseudotyped with variant SARS-CoV-2 spike proteins

Because the principal differences between ARCT-021 and ARCT-154 are primarily limited to the SARS-CoV-2 variant spike protein that is expressed, these changes only constitute a change of antigen within a platform, consistent with the "platform" approach described in relevant regulatory guidances (FDA 2020d; EMA 2021d). As such, nonclinical tissue distribution data, Good Laboratory Practices toxicology data, and prior human clinical trial data generated with ARCT-021 are used to support clinical development of ARCT-154. Please refer to the IB for a description of the results of nonclinical studies conducted with ARCT-021.

## 2.6 Clinical Trial Experience with the LUNAR COVID-19 Vaccines (ARCT-021 and ARCT-154)

At the time of finalization of this protocol (Version 1.0), no clinical trials have been conducted with ARCT-154. However, ARCT-154 is based upon the similar LNP-formulated RNA vaccine construct, ARCT-021, which has been evaluated in 3 clinical trials. Collectively, ARCT-154 and ARCT-021 are referred to as the LUNAR COVID-19 vaccines throughout this protocol. The 3 ARCT-021 clinical trials include a Phase 1/2 clinical trial (ARCT-021-01) in healthy adult participants in Singapore, which has completed (final report pending); an open-label extension trial (ARCT-021-02) following on from ARCT-021-01, which is ongoing, and a Phase 2 trial (ARCT-021-04), which is ongoing in the USA and Singapore. The interim analyses of these studies, which have enrolled more than 600 participants, is described in detail in the IB.

ARCT-021 has been administered to more than 500 participants in these clinical trials. The data that are shared in this protocol are regarded as draft but inform the characterization of the safety and immunogenicity profile and thereby the preferred dose for use in later-phase trials. The data from the ARCT-021-04 trial have been shared with the independent DSMB that has endorsed the continuation of the study without modification.

Analysis of the open-label extension study ARCT-021-02 has not yet been conducted.

At the time of second Interim Analysis, the Phase 2 study (ARCT-021-04) had completed enrollment of 426 participants, with no SAEs related to study vaccine, and 8 AEs which led to discontinuation. Across the analyzed clinical trials to date, ARCT-021 has been generally safe and well tolerated at doses ≤7.5 µg.

Most (>90%) ARCT-021-treated participants in each study experienced at least one solicited AE. In each study, the majority of solicited events were Grade 1/mild or Grade 2/moderate. A single participant reported Grade 4 solicited events (dizziness, fatigue, nausea, chills, headache).

The most common solicited AEs were injection site pain, injection site tenderness, headache, fatigue, myalgia, arthralgia and chills. The majority of solicited events had onset on the day after dosing and were no longer reported by the end of 7 days post-dose.

Solicited AE reporting does not appear to increase with second dose of ARCT-021 and solicited AE reporting may be less frequent in older participants

Unsolicited AE reporting is less frequent following ARCT-021 as compared to solicited AEs. The most common unsolicited AEs in both studies include headache, fatigue, injection site tenderness, diarrhea, elevated CPK, neutropenia, lymphopenia, urticaria and hematuria (which was not confirmed on microscopy).

Overall, no trends for vaccine-related abnormalities were observed in ECGs (evaluated in the ARCT-021-01 study only) or vital signs following vaccination with ARCT-021. Other safety laboratory assessments with patterns of interest include declining hemoglobin values (most likely due to blood sampling during trial participation), slight shifts in potassium values, and slight shifts in lymphocyte and neutrophil count. These laboratory abnormalities have been asymptomatic, and the minority have been reported as clinically significant changes.

Transient, asymptomatic lymphopenia and neutropenia have been observed with other mRNA SARS-CoV-2 vaccines (Mulligan 2020; Sah 2021; Sahin 2020; VRBPAC 2020), however, lymphopenia is thought to be a normal physiological response (innate immune-stimulation-related redistribution of lymphocytes into lymphoid tissues) to immune stimulation from the vaccine (Sah 2021; Sahin 2020).

Immunogenicity results following vaccination with ARCT-021 showed a robust IgG immune response to the full-length SARS-CoV-2 spike glycoprotein at all doses evaluated. At doses ≥3 µg, GMT for PRNT50 neutralizing antibody responses tested with a clinical isolate of SARS-CoV-2 were within the range of titers observed in convalescent plasma from COVID-19 patients tested in the same assay at the same laboratory. Spike-specific T-cell responses were observed in response to stimulation with peptides from the spike glycoprotein and the CD4+ response was Th1-dominant.

The evaluation of safety data across the hundreds of participants dosed with ARCT-021 demonstrates that the vaccine leads to mild to moderate, transient AEs following vaccination for the majority of individuals. Overall AE reporting may decrease with second vaccination.

Taken together, the favorable safety profile and clear signs of immunogenicity to the vaccine antigen of ARCT-021, given the fundamental similarities between the ARCT-021 and ARCT-154 vaccines and, given the encouraging nonclinical data supporting immune responses to SARS-CoV-2 variants, support the continued clinical development of these vaccines intended to prevent COVID-19.

## 2.7 Rationale for Dose Regimen Selection

The dose and schedule of ARCT-154 used in this study are based on the evaluation of both preclinical and clinical data arising from the ARCT-021 program. As ARCT-154 includes the same lipid nanoparticle formulation and RNA encoding for a replicon and a SARS-CoV-2 spike protein (albeit spike proteins with minor differences in amino acid composition), the safety and immunogenicity profile is expected to be informed by the experience with ARCT-021.

The doses selected for the vaccination series in this study are based on data from the following sources:

- Study ARCT-021-01: a Phase 1/2 dose ranging study enrolling younger adults (21 to 55 years of age) and older adults (56 to 80 years of age). In this study, 2 doses of 5.0 µg (separated by 28 days) were well tolerated and immunogenic in younger and older adults.
- Repeat-dose toxicity studies in rabbits: in these studies, rabbits were given 2 or 3 doses of either 20 µg or 40 µg ARCT-021 separated by 14 days. In these dosed animals, ARCT-021 was associated with continued increases in anti SARS-CoV-2 IgG levels and the NOAEL was assigned at the highest dose evaluated (40 µg) based on the safety profile. These data support the administration of up to 3 doses of ARCT-154 (including the two priming vaccination doses of 5.0 µg ARCT-154 planned for administration to participants in this study).

Administration of a 3$^{rd}$ dose of ARCT-154 to participants in Phase 2/3a allows the opportunity to evaluate the neutralizing antibody of activity of a 3$^{rd}$ (booster) dose of ARCT-154 against variants of concern, and in particular the newly identified omicron variant. Early data indicates that neutralizing antibody activity against omicron following two doses of other RNA vaccines may be substantially less than against other variant strains, but that activity following a third dose is considerably improved (Pfizer 2021).

Further details on ARCT-021 and ARCT-154 can be found in the Investigator's Brochure.

## 2.8 Risk: Benefit Assessment

### 2.8.1 Potential Risks

Detailed information about the known and expected benefits and risks and reasonably expected AEs of the LUNAR COVID-19 vaccines is provided in the IB. The expected benefits and risks of ChAdOx1 are summarized in the approved label for this vaccine. This reference material is

provided separately. In addition to the risk mitigation measures incorporated into the study design, a summary of the potential risks of treatment with the LUNAR COVID-19 vaccines, together with potential mitigation for these risks, is shown in Table 1.

**Table 1 Risk Minimization Measures Included in Clinical Trials of LUNAR COVID-19 Vaccines**

| Risk | Mitigation |
|---|---|
| Local and Systemic Reactogenicity | • Vaccination via the intramuscular route results in a lower rate of local reactions than intradermal (I.D.) or subcutaneous (S.C.) injection (Zhang 2015).<br>• Local injection site reactions and systemic AEs such as headache, fever, myalgia, or arthralgia may be treated with acetaminophen (paracetamol), ibuprofen, or other non-steroidal anti-inflammatory drugs and/or with topical agents (eg, ice or heat); however, administration of these agents within 24 hours prior to study vaccine administration is prohibited. Allergic AEs may be treated with corticosteroids or H1/H2 blockers as indicated and if not otherwise contraindicated.<br>• For anaphylaxis or immediate type hypersensitivity (≤4 hours after injection) of at least moderate severity following the administration of the study vaccine, subsequent doses of study vaccine will not be administered. |
| VAERD if subsequently infected with SARS-CoV-2 | • Risk minimization measures to date have included ongoing characterization of the immune responses in nonclinical and clinical studies. These demonstrate that vaccination with ARCT-021 leads to reduction in viral replication (NHP and mouse data), protection from COVID-19 disease (NHP and mouse data), no evidence of lung injury (NHP data), and Th1-biased immune response (mouse and human data).<br>• In this study, an independent DSMB or other safety committee will assess all cases of severe COVID-19 for imbalance in order to assess the risk of VAERD. The DSMB will make recommendations concerning modifying or stopping the study in order to manage any risk(s) identified |

Abbreviations: AE=adverse events; COVID-19=coronavirus disease 2019; DSMB=Data and Safety Monitoring Board; H=histamine; I.D.=intradermal; SARS-CoV-2=severe acute respiratory syndrome coronavirus 2; S.C.=subcutaneous; Th=T helper; Th1=T helper Type 1; VAERD=vaccine-associated enhanced respiratory disease

Other potential risks of trial participation beyond those associated with study vaccine include risks relating to blood sampling and breach of confidentiality.

Blood sampling may be associated with transient asthenia, risk of fainting, and bruising or infection at the blood sampling site. Risk of infection is reduced by cleansing of the skin prior to blood withdrawal. Risk of fainting is reduced by performing blood sampling with the participant in a seated position.

As study participation involves provision of personal health information by the participant to the site staff, measures are in place to reduce the risk of sharing of this personal information with others. Measures include maintenance of personal information in secure locations (eg, locked file cabinets and/or password-protected electronic filing systems). Only personnel involved in the conduct, oversight, monitoring, or auditing of this trial will be allowed access to this personal information that is collected. All publications from this study will not disclose personal information of the participants. Study results disclosed in public locations, such as ClinicalTrials.gov, will not include personal information of any enrolled participant.

There may be other risks, discomforts, or side effects from participation in this study that are currently unknown.

### 2.8.2 Potential Benefits

As the ARCT-154 has not yet been licensed for approval, the benefit of the vaccine has not been reviewed and approved by a health authority. Therefore, there is no direct benefit to study participants receiving ARCT-154 or placebo beyond a potential benefit to society resulting from improved understanding of investigational vaccines to prevent COVID-19 disease. For participants enrolled in Phase 3c, the benefit of study participation includes the possibility of randomization to an authorized vaccine (ChAdOx1) that may reduce the risk of COVID-19 disease. However, vaccination with either ARCT-154 or ChAdOx1 may or may not provide protection against SARS-CoV-2 infection and COVID-19 disease. Vaccination with placebo is not expected to provide protection against SARS-CoV-2 infection. For participants in Phase 1 and 3b and placebo participants in Phase 2 and Phase 3a, the study design includes a Switchover component. This allows participants who remain in the study to receive 2 doses of both study vaccines (ARCT-154 and placebo). While it is much preferred that participants forego vaccination against COVID-19 with other non-study vaccines during study participation, participants will not be denied access to these vaccines. Participants who elect to receive a non-study COVID-19 vaccine (Section 6.8.1) that is approved for use in Vietnam, recommended for their age group/underlying medical condition, and that is made available to them will not be allowed to participate in the Switchover portion of the study but will be asked to continue study -related procedures (Section 5.2.2).

### 2.8.3 Overall Risk: Benefit Assessment

Preclinical and clinical studies have been initiated with investigational LUNAR COVID-19 vaccines intended to prevent COVID-19 disease. This protocol includes measures taken to minimize risks to potential study participants. In addition, the available data reflect that the LUNAR COVID-19 vaccines are immunogenic and well-tolerated. This favorable risk/benefit profile supports further evaluation of ARCT-154 in this Phase 3 clinical study.

# 3 OBJECTIVES AND ENDPOINTS

Note, full details of the analysis populations and rules for handling intercurrent events are included in Sections 8.2 and 8.3.1, respectively.

In interpreting the objectives and endpoints for this study, the following considerations will apply:

- The overall primary efficacy endpoint for the study is vaccine efficacy (VE) as evaluated in the Phase 3b Modified Intent to Treat (mITT) population
- The overall primary safety endpoints are those evaluated in the Phase 3b Safety Analysis Set (SAS) and Phase 3b Reactogenicity Analysis Set (RAS)
- Phase 1/2/3a and subgroups thereof constitute a substudy with separate objectives and endpoints. As these constitute a substudy, and there is no evaluation of efficacy for this substudy, no adjustment of type 1 error is applied to the overall efficacy primary endpoint
- The Pooled Efficacy endpoint is only evaluated as exploratory, so no adjustment of type 1 error is applied to the overall efficacy primary endpoint
- Phase 3c and subgroups thereof also constitute a substudy with separate objectives and endpoints. As these constitute a substudy, and efficacy for this study is only evaluated as exploratory, no adjustment of type 1 error is applied to the overall efficacy primary endpoint.

## 3.1 Phase 1/2/3a Substudy

Immunogenicity data from participants in Phases 1, 2 and 3a will be pooled for analysis up to Day 92. With implementation of protocol version 8.0, participants in Phase 2 and 3a that received ARCT-154 in the initial vaccination series are further randomized to receive a 3rd vaccination of either ARCT-154 or placebo at Day 92 followed by placebo at Day 120 in order to compare immunogenicity after 3 injections of ARCT-154 with that after 2 injections. Therefore, after Day 92 only data from Phase 2/3a will be pooled and will be displayed separately from participants in Phase 1.

Safety data from participants in Phase 2/3a will be pooled for all time points and will be displayed separately to safety data from Phase 1.

### 3.1.1 Phase 1/2/3a Substudy Primary Objectives and Endpoints

The primary objectives and endpoints for Phase 1/2/3a are presented in Table 2.

**Table 2 Phase 1/2/3a Substudy Primary Objectives and Endpoints**

| Phase 1/2/3a Primary Objectives | Endpoint Description |
|---|---|
| 1. To assess the safety and reactogenicity of ARCT-154 compared to placebo | Safety will be evaluated in all participants in Phase 1 and Phase 2/3a Safety Analysis Sets (SAS, Section 8.1) and will be summarized for each vaccination as number and percentage of participants with:<br>• Any unsolicited AE starting within 28 days after each study vaccine administration, summarized by severity and relationship to study vaccine.<br>• Any medically attended adverse event (MAAE), serious adverse event (SAE), or AE leading to discontinuation/withdrawal through Final Visit/Early Termination (ET) summarized by relationship to study vaccine.<br>Reactogenicity will be evaluated in in the Phase 1 and Phase 2/3a Reactogenicity Analysis Sets (RAS) and will be summarized following vaccinations on Day 1 and Day 29 only in Phase 1 and Day 1, Day 29, and Day 92 in Phase 2/3a as the number and percentage of participants with:<br>• Any solicited local or systemic AE starting within 7 days after each study vaccine administration by toxicity grade.<br>Additional safety analyses in the Phase 1/2/3a SAS may also be performed as exploratory. |
| 2. To assess the neutralizing antibody (NAb) responses to ARCT-154 by surrogate virus neutralization test at Day 57 | Neutralizing antibody (NAb) responses by surrogate virus neutralization test (sVNT) will be evaluated at Day 1 (baseline) and Day 57 for assessment of seroconversion in all participants in the Phase 1/2/3a Immunogenicity Analysis Set (IAS (Section 8.2).<br>• The endpoint is defined as the proportion of participants in each study vaccine group that demonstrate seroconversion (defined as: 4-fold increase in antibody concentration from baseline).<br>Data will be summarized according to use of international reference standards, if available. |

Abbreviations: AE=adverse event; COVID-19=coronavirus disease 2019; MAAE=medically attended adverse event; SAE=serious adverse event; ET=early termination

### 3.1.2 Phase 1/2/3a Substudy Secondary Objectives and Endpoints

The secondary objectives and endpoints for Phase 1/2/3a are presented in Table 3.

**Table 3 Phase 1/2/3a Secondary Objectives and Endpoints**

| Phase 1/2/3a Secondary Objectives | Endpoint Description |
|---|---|
| 1. To assess the neutralizing and IgG binding antibody immune responses to ARCT-154 over time | Immunogenicity will be at the following time points:<br>• Phase 1 IAS: Day 1 (baseline), Day 29, Day 57, Day 92, Day 394.<br>• Phase 2/3a IAS: Day 1 (baseline), Day 29, Day 57, Day 92, Day 120, Day 394<br>• Phase 1/2/3a IAS: Day 1 (baseline), Day 29, Day 57, Day 92<br>The following will be evaluated:<br>• Neutralizing antibody (NAb) by surrogate virus neutralization test (sVNT) and IgG antibody binding the full-length SARS-CoV-2 spike protein (binding antibody [BAb]) responses will be evaluated; assessments for both NAb and BAb will include:<br>o Antibody level: GMC (BAb and NAb)<br>o Increase in antibody levels from baseline (GMFR or other measure, as appropriate for the assay concerned)<br>o Proportion of participants in each study vaccine group with seroconversion at each timepoint. Note: NAb responses at Day 57 evaluated as primary.<br>Data will be summarized according to use of international reference standards, if available.<br>Full details will be provided in the statistical analysis plan. |
| 2. To assess early neutralizing antibody responses using a live virus assay | NAb responses by plaque reduction neutralization test at 50% reduction (PRNT50) will be evaluated in the subset of the Phase 1/2 IAS (Section 8.2) comprising all participants enrolled in Phase 1 and the first 50 evaluable participants in Phase 2 at Day 1 (baseline), Day 29 and Day 57.<br>The following endpoints will be assessed:<br>• Geometric mean antibody concentration (GMC)<br>• Increase in antibody levels from baseline (GMFR)<br>• Proportion of participants in each study vaccine group with seroconversion |

Abbreviations: GMT=geometric mean titer; GMC=geometric mean concentration; GMFR=geometric mean-fold rise

### 3.1.3 Phase 1/2/3a Substudy Exploratory Objectives and Endpoints

The exploratory objectives and endpoints for Phase 1/2/3a are presented in Table 4.

**Table 4 Phase 1/2/3a Exploratory Objectives and Endpoints**

| Phase 1/2/3a Exploratory Objectives | Endpoint Description |
|---|---|
| 1. To compare the humoral immune responses to ARCT-154 with those following COVID-19 | Day 57 GMT/GMC results following vaccination with ARCT-154 may be compared with the comparable test results for convalescent sera from COVID-19 patients measured on the same assay for the subset of the Phase 1/2/3a IAS comprising all participants enrolled in Phase 1 and the first 50 evaluable participants in Phase 2. The following assays will be used:<br>• PRNT50<br>• MNT<br>Immune responses may also be assessed at additional time points and/or in additional participants in Phase 2 and Phase 3a. If performed this will be described in the statistical analysis plan (SAP).<br>For the MNT assay, data will be summarized according to use of international reference standards, if available |
| 2. To evaluate the efficacy of ARCT-154 versus placebo for the prevention of virologically confirmed COVID-19 | Efficacy may be evaluated in all participants in the Phase 1/2/3a Modified Intent to Treat (mITT) set (Section 8.1). The endpoint to be evaluated is as follows:<br>• The first occurrence of confirmed, protocol-defined (Appendix 2) COVID-19 with onset between Day 36 and Day 92 inclusive.<br>Efficacy may also be evaluated in participants that have received any dose of study vaccine in the first vaccination series, with no evidence of infection prior to vaccination. The endpoint is as follows:<br>• The first occurrence of confirmed, protocol-defined (Appendix 2) COVID-19 with onset at any time after the first study vaccination and up to Day 92. |
| 3. To assess the neutralizing antibody immune responses to ARCT-154 to SARS-CoV-2 variants of concern/variants of interest following 2 and 3 vaccinations with ARCT-154 | Blood samples from post-vaccination timepoints may be evaluated for immune responses to SARS-CoV-2 variants of concern/variants of interest for the following groups/time points:<br>• The subset of the Phase 1/2/3a IAS comprising all participants enrolled in Phase 1 and the first 50 participants in Phase 2; evaluated at Day 1 (baseline) and Day 57.<br>• The subset of the Phase 2/3a IAS comprising the first 150 participants that received ARCT-154 in the initial vaccination series in Phase 2; evaluated at Day 1 (baseline), Day 57 and Day 120.<br>The following assays may be used:<br>• PRNT50<br>• sVNT<br>• MNT |

**Table 4 Phase 1/2/3a Exploratory Objectives and Endpoints**

| | |
|---|---|
| 4. To assess neutralizing antibody responses using a pseudovirus microneutralization test (MNT) following 2 (Day 57) and 3 (Day 120) vaccinations with ARCT-154 | NAb responses by microneutralization test (MNT) will be evaluated for the following groups/time points:<br>• The subset of the Phase 1/2/3a IAS comprising all participants enrolled in Phase 1 and the first 50 evaluable participants in Phase 2 at Day 1 (baseline) and Day 57.<br>• The subset of the Phase 2/3a IAS comprising the first 150 participants that received ARCT-154 in the initial vaccination series in Phase 2; evaluated at Day 1 (baseline), Day 57 and Day 120.<br>The following endpoints will be assessed:<br>o Geometric mean antibody concentration (GMC)<br>o Increase in antibody levels from baseline (GMFR)<br>o Proportion of participants in each study vaccine group with seroconversion.<br>Data will be summarized according to use of international reference standards, if available. |
| 5. To assess the neutralizing antibody (NAb) responses by surrogate virus neutralization test to ARCT-154 following 2 (Day 57) and 3 (Day 120) vaccinations with ARCT-154 in Phase 2/3a participants | Neutralizing antibody (NAb) responses by surrogate virus neutralization test (sVNT) will be evaluated at Day 1 (baseline), Day 57 and Day 120 for assessment of seroconversion in all participants in the Phase 2/3a IAS that received ARCT-154 in the initial vaccination series<br>• The endpoint is defined as the proportion of participants that demonstrate seroconversion (defined as: 4-fold increase in antibody concentration from baseline).<br>Data will be summarized according to use of international reference standards, if available. |
| 6. To assess the IgG binding antibody responses to ARCT-154 following 2 (Day 57) and 3 (Day 120) vaccinations with ARCT-154 in Phase 2/3a participants | IgG antibody binding the full-length SARS-CoV-2 spike protein (binding antibody [BAb]) responses will be evaluated at Day 1 (baseline), Day 57 and Day 120 in all participants in the Phase 2/3a IAS that received ARCT-154 in the initial vaccination series.<br>The following endpoints will be assessed:<br>o Geometric mean antibody concentration (GMC)<br>o Increase in antibody levels from baseline (GMFR)<br>o Proportion of participants in each study vaccine group with seroconversion.<br>Data will be summarized according to use of international reference standards, if available. |

Abbreviations: COVID-19=coronavirus disease 2019

## 3.2 Phase 3b

### 3.2.1 Phase 3b Primary Objectives and Endpoints

The primary objectives and endpoints for Phase 3b are presented in Table 5. These form the primary objectives and endpoints for the study overall.

**Table 5 Phase 3b Primary Objectives and Endpoints**

| Phase 3b Primary Objectives | Endpoint Description |
|---|---|
| 1. To assess the safety and reactogenicity of ARCT-154 compared to placebo | Safety will be evaluated in all participants in the Phase 3b SAS and will be summarized for each vaccination as number and percentage of participants with:<br>• Any unsolicited AE starting within 28 days after each study vaccine administration, summarized by severity and relationship to study vaccine.<br>• Any medically attended adverse event (MAAE), serious adverse event (SAE), or AE leading to discontinuation/withdrawal through Final Visit/Early Termination (ET) summarized by relationship to study vaccine.<br>Reactogenicity will be evaluated in the Phase 3b RAS and will be summarized following vaccinations on Day 1 and Day 29 only as the number and percentage of participants with:<br>• Any solicited local or systemic AE starting within 7 days after each study vaccine administration by toxicity grade. |
| 2. To evaluate the efficacy of ARCT-154 and placebo for the prevention of virologically confirmed COVID-19 | Efficacy will be evaluated in all participants in the Phase 3b mITT. The endpoint to be evaluated is as follows:<br>• The first occurrence of confirmed, protocol-defined (Appendix 2) COVID-19 with onset between Day 36 and Day 92 inclusive. |

Abbreviations: AE=adverse event; COVID-19=coronavirus disease 2019; MAAE=medically attended adverse event; SAE=serious adverse event; ET=early termination

### 3.2.2 Phase 3b Secondary Objectives and Endpoints

The exploratory objectives and endpoints for Phase 3b are presented in Table 6.

**Table 6 Phase 3b Secondary Objectives and Endpoints**

| Phase 3b Secondary Objectives | Endpoint Description |
|---|---|
| 1. To evaluate the efficacy of ARCT-154 and placebo for the prevention of virologically confirmed severe COVID-19 | Efficacy will be evaluated in all participants in the Phase 3b mITT. The endpoint to be evaluated is as follows:<br>• The first occurrence of confirmed, protocol-defined (Appendix 2) severe COVID-19 with onset between Day 36 and Day 92 inclusive. |

### Table 6 Phase 3b Secondary Objectives and Endpoints

| Phase 3b Secondary Objectives | Endpoint Description |
|---|---|
| 2. To evaluate the efficacy of ARCT154 and placebo for the prevention of virologically confirmed COVID-19 at any time after first vaccination | Efficacy will be evaluated in all participants in the Phase 3b mITT who have received any dose of study vaccine in the first vaccination series, with no evidence of infection prior to vaccination. The endpoint is as follows:<br>• The first occurrence of confirmed, protocol-defined (Appendix 2) COVID-19 with onset at any time after the first study vaccination and up to Day 92 inclusive. |
| 3. To evaluate the efficacy of ARCT-154 versus placebo for the prevention of death due to COVID-19 | Efficacy will be evaluated in all participants in the Phase 3b mITT. The endpoint is as follows:<br>• The endpoint is the occurrence of death attributed to COVID-19 occurring between Day 36 and Day 92 inclusive. |
| 4. To evaluate the efficacy of ARCT-154 and placebo for the prevention of virologically confirmed COVID-19 regardless of baseline status for evidence of prior SARS-CoV-2 infection | Efficacy will be evaluated in all participants in the Phase 3b mITT. The endpoint to be evaluated is as follows:<br>• The first occurrence of confirmed, protocol-defined (Appendix 2) COVID-19 with onset between Day 36 and Day 92 inclusive. |

Abbreviations: COVID-19=coronavirus disease 2019; SARS-CoV-2=severe acute respiratory syndrome coronavirus 2

### 3.2.3 Phase 3b Exploratory Objectives and Endpoints

The Phase 3b exploratory objectives and endpoints for the study are presented in Table 7.

### Table 7 Phase 3b Exploratory Objectives and Endpoints

| Phase 3b Exploratory Objectives | Endpoint Description |
|---|---|
| 1. To evaluate the efficacy of ARCT-154 and placebo for the prevention of virologically confirmed severe COVID-19 at any time after first vaccination | Efficacy may be evaluated in all participants in the Phase 3b mITT that have received any dose of study vaccine in the first vaccination series, with no evidence of infection prior to vaccination. The endpoint is as follows:<br>• The first occurrence of confirmed, protocol-defined (Appendix 2) severe COVID-19 with onset at any time after the first study vaccination and up to Day 92. |
| 2. To evaluate the efficacy of ARCT-154 and placebo for the prevention of death due to COVID-19 at any time after first vaccination | Efficacy may be evaluated in all participants in the Phase 3b mITT that have received any dose of study vaccine in the first vaccination series. The endpoint is as follows:<br>• The occurrence of death attributed to COVID-19 occurring at any time after the first study vaccination and up to Day 92. |

**Table 7 Phase 3b Exploratory Objectives and Endpoints**

| Phase 3b Exploratory Objectives | Endpoint Description |
|---|---|
| 3. To evaluate the efficacy of ARCT-154 and placebo for the prevention of virologically confirmed COVID-19 at any time after first vaccination regardless of baseline status for evidence of prior SARS-CoV-2 infection | Efficacy may be evaluated in all participants in the Phase 3b mITT that have received any dose of study vaccine in the first vaccination series, regardless of baseline status for evidence of prior SARS-CoV-2 infection. The endpoints are as follows:<br>• The first occurrence of confirmed, protocol-defined (Appendix 2) COVID-19 with onset at any time after the first study vaccination and up to Day 92. |
| 4. To evaluate the efficacy of ARCT-154 and placebo for the prevention of virologically confirmed SARS-CoV-2 by specific strain | Efficacy will be evaluated in participants in Phase 3b mITT. The endpoint to be evaluated is as follows:<br>• The first occurrence of confirmed, protocol-defined (Appendix 2) COVID-19 for all participants infected with the same strain of SARS-CoV-2, with onset between Day 36 and Day 92 inclusive.<br>A second nasal swab specimen collected contemporaneously in participants under evaluation for COVID-19 will be retained for possible future testing to identify the SARS-CoV-2 strain detected in the COVID-19 case. |
| 5. To evaluate the efficacy of ARCT-154 and placebo for the prevention of virologically confirmed severe SARS-CoV-2 by specific strain | Efficacy will be evaluated in participants in Phase 3b mITT. The endpoint to be evaluated is as follows:<br>• The first occurrence of confirmed, protocol-defined (Appendix 2) severe COVID-19 for all participants infected with the same strain of SARS-CoV-2, with onset between Day 36 and Day 92 inclusive.<br>A second nasal swab specimen collected contemporaneously in participants under evaluation for COVID-19 will be retained for possible future testing to identify the SARS-CoV-2 strain detected in the COVID-19 case. |
| 6. To evaluate an immune correlate associated with reduced risk of COVID-19 | • Blood samples drawn at Baseline and Day 57 in all participants will be held for potential future testing for evaluation of a correlate of protection. |

## 3.3 Phase 3c Substudy

### 3.3.1 Phase 3c Substudy Primary Objectives and Endpoints

The primary objectives and endpoints for Phase 3c are presented in Table 8. The first approximately 1,500 participants in Phase 3c will be enrolled into Phase 3c-1, the group that will have blood drawn for immunogenicity assessments. Data from 800 participants in this subgroup will be used to establish the noninferiority of immunogenicity of ARCT-154 vaccine as compared to ChAdOx1. Samples from the remaining 700 participants in Phase 3c-1 will be retained for potential future immunological testing, for example against newly emerging variants of concern. Safety evaluations will be based on all participants in Phase 3c.

**Table 8 Phase 3c Substudy Primary Objectives and Endpoints**

| Phase 3c Primary Objectives | Endpoint Description |
|---|---|
| 1. To assess the safety and reactogenicity of ARCT-154 and ChAdOx1 | Safety will be evaluated in all participants in the Phase 3c SAS and will be summarized for each vaccination as number and percentage of participants with:<br>• Any unsolicited AE starting within 28 days after each study vaccine administration, summarized by severity and relationship to study vaccine.<br>• Any medically attended adverse event (MAAE), serious adverse event (SAE), or AE leading to discontinuation/withdrawal through Final Visit/Early Termination (ET) summarized by relationship to study vaccine.<br>Reactogenicity will be evaluated in participants in the Phase 3c RAS and will be summarized following vaccinations on Day 1 and Day 29 only as the number and percentage of participants with:<br>• Any solicited local or systemic AE starting within 7 days after each study vaccine administration by toxicity grade. |
| 2. To evaluate noninferiority of neutralizing antibody geometric mean concentration for ARCT-154 versus ChAdOx1 at Day 57 | Neutralizing antibody (NAb) responses by pseudovirus microneutralization test (MNT) will be evaluated Day 57 for assessment of geometric mean concentration in all participants in the Phase 3c-1 IAS.<br>• The endpoint is the geometric mean ratio of the surrogate virus neutralization test for ARCT-154 and ChAdOx1 at Day 57<br>Data will be summarized according to use of international reference standards, if available.<br>Noninferiority will be concluded if the lower bound of the 95% confidence interval for the geometric mean ratio of ARCT-154 / ChAdOx1 >0.67. |

### 3.3.2 Phase 3c Substudy Secondary Objectives and Endpoints

The secondary objectives and endpoints for Phase 3c are presented in Table 9.

**Table 9 Phase 3c Substudy Secondary Objectives and Endpoints**

| Phase 3c Secondary Objectives | Endpoint Description |
|---|---|
| 1. To evaluate noninferiority of IgG binding antibody geometric mean concentration for ARCT-154 versus ChAdOx1 at Day 57 | IgG antibody binding the full-length SARS-CoV-2 spike protein (binding antibody [BAb]) responses will be evaluated Day 57 for assessment of geometric mean concentration in all participants in the Phase 3c-1 IAS.<br>• The endpoint is the geometric mean ratio of BAb for ARCT- 154 and ChAdOx1 at Day 57<br>Data will be summarized according to use of international reference standards, if available<br>Noninferiority will be concluded if the lower bound of the 95% confidence interval for the geometric mean ratio of ARCT-154 / ChAdOx1 >0.67 |

**Table 9 Phase 3c Substudy Secondary Objectives and Endpoints**

| Phase 3c Secondary Objectives | Endpoint Description |
|---|---|
| 2. To evaluate noninferiority of neutralizing antibody seroconversion rate for ARCT-154 versus ChAdOx1 at Day 57 | Neutralizing antibody (NAb) responses by pseudovirus microneutralization test (MNT) will be evaluated at Day 1 (baseline) and Day 57 for assessment of seroconversion in all participants in the Phase 3c-1 IAS.<br>• The endpoint is seroconversion rate (defined as: 4-fold increase in titer from baseline) for ARCT-154 and ChAdOx1 at Day 57<br>Data will be summarized according to use of international reference standards, if available.<br>Noninferiority will be concluded if the lower bound of the 95% confidence interval of the difference between neutralizing antibody seroconversion rates (ARCT-154 group minus ChAdOx1 nCoV-1 group) excludes the noninferiority margin of -10%. |
| 3. To evaluate noninferiority of IgG binding antibody seroconversion rate for ARCT-154 versus ChAdOx1 at Day 57 | IgG antibody binding the full-length SARS-CoV-2 spike protein (BAb) responses will be evaluated at Day 1 (baseline) and Day 57 for assessment of seroconversion in all participants in the Phase 3c-1 IAS.<br>• The endpoint is seroconversion rate (defined as: 4-fold increase in titer from baseline) for ARCT-154 and ChAdOx1 at Day 57<br>Data will be summarized according to use of international reference standards, if available.<br>Noninferiority will be concluded if the lower bound of the 95% confidence interval of the difference between binding antibody seroconversion rates (ARCT-154 group minus ChAdOx1 nCoV-1 group) excludes the noninferiority margin of -10%. |
| 4. To evaluate superiority of neutralizing antibody geometric mean concentration for ARCT-154 versus ChAdOx1 at Day 57 | Neutralizing antibody (NAb) responses by pseudovirus microneutralization test (MNT) will be evaluated at Day 57 for assessment of geometric mean concentration in all participants in the Phase 3c-1 IAS.<br>• The endpoint is the geometric mean ratio of the MNT for ARCT- 154 and ChAdOx1 at Day 57<br>Data will be summarized according to use of international reference standards, if available.<br>Superiority will be concluded if the 95% confidence interval for the geometric mean ratio of ARCT-154 / ChAdOx1 >1.0 |
| 5. To evaluate superiority of IgG binding antibody geometric mean concentration for ARCT-154 versus ChAdOx1 at Day 57 | IgG antibody binding the full-length SARS-CoV-2 spike protein (BAb) responses will be evaluated at Day 57 for assessment of geometric mean concentration in all participants in the Phase 3c-1 IAS.<br>• The endpoint is the geometric mean ratio of BAb for ARCT-154 and ChAdOx1 at Day 57<br>Data will be summarized according to use of international reference standards, if available.<br>Superiority will be concluded if the 95% confidence interval for the geometric mean ratio of ARCT-154 / ChAdOx1 >1.0. |

**Table 9 Phase 3c Substudy Secondary Objectives and Endpoints**

| Phase 3c Secondary Objectives | Endpoint Description |
|---|---|
| 6. To assess the neutralizing and IgG binding antibody immune responses for ARCT-154 and ChAdOx1 over time | Neutralizing antibody (NAb) by pseudovirus microneutralization test (MNT) and IgG antibody binding the full-length SARS-CoV-2 spike protein (BAb) responses will be evaluated at: Day 1 (baseline), Day 29, Day 57 and Day 211 for all participants in the Phase 3c-1 IAS. Assessments for both BAb and NAb will include:<br>• Antibody level: GMC<br>• Increase in antibody levels from baseline (GMFR or other measure, as appropriate for the assay concerned)<br>• Geometric mean ratio (GMR)<br>• Proportion of participants in each study vaccine group with seroconversion at each timepoint.<br>Samples will also be collected at Day 394 and may be evaluated in the same assays.<br>Note: NAb and BAb responses that have been evaluated as other primary and secondary endpoints will be included in this endpoint for completeness of data presentation across time points. Full details will be provided in the statistical analysis plan. |

### 3.3.3 Phase 3c Substudy Exploratory Objectives and Endpoints

The exploratory objectives and endpoints for Phase 3c are presented in Table 10.

**Table 10 Phase 3c Substudy Exploratory Objectives and Endpoints**

| Phase 3c Exploratory Objectives | Endpoint Description |
|---|---|
| 1. To evaluate the efficacy of ARCT-154 and ChAdOx1 nCoV-1 for the prevention of virologically confirmed COVID-19 | Efficacy will be evaluated in all participants in the Phase 3c mITT. The endpoint to be evaluated is as follows:<br>• The first occurrence of confirmed, protocol-defined (Appendix 2) COVID-19 with onset after Day 35.<br>Efficacy may also be evaluated in participants that have received any dose of study vaccine in the first vaccination series, with no evidence of infection prior to vaccination. The endpoint is as follows:<br>• The first occurrence of confirmed, protocol-defined (Appendix 2) COVID-19 with onset at any time after the first study vaccination. |
| 2. To evaluate the efficacy of ARCT-154 and ChAdOx1 nCoV-1 for the prevention of virologically confirmed severe COVID-19 | Efficacy will be evaluated in all participants in the Phase 3c mITT. The endpoint to be evaluated is as follows:<br>• The first occurrence of confirmed, protocol-defined (Appendix 2) severe COVID-19 with onset after Day 35.<br>Efficacy may also be evaluated in participants that have received any dose of study vaccine in the first vaccination series, with no evidence of infection prior to vaccination. The endpoint is as follows: |

**Table 10 Phase 3c Substudy Exploratory Objectives and Endpoints**

| Phase 3c Exploratory Objectives | Endpoint Description |
|---|---|
| | • The first occurrence of confirmed, protocol-defined (Appendix 2) severe COVID-19 with onset at any time after the first study vaccination. |
| 3. To evaluate the efficacy of ARCT-154 and ChAdOx1 nCoV-1 for the prevention of death due to COVID-19 | Efficacy will be evaluated in all participants in the Phase 3c mITT. The endpoint to be evaluated is as follows:<br>• The occurrence of death attributed to COVID-19 disease occurring after Day 35.<br>Efficacy may also be evaluated in participants that have received any dose of study vaccine in the first vaccination series, with no evidence of infection prior to vaccination. The endpoint to be evaluated is as follows:<br>• The occurrence of death attributed to COVID-19 occurring at any time after the first study vaccination. |
| 4. To evaluate the efficacy of ARCT-154 and ChAdOx1 for the prevention of virologically confirmed COVID-19 regardless of baseline status for evidence of prior SARS-CoV-2 infection | Efficacy will be evaluated in all participants in the Phase 3c mITT regardless of baseline status for evidence of prior SARS-CoV-2 infection. The endpoint to be evaluated is as follows:<br>• The first occurrence of confirmed, protocol-defined (Appendix 2) COVID-19 with onset after Day 35.<br>Efficacy may also be evaluated in participants that have received any dose of study vaccine in the first vaccination series. The endpoint is as follows:<br>• The first occurrence of confirmed, protocol-defined (Appendix 2) COVID-19 with onset at any time after the first study vaccination. |
| 5. To evaluate the neutralizing antibody responses of ARCT-154 and ChAdOx1 using a surrogate virus neutralization test | Neutralizing antibody (NAb) responses by surrogate virus neutralization test (sVNT) may be evaluated at individual time points. The following endpoints may be assessed:<br>• Geometric mean antibody concentration (GMC)<br>• Increase in antibody levels from baseline (GMFR)<br>• Proportion of participants in each study vaccine group with seroconversion<br>Data will be summarized according to use of international reference standards, if available. |
| 6. To evaluate the neutralizing antibody responses of ARCT-154 and ChAdOx1 to SARS-CoV-2 variants of concern/variants of interest | Neutralizing antibody responses to SARS-CoV-2 variants of concern may be evaluated at individual time points in participants in the Phase 3c-1 IAS using a pseudovirus microneutralization test (MNT) and/or surrogate virus neutralization test (sVNT) for assessment of:<br>• Antibody level: GMC<br>• Increase in antibody levels from baseline (GMFR or other measure, as appropriate for the assay concerned)<br>• Geometric mean ratio (GMR)<br>• Proportion of participants in each study vaccine group with seroconversion at each timepoint.<br>The following assays may be used:<br>• PRNT50 |

**Table 10 Phase 3c Substudy Exploratory Objectives and Endpoints**

| Phase 3c Exploratory Objectives | Endpoint Description |
|---|---|
| | • sVNT<br>• MNT |

## 3.4 Pooled Objectives and Endpoints

### 3.4.1 Pooled Primary Objectives and Endpoints

The pooled primary objectives and endpoints for the study are presented in Table 11. These will be evaluated as sensitivity analyses to efficacy and safety endpoints being evaluated in Phase 3b.

**Table 11 Pooled Primary Objectives and Endpoints**

| Pooled Primary Objectives | Endpoint Description |
|---|---|
| 1. To assess the safety and reactogenicity of ARCT-154 and comparator vaccines (placebo, ChAdOx1 nCoV-1) | Safety data will be evaluated in the Pooled SAS and will be summarized for each vaccination as number and percentage of participants with:<br>• Any unsolicited AE starting within 28 days after each study vaccine administration, summarized by severity and relationship to study vaccine.<br>• Any medically attended adverse event (MAAE), serious adverse event (SAE), or AE leading to discontinuation/withdrawal through Final Visit/Early Termination (ET) summarized by relationship to study vaccine.<br>Reactogenicity will be evaluated in the Pooled RAS and will be summarized following vaccinations on Day 1 and Day 29 only as the number and percentage of participants with:<br>• Any solicited local or systemic AE starting within 7 days after each study vaccine administration by toxicity grade. |
| 2. To evaluate the efficacy of ARCT-154 and placebo for the prevention of virologically confirmed COVID-19 | Efficacy will be evaluated in all participants in Pooled mITT. The endpoint to be evaluated is as follows:<br>• The first occurrence of confirmed, protocol-defined (Appendix 2) COVID-19 with onset between Day 36 and Day 92 inclusive. |

### 3.4.2 Pooled Secondary Objectives and Endpoints

The pooled secondary objectives and endpoints for the study are presented in Table 12.

**Table 12 Pooled Secondary Objectives and Endpoints**

| Pooled Secondary Objectives | Endpoint Description |
|---|---|
| 1. To evaluate the efficacy of ARCT-154 and placebo for the prevention of virologically confirmed severe COVID-19 | Efficacy will be evaluated in all participants in the Pooled mITT. The endpoint to be evaluated is as follows:<br>• The first occurrence of confirmed, protocol-defined (Appendix 2) severe COVID-19 with onset between Day 36 and Day 92 inclusive.<br>• Severity as scored using the FDA criteria will be the principal analysis. Severity using the WHO criteria will be performed as a sensitivity analysis |
| 2. To evaluate the efficacy of ARCT154 and placebo for the prevention of virologically confirmed COVID-19 at any time after first vaccination | Efficacy will be evaluated in participants that have received any dose of study vaccine in the first vaccination series, with no evidence of infection prior to vaccination. The endpoint is as follows:<br>The first occurrence of confirmed, protocol-defined (Appendix 2) COVID-19 with onset at any time after the first study vaccination and up to Day 92 inclusive. |
| 3. To evaluate the efficacy of ARCT-154 and placebo for the prevention of death due to COVID-19 | Efficacy will be evaluated in all participants in the Pooled mITT The endpoint is as follows:<br>• The occurrence of death attributed to COVID-19 with onset between Day 36 and Day 92 inclusive. |
| 4. To evaluate the efficacy of ARCT-154 and placebo for the prevention of virologically confirmed COVID-19 regardless of baseline status for evidence of prior SARS-CoV-2 infection | Efficacy will be evaluated in all participants in Pooled mITT regardless of baseline status for evidence of prior SARS-CoV-2 infection. The endpoint to be evaluated is as follows:<br>• The first occurrence of confirmed, protocol-defined (Appendix 2) COVID-19 with onset between Day 36 and Day 92 inclusive. |

### 3.4.3 Pooled Exploratory Objectives and Endpoints

The Pooled exploratory objectives and endpoints for the study are presented in Table 13.

**Table 13 Pooled Exploratory Objectives and Endpoints**

| Pooled Exploratory Objectives of Study | Endpoint Description |
|---|---|
| 1. To evaluate the efficacy of ARCT-154 and placebo for the prevention of virologically confirmed severe COVID-19 at any time after first vaccination | Efficacy may be evaluated in all participants in the Pooled mITT that have received any dose of study vaccine in the first vaccination series, with no evidence of infection prior to vaccination. The endpoint is as follows:<br>• The first occurrence of confirmed, protocol-defined (Appendix 2) severe COVID-19 with onset at any time after the first study vaccination and up to Day 92. |

### Table 13 Pooled Exploratory Objectives and Endpoints

| Pooled Exploratory Objectives of Study | Endpoint Description |
|---|---|
| 2. To evaluate the efficacy of ARCT-154 and placebo for the prevention of death due to COVID-19 at any time after first vaccination | Efficacy may be evaluated in all participants in the Pooled mITT that have received any dose of study vaccine in the first vaccination series. The endpoint is as follows:<br>• The occurrence of death attributed to COVID-19 occurring at any time after the first study vaccination and up to Day 92. |
| 3. To evaluate the efficacy of ARCT-154 and placebo for the prevention of virologically confirmed COVID-19 at any time after first vaccination regardless of baseline status for evidence of prior SARS-CoV-2 infection | Efficacy may be evaluated in all participants in the Pooled mITT that have received any dose of study vaccine in the first vaccination series, regardless of baseline status for evidence of prior SARS-CoV-2 infection. The endpoints are as follows:<br>• The first occurrence of confirmed, protocol-defined (Appendix 2) COVID-19 with onset at any time after the first study vaccination and up to Day 92. |
| 4. To evaluate the efficacy of ARCT-154 and placebo for the prevention of virologically confirmed SARS-CoV-2 by specific strain | Efficacy will be evaluated in participants in the Pooled mITT. The endpoint to be evaluated is as follows:<br>• The first occurrence of confirmed, protocol-defined (Appendix 2) COVID-19 for all participants infected with the same strain of SARS-CoV-2, with onset between Day 36 and Day 92 inclusive.<br>A second nasal swab specimen collected contemporaneously in participants under evaluation for COVID-19 will be retained for possible future testing to identify the SARS-CoV-2 strain detected in the COVID-19 case. |
| 5. To evaluate the efficacy of ARCT-154 and placebo for the prevention of virologically confirmed severe SARS-CoV-2 by specific strain | Efficacy will be evaluated in participants in Pooled mITT. The endpoint to be evaluated is as follows:<br>• The first occurrence of confirmed, protocol-defined (Appendix 2) severe COVID-19 for all participants infected with the same strain of SARS-CoV-2, with onset between Day 36 and Day 92 inclusive.<br>A second nasal swab specimen collected contemporaneously in participants under evaluation for COVID-19 will be retained for possible future testing to identify the SARS-CoV-2 strain detected in the COVID-19 case. |
| 6. To evaluate for an immune correlate associated with reduced risk of COVID-19 | Blood samples drawn at Baseline and Day 57 in all participants will be held for potential future testing for evaluation of a correlate of protection. |

# 4 INVESTIGATIONAL PLAN

Before any study procedures are performed, all potential participants will sign an informed consent form (ICF). Additional procedural details related to the ICF are provided in Section 10.3.

## 4.1 Number of Participants

Enrollment of approximately 19,400 participants across all phases of the study is planned, as follows:

- Phase 1: 100 (75 ARCT-154:25 placebo per study group)
- Phase 2: approximately 300 (225 ARCT-154:75 placebo per study group)
- Phase 3a: approximately 600 (450 ARCT-154:150 placebo per study group)
- Phase 3b: approximately 16,000 (8,000 per study group)
- Phase 3c: approximately 2,400 (~1,200 per study group)
    - The first approximately 1,500 participants will be enrolled into Phase 3c-1, the group that will have blood drawn for immunogenicity assessments. Data from 800 participants in this subgroup will be used to establish the noninferiority of immunogenicity of ARCT-154 vaccine as compared to ChAdOx1
    - The remaining ~900 participants will form Phase 3c-2

## 4.2 Number of Sites

Approximately 20 sites in Vietnam will participate.

## 4.3 Duration of Participation

The expected duration of participation for an individual participant (including the Screening period) is approximately 14 months, 12 months after completion of the initial two-dose vaccination series.

## 4.4 Study Design

This is a Phase 1/2/3, randomized, controlled, observer-blind study designed to evaluate the safety, immunogenicity and efficacy of ARCT-154 in adult participants to be enrolled in Vietnam. This study design has been updated to include a cohort (Phase 3c) to evaluate the immunogenicity, safety, and efficacy of ARCT-154 versus a comparator COVID-19 vaccine.

The study is divided into five parts: Phase 1, Phase 2, Phase 3a, Phase 3b and Phase 3c.

Participants in Phase 1 and 3b will be randomly assigned (Section 4.5.2) to a study group that will receive up to 2 vaccination series (4 doses of study vaccine total). Each vaccination series comprises two vaccinations at 28-day intervals: an initial vaccination series with vaccinations on Day 1 and Day 29 and an additional two-dose vaccination series with the opposite vaccine at the 6-month timepoint on Day 92 and 120, which is at the 2-months after the second dose (referred to as the "Switchover" vaccination series throughout the protocol). Each participant in Phase 1 and 3b is planned to receive a two-dose vaccination series of ARCT-154 at a dose of 5 µg and a

two-dose vaccination series of placebo (saline). Therefore, the study group participants that initially received two doses of ARCT-154 will receive two doses of placebo on Switchover and study group participants that initially received placebo will receive two doses of ARCT-154 5 µg on Switchover (Figure 1).

Participants in Phase 2 and 3a will be randomly assigned to a study group that will receive two vaccinations with either ARCT-154 or placebo at a 28-day interval with vaccinations on Day 1 and Day 29. With protocol version 8.0, participants that received ARCT-154 in the initial two-dose vaccination series will be further randomized to receive either ARCT-154 or placebo (in a 3:1 ratio) at Day 92 (referred to as "Further Study Vaccine" throughout the protocol) followed by placebo at Day 120. Participants that received placebo in the initial vaccination series will undergo Switchover to receive ARCT-154 at Day 92 and Day 120.

Participants in Phase 3c will be randomized to receive either ARCT-154 or Astra Zeneca COVID-19 vaccine (ChAdOx1 nCoV-19; referred to as ChAdOx1from here on) which has received authorization for use in Vietnam. Participants in Phase 3c will receive a 2-dose priming vaccination series of either ARCT-154 5µg or ChAdOx1, with vaccinations being administered on Day 1 and Day 29. Because all participants in Phase 3c will receive an active vaccine, no switchover will occur for these participants.

Phase 1 is a sentinel cohort for assessment of safety. 100 healthy participants will be randomly assigned 3:1 to receive ARCT-154 or placebo (75:25) for the initial series of vaccinations. The Safety Review Committee (SRC) and Vietnam Ministry of Health (MOH) will independently review available blinded safety data for Phase 1 participants through at least 7 days post-second vaccination (Day 36) and, if study vaccination is judged to be adequately tolerated by SRC and the MOH, the study will initiate simultaneous enrollment of both Phase 2 and Phase 3a.

In Phase 2, which expands the safety and immunogenicity characterization of the study vaccine in a larger group of participants, 300 participants will be randomly assigned 3:1 to receive ARCT-154 or placebo (225:75) for the initial series of vaccinations.

In Phase 3a, which expands the enrollment further to include 600 participants, who will be randomly assigned 3:1 to receive ARCT-154 or placebo (450:150) for the initial series of vaccinations. After completion of enrollment of Phases 2 and 3a, the SRC and MOH will independently review available blinded safety data through at least 7 days (Day 8) post-first vaccination and, if study vaccination is judged to be adequately tolerated by SRC and the MOH, the study will initiate enrollment of Phase 3b. The second dose of study vaccine will not be administered to participants in Phase 3b until the SRC has reviewed the Phase 2 and Phase 3a safety data through 7 days post-second vaccination (Day 36) and judged that study vaccine is adequately tolerated. Data from participants in Phase 1, Phase 2, and Phase 3a will be pooled for a primary analysis of immunogenicity at Day 57. Data from this analysis will be submitted to the Vietnamese Ministry of Health for evaluation for potential emergency use authorization (EUA). Data from Phase 2 and 3a will also be pooled to allow comparison of immunogenicity following the 3$^{rd}$ dose (immune responses at Day 120 with that after the 2$^{nd}$ dose (immune responses at Day 57).

In Phase 3b, approximately 16,000 participants will be randomly assigned 1:1 to receive ARCT-154 or placebo for the initial series of vaccinations. Phase 3b constitutes the Exploratory

Efficacy Subgroup. Data from this subgroup will support regulatory filings along with immunogenicity data from Phase 1/2/3a and 3c. Participants in this Phase 3b portion of the study will contribute substantially to the safety analyses of ARCT-154.

Phase 3c will enroll approximately 2,400 participants randomized 1:1 to receive ARCT-154 or ChAdOx1. A subset of these enrolled participants (the first 1,500 participants enrolled in Phase 3c) will form an Immunogenicity Noninferiority Subgroup (Phase 3c-1); the remaining ~900 participants will form Phase 3c-2. Day 57 data from 800 participants in this Subgroup will be used to establish the noninferiority of immunogenicity of ARCT-154 vaccine as compared to ChAdOx1, a licensed comparator vaccine. These data, along with an analysis of safety from participants exposed to ARCT-154 and followed for a median period of 2 months (60 days) may be submitted to health authorities for evaluation for a Marketing Authorization or EUA dependent on jurisdiction.

Phase 1 participants must be <60 years of age. Phase 2, 3a, 3b and 3c participants will include elderly (≥60 years) participants as well. Phase 3b and Phase 3c will enrich for participants at higher risk for COVID-19 by attempting to enroll individuals considered at high risk due to their workplace environment or living conditions.

For Phases 2, 3a, 3b and 3c, after a 10-day Screening period, prior to randomization, participants will be stratified by age (< 60 or ≥ 60 years of age) and for participants < 60 years of age by risk of severe COVID-19. Participants will be defined as "at risk" if they are 60 years of age or older OR have medical history described as putting the individual at risk or possibly at risk of severe coronavirus disease 2019 (COVID-19) (Appendix 5). As the eligibility criteria for Phase 1 exclude participants ≥ 60 years of age or otherwise at risk for severe COVID-19, stratification will not occur for Phase 1.

Participants in all study phases will undergo informed consent and screening procedures prior to enrollment. After enrollment, participants will be randomized to groups. Study vaccines will be given in a supervised medical setting. All participants will be evaluated for reactogenicity (solicited adverse events) and reporting of unsolicited adverse events and concomitant medications through a series of clinic visits and phone calls. Depending on the study phase, blood sampling for immunogenicity and testing for COVID-19 may be performed. See Appendix 1 for an overview of study visits by study phase.

For participants in Phase 1/2/3a/3b, at the Day 92 visit and prior to receipt of further study vaccine, participants will be evaluated by the Investigator according to Further Study Vaccine eligibility criteria (Section 5.1.5). Any participant who does not meet these criteria will not receive further vaccinations with study vaccine and will be asked to continue in the study through Final Visit (Day 394).

For participants that receive further doses of Study Vaccine at Day 92 and Day 120, this will occur in a blinded fashion in that participants and blinded site and Sponsor personnel will remain blinded to treatment assignment until End of Study (EOS).

Participants who choose to receive vaccination with off-study COVID-19 vaccine will be encouraged to remain in the study, but those that choose to leave the study to pursue alternate vaccination will not be unblinded to study treatment unless there is an unexpected post study SAE attributed as related to study vaccine by the study investigator.

Data will be collected through a combination of clinic visits and use of electronic diaries (and paper diaries in circumstances where electronic diaries are not feasible) to capture solicited adverse events and to prompt for symptoms of COVID-19 and risk of SARS-CoV-2 exposure, other adverse events, and concomitant medications. All participants enrolled in Phase 1 and Phase 2 must have access to the eDiary to facilitate rapid review of safety data by the SRC.

Exposure to SARS-CoV-2 is defined as a participant who has had close contact (within 6 feet [2 meters] to a person with virologically confirmed COVID-19 during the active transmission period.

The active transmission period is:

1) for a symptomatic COVID-19 case, 3 days before the first symptom, or
2) for an asymptomatic COVID-19 case, from 14 days prior to the positive COVID-19 test result.

The close contact definition applies regardless of whether the person with COVID-19 or the contact was wearing a mask or whether the contact was wearing respiratory personal protective equipment. Individuals with suspected COVID-19 symptoms and/or exposures will be evaluated, ideally within 72 hours to determine if there is a potential COVID-19 case. Participants may also be seen at unscheduled clinic visits if there are any safety issues that an investigator wishes to evaluate.

Participant data from each of the study phases may be unblinded prior to study completion to support regulatory filings. When these unblindings occur, unblinded data will only be provided to Sponsor or CRO employees not involved in the subsequent conduct of the study; the specific timing of these unblindings is defined in Section 8.9. Individual participants, blinded study site staff and Sponsor team members involved in overseeing the conduct of the study will remain blinded until the end of study for all Phases (Phases 1, 2, and 3a/b/c). Sponsor staff and other persons that will become unblinded at the various data analysis time points will be specified prior to unblinding occurring.

Phase 1, 2, 3a and 3c-1: participants will be consented to have blood collected more frequently for assessment of SARS-CoV-2 neutralizing antibody responses and binding antibody responses to the SARS-CoV-2 spike protein. The study will also include collection of blood samples in all participants for potential future exploratory testing of potential correlates of protection and vaccine responses to SARS-CoV-2 variants.

An independent Data and Safety Monitoring Board (DSMB) will perform ongoing review of blinded and unblinded data, including both safety and confirmed cases of COVID-19 at scheduled data review meetings. At each meeting, the DSMB will review the available data and make recommendations to the Sponsor to continue, modify, or discontinue study enrollment (if the study is enrolling). In addition to the ongoing review of safety data, the DSMB will also review available severe COVID-19 case data to determine the risk of Vaccine-Associated Enhanced Respiratory Disease (VAERD). The DSMB will be constituted prior to commencement of Phase 3b.

An independent blinded endpoint adjudication committee will adjudicate all suspected COVID-19 cases to determine if they meet the primary endpoint requirements (Appendix 2).

## 4.5 Study Assessments and Procedures

A schedule of assessments is provided in Appendix 1.

### 4.5.1 Informed Consent

"Informed consent" is the voluntary agreement of an individual to participate in research. Consent must be given with free will of choice and without undue inducement. The individual must have sufficient knowledge and understanding of the nature of the proposed research, the anticipated risks and potential benefits, and the requirements of the research to be able to make an informed decision.

The process of obtaining informed consent must be documented in the participants source documents in addition to maintaining a copy of the signed and dated informed consent. Additional specifics regarding the informed consent process are located in Section 10.3.

If a participant or participant's legal guardian(s) is/are unable to read, an impartial witness should be present during the entire informed consent. An impartial witness is defined as a person who is independent from trial conduct, who cannot be unfairly influenced by those involved with the trial, who attends the informed consent process if the participant or the participant's legally acceptable representative cannot read, and who reads the informed consent/assent form and any other written information supplied to the participant. After the written ICF/assent and any other written information to be provided to participants is read and explained to the participant or participant's legal guardian(s), after the participant or participant's legal guardian(s) has verbally consented to the participant's participation in the trial, and after the participant, if capable of doing so, has signed and personally dated the informed consent/assent form, the witness should sign and personally date the consent/assent form. By signing the consent form, the witness attests that the information in the consent form and any other written information was accurately explained to, and apparently understood by, the participant or participant's legal guardian(s) and that informed consent was freely given by the participant or participant's legal guardian(s).

### 4.5.2 Randomization and Enrollment

Phase 1, 2 and 3a participants will be randomly assigned 3:1 (ARCT-154:placebo), while Phase 3b participants will be randomly assigned 1:1 (ARCT-154:placebo).

- Participants in Phase 1 and 3b will receive two doses of one type of study vaccine on Day 1 and 29 (ARCT-154 or placebo) and then two doses of the opposite vaccine (placebo or ARCT-154) on Day 92 and 120 (referred to as 'Switchover').
- Participants in Phase 2/3a who received ARCT-154 in the initial two-dose vaccination series will be further randomized to receive either ARCT-154 or placebo (in a 3:1 ratio) at Day 92 followed by placebo at Day 120. Participants that received placebo in the initial vaccination series will undergo Switchover to receive ARCT-154 at Day 92 and Day 120.

Phase 3c participants will be randomized 1:1 to receive ARCT-154 or ChAdOx1; no Switchover will occur for Phase 3c participants as all participants in this cohort receive active vaccine.

For Phases 2, 3a, 3b and 3c, prior to randomization, participants will be stratified by the following 3 stratification factors:

- Age < 60 at high risk of severe COVID-19
- Age < 60 not at high risk of severe COVID-19
- Age ≥ 60 years of age (considered at risk of severe COVID-19 by default)

  Participants will be defined as "at risk" if they are 60 years of age or older OR have medical history described as putting an individual at risk or possibly at risk of severe coronavirus disease 2019 (COVID-19) (Appendix 5).

  As the eligibility criteria for Phase 1 exclude participants ≥ 60 years of age or otherwise at risk for severe COVID-19, stratification will not occur for Phase 1.

At screening, a Participant Identifier will be assigned by the site via the Interactive Response Technology (IRT) system. After the participant has completed screening and is determined to be eligible for study participation, the participant will be randomized by the site via the IRT system. At randomization, the participant will automatically be assigned to a study group. This participant's randomization must be documented in the Screening and Enrollment Log.

Randomization in IRT can be performed by the site via the internet by the investigator or site personnel (as delegated by the investigator) who are permitted by institutional policy and trained to perform this task.

### 4.5.3 Visits

The number of visits and calls planned for study participants is linked to the study phase into which a participant is enrolled and Switchover/Further Study Vaccine status.

Follow-up visits and Unscheduled visits may be performed by telemedicine visits in combination with home health nursing visits or in a hospital setting if warranted by clinical circumstances (eg, COVID-19 lockdown in the vicinity) and if permitted by local regulations.

Phase 1 and 3b Participants Eligible for Switchover:

- Phase 1 participants eligible for Switchover have:
  - 9 clinic visits, including Screening, Dose 1 (Day 1), Follow-up Visit (Day 8), Dose 2 (Day 29), Follow-up Visit (Day 36), Follow-up Visit (Day 57), Switchover Dose 1 (Day 92), Switchover Dose 2 (Day 120), Final Visit (Day 394)/Early Termination (ET) visit (if applicable).
  - 25 telephone calls will be made, including: weekly on Days 15, 22, 43, 50, 64, 71, 78, 85, 99, 106, 113, 127, 134, 141, 148, 155, 162, 169, 176 and then monthly calls on Days 210, 240, 270, 300, 330 and 360.
- Phase 3b participants eligible for Switchover have:
  - 7 clinic visits including Screening, Dose 1 (Day 1), Dose 2 (Day 29), Follow-up Visit (Day 57), Switchover Dose 1 (Day 92), Switchover Dose 2 (Day 120), Final Visit (Day 394)/Early Termination (ET) visit (if applicable).

- 27 telephone calls will be made, including: weekly calls on Days 8, 15, 22, 36, 43, 50, 64, 71, 78, 85, 99, 106, 113, 127, 134, 141, 148, 155, 162, 169, 176 and then monthly calls on Days 210, 240, 270, 300, 330 and 360.

Participants Ineligible for Switchover:

- Phase 1 participants ineligible for Switchover have:
  - 8 clinic visits including Screening, Dose 1 (Day 1), Follow-up Visit (Day 8), Dose 2 (Day 29), Follow-up Visit (Day 36), Follow-up Visit (Day 57), Day 92 (Switchover ineligible), and Final Visit (Day 394)/ET visit (if applicable).
  - 17 telephone calls will be made, including: weekly calls on Days 15, 22, 43, 50, 64, 71, 78, 85, and then monthly calls on Days 120, 150, 180, 210, 240, 270, 300, 330 and 360.
- Phase 3b participants ineligible for Switchover have:
  - 6 clinic visits including Screening, Dose 1 (Day 1), Dose 2 (Day 29), Follow-up Visit (Day 57), Day 92 (Switchover ineligible), and Final Visit (Day 394)/ET visit (if applicable).
  - 19 telephone calls will be made, including: weekly calls on Days 8, 15, 22, 36, 43, 50, 64, 71, 78, 85, and then monthly calls on Days 120, 150, 180, 210, 240, 270, 300, 330 and 360.

Phase 2 and 3a Participants Eligible for additional study vaccine at Day 92 have:

- 7 clinic visits including Screening, Dose 1 (Day 1), Dose 2 (Day 29), Follow-up Visit (Day 57), Further Dose 1 (Day 92), Further Dose 2 (Day 120), Final Visit (Day 394)/Early Termination (ET) visit (if applicable).
- 27 telephone calls will be made, including: weekly calls on Days 8, 15, 22, 36, 43, 50, 64, 71, 78, 85, 99, 106, 113, 127, 134, 141, 148, 155, 162, 169, 176 and then monthly on Days 210, 240, 270, 300, 330 and 360.

Phase 2 and 3a Participants NOT Eligible for additional study vaccine at Day 92 have:

- 6 clinic visits including Screening, Dose 1 (Day 1), Dose 2 (Day 29), Follow-up Visit (Day 57), Day 92 (further study vaccine ineligible), and Final Visit (Day 394)/ET visit (if applicable).
- 19 telephone calls will be made, including: weekly calls on Days 8, 15, 22, 36, 43, 50, 64, 71, 78, 85, and then monthly calls on Days 120, 150, 180, 210, 240, 270, 300, 330 and 360.

Participants in Cohort 3c will have the following visit/telephone call schedule. In addition, participants may have an Early Termination (ET) Visit when relevant, as well as Unscheduled Visits.

- Phase 3c-1 participants:
  - 6 clinic visits including Screening, Dose 1 (Day 1), Dose 2 (Day 29), Follow-up Visits (Day 57 and Day 211), and Final Visit (Day 394)/ET visit (if applicable).
  - 37 telephone calls will be made, including: weekly calls on Days 8, 15, 22, 36, 43, 50, 64, 71, 78, 85, 92, 99, 106, 113, 120, 127, 134, 141, 148, 155, 162, 169, 176, 183, 190, 197, 204, 218, 225, 232, 239, 246, 253, 260 then monthly calls on Days 290, 320 and 350.
- Phase 3c-2 participants:
  - 5 clinic visits including Screening, Dose 1 (Day 1), Dose 2 (Day 29), Follow-up Visits (Day 57), and Final Visit (Day 394)/ET visit (if applicable).
  - 38 telephone calls will be made, including: weekly calls on Days 8, 15, 22, 36, 43, 50, 64, 71, 78, 85, 92, 99, 106, 113, 120, 127, 134, 141, 148, 155, 162, 169, 176, 183, 190, 197, 204, 211, 218, 225, 232, 239, 246, 253, 260 and then monthly calls on Days 290, 320 and 350. In addition, participants in all cohorts may have an Early Termination (ET) Visit when relevant, as well as Unscheduled Visits.

#### 4.5.3.1 Screening Visit (Up to 10 days prior to Day 1)

At Screening, individuals willing to participate in the study will undergo informed consent followed by an interview to review medical history, current symptoms, COVID-19 exposures, AEs, medications, recent vaccinations, and availability for study procedures; will undergo complete physical examination, pregnancy testing (if the participant is a woman of childbearing potential), vital sign measurements (heart rate, respiratory rate, blood pressure), body temperature measurement, as well as height and weight measurements. Blood sampling may be performed for pregnancy test (if urine testing is not permitted by site policy), or -follicle stimulating hormone (FSH) in women suspected to be menopausal (Appendix 1). Nasal swabs for SARS-CoV-2 RT-PCR and blood sampling for screening will be performed as outlined in Section 4.5.5.3. The information gathered from this interview and the assessments will be evaluated against inclusion/exclusion criteria to determine study eligibility. Prior to randomization, participants will undergo determination of "at risk" status for severe COVID-19 (Appendix 5).

#### 4.5.3.2 Vaccine Dose Administration Visits

At each vaccine administration visit, participants will undergo an interview for AEs, concomitant medications/vaccines, updates to medical history, COVID-19 exposures and/or symptoms: pregnancy testing (if the participant is a woman of childbearing potential), complete physical examination, vital signs, and body temperature measurement prior to and after receipt of study vaccine.

Nasal swabs and blood will be collected as outlined in Section 4.5.5.3.

For participants in Phase 1, additional blood will be collected for safety laboratory assessments as outlined in Section 4.5.5.3.

During all Dose Administration visits (Day 1, Day 29, and (in Cohorts 1, 2, 3a, 3b only) Day 92, Day 120), all participants will be trained in how to measure body temperature, ruler use, what signs or symptoms of COVID-19 might be (Section 4.5.3.7), whom to call at the site and how quickly to call in the event of severe or serious AEs or signs of possible COVID-19. At all later points of contact, participants will be reminded of this training. On Day 1 and Day 29, participants will also be shown how to report AEs in the Diary (electronic or paper); note diaries are not used after the Switchover vaccinations. For participants in Phase 2 and 3a, that are receiving additional doses of study vaccine, further training on Diary use will be performed prior to vaccination on Day 92. No solicited events will be collected via Diary after vaccination on Day 120.

For Phase 2, 3a, 3b and 3c participants who did not use the eDiary, paper diaries for the first injection will be collected at Day 29. For Phase 2 and 3a, participants who did not use the eDiary, paper diaries for the third injection will be collected at Day 120.

The exception to performing these procedures is if the participant is screened and enrolled on the same day (Day 1). In this instance, pregnancy testing, screening and safety laboratory assessments, physical examination, vital signs, body temperature, AEs and concomitant medication collection, and solicitation of potential COVID-19 exposure or symptoms will not be repeated.

After confirmation of eligibility and determination of "at risk" status at Day 1 (for Phases 2, 3a, 3b and 3c), the participant will be regarded as enrolled, and randomization of the participant in the IRT will occur.

Study vaccine will be administered in an observer-blind fashion as described in Section 6.3. The second study vaccination of each series must be at least 28 days after the first study vaccination. For Phase 1 and 3b, the first Switchover vaccination (Day 92) must be at least 56 days (+30 days) after the second vaccination of the initial series. For Phase 2 and 3a, the 3rd vaccination with study vaccine must be at least 56 days (+30 days) after the second vaccination series.

After receipt of study vaccine in the initial series of vaccinations, the participant will remain under observation for at least 3 hours for Phase 1, and 30 minutes for Phase 3b, and until clinically stable. For Switchover vaccinations, participants in Phase 1 and Phase 3b will be observed for at least 15 minutes and until clinically stable. Participants in Phase 2 and 3a will be observed for at least 30 minutes and until clinically stable for vaccinations 1, 2, and 3 (Day 92) and for at least 15 minutes and until clinically stable for vaccination 4 (Day 120). During the post-vaccination observation period, the participant will undergo assessment of vital signs, body temperature, AEs, and concomitant medications prior to discharge from the clinic.

Should a participant experience a severe AE (including but not limited to hypersensitivity or anaphylaxis) immediately following study vaccine administration, the participant should be observed until clinically stable, and the Clinical Research Organization (CRO) Medical Monitor should be notified immediately. Additional evaluation or testing may be performed based on

discussion between the Medical Monitor and the investigator and subject to Medical Monitor approval.

#### 4.5.3.3 Follow-up Visit on Day 8 and 36 (Phase 1 only)

For Phase 1, follow-up visits on Day 8 and 36 must occur at least 7 days after each vaccination. Participants in Phase 1 will undergo the following: an interview for AEs, concomitant medications/vaccines, updates to medical history, COVID-19 exposures and/or symptoms, vital signs, body temperature, reminder to notify the site of unsolicited AEs and COVID-19 exposures and/or symptoms. Physical examination may be performed, if warranted, by the interview.

Blood will be drawn as outlined in Section 4.5.5.3.

#### 4.5.3.4 Follow-up Visit on Day 57

The Follow-up visit on Day 57 must occur at least 28 days after the second vaccination. Participants will undergo the following: an interview for AEs, concomitant medications/vaccines, updates to medical history, COVID-19 exposures and/or symptoms, vital signs, body temperature, reminder to notify the site of unsolicited AEs and COVID-19 exposures and/or symptoms. Physical examination may be performed, if warranted, by the interview.

Blood will be drawn as outlined in Section 4.5.5.3.

For Phase 2, 3a, 3b and 3c participants who did not use the eDiary, paper diaries for the second vaccination will be collected.

#### 4.5.3.5 Follow-up Visit on Day 211 (Phase 3c-1 only)

Participants will undergo the following: an interview for AEs, concomitant medications/vaccines, updates to medical history, COVID-19 exposures and/or symptoms, vital signs, body temperature, reminder to notify the site of unsolicited AEs and COVID-19 exposures and/or symptoms. Physical examination may be performed, if warranted, by the interview.

Blood will be drawn as outlined in Section 4.5.5.3.

#### 4.5.3.6 Weekly and Monthly Study Calls

Telephone calls will be performed weekly (when the participant is not otherwise coming in for a clinic visit) then monthly as outlined in Section 4.5.3.

The schedule of the weekly calls (at 7-day intervals) is based on the study day of the prior study vaccination. For Phases 2, 3a, 3b and 3c, the weekly call at Day 8 and Day 36 must be at least 7 days after the prior study vaccination and for participants receiving vaccinations on Days 92 and 120, the weekly study calls on Day 99 and Day 127 must be at least 7 days after the prior study vaccination in order to ensure adequate recording of solicited events.

Telephone calls will be conducted to evaluate participants for safety (collection of AEs and concomitant medications/vaccines, and inquire about COVID-19 exposure and/or symptoms (prompting an Unscheduled visit, ideally within 72 hours for evaluation), and arrange evaluation in clinic as needed [Section 7.1.1]).

#### 4.5.3.7 Unscheduled Visits

Unscheduled visits should be arranged for the evaluation of reported COVID-19 symptoms, exposure to COVID-19, or for the evaluation of safety issues. If a participant experiences these symptoms, reports a COVID-19 exposure, or is experiencing a severe or serious safety concern, he or she is instructed to contact the site immediately.

Symptoms of COVID-19 include:

- Fever
- Chills
- Cough
- Shortness of breath or difficulty breathing
- Fatigue
- Muscle or body aches
- Headache
- New loss of taste or smell
- Sore throat
- Congestion or runny nose
- Nausea
- Vomiting
- Diarrhea

Some of these symptoms (fever/chills, fatigue, muscle aches, headache, nausea, diarrhoea, vomiting) overlap with solicited event symptoms that may be experienced as a consequence of receiving vaccine. However, based on experience to date, onset of solicited events following ARCT-154 is typically within the first 3 days after vaccination and the symptoms have typically resolved or improved by 7 days after vaccination. Therefore, it is recommended that:

- If any of the following symptoms are observed WITHIN 3 days after vaccination, then clinical judgement should be used to determine if COVID-19 is likely. However, if ANY ONE or more of these symptoms occur BEYOND 3 days after vaccination or if they PERSIST beyond 7 days after vaccination then testing for COVID-19 should be performed
    - Fever or chills
    - Fatigue
    - Muscle or body aches
    - Headache
    - Nausea or vomiting
    - Diarrhea
- Other symptoms, as listed below, are not typical solicited adverse events, so COVID testing should be performed if ANY ONE or more of these symptoms is experienced AT ANY time, INCLUDING within the first 3 days after vaccination
    - Cough

- Shortness of breath or difficulty breathing
- New loss of taste or smell
- Sore throat
- Congestion or runny nose

In all participants brought in for Unscheduled Visits, an evaluation of AEs, medical history and concomitant medications/vaccines, and collection of nasal swab samples (Section 4.5.5.3) if COVID-19 is suspected, will be performed.

Based on local COVID-19 restrictions or clinical considerations, clinic visits may not be feasible. If this cannot be arranged, an evaluation of AEs, medical history, concomitant medications/vaccines with rapid antigen testing for SARS-CoV-2 may be pursued by telemedicine visit or telephone contact. See also Section 4.5.5.3.

In participants who are able to come to the clinic for evaluation of possible COVID-19, the evaluation should include review for symptoms suggestive of COVID-19 as listed in Appendix 2, as well as 2 nasal swab samples to be sent to a qualified laboratory. This Unscheduled Visit may occur in person, by home visit, by telemedicine visit, or in the hospital (if permitted by local policy). If in-person visits are performed, collection of vital signs and body temperature, performance of symptom-directed physical examination, and measurement of pulse oximetry, are encouraged.

In some cases, such as when the participant lives remote from the study site, the participant may be given two test kits to take home as it may be necessary to perform a rapid antigen test prior to bringing the participant to the site for nasal swab RT-PCR testing. In such instances, if the first rapid antigen test is negative then a SECOND test should be performed 48 to 72 hours later.

Participants who are evaluated for COVID-19 outside of the context of an on-site Unscheduled Visit should have details of this evaluation captured in the participants' source documents as an Unscheduled Visit (see Section 7.1.1).

In order to establish virological confirmation of SARS-CoV-2 infection, testing must be performed using an FDA-cleared, Clinical Laboratory Improvement Amendment (CLIA)-certified, International Organization for Standardization (ISO) 15189-accredited, or a Vietnamese nationally accredited laboratory at the time that the test was performed. The laboratory name, test, and certification must be documented in the source. Failure to document the details will result in the exclusion of the participant's COVID-19 diagnosis from the exploratory efficacy endpoint analysis.

Participants with other severe or AEs warranting an Unscheduled visit should be managed according to standard of care, including referral to treating physician, if clinically relevant.

Beyond this protocol-specified testing, participants should be referred to their treating primary care health professional for further evaluation and treatment of potential COVID-19. This may include locally run testing to confirm the COVID-19 diagnosis and/or to exclude other potential respiratory pathogens. The investigator should continue to follow and report the COVID-19 clinical course, including disease severity.

For those participants evaluated for suspected COVID-19 symptoms or exposures, the details of the clinical evaluation and outcome will be captured in dedicated electronic case report form (eCRF) pages. This includes the confirmation or exclusion of SARS-CoV-2 infection.

#### 4.5.3.8 Final Visit/Early Termination Visit

The Final Visit should be Day 394 for all participants (including if they do not receive study vaccinations after the first vaccination). The Final Visit/ET Visit will occur at the participant's last study visit or when the participant withdraws from study participation. ET visits could occur at the clinic, by home visit, or in the hospital (if medically unstable). At this visit, participants will undergo the following: an interview for AEs, concomitant medications/vaccines, COVID-19 exposures and/or symptoms, updates to medical history, blood sampling as outlined in Section 4.5.5.3; a complete physical examination will be performed, and vital signs and body temperature will be measured.

An overview of key study procedures is provided in Figure 1.

### 4.5.4 End of Study

The EOS is defined as the date on which the last participant completes the last study visit (the Final Visit is Day 394; or ET Visit). Any additional long-term follow-up that is required for monitoring of the resolution of an AE and any additional exploratory analyses that are not conducted after this milestone passes may be appended to the clinical study report.

### 4.5.5 Additional Procedural Specifics

#### 4.5.5.1 Diary Use to Collect Solicited Adverse Events

A Diary (electronic or paper if electronic is not feasible) will be used to collect solicited AEs (i.e., reactogenicity) directly from the participant:

- Solicited local and systemic AEs will be recorded daily for 7 days starting on the day of each study vaccination in the primary vaccination series (Day 1 and 29). For participants in Phase 2 and 3a, solicited AEs will also be recorded daily for 7 days following the 3rd injection (Day 92).
    - Solicited local AEs include injection site erythema, injection site pain, injection site induration/swelling, and injection site tenderness.
    - Solicited systemic AEs include arthralgia, chills, diarrhea, dizziness, fatigue, fever (categorized by measured body temperature), headache, myalgia, and nausea/vomiting.
    - Also solicited but not categorized as AEs are antipyretic and analgesic use.
    - All participants will be trained on what information will be collected from them following each vaccination. This includes how to evaluate and score each solicited AE. Some solicited AEs are not scored directly by the participant, but the values recorded will be analyzed for severity including body temperature and visible injection site reactions (erythema or induration/swelling). Body

temperature and visible injection site reactions will be measured daily by thermometer and ruler, respectively, provided by the site. Participants should be trained on how to use the thermometer (including capture of highest body temperature if the participant perceives the onset of possible fever) and the ruler. Participants should be encouraged to complete the diary at roughly the same time of day for the 7 days following vaccination when recording the solicited AEs.

- An electronic Diary (eDiary) will involve the use of an application on the individual participant's mobile phone or through the use of a loaner mobile phone with the application installed. If an eDiary if not feasible, then a paper diary will be used by the participant. Training on how to use either the electronic or paper diary will be performed at the time of the Day 1 visit. The eDiary will use prompts to encourage the participant to enter data, alerts to inform the site and other team members of potential toxicity grade solicited AEs (if relevant). Timely follow-up of these alerts is expected.

#### 4.5.5.2 Collection of Unsolicited Adverse Events and COVID-19 Exposure/Symptoms

Weekly and monthly phone calls and clinic visits (Section 4.5.3) including unscheduled and ET, if applicable) will be used to collect unsolicited AEs and COVID-19 exposure/symptoms:

- Unsolicited AEs will be recorded through Final Visit or ET Visit. For unsolicited AEs categorized as AEs leading to discontinuation/withdrawal, medically attended adverse events (MAAEs), and serious adverse events (SAEs) documentation will occur through 365 days after the completion of the initial vaccination series (up to Day 394).
- Symptoms of COVID-19 (Section 4.5.3.7 and Appendix 2) or exposure to SARS-CoV-2 will be recorded from first study vaccine administration throughout the entire study (Final Visit or ET Visit).
- Any participant reporting onset of a possible symptom of COVID-19 or a possible exposure to SARS-CoV-2 should trigger an Unscheduled Visit (Section 4.5.3.7) for further evaluation. This visit should ideally occur within 72 hours of COVID-19 symptom onset or reported SARS-CoV-2 exposure. Should the participant manifest symptoms within the first 4 days after each vaccination, refer to Section 4.5.3.7 for further consideration.

#### 4.5.5.3 Blood and SARS-CoV-2 Sampling

Blood and SARS-CoV-2 sampling will be collected as follows:

- At Screening:

  In all participants:

  - Blood may be drawn for pregnancy testing at sites that do not allow urine pregnancy testing.
  - Blood may be drawn for FSH measurement in a female to confirm menopausal status (Appendix 3).

- Nasal swab testing for SARS-CoV-2 by RT-PCR. An additional sample will be collected and retained in the event of SARS-CoV-2 sequencing for possible variant determination. If obtained within 5 days prior to Screening period, nasal swab testing does not need to be repeated at Screening.

In Phase 1 participants:

- Blood will be drawn for additional screening assessments (complete blood count [CBC], chemistry panel, and virology [HbsAg, HCV Ab, HIV]). At Screening, in addition to sending samples to the central laboratory, a locally processed sample can be used for eligibility for enrollment. Samples will be obtained prior to study vaccine administration.

- Day 1:

  In all participants:

  - Antibody assessments for SARS-CoV-2 seropositivity for nucleocapsid antigen will be performed.
  - Blood will be drawn and saved as a baseline sample for a possible future analysis of the correlate of protection and possible future analysis for immune responses to SARS-CoV-2 variants.
  - Nasal swab testing for SARS-CoV-2 by RT-PCR. If Screening test is within 5 days of Day 1, nasal swab testing does not need to be repeated at Day 1. If screening and randomization are being performed on the same day the result of the RT-PCR test must be known to be negative prior to randomization.

  In Phase 1/2/3a and Phase 3c-1 participants:

  - Blood will be drawn as a baseline sample for testing of SARS-CoV-2 antibody responses.

- Day 8

  In Phase 1 participants:

  - Blood will be drawn for safety laboratory assessments (CBC and chemistry panel).

- Day 29

  In Phase 1 participants:

  - Blood will be drawn for safety laboratory assessments (CBC and chemistry panel), prior to study vaccine administration.

  In all participants:

  - Antibody assessments for SARS-CoV-2 seropositivity for nucleocapsid antigen will be performed.

  In Phase 1/2/3aand Phase 3c-1 participants:

  - Blood will be drawn for testing of SARS-CoV-2 antibody responses. An archive sample will also be drawn for potential future analysis for immune responses to SARS-CoV-2 variants.

- Day 36

  In Phase 1 participants:

  - Blood will be drawn for safety laboratory assessments (CBC and chemistry panel).

- Day 57:

  In Phase 1 participants:

  - Blood will be drawn for safety laboratory assessments (CBC and chemistry panel).

  In all participants:

  - Blood will be drawn and saved for a possible future analysis of a correlate of protection and possible future analysis for immune responses to SARS-CoV-2 variants.

  In Phase 1/2/3a and the Phase 3c-1 participants:

  - Blood will be drawn for testing of SARS-CoV-2 antibody responses, including anti-nucleocapsid antibody responses.

- Day 92 (Cohorts 1/2/3a and 3b only):

  In Phase 1/2/3a participants:

  - Blood will be drawn for testing of SARS-CoV-2 antibody responses, including anti-nucleocapsid antibody responses. An archive sample will also be drawn for potential future analysis for immune responses to SARS-CoV-2 variants.

- Day 120 (Cohorts 1/2/3a and 3b only):

  In Phase 2/3a participants:

  - Blood will be drawn for testing of SARS-CoV-2 antibody responses, including anti-nucleocapsid antibody responses. An archive sample will also be drawn for potential future analysis for immune responses to SARS-CoV-2 variants.

- Day 211 (Cohort 3c-1 only)

  - Blood will be drawn for testing of SARS-CoV-2 antibody responses, including anti-nucleocapsid antibody responses. An archive sample will also be drawn for potential future analysis for immune responses to SARS-CoV-2 variants.

- Unscheduled Visits:

  In all participants that have COVID-19 symptoms and/or COVID-19 exposure, the participant is asked to contact the site as soon as feasible to make arrangements for testing.

  The preferred assessment is to evaluate for SARS-CoV-2 via nasal swab. Testing of the nasal swab will be performed as follows:

  - To measure SARS-CoV-2 by reverse transcriptase-polymerase chain reaction (RT-PCR).
  - An additional sample will be collected and retained in the event of SARS-CoV-2 sequencing for possible variant determination.
  - In some cases, such as when the participant lives remote from the study site, the participant may be given two test kits to take home as it may be necessary to perform a rapid antigen test prior to bringing the participant to the site for nasal swab RT-PCR testing. In such instances, if the first rapid antigen test is negative then a SECOND test should be performed 48 to 72 hours later.

- Day 394 (Final Visit); or ET:

  In the Immunogenicity Subgroup Participants:

  Blood will be drawn for testing of SARS-CoV-2 antibody responses, including anti-nucleocapsid antibody responses. An archive sample will also be drawn for potential future analysis for immune responses to SARS-CoV-2 variants.

#### 4.5.5.4 Telephone Calls

Telephone calls will be performed at intervals specified in Section 4.5.3 and the schedule of assessments (Appendix 1) for the following reasons:

- To determine if any solicited AEs are persisting longer than 7 days after each study vaccination
- To evaluate for unsolicited AEs (including MAAEs, SAEs, AEs leading to discontinuation/withdrawal)
- To collect information for concomitant medication/vaccines
- To evaluate for symptoms of COVID-19 disease and risks of exposure to COVID-19. Solicitation of symptoms and risks of exposure to COVID-19 will follow a scripted interview provided in the Investigator Site File

Calls to the participant will be performed by a trained site team member designated by the investigator. However, in the event the participant reports any change in health status or change in medications, a qualified health care practitioner will need to complete the safety assessment.

#### 4.5.5.5 Physical Examinations

For participants in Phase 1, complete physical examination will be performed at Screening, Days 1, 8 29, 36, 92 and 120, and Final Visit (or ET). For Phase 2, 3a and 3b complete physical examination will be performed at Screening, Days 1 and 29, 92 and 120, and Final Visit (or ET). For participants in Phase 3c complete physical examination will be performed at Screening, Days 1 and 29, and Final Visit (or ET). Symptom-directed examination (if any symptoms) may be performed at other timepoints as indicated to assess changes from Screening.

- A complete physical examination will include, at a minimum, assessments of the general status of the participant, the skin of the intended study vaccine administration site, superficial lymph nodes, cardiovascular, respiratory, gastrointestinal, and neurological systems.
- A directed physical examination will include, at a minimum, assessments of the skin of the intended/actual study vaccine administration site, and/or any organ systems relevant to symptoms or AEs reported by the participant. Investigators should pay special attention to clinical signs related to previous serious illnesses.

Physical examination is to be performed by a qualified health care practitioner designated by the investigator.

#### 4.5.5.6 Height and Weight

At Screening, height and weight will be measured and recorded using calibrated instruments. Body mass index for "at risk" determination will be calculated using these values.

#### 4.5.5.7 Vital Signs, Body Temperature, and Pulse Oximetry

Vital signs will be obtained at each clinic visit. Vital signs will be measured in a semi-supine position after 5 minutes of rest and will include systolic and diastolic blood pressure, heart rate, and respiratory rate.

Body temperature will be measured according to standard of care and thermometer instructions. If body temperature is taken orally, the participant should not have consumed hot or cold beverages or smoked within 15 minutes prior to measurement.

Pulse oximetry will be performed at Unscheduled Visits, if warranted, to evaluate symptoms of COVID-19. Vital sign measurements are to be performed by a qualified health care practitioner designated by the investigator.

#### 4.5.5.8 Pregnancy Testing

For women of childbearing potential (refer to Appendix 3 for definition), urine pregnancy testing ("dipstick" test) will be performed at the sites, if available and permissible by local policy, prior to each vaccination dose. The site may perform serum testing of beta human chorionic gonadotropin if this is required by policy. The test may be performed at the local laboratory or central laboratory. The results of the pregnancy test must be read before each dose of study vaccine, and the result must be recorded in the source documents.

#### 4.5.5.9 Medical History

At Screening, medical history will also be collected including, but not limited to, any medical history that may be relevant to participant eligibility for study participation such as prior vaccinations, concomitant medications/vaccines, and previous and ongoing illnesses or injuries. Relevant medical history can also include any medical history that contributes to the understanding of an AE that occurs during study participation if it represents an exacerbation of an underlying disease/pre-existing problem. Medical history and vaccination history should be obtained for a minimum of, but not limited to, the 2-month period prior to enrollment.

Vaccination history specifically for COVID-19, SARS-CoV, or Middle East respiratory syndrome (MERS) must include **any** known exposure to these investigational or authorized vaccines.

Should review of on-study AEs or concomitant medications determine that there was a pre-existing medical condition present but not captured at Screening, this medical condition should be added to the Medical History.

# 5 PARTICIPANT SELECTION AND WITHDRAWAL CRITERIA

## 5.1 Selection of Study Participants

Study sites may be selected based on SARS-CoV-2 infection risk of the local population.

Participants will be assigned to study treatment only if they meet all the inclusion criteria and none of the exclusion criteria.

Participants who withdraw from the study after randomization will not be replaced.

Deviations from the inclusion and exclusion criteria are not allowed because they can potentially jeopardize the scientific integrity of the study, regulatory acceptability, or participant safety. Therefore, adherence to the eligibility criteria as specified in the protocol is essential.

### 5.1.1 Inclusion Criteria

Each participant must meet all the following criteria to be enrolled in this study:

**Consent and Compliance**

1. Individuals or legally authorized representatives must freely provide consent to study participation.
2. Individuals must agree to comply with all study visits and procedures (including blood and nasal swab sampling, Diary completion, receipt of telephone calls from the site, willingness to be available for Unscheduled clinic visits).
3. Individuals in Phase 1 and Phase 2 must have access to and be capable of using an eDiary.
4. Individuals of childbearing potential who are sexually active must be willing to adhere to contraceptive requirements (Appendix 3).

**Type of Participant**

5. Adult males or females of at least 18 years of age at the time of signing of the informed consent.
6. Individuals considered at risk for COVID-19 due to work or living environment in the opinion of the Investigator.

### 5.1.2 Exclusion Criteria

Individuals meeting any of the following criteria will be excluded from the study:

**Medical Conditions**

1. Significant infection or other acute illness, including body temperature >100.4°F (>38.0°C) on the day prior to or Day 1. Participants meeting this criterion may be rescheduled within the relevant window periods (Section 5.2.1). Afebrile participants with minor illnesses can be enrolled at the discretion of the investigator.
2. Pregnant or breastfeeding.

3. Known history of COVID-19 (nucleocapsid positive test is not exclusionary) or positive nasal swab SARS-CoV-2 by RT-PCR test. Note: if obtained within 5 days prior to Screening period, do not need to repeat RT-PCR at Screening.
4. Close contact with a person known to be SARS-CoV-2 positive or with a clinical diagnosis of COVID-19 within 7 days prior to enrollment. Participants meeting this criterion who remain asymptomatic for 7 days may be rescheduled for enrollment within the relevant window periods (Section 5.2.1).
5. Known history of anaphylaxis, urticaria, or other significant adverse reaction to the vaccine or its excipients. See the Investigator's Brochure for list of vaccine components.
6. Known history of anaphylaxis to other vaccines.
7. Bleeding disorder considered a contraindication to intramuscular (IM) injection or phlebotomy.
8. Immunosuppressive or immunodeficient state, asplenia, recurrent severe infections, or known to be HIV positive.
9. An underlying clinically significant acute or chronic medical condition or physical examination findings for which, in the opinion of the investigator, participation would not be in the best interest of the participant (eg, compromise the well-being) or that could prevent, limit, or confound the protocol-specified assessments.

**Prior/Concomitant Therapy**

10. Has previously received investigational or approved MERS-CoV, SARS-CoV, SARS-CoV-2 vaccines or who have plans to receive off-study COVID-19 vaccines.
11. Has received a live replicating vaccine within 28 days prior to each study vaccination or a licensed inactivated or non-replicating vaccine within 14 days prior to first study vaccination.
12. Has received treatment with immunosuppressive therapy, including cytotoxic agents or systemic corticosteroids, eg, for cancer or an autoimmune disease, within 6 months prior to Screening, or planned receipt throughout the study. If systemic corticosteroids have been administered short term (<14 days) for treatment of an acute illness, participants should not be enrolled into the study until corticosteroid therapy has been discontinued for at least 28 days prior to first study vaccine administration. Inhaled/nebulized, intra-articular, intrabursal, or topical (skin or eyes) corticosteroids are permitted.
13. Has received systemic immunoglobulins or blood products within 3 months prior to first study vaccine administration or plans to receive such products during the study.

**Other Exclusions**

14. Demonstrated inability to comply with the study procedures.
15. Investigator site staff members, employees of the Sponsor or the CRO directly involved in the conduct of the study, or site staff members otherwise supervised by the investigator, or immediate family members of any of the previously mentioned individuals.

**Additional Exclusion Criteria for Phase 1 Participants Only**

16. Individuals ≥ 60 years of age.
17. Individuals with clinically significant abnormalities in medical history or physical examination, including but not limited to, the following:
    a) Respiratory disease (e.g., chronic obstructive pulmonary disease [COPD], asthma) requiring daily medications or oxygen currently or any treatment of respiratory disease exacerbations (e.g., COPD or asthma exacerbation) warranting hospitalization or an emergency room visit or supplemental oxygen in the last 5 years.
    b) Significant cardiovascular disease (e.g., congestive heart failure, cardiomyopathy, ischemic heart disease) or history of myocarditis or pericarditis as an adult.
    c) Neurological or neurodevelopmental conditions (e.g., migraines, epilepsy, stroke, seizures in the last 3 years, encephalopathy, focal neurologic deficits, Guillain-Barré syndrome, encephalomyelitis or transverse myelitis).
    d) Significant hematologic abnormalities (e.g., sickle cell disease, beta thalassemia, coagulation disorders).
    e) Major surgery within the past 6 months.
    f) Individuals with a history of chronic liver disease.
18. Individuals with the following abnormal Screening laboratory results:
    a) Positive nasal swab SARS-CoV-2 by RT-PCR test. Note: if obtained within 5 days prior to Screening period, do not need to repeat RT-PCR at Screening.
    b) Alanine aminotransferase (ALT), aspartate aminotransferase (AST), gamma-glutamyl transferase (GGT), total bilirubin, or alkaline phosphatase >1.5 upper limit of normal.
    c) Hemoglobin <9.5 g/dL for females and <10.5 g/dL for males.
    d) Platelet count $<100 \times 10^9$/L.
    e) Other clinically significant abnormal (according to the judgment of the Investigator) Screening laboratory values.
    f) Individuals with Type 2 diabetes and Screening hemoglobin A1c >8.0%.
    g) Hepatitis B core antigen (HbsAg), Hepatitis C virus (HCV) antibody or HIV positive.

**Additional Exclusion Criteria for Phase 3c Participants Only**

19. No contraindications (as specified in the prescribing information) to receiving the ChAdOx1 vaccine.

### 5.1.3 Determination of "At Risk" of Severe COVID-19

Prior to randomization, the medical history of the participant will be reviewed. In Phases 2, 3a, Phase 3b and 3c, participants identified as being either 60 years of age or older OR who have

any of the medical conditions provided in Appendix 5 will be categorized as "at risk" of severe COVID-19

Each of the medical conditions provided in Appendix 5 is currently regarded as a known or suspected medical condition affiliated with risk of severe COVID-19 (CDC 2021).

At time of randomization, participants in Phases 2, 3a, 3b and 3c will be stratified by age (< 60 or ≥ 60 years of age) and for participants < 60 years of age by risk of severe COVID-19; a total of 3 stratification factors.

### 5.1.4 Screen Failures

Screen failures are individuals who fail to meet all inclusion criteria or who meet at least 1 of the exclusion criteria. Rescreening will not be permitted but enrollment may be delayed if the entry criterion is specific to a time window; see Section 5.2.1. Reasons for screen failure will be collected and entered in the electronic data capture (EDC) system.

### 5.1.5 Switchover/Further Study Vaccine Eligibility Criteria

At Day 92, all participants in Phase 1, 2, 3a and 3b will be reviewed for eligibility to receive further study vaccine based on meeting any of the following exclusionary criteria on Day 92. Participants meeting any of the following criteria will be excluded from receiving further study vaccine administration:

- Significant infection or other acute illness, including body temperature >100.4°F (>38.0°C) on the day prior to or day of planned Switchover/Further Study Vaccine vaccine administration (see Section 5.2.1).
- Pregnant or breastfeeding.
- Persistence of symptoms from COVID-19 disease (recovered COVID-19, asymptomatic SARS-CoV-2 infection and/or nucleocapsid positive test is not exclusionary).
- Receipt of an off-study COVID-19 vaccine since study enrollment.
- Demonstrated inability to comply with the study procedures since study enrollment.
- Development of new severe allergy to vaccine components since study enrollment. See the IB for list of vaccine components.
- New onset of bleeding disorder considered a contraindication to IM injection or phlebotomy.
- New onset of immunosuppressive or immunodeficient state, asplenia, or recurrent severe infections.
- Has received a live replicating vaccine within 28 days prior to intended Switchover/Further Study Vaccine vaccine administration or an inactivated or non-replicating vaccine within 14 days prior to intended Switchover/Further Study Vaccine vaccine administration. Please see Section 5.2.1 for guidance on deferral of study vaccination.

- Has received immunosuppressive therapy, including cytotoxic agents or has initiated systemic corticosteroid therapy. If systemic corticosteroids have been administered short term (<14 days) for treatment of an acute illness, see Section 5.2.1 for additional guidance. Inhaled/nebulized, intra-articular, intrabursal, or topical (skin or eyes) corticosteroids are permitted.
- Has received systemic immunoglobulins or blood products within the past 3 months.
- Has newly identified that they are investigator site staff members, employees of the Sponsor or the CRO directly involved in the conduct of the study, or site staff members otherwise supervised by the investigator or immediate family members of any of the previously mentioned individuals.
- Has a clinically significant acute or chronic medical condition or physical examination findings for which, in the opinion of the investigator, further study vaccine administration would not be in the best interest of the participant (eg, compromise the well-being) or that could prevent, limit, or confound the protocol-specified assessments.

## 5.2 Delaying or Discontinuing Study Vaccination and Participant Withdrawal From the Study

### 5.2.1 Delay in Study Vaccination

Body temperature must be measured and the participant asked about recent concomitant medication/vaccine receipt prior to each administration of study vaccine.

The following events constitute criteria for delay of study vaccination and the window of delay specified forthwith:

- On planned Day 1, close contact with a person known to be SARS-CoV-2 positive or with a clinical diagnosis of COVID-19 within 7 days prior to enrollment.
    - Participants meeting this criterion who remain asymptomatic for 7 days may be rescheduled for vaccination.
- Acute moderate or severe infection with or without fever at the time of dosing (delay: until afebrile for 48 hours and clinically improved according to investigator judgment)
    - Participants with minor illness and without fever may be vaccinated if deemed appropriate according to investigator judgment.
- Fever without signs of acute illness, defined as body temperature 100.4°F (≥38.0°C) (delay: until afebrile for 48 hours).
- Use of antipyretics or analgesics within 24 hours.
- Receipt of vaccines or specified steroid doses close to the time of intended study vaccination.
    - Vaccination will be deferred to confirm 28 days since receipt of a live replicating vaccine.

  - Vaccination will be deferred to confirm 14 days since receipt of an inactivated or non-replicating vaccine.
  - Vaccination will be deferred to confirm at least 28 days since receipt of systemic steroid therapy.
- Participants who are confirmed to be RT-PCR positive for SARS-CoV-2 after the first study vaccine administration, should still receive the second study vaccine administration if deemed appropriate by the Investigator. However, the second vaccine administration should be at least 28 days after the date of the positive RT-PCR result, and at the time of vaccination the participant must not meet any other criteria for delay in study vaccination (as outlined above).

Vaccination may be performed once these criteria are no longer met. For the first vaccination this will not constitute a protocol deviation as long as the vaccine is administered within 28 days after signing the informed consent. If greater than 28 days, then participants must be rescreened.

If vaccination is delayed for any of the reasons described above, subsequent study visits will be based on calendar days after the receipt of the study vaccine.

### 5.2.2 Discontinuation of Study Vaccine (Participant)

A participant may not receive additional study vaccine administrations for any of the reasons outlined in Section 5.1.5.

Additional reasons for discontinuation of study vaccine for a participant would include:

- Participant request to discontinue study vaccinations.
- Participant is lost to follow-up.
- New information becomes available that makes further participation unsafe.
- Termination of the trial study vaccinations.

Should study vaccine administration be discontinued, the participant will be encouraged to continue study participation for study evaluations through Day 394 (Final Visit).

### 5.2.3 Withdrawal/Discontinuation from the Study

Participants are free to withdraw from participation in the study at any time upon request, without prejudice to further treatment the participant may need to receive. The investigator will request that the study participant complete all study procedures pending at the time of withdrawal.

A participant will be recorded as a screen failure for the study if any of the following occur **prior to** first administration of study vaccine:

- Eligibility criteria are not met.
- The participant requests to discontinue study participation.

If, after randomization, a participant withdraws from the study, an EOS eCRF will be completed and information related to the withdrawal will be documented in the eCRF. The investigator will document whether the decision to withdraw a participant from the study was made by the participant or by the investigator, as well as which of the following possible reasons was responsible for withdrawal:

- Lost to follow-up
- Death
- Study terminated by Sponsor
- AE or SAE (specify)
- Participant request (specify)
- Protocol deviation (specify)
- Physician/Investigator decision (specify)
- Pregnancy
- Other (specify)

The investigator must capture the reason for withdrawal from the study in the eCRF for all participants that withdraw.

If, after enrollment, a participant desires to withdraw from the study because of an AE, the investigator will try to obtain agreement to follow-up with the participant until the event is considered resolved or stable and will then complete the EOS eCRF.

If a participant approaches the site requesting to receive off-study COVID-19 vaccine, the participant should be counseled and reminded of the Switchover study design (for Phase 1, 2, 3a and 3b cohorts) or the comparison with another active vaccine (for Cohort 3c) whereby all participants will receive active investigational vaccine. The participant should be encouraged to demonstrate evidence of a scheduled appointment to receive off-study COVID-19 vaccine. If, after counseling and confirmation of the appointment, a study participant maintains his/her interest in receiving off-study vaccine, he/she will be advised to preferably schedule off-study vaccine at least 28 days after last dose of study vaccine, the participant will not be eligible to receive Switchover/Further Study Vaccine vaccine (if the Switchover/Further Study Vaccine day has not passed) and the participant is asked to continue study procedures through Final Visit (Day 394).

If a participant withdraws from the study, he/she may request destruction of any remaining samples taken and not tested, and the investigator must document any such requests in the site study records and notify the Sponsor or CRO accordingly.

If a participant withdraws consent and specifies no further contact with him/her or persons previously authorized by the participant to provide this information, this should be documented by the investigator in the study source documents. If vital status (whether the participant is alive or dead) is being measured, publicly available information should be used to determine vital status only as appropriately directed in accordance with local law.

### 5.2.4 Discontinuation of Study Dosing (Study-wide)

All cases of severe COVID-19, SAEs determined to be related to study vaccine or deaths determined to be related to study vaccine will be referred to the DSMB (Section 8.10.3) in an expedited fashion.

In the event of death determined to be related to study vaccine, further vaccination will be paused until the relevant safety data has been reviewed by DSMB.

The DSMB, based on their review of the blinded or unblinded safety data (including assessment for potential vaccine-associated enhanced respiratory disease [VAERD]) will recommend whether the study should terminate, proceed unmodified, or proceed but with changes to the protocol.

Although the Sponsor has every intention of completing the study, the Sponsor reserves the right to discontinue the study at any time for clinical or administrative reasons.

## 5.3 Lost to Follow-Up

A participant will be considered lost to follow-up if he or she fails to return for scheduled visits or is unable to be contacted by the study site.

The following actions must be taken if a participant fails to return to the clinic for a required study visit:

- The site must attempt to contact the participant and reschedule the missed visit as soon as possible and counsel the participant on the importance of maintaining the assigned visit schedule and ascertain whether the participant wishes to and/or should continue in the study.
- Before a participant is deemed lost to follow-up, the investigator or designee must make every effort to regain contact with the participant (where possible, 3 telephone calls and, if necessary, a certified letter to the participant's last known mailing address or local equivalent methods). These contact attempts should be documented in the participant's medical record.

If the participant continues to be unreachable, he/she will be considered to have withdrawn from the study at the date of last study contact.

## 5.4 Replacement of Participants

Participants who withdraw from the study after randomization will not be replaced.

# 6 STUDY TREATMENTS

## 6.1 Method of Assigning Participants to Study Groups

Assignment of participants to Study Groups will proceed using an IRT system. The site personnel (study coordinator or specified designee) will be required to enter or select information including but not limited to the password, the protocol number, participant age and the participant code (a unique number identifying the participant), and participant's relevant underlying health conditions that will be used to determine "at risk" status prior to randomization. The site personnel will then be provided with a vaccine assignment. The IRT system will provide an unblinded confirmation report containing the participant code and study intervention allocation assigned. The confirmation report must be stored in the site's unblinded files away from the view of blinded site staff.

Participants will then be randomly assigned and stratified as described in Section 4.5.2. Treatment designation will be assigned in the IRT.

Confirmation of a participant's eligibility to receive additional study vaccine must be entered into the IRT prior to dispensing the assigned vaccination on Day 92.

## 6.2 Study Vaccine Manufacturer

For Phase 1, 2, 3a and 3b ARCT-154 and placebo (0.9% sterile saline) constitute the study vaccine.

For Phase 3c ARCT 154 and the Astra Zeneca vaccine ChAdOx1 constitute the study vaccines.

Arcturus Therapeutics, Inc. (10578 Science Center Drive, Suite 150, San Diego, CA 92121 USA), as owner of the drug product, is the designated manufacturer of ARCT-154. Arcturus contracts manufacture of ARCT-154 as follows:

- Catalent Pharma Solutions (726 Heartland Trail, Madison, WI 53717 USA) is responsible for manufacture of drug substance
- Polymun Scientific (Immunbiologische Forschung GmbH, Donaustrasse 99, 3400 Klosterneuburg, Austria) is responsible for manufacture of bulk drug product
- Recipharm AB (Wasserburger Arzneimittelwerk GmbH, Herderstrasse 2, D – 83512 Wasserburg, Germany) is responsible for fill and finish of drug product

0.9% saline and ChAdOx1 are sourced from commercial supply and emergency use supply (in Vietnam), respectively and manufacturer details can be found in the prescribing information.

## 6.3 Study Vaccine, Dosage, and Route of Administration

The ARCT-154 study vaccine is a lipid-nanoparticle-formulated RNA product. The RNA comprises a replicon based upon an alphavirus in which RNA coding for the virus structural proteins has been replaced with RNA coding for the SARS-CoV-2 S glycoprotein containing the D614G variant mutation.

The nanoparticle composition includes 4 lipid excipients (ionizable cationic lipid, Arcturus proprietary lipid; neutral lipid, 1,2-distearoyl-sn-glycero-3-phosphocholine; cholesterol; and polyethylene glycol-lipid conjugate).

ARCT-154 is provided as a lyophilized formulation in a 12-mL-size glass vial presentation that allows for multiple 5.0-µg doses of ARCT-154 after reconstitution. The diluent for reconstitution consists of sterile 0.9% sodium chloride for injection, that is USP, BP or equivalent grade, and is preservative-free.

The comparator (placebo) for Phase 1, 2, 3a and 3b is sterile 0.9% sodium chloride for injection that is United States Pharmacopeia (USP), British Pharmacopeia (BP) or equivalent grade, and is preservative-free.

The comparator for Phase 3c is the Astra Zeneca vaccine ChAdOx1, which is already authorized for use in Vietnam. ChAdOx1 active substance is a chimpanzee Adenovirus encoding the SARS-CoV-2 Spike glycoprotein. Further details can be found in the prescribing information for ChAdOx1.

The study vaccines will be prepared by an unblinded trained site staff member in accordance with the Pharmacy Manual, and outside of the view of blinded team members. Study vaccines will be matched for volume (0.5 mL) and appearance (1-mL syringe) prior to administration. Study vaccine will be administered by IM injection to the deltoid muscle by an unblinded health care provider who will not be involved in assessments of any study endpoints.

Study vaccine preparation and administration should be performed in an area outside of the view of blinded team members. All documents containing unblinded information must also be kept out of view of the blinded team members and the study participants. Unblinded team members will not otherwise participate in other study-related procedures or assessments of the participant.

The IM injection of the study vaccine will be into the lateral aspect of the deltoid muscle of the non-dominant arm, where possible.

Epinephrine for subcutaneous (S.C.) injection, diphenhydramine for intravenous injection, and any other medications and resuscitation equipment for the emergency management of anaphylactic reactions must be available in close proximity to the room where the vaccination is administered.

See Section 4.5.3.2 for additional guidance on procedures during the Dose Administration visit.

## 6.4 Management of Clinical Supplies

### 6.4.1 Investigational Product Packaging and Storage

The Sponsor or designee will provide the Investigator with:

- packaged ARCT-154 containers labeled in accordance with specific country regulatory requirements.
- sodium chloride intended for use as diluent and placebo will be provided
- ChAdOx1 vaccine

See the Pharmacy Manual for additional guidance study vaccine packaging and study vaccine storage requirements.

The study staff is required to document the receipt, dispensing, and return/destruction of ARCT-154 and ChAdOx1 supplies provided by the Sponsor or designee. The study center must destroy or return for destruction all unused frozen vials of ARCT-154/ ChAdOx1 to the Sponsor or designee. Vials of ARCT-154/ ChAdOx1 should be destroyed by the study center or delegate **only** after drug accountability has been done by the unblinded study monitor. Destruction or return of study vaccine must be documented.

### 6.4.2 Clinical Supplies for Reconstitution and Administration of Study Vaccine

Syringes, needles and filters will be supplied by the Sponsor or designee. See the Pharmacy Manual for guidance on the use of ancillary supplies for vaccinations.

### 6.4.3 Study Vaccine Accountability

The investigator will maintain accurate records of receipt of all study vaccines (ARCT-154, placebo/diluent, ChAdOx1), including dates of receipt. In addition, accurate records will be kept regarding when and how much study vaccine is administered to each participant in the study. Only participants enrolled in the study may receive any study vaccine and only authorized site personnel may supply or administer the study vaccine. Reasons for departure from the expected dispensing regimen must also be recorded. At the completion of the study, to satisfy regulatory requirements regarding drug accountability, all study vaccines will be reconciled and destroyed according to applicable regulations. Further guidance and information for the final disposition of unused study vaccines are provided in the Pharmacy Manual.

### 6.4.4 Other Supplies for Participant Use

Study sites will distribute sponsor-provided thermometers (for body temperature measurement) and rulers to measure injection site erythema and induration/swelling reactions, respectively. The measurements will be captured in the Diary provided by the site. Electronic Diaries will be used wherever possible and, if a participant does not have his/her own smart phone devices for eDiary use, the site will provide an electronic alternative or a paper diary.

If the participant should have suspected or confirmed COVID-19 and cannot come into the clinic for evaluation, the participant may undergo a telemedicine or Home Visit.

## 6.5 Overdose Management

Study vaccine (ARCT-154, placebo or ChAdOx1) errors (including overdose, underdose, and administration error) must be documented as protocol deviations. A brief description should be provided of the deviation, including whether the participant was symptomatic (list symptoms) or asymptomatic, and whether the event was accidental or intentional.

Dosing details should be captured on the Dosing eCRF.

An overdose is the accidental or intentional administration of a vaccine in an amount higher than the dose being studied. An overdose or incorrect administration of study vaccine is not itself an AE, but it may result in an AE. If the participant receives a dose of study vaccine (ARCT-154,

placebo or ChAdOx1) that exceeds protocol specifications and the participant is symptomatic, the symptom(s) should be documented as an AE.

Should an overdose occur, the investigator or designee should contact the CRO medical monitor or designee within 24 hours.

There are no known treatments for potential overdose of study vaccine. In the event of an overdose, the individual should be monitored and provided with symptomatic treatment as appropriate.

Unless there is a user error leading to failure to administer any volume of study vaccine, "catch-up" vaccine administrations will not be performed.

## 6.6 Blinding

The study vaccines will be administered in an observer-blind fashion.

Each of the study vaccines will be prepared by an unblinded trained team member and in accordance with the Pharmacy Manual.

Unblinded personnel (of limited number) will be assigned to vaccine accountability procedures and will prepare study vaccine for all participants. These personnel will have no study functions other than study vaccine management, documentation, accountability, preparation, and administration. They will not be involved in participant evaluations and will not reveal the identity of study vaccine to either the participant or the blinded study site personnel involved in the conduct of the study unless this information is necessary in the case of an emergency.

- Unblinded health care providers will administer the study vaccine. They will not be involved in assessments of any study endpoints.
- All documents containing unblinded information must also be kept out of view of the blinded team members and the study participants.
- Unblinded site monitors not involved in other aspects of monitoring will be assigned as the study vaccine accountability monitors. They will have responsibilities to ensure that sites are following all proper study vaccine accountability, preparation, and administration procedures.
- An unblinded DSMB will perform the ongoing review of safety data from enrolled participants to determine if there are any unexpected risks that warrant modification or termination of the study. The DSMB will provide recommendations to the Sponsor following each data review.

In order to maintain an observer-blind design, investigators, site staff, participants, and CRO staff with oversight of study conduct will remain blinded to vaccine assignments for the study duration. The Sponsor representatives with direct oversight of the study will remain blinded to individual participant vaccine assignments until the time of study unblinding at the final analysis. The Switchover/Further Study Vaccine will occur in a blinded fashion and participants and blinded site and Sponsor personnel will remain blinded to treatment assignment until End of Study (EOS).

Additional members of the Sponsor team that are not involved in direct oversight of the study may receive unblinded study data but will not share any unblinded information with the Sponsor team overseeing the study. Sponsor staff and other persons that will become unblinded at the various data analysis time points will be specified in a written unblinding plan prior to unblinding occurring.

The dosing assignment will be concealed by having the unblinded personnel prepare the study vaccine in a secure location not accessible or visible to blinded study staff members. Only delegated unblinded site staff will perform the injection of prepared study vaccine. Once the injection is completed, only the blinded study staff will perform further assessments and interact with the participants. Access to the randomization code will be strictly controlled at the pharmacy. All other site staff performing study-related assessments will be blinded for the duration of the study.

### 6.6.1 Breaking the Blind

An individual participant's study vaccine assignment will not be unblinded to the investigator or other blinded study site staff until the EOS unless awareness of the vaccine assignment is relevant to the care of the study participant. If the blind needs to be broken because of a medical emergency, the investigator may unblind an individual participant's treatment allocation.

To the extent possible before unblinding, the investigator should contact the medical monitor to discuss the medical emergency and the reason for revealing the actual treatment received by that participant. The study vaccine assignment will be unblinded through IRT. Reasons for unblinding must be clearly explained and justified in the eCRF. The date on which the code was broken together with the identity of the person responsible must also be documented.

Participants who choose to receive vaccination with licensed COVID-19 vaccine will be encouraged to remain in the study, but those that choose to leave the study to pursue alternate vaccination will not be unblinded to study treatment unless there is an unexpected post study SAE attributed as related to study vaccine by the study investigator.

## 6.7 Compliance With Study Treatment

When participants are dosed at the site, they will receive the study vaccine directly from the Investigator or designee under medical supervision. The date and time of each dose administered in the clinic will be recorded in the source documents and recorded in the eCRF. The receipt of study vaccine and study participant identification will be confirmed at the time of dosing by a member of the study site personnel other than the person administering the study vaccine.

## 6.8 Concomitant Therapies and Procedures

### 6.8.1 Concomitant Therapy

Study site staff must question the participant regarding any medications taken (including herbal/homeopathic substances) and vaccinations received by the participant. The following information will be recorded in the eCRF:

- All non-study vaccinations administered within the period starting 28 days before the first dose of study vaccine and for the duration of study participation.
- All concomitant medications and non-study vaccinations taken through 28 days after each study vaccination.
- Any concomitant medications/vaccines used to prevent or treat COVID-19.
- Any concomitant medications/vaccines relevant to or for the treatment of a solicited or unsolicited AE, an SAE, an MAAE, or an AE leading to discontinuation/withdrawal.
- Participants will be asked prior to vaccination and will be asked for 7 days after each vaccination of the initial series in the Diary if they have taken any antipyretic or analgesics to prevent or treat vaccine-associated side effects (AEs). For participants in Phase 2/3a, the diary will also be completed for 7 days after the 3$^{rd}$ dose of study vaccine (Day 92).

All medications and interventions necessary for the appropriate care of the study participant, particularly to treat COVID-19, should be administered and appropriately documented along with the AE.

All concomitant medications/vaccines must be recorded in the eCRF until 28 days after each dose of study vaccine. Thereafter, only vaccines and concomitant medications associated with an SAE, MAAE, or AEs leading to discontinuation/withdrawal will be entered in the eCRF.

**Prohibited Concomitant Vaccines**

Receipt of off-study COVID-19 vaccine is actively discouraged for enrolled participants. Any participant who receives an off-study COVID-19 vaccine will not receive further study vaccinations. All participants should remain in the study until the Final Visit (Day 394). If the off-study vaccine becomes known to the site prior to Switchover/Further Study Vaccine, the participant will not receive Switchover/Further Study Vaccine vaccinations and will be requested to stay in the study through Final Visit (Day 394).

#### 6.8.1.1 Concomitant Procedures

A concomitant procedure is any therapeutic intervention (eg, surgery/biopsy, physical therapy) or diagnostic assessment (eg, blood gas measurement, bacterial cultures) performed between signing of the informed consent and the participant’s last protocol-specified study visit.

All concomitant procedures must be recorded in the eCRF until 28 days after each study vaccine. Thereafter, only concomitant procedures associated with an SAE, MAAE, or evaluation of COVID-19 cases will be entered in the eCRF.

## 6.9 Dose Modification

No dose modifications will be allowed.

## 6.10 Intervention After the End of the Study

No intervention will be provided to study participants after the EOS.

# 7 STUDY ASSESSMENTS

Technical considerations for study assessments are described throughout Section 4 of the protocol.

The schedule of study assessments is provided in Appendix 1.

## 7.1 Efficacy Assessments

### 7.1.1 Evaluation of Participants With Suspected COVID-19

Participants with suspected COVID-19 will be evaluated for the presence of potential symptoms and clinical findings of COVID-19 as tabulated in Appendix 2.

To be considered as a protocol-defined COVID-19 case, virological confirmation (by RT-PCR) of SARS-CoV-2 and demonstration of at least one of the symptoms or clinical findings listed in Appendix 2 are required. This criterion may be met by any of the following tests:

RT-PCR test or other equivalent nucleic acid amplification–based test (NAAT). Results from lab test will only be considered acceptable if it was obtained using:

- An assay accredited by the Vietnamese MOH;
- Additional accreditation of the assay by FDA is also preferred;
- OR an assay performed in a laboratory that is currently CLIA-certified;
- OR an assay performed by a laboratory accredited according to the ISO 15189 standard.

Case definitions for the evaluation of COVID-19 and severe COVID-19 are provided in Appendix 2. All suspected COVID-19 cases will undergo tiered review:

- All SARS-CoV-2-confirmed cases are reviewed by a blinded, independent adjudication committee (AC) against the symptoms and scales provided in Appendix 2. The AC will determine if the severity of symptoms of COVID-19 accompanying SARS-CoV-2 virological confirmation aligns with protocol-defined COVID-19 case definitions, asymptomatic SARS-CoV-2 infection, or atypical COVID-19 case presentations. COVID-19 cases (protocol-confirmed and atypical) will also be evaluated by severity using FDA and World Health Organization (WHO) criteria.

#### 7.1.1.1 Severe COVID-19

Two definitions will be used to define severe COVID-19: FDA and WHO (Appendix 2). Confirmed COVID-19 must meet the protocol-defined COVID-19 case definition and either the FDA or the WHO criteria for severe COVID-19 in order to be considered an event of Severe COVID-19 for evaluation of this endpoint. These criteria are summarized in Appendix 2.

#### 7.1.1.2 Asymptomatic COVID-19

Asymptomatic SARS-CoV-2 infection is defined as a participant with a positive NAAT test for SARS-CoV-2 without the presence of protocol-specified COVID-19 (Appendix 2) or atypical

COVID-19 symptoms. These cases will be collected as disease events but not analyzed towards COVID-19 cases.

#### 7.1.1.3 Atypical COVID-19

Atypical COVID-19 is defined as a participant with a positive NAAT test for SARS-CoV-2 virus and contemporaneous symptoms that are not those listed in Appendix 2 but nevertheless clinically suspected to represent a manifestation of COVID-19. These cases will be used for exploratory analyses of COVID-19 cases only.

#### 7.1.1.4 Death Attributed to COVID-19

Any participant who dies during study participation will be evaluated for suspected/confirmed causes of death. Autopsy results and histopathology results, as available, will be requested. Any death attributed to confirmed COVID-19 will be evaluated for the death endpoint. For cases of clinical suspicion of COVID-19 as the cause of the death, these cases will be subject to review by the blinded AC for confirmation of whether these meet the 'Death Attributed to COVID-19' endpoint.

## 7.2 Immunogenicity Assessments

For Phase 1, Phase 2, Phase 3a and Phase 3c-1 participants, blood samples for immunogenicity assessments will be collected at the time points indicated in Section 4.5.5.3 and the Schedule of Assessments (Appendix 1). On Days 1, 29, and 92, blood samples for immunogenicity assessment will be collected before administration of each study vaccine. The following analytes will be measured:

1) Assays run at [redacted] Vietnam
    a) Serum NAb titer against SARS-CoV-2:
        i. Genscript SARS-CoV-2 surrogate virus neutralization test (sVNT): This sVNT assay is a functional Enzyme-Linked Immunosorbent Assay (ELISA) intended for qualitative or semi-quantitative detection of total neutralizing antibodies in human serum, K2-EDTA plasma, peripheral blood and blood plasma that block the binding of SARS-CoV-2 to the human ACE2 receptor of host cells. This binding assay has been shown to correlate well with cell-based neutralizing antibody assays. When run with an appropriate reference standard, the data can be converted to international units using the WHO reference standard conversion. The assay will be performed on samples from participants in Phase 1/2/3a. It may also be performed on samples from participants in Phase 3c-1.
        ii. Plaque reduction neutralization test [PRNT]: This live virus assay is performed in a biosafety level 3 (BSL3) laboratory using a Wuhan reference standard strain and delta variant strain isolated at the Department of Virology, National Institute of Hygiene and Epidemiology. Neutralizing antibody titers are calculated as the highest serum dilution that results in 50% reduction in the

number of virus plaques (PRNT50). This assay will be performed on samples from all participants in Phase 1 and the first 50 participants in Phase 2. It may also be performed on samples from additional participants in Phase 2/3a and/or 3c-1.

iii. Genscript SARS-CoV-2 pseudovirus neutralization (MNT) assay kit Luc reporter: this kit provides a cell-based functional assay for measuring the potency of candidate drugs in neutralizing SARS-CoV-2 S protein. The S protein described in this kit is located on the surface of the lentivirus-based pseudovirus particles, so the pseudovirus can bind to the ACE2 receptor overexpressed on the target cells, simulating the fusion process of the coronavirus-cell and cell-cell membrane. After infection, the pseudovirus can express the luciferase gene in the target cell. By measuring the bioluminescence signal, the extent of pseudovirus infection can be determined. When a candidate drug that is able to neutralize SARS-CoV-2 binds to the S protein, the interaction of ACE2 and S proteins on the pseudovirus is blocked, preventing the pseudovirus infection and resulting in a decrease in the bioluminescence signal. Therefore, the sample potential for neutralizing SARS-CoV-2 can be determined from the signal reduction. This assay may be performed on samples from participants in Phase 3c-1 and on samples from participants in Phase 1/2/3a.

b) Serum IgG antibodies binding the SARS-CoV-2 spike protein

i. Siemens ADVIA Centaur XP/XPT system: this is a fully automated 2-step antigen sandwich immunoassay using acridinium ester chemiluminescent technology. The solid phase contains a preformed complex of streptavidin-coated microparticles and biotinylated SARS-CoV-2 recombinant antigens. This reagent complex is used to capture full-length SARS-CoV-2 S protein-specific antibodies in the patient samples. This assay will be performed on samples from participants in Phase 1/2/3a and 3c-1.

2) Assays run at [redacted] USA

a) Serum NAb titer against SARS-CoV-2

i. Pseudovirus microneutralization test (MNT; (Wuhan-Hu-1 [D614G strain])): The SARS CoV-2 MNT is a cell-based assay that is designed to determine the ability of anti-SARS CoV-2 Spike neutralizing antibodies to inhibit the infection of 293T-ACE2 cells by D614G strain-specific SARS CoV-2 Spike Reporter Virus Particles (RVP) which express green fluorescent protein (GFP). Results can be converted to IU/mL using a WHO reference standard calibration factor. This assay will be performed on samples from all participants in Phase 1 and the first 150 participants who received ARCT-154 in the initial vaccination series in Phase 2. This assay may also be performed on samples from additional participants in Phase 1/2/3a and/or participants in Phase 3c-1.

ii. ACE2 receptor binding inhibition sVNT assay: The V-PLEX COVID-19 Coronavirus (ACE2) Kit is a multiplex neutralization assay that measures the

antibodies capable of blocking the binding of angiotensin-converting enzyme 2 (ACE2) to the SARS-CoV-2 Spike and RBD antigens. The Panel serves as a high-throughput alternative to traditional neutralization assays. The Panel 13 (IgG) Kit is a multiplex serology assay for antibodies to ten Spike antigens from variants of SARS-CoV-2, including the Alpha, Beta, Gamma, Delta, and several emerging variants. Panel 13 has Spike antigens from the following lineages: A (WT), (B.1.1.7), (B.1.351), (B.1.526.1), (B.1.617), (B.1.617.1), (B.1.617.2), (B.1.617.3), (P.1), and (P.2). This assay will be performed on samples from all participants in Phase 1 and the first 150 participants who received ARCT-154 in the initial vaccination series in Phase 2. This assay may also be performed on samples from additional participants in Phase 1/2/3a and/or participants in Phase 3c-1.

iii. Delta virus MNT: This is a cell-based assay designed to determine the ability of anti-SARS CoV-2 Spike neutralizing antibodies to inhibit the infection of 293T-ACE2 cells by Delta strain-specific SARS CoV-2 Spike Reporter Virus Particles (RVP) which express green fluorescent protein (GFP). The readout is ID50. This assay will be performed on samples from all participants in Phase 1 and the first 150 participants who received ARCT-154 in the initial vaccination series in Phase 2. This assay may also be performed on samples from additional participants in Phase 1/2/3a and/or participants in Phase 3c-1.

b) Serum IgG antibodies binding the SARS-CoV-2 spike

i. Mesoscale Discovery (MSD) multiplex assay. MSD multiplex indirect binding ECL is a quantitative electrochemiluminescence (ECL) assay designed to detect antibodies to total immunoglobulin G (IgG) against the full-length spike, RBD, and N antigens of the Wuhan strain SARS-CoV-2. Results can be converted to BAU/mL using WHO reference standard calibration factors provided by MSD. This assay may be performed on samples from participants in Phase 1/2/3a and/or Phase 3c-1.

Additional testing may also be performed using other assays.

Immunogenicity laboratory results will only be considered acceptable if obtained using an assay accepted by the Vietnamese MOH.

Sample aliquots will be designed to ensure that backup samples are available and that adequate vial volumes may allow for further testing. The actual time and date of each sample collected will be recorded in the eCRF, and unique sample identification will be used to maintain the blind at the laboratory at all times and to allow for automated sample tracking and storage. Handling and preparation of the samples for analysis, as well as shipping and storage requirements, will be provided in a separate study manual.

## 7.3 Reactogenicity

Reactogenicity will be evaluated in all participants enrolled in the study (Section 7.4.1.1.1.1). Reactogenicity will be evaluated following receipt of each vaccination of the initial series and

evaluated in those participants who provide at least 1 response to the Diary. Further details on the reactogenicity analysis set are provided in Section 8.2.

Participants will complete a Diary collecting solicited AEs for 7 days following each vaccine administration of the initial series.

## 7.4 Safety Assessments

Safety assessments will include monitoring and recording of solicited and unsolicited AEs, MAAEs, AEs leading to discontinuation of the study vaccine/study withdrawal, SAEs, vital signs, body temperature and (in participants who are evaluated for suspected COVID-19) pulse oximetry.

For Phase 1 participants, safety blood sampling will include CBC, chemistry panel. Virology testing (HbsAg, HCV Ab, and HIV) will also be performed at Screening. Procedures for collection, handling, and storage of blood collected for safety assessments will be detailed in the Laboratory Manual.

### 7.4.1 Adverse Events

In this study, AEs include subcategories of AEs collected by solicitation of participants as to types of signs and symptoms observed following vaccination (reactogenicity), referred to as "solicited AEs", and a general category of unsolicited AEs that includes any spontaneously reported or observed AE occurring after the signing of informed consent.

#### 7.4.1.1 Definitions

##### 7.4.1.1.1 Adverse Events

The investigator is responsible for reporting all AEs that are observed or reported during the study, regardless of their relationship to the study vaccine or their clinical significance. AE collection will begin after the signing of informed consent; however, AEs that occur prior to the administration of first dose of study vaccine (ie, non-treatment-emergent AEs) will be listed separately in the clinical study report.

An AE is defined as an untoward medical occurrence associated with the use of a medicinal product in humans, whether or not considered related to the medicinal product.

An AE can therefore be any of the following:

- Any unfavorable and unintended sign (including an abnormal laboratory finding), symptom, or disease temporally associated with the use of the study vaccine, whether or not considered related to the study vaccine
- Any new disease or exacerbation of an existing disease (a worsening in the character, frequency, or severity of a known condition)
- Recurrence of an intermittent medical condition (eg, headache) not present at baseline
- Any deterioration in a laboratory value or other clinical test (eg, electrocardiogram, X-ray) that is associated with symptoms or leads to discontinuation from the study vaccine

7.4.1.1.1.1 Solicited adverse events

The term "reactogenicity" refers to selected signs and symptoms ("reactions") occurring in the hours and days following a vaccination. These signs and symptoms are collected as solicited AEs from participants by use of the Diary for 7 consecutive days following each of the first 2 study vaccine administrations.

- The following solicited AEs are included in the Diary:
  - **Solicited local AEs:** injection site erythema, injection site pain, injection site induration/swelling, and injection site tenderness
  - **Solicited systemic AEs:** arthralgia, chills, diarrhea, dizziness, fatigue, fever (categorized by measured body temperature), headache, myalgia, and nausea/vomiting

The occurrence of each of these solicited AEs is regarded as a vaccine-related AE.

Also solicited from participants but not categorized as an AE is the use of antipyretics and analgesics to prevent or treat solicited AEs.

Solicited AEs are graded for severity according to scales defined in the Center for Biologics Evaluation and Research's Guidance: Toxicity Grading Scale for Healthy Adult and Adolescent Volunteers Enrolled in Preventive Vaccine Clinical Trials (DHHS 2007), Appendix 4.

Solicited AEs may also lead to circumstances that warrant collection of the events as unsolicited AEs. This includes any solicited AE that leads to:

- a medically attended visit
- an SAE
- a discontinuation of study vaccine
- a withdrawal from the study
- or any solicited AE that persists beyond 7 days after the first study vaccine administration.

Furthermore, should the site determine that the participant experienced and failed to enter a Grade 3 or Grade 4 solicited AE in the Diary during the time when the Diary was accepting data entry, this Grade 3 or Grade 4 solicited AE should be entered as an unsolicited AE in the CRFs.

7.4.1.1.1.2 Unsolicited adverse events

Unsolicited AEs are defined as any spontaneously reported or discovered AE.

Unsolicited AEs will be collected in all participants who receive at least 1 dose of study vaccine and are classified according to the following:

- mild, moderate, or severe
- whether or not the AE was categorized as an SAE, an MAAE, and/or an AE leading to discontinuation/withdrawal

- whether or not the AE was related to study vaccine or study procedure in the judgment of the investigator

7.4.1.1.2 Serious Adverse Events

An SAE is defined as any event that:

- results in death
- is immediately life-threatening
- requires inpatient hospitalization or prolongation of existing hospitalization
- results in persistent or significant disability/incapacity
- is a congenital anomaly/birth defect arising from a pregnancy conceived after receipt of study vaccine

Important medical events that may not result in death, be life-threatening, or require hospitalization may be considered SAEs when, based upon appropriate medical judgment, they may jeopardize the participant or may require medical or surgical intervention to prevent one of the outcomes listed in this definition. Examples of such medical events include allergic bronchospasm requiring intensive treatment in an emergency room or at home, blood dyscrasias or convulsions that do not result in inpatient hospitalization, or the development of drug dependency or drug abuse.

7.4.1.1.3 Suspected Unexpected Serious Adverse Reactions

A suspected unexpected serious adverse reaction (SUSAR) is any SAE wherein a causal relationship to study vaccine is at least reasonably possible and wherein the nature or severity of the AE is not consistent with the Reference Safety Information in the IB.

7.4.1.1.4 Medically Attended Adverse Events

An MAAE is an AE that leads to an unscheduled visit (including a telemedicine visit) with a healthcare provider (HCP, eg, nurse, nurse practitioner, physician's assistant, physician). This would include visits to a study site for unscheduled assessments (eg, rash assessment, possible COVID-19 symptoms, and visits to HCPs external to the study site for acute changes in health (eg, urgent care, primary care physician). Investigators will review unsolicited and solicited AEs for the occurrence of any MAAEs. If a solicited AE leads to an unplanned HCP visit for evaluation of the symptoms, this will be entered as an unsolicited AE (also marked as an MAAE) in addition to the solicited AE captured. The following considerations of MAAEs also apply:

- Routine study visits will not be considered medically attended visits.
- AEs observed in the immediate 30-minute post-vaccination observation period will be regarded as an MAAE if:
  - Treatment of the AE is required prior to discharge OR
  - Extension of observation is required due to clinical concerns or unstable clinical findings

#### 7.4.1.1.5 Adverse Events Leading to Discontinuation/Withdrawal

Any AE that leads to discontinuation of the vaccine (Section 5.2.2) and/or withdrawal from the study (Section 5.2.3) will be regarded as an AE leading to discontinuation/withdrawal. Investigators will review reasons for discontinuation/withdrawal and report this on the appropriate eCRF page (AE eCRF for AEs leading to withdrawal of the study vaccine and Study Termination eCRF for reason for study discontinuation).

#### 7.4.1.1.6 Adverse Event of Special Interest

**No AESIs are specified for this study.**

#### 7.4.1.1.7 Cases of COVID-19 or Asymptomatic SARS-CoV-2 Infection

Onset of symptoms (eg, respiratory tract infection) suggestive of COVID-19 may first be reported by sites as an AE or SAE. As reflected in other sections of the protocol, symptoms suggestive of COVID-19 should be evaluated promptly for virological confirmation of SARS-CoV-2 virus. In addition, should further evaluation of the clinical case determine that the case is virologically confirmed COVID-19 or asymptomatic SARS-CoV-2, these infections must be reported to the Sponsor within 24 hours.

All events that meet the definition of an SAE will be captured as SAEs, regardless of whether they are regarded as COVID-19-related events.

### 7.4.1.2 Eliciting and Documenting Adverse Events

AEs will be assessed from the time the participant signs the ICF until exit from the study. Any ongoing disease/condition (eg, intercurrent illness) that a participant is experiencing at the time of signing informed consent and during Screening should be captured as medical history.

Solicited AEs will be solicited by Diary for 7 days after each of the vaccinations in the initial series in all participants.

At every clinic visit and on weekly/monthly phone calls, participants will be asked a standard nonleading question to elicit any medically related changes in their well-being. Any and all new changes to the participant's health (including exacerbation of underlying disease) is regarded as an unsolicited AE.

In addition to participant observations, AEs identified from any study data (eg, physical examination findings, vital signs, or pulse oximetry) or identified from review of other documents (eg, participant Diary) that are relevant to participant safety will be documented on the AE page in the eCRF.

All solicited AEs through 7 days after each vaccination of the initial series will be collected. Continuing solicited AEs persisting beyond 7 days after each vaccination or that meet the definition of a SAE or MAAE will be captured as unsolicited AEs, categorized accordingly and followed until stabilization/resolution.

All unsolicited AEs through 28 days after study vaccine administration will be collected.

Unsolicited AEs that meet the protocol definition of MAAE, SAE, or AEs leading to withdrawal/discontinuation will be captured for the duration of study participation.

If the investigator learns of any SAE, including a death, at any time after a participant has been discharged from the study, and he/she considers the event to be reasonably related to the study vaccine or study participation, the investigator must promptly notify the Sponsor.

#### 7.4.1.2.1 Assessment of Severity

The severity, or intensity, of an AE refers to the extent to which an AE affects the participant's daily activities. Changes in the severity of an AE should be documented to allow an assessment of the duration of the event at each level of intensity. AEs characterized as intermittent do not require documentation of onset and duration of each episode.

**Unsolicited AEs:** The intensity of unsolicited AEs will be rated as mild, moderate, or severe using the following criteria:

| | |
|---|---|
| Mild: | These events require minimal or no treatment and do not interfere with the participant's daily activities.<br>OR<br>An event usually transient in nature and generally not interfering with normal activities. |
| Moderate: | These events result in a low level of inconvenience or concern with the therapeutic measures. Moderate events may cause some interference with normal functioning.<br>OR<br>An AE that is sufficiently discomforting to interfere with normal activities. |
| Severe: | These events interrupt a participant's usual daily activity and may require systemic drug therapy or other treatment. Severe events are usually incapacitating.<br>OR<br>An AE that is incapacitating and prevents normal activities. |

**Solicited AEs:** The intensity of solicited AEs will be graded for severity as Grade 0, Grade 1 (mild), Grade 2 (moderate), Grade 3 (severe), or Grade 4 (potentially life threatening). Further details on the toxicity grading scales are provided in Appendix 4.

#### 7.4.1.2.2 Assessment of Causality

The investigator's assessment of an unsolicited AE's relationship to the study vaccine is part of the documentation process, but it is not a factor in determining what is or is not reported in the study. If there is any doubt as to whether a clinical observation is an AE, the event should be reported.

The relationship or association of the study vaccine in causing or contributing to the AE will be characterized using the following classification and criteria:

| | |
|---|---|
| Related: | There is a 'reasonable possibility' that the reported adverse event was caused by the study vaccine. 'Reasonable possibility' means that there is evidence to suggest a causal relationship between the event and the study vaccine. |
| Unrelated: | There is not a 'reasonable possibility' that the adverse event was caused by the study vaccine. |

Solicited AEs are, by definition, regarded as related to study vaccine. Therefore, causality is not assessed.

#### 7.4.1.3 Reporting Adverse Events

Non-serious unsolicited AEs are recorded through 28 days after study vaccine administration, unless they meet the protocol definition of MAAE, SAE, or AEs leading to withdrawal/discontinuation, which will be recorded and reported for the duration of study participation.

All unsolicited AEs reported or observed during the study will be recorded on the AE page in the eCRF. Information to be collected includes the following:

- event term
- date of onset
- date of resolution/stabilization
- for AEs occurring within 24 hours of the vaccination only: time of onset
- investigator-specified assessment of severity and relationship to the study vaccine or study procedure
- for AEs initiating and resolving within 24 hours of the vaccination only: time of resolution of the event
- seriousness
- any required treatment or evaluations
- outcome
- whether the AE met the protocol definition of MAAE
- whether the AE led to study vaccine withdrawal

Adverse events resulting from concurrent illnesses, reactions to concurrent illnesses, reactions to concurrent medications, or progression of disease states must also be reported. All AEs (solicited and unsolicited) will be followed to adequate resolution. The Medical Dictionary for Regulatory Activities (MedDRA) dictionary will be used to code all unsolicited AEs.

Any medical condition that is present at the time that the participant is screened but does not deteriorate should be reported as medical history rather than as an AE. However, if this medical condition deteriorates at any time during the study, it should be recorded as an AE.

##### 7.4.1.3.1 Reporting Serious Adverse Events

Any AE (solicited or unsolicited) that meets SAE criteria (Section 7.4.1.1.2) must be reported to the CRO immediately (i.e., within 24 hours) after site personnel first learn of the event. Reporting instructions for SAE reports, including the fax number and/or email address for submitting paper forms, can be found in the investigator site file for the study.

7.4.1.3.2 Regulatory Reporting Requirements for SAEs Including Suspected Unexpected Serious Adverse Reactions

The Sponsor is required to notify national regulatory agencies and (in conjunction with the investigator) local regulatory authorities about the safety of a study vaccine under clinical investigation. The Sponsor will comply with country-specific regulatory requirements relating to safety reporting to the regulatory authority, institutional review board (IRB)/Independent Ethics Committee (IEC), and investigators. The Sponsor or designee will promptly evaluate all SUSARs against cumulative product experience to identify and expeditiously communicate possible new safety findings to investigators, applicable IRBs/IECs, and health authorities based on applicable legislation.

To determine reporting requirements for SUSAR cases, the Sponsor or designee will assess the expectedness of these events using the reference safety information contained in the IB.

An investigator who receives an investigator safety report describing an SAE or other specific safety information from the Sponsor or designee will review and then file it as appropriate and will notify the IRB/IEC, if appropriate according to local requirements.

7.4.1.3.3 Follow-up of Participants Reporting Adverse Events

All AEs must be reported in detail on the appropriate page in the eCRF and followed to satisfactory resolution, until the investigator deems the event to be chronic or not clinically significant, the event is considered to be stable, or the participant is lost to follow-up.

All confirmed cases of COVID-19 meeting the definition of severe COVID-19 (as defined in Appendix 2) will be shared with the DSMB within 48 hours of confirmation.

7.4.1.3.4 Laboratory Test Abnormalities

For participants in Phase 1, clinically significant abnormal laboratory test results, in the opinion of the Investigator, may constitute or be associated with an AE. Examples of these include abnormal laboratory results that are associated with symptoms or require treatment, e.g., bleeding due to thrombocytopenia, tetany due to hypocalcemia, or cardiac arrhythmias due to hyperkalemia. Whenever possible, the underlying diagnosis should be listed in preference to abnormal laboratory values as AEs. Severity grading of abnormal test results should follow the grading of unsolicited AEs when the event is deemed clinically significant by the Investigator (Section 7.4.1.1.1.2). Clinically significant abnormalities will be monitored by the Investigator until the parameter returns to its baseline value or until agreement is reached between the Investigator and Sponsor Medical Monitor (or designee) that further follow-up is not required. Laboratory abnormalities deemed NCS by the Investigator should not be reported as AEs. Similarly, laboratory abnormalities reported as AEs by the Investigator should not be deemed NCS on the laboratory sheet. Where relevant, safety laboratory assessments will be analyzed by toxicity grade at the time of interim and final analysis.

The Investigator is responsible for reviewing and signing all laboratory reports. The signed clinical laboratory reports will serve as source documents and should include the Investigator's assessment of clinical significance of out of range/abnormal laboratory values.

#### 7.4.2 Physical Examinations

Any clinically significant change in physical examination should be evaluated for underlying cause and the associated AE reported. If no known cause is determined, the clinically significantly abnormal parameter may be reported as an AE.

#### 7.4.3 Vital Signs, Body Temperature, and Pulse Oximetry

Vital signs, body temperature, and pulse oximetry will be evaluated for clinical significance. Any clinically significant change in vital signs, body temperature or pulse oximetry should be evaluated for underlying cause and the associated AE reported. If no known cause is determined, the clinically significantly abnormal parameter may be reported as an AE.

### 7.5 Pregnancy

If a participant becomes pregnant or a pregnancy is suspected, or if a male participant believes his sexual partner has become pregnant during the study participation, the study center staff must be informed immediately. The pregnancy will be reported using a paper Pregnancy Notification Form and collected in the safety database. The Pregnancy Notification Form should be faxed or emailed to the Sponsor or designee. Follow-up information, including delivery or termination, should be reported within 24 hours. Pregnancy reporting instructions, including the fax number and email address for submitting paper forms can be found in the investigator site file for the study.

Female participants: If a suspected pregnancy occurs while on the study in a female participant who has received study vaccine, a pregnancy test will be performed. If the pregnancy test is positive, the participant will be encouraged to complete all study procedures. Regardless of continued study participation, the study physician will assist the participant in getting obstetrical care and the progress of the pregnancy will be followed until the outcome of the pregnancy is known (ie, delivery, elective termination, or spontaneous abortion). The vaccine assignment of the participant may be unblinded according to the judgment of the investigator, provided unblinding informs a decision relating to pursuit of additional care or vaccination. If the pregnancy results in the birth of a child, the Study Center and Sponsor may require access to the mother’s and infant’s medical records to obtain additional information relevant to the pregnancy progress and outcome. A longer follow-up may be required if a newborn child experiences a medical condition. Follow-up will be performed to the extent permitted by the applicable regulations and privacy considerations, for example, a pregnancy ICF may be required.

Male participants: If the female partner of a male participant who has received study vaccine becomes pregnant, the progress of the pregnancy of the male participant’s partner should be followed until the outcome of the pregnancy is known (ie, delivery, elective termination, or spontaneous abortion). If the pregnancy results in the birth of a child, the Study Center and Sponsor may follow-up with the mother and may request access to the mother and infant’s medical records to obtain additional information relevant to the pregnancy progress and outcome. A longer follow-up may be required if a newborn child experiences a medical condition. Follow-up will be performed to the extent permitted by the applicable regulations and privacy considerations, for example, partner ICF may be required.

# 8 STATISTICAL CONSIDERATIONS

This section summarizes the planned statistical analysis strategy and procedures for the study. The details of statistical analysis will be provided in the SAP, which will be finalized before database lock for each study analysis. If after the study has begun, but prior to any unblinding, material changes are made to primary and/or key secondary objectives/hypotheses, or the statistical methods related to those hypotheses, then the protocol will be amended (consistent with International Council for Harmonisation [ICH] Guideline E9). Changes to other secondary or exploratory analyses made after the protocol has been finalized, along with an explanation as to when and why they occurred, will be listed in the SAP or clinical study report (CSR) for the study. Ad hoc exploratory analyses, if any, will be clearly identified in the CSR.

The design of the study is structured to maintain the integrity of the safety, efficacy and immunogenicity noninferiority endpoints for Phase 3b and 3c such that these data remain blinded to the blinded Sponsor employees and CRO until the analysis of time points for these endpoints whilst earlier unblinding of Phase 1/2/3a participants for analysis of Phase 1/2/3a immunogenicity and safety may occur earlier to inform filing for EUA.

## 8.1 Overall Endpoint Testing Strategy

The overall primary efficacy objective for the study is determination of vaccine efficacy (VE) as evaluated in the Phase 3b Modified Intent to Treat (mITT) population (Section 8.2). Hence the study will be regarded to have met the primary objective if the null hypothesis, as described in Section 8.4.1, is rejected. Hence the primary efficacy objective will be met, and the study overall declared to be positive, if the lower limit of the 95% confidence interval (CI) for VE exceeds 30%. VE is defined as the percent reduction in the hazard of the primary endpoint (ARCT-154 versus placebo).

The overall key secondary objectives for the study are Phase 3b Secondary Endpoints 1 and 2 (Section 3.2.2). These endpoints will be evaluated in a hierarchical fashion such that Pooled Secondary Endpoint 1 will only be evaluated in a hypothesis testing fashion if the overall primary objective of the study has been met and Pooled Secondary Endpoint 2 will only be evaluated in a hypothesis testing fashion if the null hypothesis for Pooled Secondary Endpoint 1 has been rejected.

Phase 3b secondary endpoints 3 and 4 will be evaluated in a hypothesis generating fashion with any statistical significance being nominal.

For the Pooled analyses, endpoints are nominally declared as primary/secondary/exploratory, but all endpoints are evaluated as sensitivity analyses to the equivalent endpoints being assessed in the Phase 3b populations.

Phase 1/2/3a and subgroups thereof constitute a distinct substudy with its own distinct objectives and endpoints. There is no testing procedure for controlling type 1 error for Phase 1/2/3a substudy primary or secondary endpoints. As this is a substudy, and there is no evaluation of efficacy for this substudy, no adjustment of type 1 error is applied to the overall efficacy primary endpoint

Phase 3c and subgroups thereof constitutes a distinct substudy with its own distinct objectves and endpoints and testing strategy. A sequential/hierarchical testing procedure will be used to control type 1 error rate over the Phase 3c primary immunogenicity noninferiority endpoint and the secondary endpoints such that the Phase 3c secondary immunogenicity endpoints will only be tested if the null hypothesis is rejected for the primary endpoint and Phase 3c secondary endpoints numbers 1 through 5 will be tested sequentially using the same hierarchical test procedure with each endpoint only being tested if the null hypothesis is rejected for the preceding endpoint. As this constitutes a substudy, and efficacy for this study is only evaluated as exploratory, no adjustment of type 1 error is applied to the overall efficacy primary endpoint.

## 8.2 Analysis Sets

The following analysis sets will be used in the statistical analyses:

**Randomized Set:** Includes all participants who are randomly assigned in the study regardless of the participants' vaccination status in the study. Participants will be analyzed according to the vaccine to which the participant was randomly assigned. RS are defined for each Phase of the study as follows:

- Phase 1/2/3a RS: this pools data from Phase 1, 2 and 3a participants
- Phase 3b RS
- Phase 3c RS
- Pooled RS: this pools data from all Phases of the Study

**Intent-to-Treat (ITT) Analysis Set:** Includes all participants who receive any dose of study vaccine (ARCT-154 or placebo or ChAdOx1). Participants will be analyzed according to the vaccine to which the participant was randomly assigned. ITT are defined for each Phase of the study as follows:

- Phase 1 ITT
- Phase 2/3a ITT: this pools data from Phase 2 and 3a participants
- Phase 1/2/3a ITT: this pools data from Phase 1, 2 and 3a participants and will include evaluations up to Day 92 only
- Phase 3b ITT
- Phase 3c ITT
- Pooled ITT: this pools data from all Phases of the Study and will include evaluations up to Day 92 only

**Safety Analysis Set (SAS):** Includes all participants who receive any dose of study vaccine (ARCT-154 or placebo or ChAdOx1). Participants will be analyzed according to the study vaccine received. SAS analysis sets are defined for each Phase of the study as follows:

- Phase 1 SAS
- Phase 2/3a SAS: this pools data from Phase 2 and Phase 3a participants
- Phase 1/2/3a SAS: this pools data from Phase 1, Phase 2 and Phase 3a participants (up to Day 92 only)
- Phase 3b SAS

- Phase 3c SAS
- Pooled SAS: this pools data from Phase 1/2/3a/3b for ARCT-154 and placebo, respectively. It will include evaluations up to Day 92 only

**Reactogenicity Analysis Set (RAS):** Includes all participants who receive any dose of study vaccine (ARCT-154 or placebo or ChAdOx1) and provide at least 1 reactogenicity diary report. Participants will be analyzed according to the vaccine received. Reactogenicity analysis sets are defined for each Phase of the study as follows:

- Phase 1 RAS
- Phase 2/3a RAS: this pools data from Phase 2 and Phase 3a participants
- Phase 1/2/3a RAS: this pools data from Phase 1, Phase 2 and Phase 3a participants (up to pre-dose Day 92 only)
- Phase 3b RAS
- Phase 3c RAS
- Pooled RAS: this pools data from Phase 1/2/3a/3b for ARCT-154 and placebo, respectively. It will include evaluations up to Day 92 only

**Modified Intent-to-Treat (mITT) Analysis Set:** Includes all participants who received all protocol-required doses of study vaccine (ARCT-154 or placebo or ChAdOx1) up to the evaluation timepoint concerned, and who have no evidence of SARS-CoV-2 infection on Day 1 or up to 7 days after the 2$^{nd}$ study vaccination. The mITT analysis set will be analyzed according to vaccine assigned. mITT sets are defined for the following Phases of the study:

- Phase 3b mITT
- Phase 3c mITT
- Pooled mITT: this pools data from Phases 1/2/3a/3b and will include evaluations up to Day 92 only

**Per-Protocol (PP) analysis Set:** Includes all eligible randomized participants who received all protocol-required doses of study vaccine (ARCT-154 or placebo or ChAdOx1) up to the evaluation timepoint concerned and within the protocol predefined window, and who have no major protocol deviations expected to affect efficacy, immunogenicity, safety or reactogenicity assessments as determined by the Sponsor Medical Monitor or designee in a blinded manner. The PP analysis set will be analyzed according to which study vaccine was received, in the event there is a discrepancy. PP analysis sets are defined for the following Phases of the study:

- Phase 1 PP set: this excludes any participant that has evidence of SARS-CoV-2 infection at baseline or prior to the analysis time point concerned (IcEv3) or that receives an off-study COVID-19 vaccine prior to the analysis time point concerned (IcEv2).
- Phase 2/3a PP set: this pools data from Phase 2 and Phase 3a participants and excludes any participant that has evidence of SARS-CoV-2 infection at baseline or prior to the analysis time point concerned (IcEv3) or that receives an off-study COVID-19 vaccine prior to the analysis time point concerned (IcEv2).
- Phase 1/2/3a PP set: this pools data from Phase 1, 2 and Phase 3a participants and excludes any participant that has evidence of SARS-CoV-2 infection at baseline

or prior to the analysis time point concerned (IcEv3) or that receives an off-study COVID-19 vaccine prior to the analysis time point concerned (IcEv2). This set will include evaluations of immunogenicity up to Day 92 only.

- Phase 3b PP set: this excludes any participant with evidence of SARS-CoV-2 infection on Day 1 or up to 7 days after the 2nd study vaccination (IcEv3) or that receives an off-study COVID-19 vaccine prior to Day 92 (IcEv2)
- Phase 3c-1 PP set: this includes just the participants enrolled in the Phase 3c-1 subset of Phase 3c but excludes any participant that has evidence of SARS-CoV-2 infection at baseline or prior to the analysis time point concerned (IcEv3) or that receives an off-study COVID-19 vaccine prior to the analysis time point concerned (IcEv2).
- Phase 3c PP set: this includes all of Phase 3c but excludes any participant that has evidence of SARS-CoV-2 infection at baseline or prior to the analysis time point concerned (IcEv3) or that receives an off-study COVID-19 vaccine prior to the analysis time point concerned (IcEv2).
- Pooled PP set: this pools data from Phase 1/2/3a/3b and excludes any participant with evidence of SARS-CoV-2 infection on Day 1 or up to 7 days after the 2nd study vaccination (IcEv3) or that receives an off-study COVID-19 vaccine prior to Day 92 (IcEv2). This will include evaluations up to Day 92 only.

**Immunogenicity Analysis Set (IAS):** Includes all participants who received all protocol-required doses of study vaccine (ARCT-154 or placebo or ChAdOx1) up to the evaluation timepoint concerned, who have no evidence of prior SARS-CoV-2 infection at Day 1 (IcEv3; Section 8.3.1) and who have at least 1 valid post-vaccination immunogenicity assay result. Data at timepoints following evidence of a SARSCoV2 infection (IcEv3), use of immune-modifying drugs, blood products or immunogloblins (IcEv5) or non-study COVID-19 vaccines (IcEv2), or protocol deviations (eg, time windows for doses and blood draws for the time period summarized) that may impact immunogenicity as determined by the Sponsor Medical Monitor or designee in a blinded manner will be excluded. All participants in the IAS will be analyzed according to which study vaccine was received. IAS are defined for the following Phases of the study:

- Phase 1 IAS
- Phase 2/3a IAS: this pools data from participants in Phase 2 and 3a
- Phase 1/2/3a IAS: this pools data from Phase 1, 2 and 3a participants and will include evaluations of immunogenicity up to Day 92 only
- Phase 3b IAS
- Phase 3c-1 IAS

## 8.3 Statistical Considerations for Phase 3

The overall primary efficacy and safety objectives for the study will be evaluated in the Phase 3b mITT and Phase 3b SAS/RAS, respectively.

Participants in Phases 1, 2 and 3a constitute the Immunogenicity Substudy group and data from these participants will be pooled up to Day 92 for the analysis of safety and immunogenicity for Phase 1/2/3a. A subset of participants in Phase 2 and 3a receive a third (booster) vaccination at Day 92 and their data will be pooled at all immunogenicity assessment timepoints.

Data from Phases 1, 2, 3a and 3b will be pooled up to Day 92 for sensitivity analysis of efficacy and safety (the 'Pooled Analyses').

Unblinding of data from individual cohorts may occur over time as shown in Section 8.9, in order to support regulatory filings in Vietnam. At these time points, unblinded data will only be provided to Sponsor and CRO employees that are designated as unblinded and these employees will have no further active role in the conduct of the study after the point at which they become unblinded. Individual participants, blinded study site staff and Sponsor team members involved in overseeing the conduct of the study will remain blinded until the end of study for all phases of the study.

Estimand 1 in Section 8.3.1 relates to the immunogenicity and immunogenicity noninferiority primary, secondary and exploratory endpoints for the Phase 1/2/3a and Phase 3c substudies. Estimand 2 relates to the primary, secondary and exploratory efficacy endpoints. Estimands 3 and 4 relate to the safety endpoints of Phase 1/2/3a, Phase 3b, Phase 3c and the Pooled Analysis (Phases 1/2/3a/3b) safety endpoints.

The exploratory efficacy endpoints will be descriptive, and 95% CIs will be provided whenever applicable.

### 8.3.1 Estimands and Intercurrent Events for Phase 1/2/3a, Phase 3b and Phase 3c

Possible intercurrent events (IcEvs) are presented in Table 14.

**Table 14 Intercurrent Event Types**

| Label | Intercurrent Event Type |
|---|---|
| IcEv1 (Death not due to COVID-19) | Death due to any cause other than COVID-19 associated. |
| IcEv2 (Non-study COVID-19 vaccine) | Use of non-study COVID-19 vaccine. |
| IcEv3 (Early infection) | Positive RT-PCR test or seropositivity for antinucleocapsid antibody or neutralizing antibody (as assessed by sVNT for Phase 1/2/3a and MNT for Phase 3c) indicating exposure to SARS-CoV-2 prior to vaccination or SARS-CoV-2 infection prior to 7 days (inclusive) after the second study vaccination dose (Day 35).<br>Note: for immunogenicity endpoints, Day 57 is the watershed for early infection. |
| IcEv4 (Study infection) | Develops virologically confirmed SARS-CoV-2 infection, with or without COVID-19 symptoms more than 7 days after second study vaccine dose (≥Day 36). |
| IcEv5 (Confounding On-study Medications) | Receives any of the following concomitant medications/vaccines prior to the time point for which the analysis is being performed:<br>• Live vaccines within 28 days before or after any study vaccination<br>• Systemic (oral, intravenous, intramuscular or subcutaneous) corticosteroids given at any study time point prior to the timepoint at which the analysis is being performed<br>• Blood products or immunoglobulins given at any study time point prior to the timepoint at which the analysis is being performed<br>• Immunosupressive medications, including cytotoxic medication for cancer or autoimmune disease, given at any study time point prior to the timepoint at which the analysis is being performed |

Abbreviations: COVID-19=coronavirus disease 2019; IcEv=intercurrent event; SARS-CoV-2=severe acute respiratory syndrome coronavirus 2

Attributes for the principal safety and immunogenicity estimands for Phase 1/2/3a, 3b and 3c with strategies for IcEvs are presented in the tables below.

**Table 15 Estimands for Phase 1/2/3a Substudy and Phase 3c Substudy Primary, Secondary and Exploratory Immunogenicity Endpoints**

| Estimand Label | Estimand 1 (immunogenicity) |
|---|---|
| Estimand Description | NAb by surrogate virus neutralization test (primary for Phase 1/2/3a), NAb by PRNT50 (secondary for Phase 1 and 2 only), NAb by pseudovirus microneutralization test (MNT; secondary for Phase 3c) and BAb (secondary for Phase 1/2/3a and Phase 3c) responses at defined time points following vaccination (as defined in Sections 3.1.1, 3.1.2 and 3.3.2) with ARCT-154 or placebo. These endpoints will be assessed based on responses at Day 57. International units (i.e., the WHO/NIBSC international reference standard) will be used if available. |

**Table 15 Estimands for Phase 1/2/3a Substudy and Phase 3c Substudy Primary, Secondary and Exploratory Immunogenicity Endpoints**

| | |
|---|---|
| Target Population | Vaccinated participants (as defined by eligibility criteria) in Phases 1, 2, 3a and 3c (Phase 3c-1 participants only). The population includes participants without evidence of COVID-19, SARS-CoV-2 infection, or nucleocapsid antibody positivity on or prior to Day 57. |
| Endpoint | Phase 1/2/3a: NAb and BAb responses as defined in Sections 3.2.1 and 3.2.2<br>Phase 3c: NAb and BAb responses as defined in Section 3.3.2 |
| Treatment Conditions | Phase 1, 2 and 3a: ARCT-154 dose 5 µg or placebo administered on Day 1 and Day 29.<br>Phase 3c: ARCT-154 dose 5 µg or ChAdOx1 administered on Day 1 and Day 29. |
| Population-Level Summary | The following will be assessed<br>• Geometric mean concentrations for NAb and BAb<br>• The geometric mean ratio (ARCT-154 /Placebo and ARCT-154 / ChAdOx1) for NAb and BAb concentrations<br>• Proportion of participants in each study vaccine group that demonstrate seroconversion (as defined by 4-fold increase in antibody concentration [based on international units, if available] from baseline).<br>• Geometric mean fold rise (GMFR) for NAb and BAb |
| Intercurrent Event (IcEv) Strategy | |
| IcEv1 (Death not due to COVID-19) | Hypothetical strategy |
| IcEv2 (Non-study COVID-19 vaccine) | Treatment policy strategy |
| IcEv3 (Early infection) | Principal stratum strategy |
| IcEv4 (Study infection) | Principal stratum strategy |
| IcEv5 (Confounding On-study Medications) | Treatment policy strategy |
| Rationale for Strategies | IcEv 2 and IcEv5 could be potentially confounding to the analysis of the endpoint and result in censoring of the participant for all endpoint assessments after the time of the IcEv treatment policy). IcEv4 and IcEv5 could also be potentially confounding but it is more difficult to determine the precise timing of these events, so a principal stratum strategy is used to exclude these participants from immunogenicity analyses. |

Abbreviations: NAb=neutralizing antibody; BAb=binding antibody; PRNT50=plaque reduction neutralization test at 50% reduction; COVID-19=coronavirus disease 2019; IcEv=intercurrent event; SARS-CoV-2=severe acute respiratory syndrome coronavirus 2

**Table 16 Estimands for Primary, Secondary and Exploratory Efficacy Analyses**

| Estimand Label | Estimand 2 (Vaccine Efficacy) |
|---|---|
| Estimand Description | • The proportion of participants (and 95% confidence interval) with virologically confirmed COVID-19 (Appendix 2) detected after 7 days post the second study vaccine dose.<br>• Proportion of participants (and 95% confidence interval) with virologically confirmed COVID-19 (Appendix 2) detected at any time after first vaccine dose |
| Target Population | Eligible participants who have received both doses of study vaccine in the initial series. The population includes participants without evidence of COVID-19, SARS-CoV-2 infection, or nucleocapsid antibody positivity on or prior to Day 35. |
| Endpoint | Incidence of COVID-19/severe COVID 19/death due to COVID-19 (dependent on the endpoint concerned (Section 3)) detected after 7 days post the second study vaccine dose and based upon virologically confirmed COVID-19 (Appendix 2)<br>Additional endpoints will include proportion of participants with virologically confirmed COVID-19 (Appendix 2) detected at any time after first vaccine dose. |
| Treatment Conditions | Phase 1, 2, 3a and 3b: ARCT-154 dose 5 µg or placebo administered on Day 1 and Day 29.<br>Phase 3c: ARCT-154 dose 5 µg or ChAdOx1 administered on Day 1 and Day 29. |
| Population-Level Summary | The proportion of participants (and 95% confidence interval) with virologically confirmed COVID-19 cases (Appendix 2) detected after 7 days post the second study vaccine dose.<br>Additional exploratory endpoints will include proportion of participants with virologically confirmed COVID-19 (Appendix 2) detected at any time after first vaccine dose. |
| Intercurrent Event (IcEv) Strategy | |
| IcEv1 (Death not due to COVID-19) | Hypothetical strategy |
| IcEv2 (Non-study COVID-19 vaccine) | Hypothetical strategy |
| IcEv3 (Early infection) | Principal stratum strategy |
| IcEv4 (Study infection) | Composite strategy |
| IcEv5 (Confounding On-study Medications) | Hypothetical strategy |
| Rationale for Strategies | Early infection (IcEv3) results in complete exclusion from analyses because prior to Day 36, the vaccine may not have achieved full efficacy. Additional exploratory analyses are performed where participants with early infection occurring after Day 1 but before Day 36 are included. |

**Table 17 Estimands for Primary Safety Analyses for Phase 1/2/3a, Phase 3b, Phase 3c and Pooled Cohorts**

| **Estimand Label** | **Estimand 3 (Safety – Unsolicited AEs/AEs leading to discontinuation or withdrawal/MAAE/SAE)** |
|---|---|
| Estimand Description | Count and percentage of vaccinated participants who would develop unsolicited AEs, AEs leading to discontinuation/withdrawal, MAAEs, and SAEs.<br>These will be evaluated with each dose of study vaccine, ARCT-154 or placebo. |
| Target Population | The population who are enrolled in the study and receive any dose of study vaccine (Phase 1, 2, 3a, 3b, 3c-1, and 3c-2 participants) |
| Endpoint | Occurrence of unsolicited AEs will be analyzed up to 28 days after each study vaccine administration.<br>Occurrence of AEs leading to discontinuation/withdrawal, MAAEs, or SAEs will be analyzed up to Day of Switchover/Further Study Vaccine, receipt of non-study vaccine, early withdrawal, or last on-study assessment visit, whichever comes first.<br>Occurrence of AEs leading to discontinuation/withdrawal, MAAEs and SAEs after the Switchover/Further Study Vaccine will be recorded and summarized for the interval from the switchover/Further Study Vaccine up to Day 394 (Phase 1/2/3a and Phase 3b only).<br>For Phase 1/2/3a and 3b, AEs in participants who do not undergo Switchover/Further Study Vaccine will be recorded for the interval from Day 92 up to Day 394 |
| Treatment Conditions | Phase 1/2/3a and 3b: ARCT-154 dose 5 µg or placebo administered on Day 1, Day 29, or at Switchover/Further Study Vaccine (Day 92 and Day 120)<br>Phase 3c: ARCT-154 dose 5 µg or ChAdOx1 administered on Day 1 and Day 29. |
| Population-Level Summary | Percentage of vaccinated participants who develop each type of AE |
| Intercurrent Event (IcEv) Strategy | |
| IcEv1 (Death not due to COVID-19) | Composite strategy |
| IcEv2 (Non-study vaccine) | Treatment policy strategy |
| IcEv3 (Early infection) | Composite strategy |
| IcEv4 (Study infection) | Composite strategy |
| …IcEv5 (Confounding On-study Medications) | Composite strategy |
| Rationale for Strategies | Events occurring after IcEv2 could contribute spurious safety data so a treatment policy is used for these events and events occurring after this IcEv's will be summarized separately. Sensitivity analyses will be conducted in the ITT population where these events will be included (composite strategy). |

**Table 17 Estimands for Primary Safety Analyses for Phase 1/2/3a, Phase 3b, Phase 3c and Pooled Cohorts**

| **Estimand Label** | **Estimand 4 (Reactogenicity – Solicited AEs)** |
|---|---|
| Estimand Description | Count and percentage of vaccinated participants who would develop solicited AEs.<br>These will be evaluated after vaccination on Day 1 and Day 29, ARCT-154 or placebo. |
| Target Population | Vaccinated adults providing at least 1 Diary assessment, including reported absence of solicited events. |
| Endpoint | Occurrence of solicited AEs:<br>Solicited AEs within 7 days after study vaccination on Day 1 and Day 29, by Toxicity Grade (Appendix 4) |
| Treatment Conditions | Placebo or ARCT-154 at dose 5 µg administered on Day 1 and Day 29. |
| Population-Level Summary | Percentage of vaccinated participants who provide at least 1 safety assessment. |
| Intercurrent Event (IcEv) Strategy | |
| IcEv1 (Death not due to COVID-19) | Composite strategy |
| IcEv2 (Non-study vaccine) | Treatment policy strategy |
| IcEv3 (Early infection) | Treatment policy strategy |
| IcEv4 (Study infection) | Composite strategy |
| …IcEv5 (Confounding Concomitant Medications) | Treatment policy strategy |
| Rationale for Strategies | Events occurring after IcEv2, IcEv3 and IcEv5 could contribute spurious reactogenicity data so a treatment policy is used for these events and events occurring after these IcEv's will be summarized separately. |

Abbreviations: AE=adverse event; COVID-19=coronavirus disease 2019; IcEv=intercurrent event; MAAE=medically attended adverse event; SAE=serious adverse event; SARS-CoV-2=severe acute respiratory syndrome coronavirus 2.

## 8.4 Statistical Hypothesis

### 8.4.1 Phase 1/2/3a Substudy Statistical Hypothesis

For the seroconversion rate primary immunogenicity objective, the null hypothesis ($H0^{Immunogenicity}$) is that the lower bound of the 95% CI for ARCT-154 seroconversion rate ($SC^{154}$) is ≤ seroconversion rate for placebo ($SC^{Placebo}$); i.e., $H0^{Immunogenicity}$: lower bound 95% CI for $SC^{154} \leq SC^{Placebo}$. Hence the Phase 1/2/3a part of the study will be considered to meet the primary immunogenicity objective if the lower bound of the 95%CI for $SC^{154} > SC^{Placebo}$.

There is no hypothesis testing for safety data analysis. Where statistical methods are applied, the emphasis will be on estimation with 95% CIs.

### 8.4.2 Phase 3b Statistical Hypothesis

The overall primary endpoints for the study are evaluated in the Phase 3b population as follows:

- The overall primary efficacy endpoint for the study is vaccine efficacy as evaluated in the Phase 3b Modified Intent to Treat (mITT) population
- The overall primary safety endpoints are those evaluated in the Phase 3b Safety Analysis Set (SAS) and Phase 3b Reactogenicity Analysis Set (RAS)

There is no hypothesis testing for safety data analysis. Where statistical methods are applied, the emphasis will be on estimation with 95% CIs.

The primary statistical analyses for VE will be performed using virologically confirmed COVID-19 cases as adjudicated in a blinded fashion by the Independent Adjudication Committee according to definitions provided in Appendix 2. This analysis of the VE will be based on the Phase 3b mITT analysis set (Section 8.2). Sensitivity analyses will be performed in the Phase 3b PP set.

The null hypothesis ($H0^{Efficacy}$) as follows: $H0^{Efficacy}$: VE ≤ 30%

VE is estimated as 1-HR, where HR is the hazard ratio, and will be presented together with 95% CIs. The primary efficacy objective will be met if the lower limit of the 95% CI for VE exceeds 30%.

Under the assumptions of proportional hazards over time and a 50% reduction in hazard rate (50% VE) and with 1:1 randomization of ARCT-154 and placebo, a total of 372 confirmed COVID-19 cases will provide approximately 90% power to reject the null hypothesis (H0: VE ≤ 30%), using a log-rank test statistic with a 1-sided false positive error rate of 0.025.

### 8.4.3 Phase 3c Substudy Statistical Hypotheses

There is no hypothesis testing for primary Phase 3c safety objective. Where statistical methods are applied, the emphasis will be on estimation with 95% CIs.

For the primary Phase 3c immunogenicity noninferiority objective the null hypothesis is as follows:

$H0^{GMR}$: R ≤ NIM

Where R is the ratio of GMC (i.e., GMC for ARCT-154 arm / GMC for the ChAdOx1 arm) and NIM is the acceptable noninferiority margin (0.67). The null hypothesis will be rejected if the lower bound of the 95% confidence interval for GMR is > 0.67.

A sequential/hierarchical testing procedure will be used to control type 1 error rate over the Phase 3c primary immunogenicity noninferiority endpoint and the secondary endpoints such that the Phase 3c secondary immunogenicity endpoints will only be tested if the null hypothesis is rejected for the primary endpoint and Phase 3c secondary endpoints numbers 1 through 5 will be tested sequentially using the same hierarchical test procedure with each endpoint only being tested if the null hypothesis is rejected for the preceding endpoint.

For secondary endpoints based on the ratio of GMC (i.e., GMR; secondary endpoints 1, 4, 5):

- The null hypothesis for noninferiority and the grounds for rejecting the null hypothesis is as stated for the primary Phase 3c endpoint.
- The null hypothesis for superiority of ARCT-154 to ChAdOx1 arm (ie, GMC of ARCT-154 statistically significantly higher than ChAdOx1) is as follows:

  $H0^{GMR}$: R ≤ 1

  The null hypothesis will be rejected if the lower bound of the 95% confidence interval for GMR is > 1.0

For secondary endpoints based on seroconversion rate (Secondary endpoints 2 and 3):

- The null hypothesis for noninferiority of the ARCT-154 arm to the ChAdOx1 arm ($H0^{Seroconversion}$) is as follows:

  $H0^{Seroconversion}: SC^{154} - SC^{ChAdOx1} \leq D0$

  Where $SC^{154}$ is the proportion seroconverting in the ARCT-154 group, $SC^{ChAdOx1}$ is the proportion seroconverting in the ChAdOx1 group and D0 is the acceptable noninferiority margin (-10.0%). Seroconversion is defined as: 4-fold increase in concentration from baseline. The 95% confidence interval of the difference between two proportions will be calculated using the method of Miettinen-Nurminen (Miettinen 1985). International units (i.e., the WHO/NIBSC international reference standard) will be used if available.

  The null hypothesis will be rejected if the lower bound of the 95% confidence interval (CI) of the difference between neutralizing antibody seroconversion rates (ARCT-154 group minus ChAdOx1 group) excludes the non-inferiority margin of -10%.

#### 8.4.4 Pooled Analyses Statistical Hypotheses

Pooled analyses are performed as sensitivity analyses to the Phase 3b safety and efficacy primary endpoints. As such, Pooled analyses of efficacy and safety are nominally declared as primary, secondary, and exploratory to align with the equivalent endpoints in the Phase 3b population, but any statistical analysis performed will be nominal. The pooled endpoints will be evaluated in the same fashion as the equivalent analyses in the Phase 3b populations.

### 8.5 Sample Size Determination

The sample size for the study overall is driven by the sum of the sample size assumptions for the individual phases of the study, as outlined in the subsections below.

#### 8.5.1 Sample Size for Phase 1/2/3a Substudy

Data from Phases 1, 2 and 3a will be pooled up to Day 92 for safety analyses, which will be performed in the Phase 1/2/3a SAS and Phase 1/2/3a RAS (Section 8.2). The total sample size for Phase 1/2/3a is primarily driven by the size of safety database required at the time of potential EUA application. With approximately 750 participants randomized in Phases 1, 2 and 3a to receive ARCT-154 for the Phase 1/2/3a primary safety analysis, if the incidence rate of an adverse event is 1.0%, the probability to detect one event in 750 vaccinated participants is >99%, based upon the following formula:

$$p = 1 - (1 - R)^{\mathrm{N}}$$

where R = incidence rate and N = sample size

Data from Phases 1, 2 and 3a will be pooled up to Day 92 for immunogenicity analyses, which will be performed in the Phase 1/2/3a IAS (Section 8.2).The primary immunogenicity endpoint is defined as the proportion of participants in each study vaccine group that demonstrate seroconversion (defined as: 4-fold increase in titer from baseline) by surrogate virus NAb assay at Day 57. Sensitivity analyses will be performed in the Phase 1/2/3a per protocol (PP) set.

The null hypothesis is $H0^{\mathrm{Immunogenicity}}$: lower bound 95% CI for $SC^{154} \leq SC^{\mathrm{Placebo}}$. A sample size of 750 and 250 participants in the ARCT-154 and placebo groups respectively will provide greater than 90% power to exclude the null hypothesis with a 1-sided type 1 error rate of 0.025 assuming that 10% of participants are excluded from the analysis (for example due to baseline seropositivity), and that $SC^{154}$ >50% and $SC^{\mathrm{Placebo}} \leq 10\%$.

#### 8.5.2 Sample Size for Phase 3b

The primary safety endpoints will be evaluated in the Phase 3b SAS and the Phase 3b RAS (Section 8.2). With a sample size of ~16,000 participants randomized and with approximately 8,000 participants randomized in the study to receive ARCT-154 for the primary safety analysis, if the incidence rate of an adverse event is 0.1%, the probability to detect one event in 8,000 vaccinated participants is >99.9%, based upon the following formula:

$$p = 1 - (1 - R)^{\mathrm{N}}$$

where R = incidence rate and N = sample size

For the overall primary efficacy objective of the study, the null hypothesis is that the VE of ARCT-154 to prevent first occurrence of polymerase chain reaction (PCR)-confirmed COVID-19 is ≤30% (ie, $H0^{efficacy}$: VE ≤0.3).

The primary efficacy objective will be met if the lower limit of the 95% CI for VE exceeds 30%.

VE is defined as the percent reduction in the hazard of the primary endpoint (ARCT-154 versus placebo). Equivalently, the null hypothesis is as follows:

- $H0^{efficacy}$: HR >0.7 (equivalently, proportional hazards VE ≤0.3)

A Cox proportional hazard model will be used to assess the magnitude of the study group difference (ie, HR) between ARCT-154 and placebo at a 1-sided 0.025 significance level. Data will be presented overall and by the stratification factors described in Section 8.6.

Under the assumption of proportional hazards over time and with 1:1 randomization of ARCT-154 and placebo, a total of 372 COVID-19 cases will provide approximately 90% power to detect a 50% reduction in hazard rate (50% VE), rejecting the null hypothesis with a 1-sided false positive error rate of 0.025.

The study will be considered positive at the primary analysis if the one-sided p-value for rejecting HR ≥ 0.7 is less than or equal to 0.025.

### 8.5.3 Sample Size for Phase 3c Substudy

Immunogenicity endpoints will be evaluated in participants in the Phase 3c-1 IAS (Section 8.2). Sensitivity analyses will be performed in the Phase 3c PP set. For the primary immunogenicity noninferiority endpoint of neutralizing antibody (using the pseudovirus microneutralization assay [MNT]) geometric mean ratio (GMR), assuming a GMR of ≥0.9 and a coefficient of variation of variation of 4.6, a sample size of 800 participants in Phase 3c-1will provide greater than 90% power to exclude a non-inferiority boundary of 0.67 based on the use of a one-sided test at the alpha=0.025 level of significance, accounting for a dropout rate of approximately 20-25% due to the national COVID-19 vaccination campaign in Vietnam.

The primary safety endpoints of Phase 3c will be conducted in the Phase 3c Safety Analysis Set and Phase 3c Reactogenicity Analysis Set (Section 8.2). With a sample size of approximately 2,400 participants randomized and with approximately 1,200 participants randomized in the study to receive ARCT-154 or ChAdOx1 for the safety analysis, based upon the following formula, if the incidence rate of an adverse event is 0.1%, the probability to detect one event in 1,200 vaccinated participants is 69.9% and if the incidence rate is 1.0% the probability is more than 99%:

$$p = 1 - (1 - R)^{N}$$

where R = incidence rate and N = sample size

## 8.6 Description of Additional Subgroups to Be Analyzed

To determine consistency of immunogenicity across various subgroups in Phase 1/2/3a and Phase 3c, the immunogenicity and the 95% CIs may be estimated using similar methods to the primary analyses for groups stratified by the following classification variables, if feasible:

- Seropositive or seronegative by SARS-CoV-2 nucleocapsid antibody test on or prior to Day 29
- Age groups: ≥18 to <60 years and ≥60 years
- Age and health risk for severe disease: ≥18 to <60 years and "healthy," ≥18 and <60 years and "at risk," and ≥60 years
- Sex (female, male)
- Race/ethnicity
- Receipt of a live non-COVID-19 vaccine within 28 days before or after any study vaccination

These subgroup analyses may also be performed for the exploratory analyses of vaccine efficacy if there are sufficient COVID-19 events to make such subgroup analyses feasible.

Safety may also be evaluated by Age group, Age and health risk for severe disease and Sex.

## 8.7 Statistical Analysis Methodology

Details of the statistical analyses, methods, and data conventions are described in the SAP. Statistical analysis will be performed using SAS® software version 9.4 or later. Continuous variables will be summarized using the mean, standard deviation, median, minimum value, and maximum value. Categorical variables will be summarized using frequency counts and percentages. Data will be listed in data listings.

### 8.7.1 General Considerations

#### 8.7.1.1 General Considerations for Phase 1/2/3a Substudy

For Phase 1/2/3a, immunogenicity data analysis will be considered to meet the primary immunogenicity objective if the lower bound of the 95%CI for $SC^{154} > SC^{Placebo}$. This analysis will be based on the Phase 1/2/3a IAS (Section 8.1). Sensitivity analyses will be performed in the per protocol (PP) set.

Statistical analysis of safety data will be descriptive, and 95% CIs will be provided whenever applicable. It will be based on the Phase 1/2/3a and Phase 2/3a SAS and reactogenicity analysis set (RAS). Sensitivity analysis of unsolicited events will also be performed in the Phase 1/2/3a ITT and Phase 2/3a ITT analysis sets.

#### 8.7.1.2 General Considerations for Phase 3b

Statistical analysis of safety data will be descriptive, and 95% CIs will be provided whenever applicable. It will be based on the Phase 3b safety analysis set (SAS) and Phase 3b reactogenicity analysis set (RAS). Sensitivity analysis of unsolicited events will also be performed in the Phase 3b ITT analysis set and in the Pooled SAS. Sensitivity analysis of solicited events will be performed in the Pooled RAS.

The primary efficacy analysis will be conducted in the Phase 3b mITT and will be considered to meet the primary endpoint if the lower limit of the 95% CI for VE exceeds 30%. Sensitivity analyses will be performed in the Phase 3b per protocol (PP) set and the Pooled mITT.

#### 8.7.1.3 General Considerations for Phase 3c Substudy

Phase 3c-1 immunogenicity noninferiority data will be considered to meet the primary immunogenicity noninferiority endpoint if the lower bound of the 95% confidence interval for the geometric mean ratio of ARCT-154 / ChAdOx1 excludes 0.67. Geometric mean concentrations will be displayed in international units (i.e., using the WHO/NIBSC reference standard) if available.

A sequential/hierarchical testing procedure will be used to control type 1 error rate over the Phase 3c primary endpoint and the secondary endpoints such that the secondary immunogenicity endpoints will only be tested if the null hypothesis is rejected for the primary endpoint and the secondary endpoints numbers 1 through 5 will be tested sequentially using the same hierarchical test procedure with each endpoint only being tested if the null hypothesis is rejected for the preceding endpoint.

### 8.7.2 Overview of Statistical Methods: Estimation of Estimands and Sensitivity Analyses

A summary of the statistical methods and sensitivity analyses is presented in Table 18.

**Table 18 Summary of Statistical Methods, Including Sensitivity Analyses**

| Estimand Label | Estimand Description | Main Estimation | | | Sensitivity Analysis |
|---|---|---|---|---|---|
| | | Analysis Set | Imputation/Data/ Censoring Rules | Analysis Model/Method | |
| Estimand 1 | NAb by surrogate virus neutralization test (primary for Phase 1/2/3a, NAb by PRNT50 and Bab (secondary for Phase 1 and 2 only), NAb by pseudovirus microneutralization test (MNT; secondary for Phase 3c) and BAb (secondary for Phase 1/2/3a and Phase 3c) responses at defined time points following vaccination (as defined in Sections 3.1.1, 3.1.2, and 3.3.2) with ARCT-154 or placebo. The primary immunogenicity endpoint (Phase 1/2/3a) and secondary immunogenicity noninferiority endpoints (Phase 3c) will be measured based on responses at Day 57. | Phase 1/2/3a IAS<br>Phase 3c-1 IAS | For deaths unrelated to COVID-19, censor at date of death. For participants who undergo Switchover/Further Study Vaccine or receive a non-study vaccine, censor at date of Switchover/Further Study Vaccine or non-study vaccination. Participants with early infection (prior to Day 57) are excluded from the analysis. | Proportion of participants in each study vaccine group that demonstrate seroconversion (as defined by 4-fold increase in antibody concentration from baseline). International units (i.e., the WHO/NIBSC international reference standard) will be used if available.)<br>NAb and BAb geometric mean antibody titers/concentrations | Sensitivity: the same analysis will be performed in the Phase 1/2/3a and Phase 3c-1 PP analysis sets. |

**Table 18 Summary of Statistical Methods, Including Sensitivity Analyses**

| Estimand Label | Estimand Description | Main Estimation | | | Sensitivity Analysis |
|---|---|---|---|---|---|
| | | **Analysis Set** | **Imputation/Data/ Censoring Rules** | **Analysis Model/Method** | |
| Estimand 2 | • The proportion of participants (and 95% confidence interval) with virologically confirmed COVID-19 (Appendix 2) detected after 7 days post the second study vaccine dose.<br>• Proportion of participants (and 95% confidence interval) with virologically confirmed COVID-19 detected at any time after first vaccine dose | Phase 3b mITT (primary)<br>Phase 3c mITT (exploratory) | For deaths unrelated to COVID-19, censor at date of death. For participants who undergo Switchover/Further Study Vaccine or receive a non-study COVID-19 vaccine, censor at date of Switchover or non-study vaccination. | The proportion of participants with virologically confirmed COVID-19 (Appendix 2) in each vaccine group will be displayed together with 95% CI. | Sensitivity: the same analysis will be performed in the Pooled mITT and Phase 3b PP analysis sets. |

| | | | | | |
|---|---|---|---|---|---|
| Estimand 3 | Percentage of vaccinated participants who would develop unsolicited AEs, AEs leading to discontinuation/withdrawal, MAAEs, and SAEs within timeframe up to the earlier of Day 92/Switchover/Further Study Vaccine or receipt of non-study COVID-19 vaccine.<br>A treatment policy strategy is used for assessing safety irrespective of a current (or prior) SARS-CoV-2 infection at time of the study vaccination. See Table 17 for full definition. Events occurring before and after Switchover/Further Study Vaccine will be analyzed separately. | Phase 1/2/3a SAS<br>Phase 2/3a SAS<br>Phase 3b SAS<br>Phase 3c SAS<br>Pooled SAS | SARS-CoV-2 infections and death due to COVID-19 (if they meet the AE and time window criteria) are included in the endpoint (composite strategy). | Summaries of number of participants (%) with unsolicited AEs (including summary by severity and relatedness to study vaccine), AEs leading to discontinuation/withdrawal, MAAEs, and SAEs will be presented. The Clopper-Pearson 95% CIs will be presented for the incidence rate of these events. See Section 8.7.3.2 and the SAP for further details. | Sensitivity analyses will also be performed in the<br>Phase 1/2/3a ITT<br>Phase 2/3a ITT<br>Phase 3b ITT<br>Phase 3c ITT<br>Pooled ITT<br><br>Supportive: Analysis will be performed for different time intervals, specifically:<br>All MAAEs, AE/SAEs, AEs leading to discontinuation/ withdrawal up to Day 29, Day 57, Day 92, and Day 394 or Final Visit/Early Termination Visit/receipt of non-study COVID-19 vaccine after the first vaccination will be summarized accordingly.<br>AEs occurring after the Day 92 will be recorded and summarized for the interval Day 92 up to Day 394 (Phase 1/2/3a and Phase 3b only).<br>For Phase 1/2/3a and 3b, AEs in participants that do not receive additional study vaccine at Day 92 will be recorded separately for the interval |

**Table 18 Summary of Statistical Methods, Including Sensitivity Analyses**

| Estimand Label | Estimand Description | Main Estimation | | | Sensitivity Analysis |
|---|---|---|---|---|---|
| | | Analysis Set | Imputation/Data/ Censoring Rules | Analysis Model/Method | |
| | | | | | from Day 92 up to Day 394<br>All AEs including those occurring after receipt of a non-study COVID-19 vaccination will be summarized separately as supportive analyses, if applicable. |
| Estimand 4 | Percentage of vaccinated participants who would provide reactogenicity information within timeframe.<br>A treatment policy strategy is used for assessing safety irrespective of a current (or prior) SARS-CoV-2 infection at time of the study vaccination. See Table 17 for full definition. | Phase 1/2/3a RAS<br>Phase 2/3a RAS<br>Phase 3b RAS<br>Phase 3c RAS<br>Pooled RAS | Events are only recorded for the initial vaccination series. SARS-CoV-2 infections and death due to COVID-19 (if they meet the AE and time window criteria) are included in the endpoint (composite strategy). | Summaries of number of participants (%) with solicited AEs (by toxicity grade) will be presented. The Clopper-Pearson 95% Cis will be presented for the incidence rate of these events. See Section 8.7.3.2 and the SAP for further details. | |

Abbreviations: NAb=neutralizing antibody; Bab=binding antibody; PRNT50=plaque reduction neutralization test at 50% reduction; GMT=geometric mean titer; GMC=geometric mean concentration; AE=adverse event; CI=confidence interval; COVID-19=coronavirus disease 2019; HR=hazard ratio; MAAE=medically attended adverse event; mITT=modified intent-to-treat; PP=per protocol; RAS=reactogenicity analysis set; SAE=serious adverse event; SAP=statistical analysis plan; SARS-CoV-2=severe acute respiratory syndrome coronavirus 2; SAS=statistical analysis set

### 8.7.3 Analysis of Primary Endpoints for Phase 1/2/3a, Phase 3b and Phase 3c

#### 8.7.3.1 Main Estimation of Primary Immunogenicity Endpoint for Phase 1/2/3a Substudy (Estimand 1)

The primary immunogenicity endpoint for Phase 1/2/3a and the primary immunogenicity noninferiority endpoint for Phase 3c-1 will be evaluated in the Phase 1/2/3a IAS and Phase 3c-1 IAS respectively.

- For Phase 1/2/3a it is defined as the proportion of participants in each study vaccine group that demonstrate seroconversion (defined as: 4-fold increase in titer from baseline) by surrogate virus NAb assay at Day 57. The lower bound of the 95% CI for $SC^{154}$ will be used for evaluation of ARCT-154- vs placebo-treated participants (Section 8.3.1).
- For Phase 3c it is defined as the geometric mean ratio (i.e., the ratio of geometric mean concentration for ARCT-154 / ChAdOx1 nCoV-1) by pseudovirus microneutralization test at Day 57.
- International units (i.e., the WHO/NIBSC international reference standard) will be used if available.

Seroconversion is defined as at least 4-fold increase of post-study intervention antibody titers from the baseline titer or from half of the LLOQ if undetectable at baseline. GMT ratio is defined as geometric mean of fold increase of post-study intervention titers over the baseline titers.

Geometric mean concentration (GMC) is the geometric mean of the antibody concentrations of all participants in each treatment arm; geometric mean ratio (GMR) is the ratio of geometric mean concentrations for the two study vaccines being compared, and geometric mean fold rise is the geometric mean of the proportional rise in GMC from baseline. These will be calculated as follows:

$$\text{GMC} = \ln^{-1}\left(\frac{\sum_{i=1}^{n} \ln X}{n}\right)$$, where X: antibody titer

$$\text{GMR} = \frac{GMC_{vaccine\ 1}}{GMC_{vaccine\ 2}}$$

$$\text{GMFR} = \ln^{-1}\left(\frac{\sum_{i=1}^{n} \ln\left(\frac{X2}{X1}\right)}{n}\right)$$, where X2 is the antibody concentration at time point of evaluation and X1 is the antibody concentration at the reference time point (for example baseline)

For SCR, chi-square or Fisher's exact test will be used to perform comparison of the two treatment arms. 95% CI of the SCR will also be presented. Pearson's Chi-square test will be used except for the case of small cell count (less than 5).

Definition and handling of intercurrent events is described in Section 8.3.1.

#### 8.7.3.2 Main Estimation of Primary Safety Endpoint for Phase 1/2/3a, 3b, and 3c (Estimand 3 & 4)

Summaries of the number of participants (%) with solicited AEs, unsolicited AEs, MAAEs, SAEs, and AEs leading to discontinuation/withdrawal will be presented. Unsolicited events will be presented overall and with a focus on those related to vaccine administration. All summaries will be evaluated by study group.

As summarized in Table 18, the Clopper-Pearson 95% CIs will be presented for the incidence rate of solicited AEs, unsolicited AEs, SAEs, MAAEs, and AEs leading to discontinuation/withdrawal, as well as those that are considered related (possibly, probably, or definitely) to study vaccine.

Solicited AEs will be analyzed in the RAS and unsolicited AEs, MAAEs, AE/SAEs will be summarized in the SAS relevant to the Phase of the study being evaluated (Section 8.2).

#### 8.7.3.3 Supportive Analysis of Primary Safety Endpoints for Phase 1/2/3a, 3b, and 3c (Estimand 3 & 4)

Analysis of safety, as described Section 8.7.3.2 and Table 18 will be performed for a number of different time intervals. Evaluation may also be performed by the stratification factors described in Section 8.6.

The number and percentage of participants with any solicited AEs (local injection site and systemic events) before and after each vaccination will be reported, including summaries by grade (Appendix 4). The number and percentage of participants with unsolicited AEs will be reported, including summaries by severity (mild/moderate/severe). The number and percentage of SAEs and MAAEs will be summarized by incidence. The incidence rate of these events will be presented with 2-sided 95% CIs using the Clopper-Pearson method.

Solicited AE will be analyzed in the RAS and unsolicited AEs, MAAEs, AEs leading to discontinuation/withdrawal, SAEs will be summarized in the SAS relevant to the Phase of the study being evaluated (Section 8.2).

Adverse events after to any non-study vaccination will be analyzed accordingly as a supportive analysis.

#### 8.7.3.4 Main Estimation of Phase 3b Primary Efficacy Endpoint (Estimand 2)

This endpoint is the incidence rate of virologically confirmed COVID-19. The main analysis on this endpoint will be carried out in the Phase 3b mITT and the sensitivity analysis will be performed in the Phase 3b PP analysis set. Incidence rate in each arm will be displayed together with 95% CI.

The statistical hypothesis for this analysis is described in Section 8.4.2.

Definition and handling of intercurrent events is described in Section 8.3.1.

### 8.7.4 Analysis of Secondary Endpoints for Phase 1/2/3a, Phase 3b and Phase 3c

#### 8.7.4.1 Main Estimation of Secondary Immunogenicity Endpoints for Phase 1/2/3a and Phase 3c Substudies

Secondary endpoints will be evaluated in participants in Phases 1, 2 and 3a (the Immunogenicity Subgroup) and Phase 3c-1 (the Immunogenicity Noninferiority Subgroup) who have no evidence of early infection (Section 8.3.1) and have received two doses of study vaccine (ARCT-154 or placebo for Phase 1/2/3a; ARCT-154 or ChAdOx1 for Phase 3c) in the initial vaccination series. International units (i.e., the WHO/NIBSC international reference standard) will be used if available, and comparisons will be based on lower bound of the 95% CI.

Definition and handling of intercurrent events is described in Section 8.3.1.

#### 8.7.4.2 Main Estimation of Secondary Efficacy Endpoints for Phase 3b

These will be evaluated in the Phase 3b mITT using the same testing methods as for the Phase 3b primary efficacy endpoint

### 8.7.5 Analysis of Pooled Efficacy and Safety Endpoints

The pooled safety primary endpoints include the number of participants (%) with solicited AEs, unsolicited AEs, MAAEs, SAEs, and AEs leading to discontinuation/withdrawal and will be evaluated in the Pooled SAS and the Pooled RAS.

The pooled efficacy primary endpoint is the incidence rate of virologically confirmed COVID-19. This analysis will be conducted as a sensitivity analysis to the Phase 3b primary endpoint for the study and will be carried out in the Pooled mITT.

The analysis will be conducted using the same methods as described for the Phase 3b primary efficacy analysis (Section 8.7.3.4) and Phase 3b primary safety analysis (Section 8.7.3.2).

Definition and handling of intercurrent events is described in Section 8.3.1.

### 8.7.6 Analyses of Other Exploratory Endpoints

The analysis of other exploratory endpoints will be described in the SAP.

### 8.7.7 Other Analyses

Summary statistical analyses will be provided for demographics, medical history, physical examination, social history, and risk factor variables at baseline, as well as vital signs, clinical laboratory tests, etc.

### 8.7.8 Analyses of Immunogenicity, Safety and Efficacy Data Post Day 92 (Phase 1//2/3a, Phase 3b and Pooled Cohorts)

Participants in Phase 1/2/3a and Phase 3b receive additional study drug (after the initial two-dose vaccination series) at Day 92 (Section 4.5.2). Immunogenicity data before and after Day 92 will be presented separately for these participants.

All safety data collected post Day 92 will be presented separately, using the same methods described in Estimand 2 or 3. Safety data collected post Day 92 include solicited AEs (Phase 2/3a participants only), unsolicited AEs, MAAEs, AEs leading to discontinuation/withdrawal, and SAEs.

Efficacy data after Day 92 will only be collected for Phase 3c participants and will be presented up to Day 394. Rate and 95% CI of the confirmed infection will be calculated using Poisson distribution.

## 8.8 Handling of Missing Data

Details regarding handling of missing Diary data for the evaluation of solicited AEs and missing immunogenicity assessments will be described in the SAP.

## 8.9 Primary and Interim Analyses Timepoints

### 8.9.1 Phase 1/2/3a Substudy

The primary immunogenicity analysis for Phase 1/2/3a will be performed after all participants in this cohort reach the Day 57 timepoint.

The primary Phase 1/2/3a analysis of safety will be performed when all participants in this cohort have reached Day 92. Subsequent analyses of safety will be exploratory.

Interim analysis of safety data through at least Day 57 may be performed for Phase 1/2/3a at the time of analysis of the primary immunogenicity endpoint of Phase 1/2/3a to support application for emergency use authorization. Participants that are further randomized to receive ARCT-154 or placebo at Day 92 may be unblinded again at Day 120 for evaluation of safety and immunogenicity following the third dose. Sponsor and CRO staff responsible for day-to-day conduct of the study will remain blinded until the final unblinding of data at the end of study occurs in order that safety assessments can continue in an unbiased fashion.

### 8.9.2 Phase 3b

The primary Phase 3b analysis of safety and efficacy will be performed when all participants in Phase 3b that are evaluable for the primary efficacy analysis have reached Day 92 and all potential COVID-19 events in these participants up to Day 92 have been adjudicated by the blinded Independent Adjudication Committee.

Subsequent analyses of safety will be exploratory. However, Sponsor and CRO staff responsible for day-to-day conduct of the study will remain blinded until the final unblinding of data at the end of study occurs in order that safety assessments can continue in an unbiased fashion.

### 8.9.3 Phase 3c Substudy

The primary Phase 3c analysis of safety will be performed when all participants have reached the last study visit (Day 394).

The primary immunogenicity non-inferiority analysis for Phase 3c-1 will be performed after all participants in this cohort reach the Day 57 timepoint.

Interim analysis of safety data through at least Day 57 may be performed for Phase 3c at the time of analysis of the primary immunogenicity non-inferiority endpoint of Phase 3c-1 to support regulatory submission.

## 8.10 Monitoring Committees

### 8.10.1 Blinded Safety Review Committee

A blinded Safety Review Committee (SRC) composed of the medical monitoring team from the Sponsor and the CRO will perform ongoing medical review of the blinded safety data of enrolled participants throughout the study.

The SRC will review all available blinded safety data for the 100 participants in Phase 1 through at least 7 days post-second vaccination (Day 36) and, if study vaccination is judged to be adequately tolerated, the study will initiate simultaneous enrollment of both Phase 2 and Phase 3a. After completion of enrollment of Phases 2 and 3a, the SRC will review all available blinded safety data through at least 7 days (Day 8) post-first vaccination and, if study vaccination is judged to be adequately tolerated, the study will initiate enrollment of Phase 3b. The second dose of study vaccine will not be administered to participants in Phase 3b until the SRC has reviewed the Phase 2 and Phase 3a safety data through 7 days post-second vaccination (Day 36) and judged that study vaccine is adequately tolerated. This committee may request an ad hoc meeting of the DSMB in the event of safety concerns.

### 8.10.2 Blinded Independent Adjudication Committee

A blinded independent AC will be assembled for the purpose of reviewing potential cases of COVID-19 to determine if the criteria for the primary and secondary endpoints have been met. The AC will remain blinded to treatment assignment. The AC composition, its remit, and frequency of data review will be further defined in a charter.

### 8.10.3 Unblinded Independent Data and Safety Monitoring Board

An independent DSMB, which will be guided by a signed charter, will perform ongoing review of blinded and unblinded data, including both safety and confirmed cases of COVID-19 at scheduled data review meetings.

At each analysis, the DSMB will review the available data and make recommendations to the Sponsor to continue, modify, or discontinue study enrollment (if the study is enrolling). In addition to the ongoing review of safety data, the DSMB will also review available severe COVID-19 case data to determine the risk of VAERD. Prespecified harm rules will determine if stopping boundaries for risk of VAERD have been exceeded. The DSMB will be constituted prior to commencement of enrollment of Phase 3b.

# 9 DATA QUALITY ASSURANCE

This study will be conducted according to the ICH E6(R2) risk and quality processes described in the applicable procedural documents. The quality management approach to be implemented in this study will be documented and will comply with the current ICH guidance on quality and risk management (ICH Q9). The Sponsor assumes accountability for actions delegated to other individuals (eg, CROs).

## 9.1 Data Management

As part of the responsibilities assumed by participating in the study, the investigator agrees to maintain adequate case histories for the participants treated as part of the research under this protocol. The investigator agrees to maintain accurate eCRFs and source documentation as part of the case histories. These source documents may include laboratory reports and hospital records.

All CRF information is to be filled in. If an item is not available or is not applicable, this fact should be indicated. Blank spaces should not be present unless otherwise directed.

For eCRFs, investigative site personnel will enter participant data into the electronic data capture (EDC) system. The analysis data sets will be a combination of these data and data from other sources (eg, laboratory data).

Clinical data management will be performed in accordance with applicable CRO standards and data cleaning procedures to ensure the integrity of the data (eg, removing errors and inconsistencies in the data). AE terms will be coded using MedDRA, an internal validated medical dictionary, and concomitant medications/vaccines will be coded using the WHO Drug Dictionary.

## 9.2 Data Disclosure

The Sponsor or designee will publicly disclose clinical study results through posting the results of studies on www.clinicaltrials.gov (ClinicalTrials.gov 2020) and other public registries in accordance with applicable local laws/regulations.

In all cases, study results will be reported in an objective, accurate, balanced, and complete manner and are reported regardless of the outcome of the study or the country in which the study was conducted.

# 10 ETHICS

## 10.1 Independent Ethics Committee or Institutional Review Board

Federal regulations and the ICH guidelines require that approval be obtained from an IRB/IEC before human participants participate in research studies. Before study onset, the protocol, informed consent, advertisements to be used for the recruitment of study participants, and any other written information regarding this study to be provided to the participant or the participant's legal guardian must be approved by the IRB/IEC. Documentation of all IRB/IEC approvals and of the IRB/IEC compliance with ICH harmonized tripartite guideline E6(R2): Good Clinical Practice (GCP) will be maintained by the site and will be available for review by the Sponsor or its designee.

All IRB/IEC approvals should be signed by the IRB/IEC chairman or designee and must identify the IRB/IEC name and address, the clinical protocol by title or protocol number or both, and the date approval or a favorable opinion was granted.

The investigator is responsible for providing written summaries of the progress and status of the study at intervals not exceeding 1 year or otherwise specified by the IRB/IEC. The investigator must promptly supply the Sponsor or its designee, the IRB/IEC, and, where applicable, the institution, with written reports on any changes significantly affecting the conduct of the study or increasing the risk to participants.

## 10.2 Ethical Conduct of the Study

The study will be performed in accordance with the ethical principles that originated from the Declaration of Helsinki, ICH GCP, and all applicable regulations.

## 10.3 Participant Information and Consent

A written informed consent in compliance with regulatory authority regulations or US Title 21 Code of Federal Regulations (CFR) Part 50 shall be obtained from each participant before entering the study or performing any unusual or nonroutine procedure that involves risk to the participant. An informed consent template may be provided by the Sponsor to investigative sites. If any institution-specific modifications to study-related procedures are proposed or made by the site, the consent should be reviewed by the Sponsor or its designee or both before IRB/IEC submission. Once reviewed, the consent will be submitted by the investigator to his or her IRB/IEC for review and approval before the start of the study. If the ICF is revised during the course of the study, all active participants must be reconsented by signing the revised form, if required by IRB/IEC.

Before recruitment and enrollment, each prospective participant or his or her legal guardian will be given a full explanation of the study, allowed to read the approved ICF, and allowed to have any questions answered. Once the investigator is assured that the participant/legal guardian understands the implications of participating in the study, the participant/legal guardian will be asked to give consent to participate in the study by signing the ICF. The authorized person obtaining the informed consent will also sign the ICF.

The ICF will contain a separate section that addresses the use of remaining mandatory samples for optional exploratory research (if applicable) and explain/address the exploratory research portion of the study. Participant medical records need to state that written informed consent was obtained.

The investigator shall retain the signed original ICF(s) and give a copy of the signed original ICF(s) to the participant or legal guardian.

# 11 INVESTIGATOR'S OBLIGATIONS

The following administrative items are meant to guide the investigator in the conduct of the study but may be subject to change based on industry and government standard operating procedures, working practice documents, or guidelines. Changes will be reported to the IRB/IEC but will not result in protocol amendments.

## 11.1 Confidentiality

All laboratory specimens, evaluation forms, reports, and other records will be identified in a manner designed to maintain participant confidentiality. All records will be kept in a secure storage area with limited access. Clinical information will not be released without the written permission of the participant (or the participant's legal guardian), except as necessary for monitoring and auditing by the Sponsor, its designee, the FDA, or the IRB/IEC.

The investigator and all employees and coworkers involved with this study may not disclose or use for any purpose other than performance of the study, any data, record, or other unpublished, confidential information disclosed to those individuals for the purpose of the study. Prior written agreement from the Sponsor or its designee must be obtained for the disclosure of any said confidential information to other parties.

## 11.2 Data Protection

All personal data collected related to participants, investigators, or any person involved in the study, which may be included in the Sponsor's databases, shall be treated in accordance with local data protection law, including laws regarding implementation of technical measures to ensure protection of participant data.

Data collected must be adequate, relevant, and not excessive, in relation to the purposes for which they are collected. Each category of data must be properly justified and in line with the study objective.

Participants' personal data will be stored at the study site in encrypted electronic and/or paper form and will be password protected or secured in a locked room to ensure that only authorized study staff have access. The study site will implement appropriate technical and organizational measures to ensure that the personal data can be recovered in the event of disaster. In the event of a potential personal data breach, the study site will be responsible for determining whether a personal data breach has in fact occurred and, if so, providing breach notifications as required by law.

To protect the rights and freedoms of participants with regard to the processing of personal data, participants will be assigned a single, participant-specific numerical code (participant code). Any participant records or data sets that are transferred to the Sponsor will contain the participant code; participant names **must not** be transferred. All other identifiable data transferred to the Sponsor will be identified by this participant code. The study site will maintain a confidential list of participants who participated in the study, linking each participant code to his or her actual identity and medical record identification. In case of data transfer, the Sponsor will protect the confidentiality of participants' personal data consistent with the clinical study agreement and applicable privacy laws.

### 11.3 Financial Disclosure and Obligations

Investigators and sub-investigators will provide the Sponsor with sufficient, accurate financial information as requested to allow the Sponsor to submit complete and accurate financial certification or disclosure statements to the appropriate regulatory authorities. Investigators are responsible for providing information on financial interests during the course of the study and for 1 year after completion of the study.

Neither the Sponsor nor the CRO is financially responsible for further testing or treatment of any medical condition that may be detected during the Screening process. In addition, in the absence of specific arrangements, neither the Sponsor nor the CRO is financially responsible for further treatment of the participant's disease.

### 11.4 Investigator Documentation

Prior to beginning the study, the investigator will be asked to comply with ICH E6(R2) Section 9.2 by providing all essential documents.

### 11.5 Study Conduct

The investigator agrees that the study will be conducted according to the principles of ICH E6(R2). The investigator will conduct all aspects of this study in accordance with all national, state, and local laws or regulations. Study information from this protocol will be posted on publicly available clinical trial registers before enrollment of participants begins.

### 11.6 Adverse Events and Study Report Requirements

The investigator agrees to submit reports of SAEs to the Sponsor and/or IRB/IEC according to the timeline and method outlined in the protocol. In addition, the investigator agrees to submit reports according to local reporting requirements to the study site IRB/IEC as appropriate.

### 11.7 Investigator's Final Report

Upon completion of the study, the investigator, where applicable, should inform the institution; the investigator/institution should provide the IRB/IEC with a summary of the study's outcome and the Sponsor and regulatory authority(ies) with any reports required.

### 11.8 Records Retention

Essential documents should be retained until at least 2 years after the last approval of a marketing application in an ICH region and until there are no pending or contemplated marketing applications in an ICH region or at least 2 years have elapsed since the formal discontinuation of clinical development of the study vaccine. These documents should be retained for a longer period if required by the applicable regulatory requirements or by an agreement with the Sponsor. It is the responsibility of the Sponsor to inform the investigator/institution as to when these documents no longer need to be retained. No records may be transferred to another location or party without written notification to the Sponsor.

# 12 STUDY MANAGEMENT

The study administrative structure will include an SRC, an independent DSMB, CRO, third party vendors, and laboratories.

## 12.1 Monitoring

### 12.1.1 Monitoring of the Study

This study will be monitored according to an approved monitoring plan based on the objectives, purpose, design, and complexity of the study. Site monitoring is conducted to ensure that the rights of human participants are protected, that the study is implemented in accordance with the protocol and/or other operating procedures, and that the study uses high quality data collection processes. The monitor will evaluate study processes based on ICH E6(R2) and all applicable, regulatory guidelines. The investigator will allocate adequate time for all monitoring visits and between visits to facilitate the requirements of the study and study timelines. The investigator will also ensure that the monitor or other compliance or quality assurance reviewer is given direct access to all study-related documents and study-related facilities (eg, pharmacy and diagnostic laboratory), phone, fax, and internet and has adequate space to conduct the monitoring visit (if the monitoring visit is conducted in person). If the COVID-19 outbreak does not allow direct access to the site by the monitor, the investigator will make every effort to provide access to source documents in a remote fashion. This includes anticipation of medical release forms to be signed by the participant at the time of study enrollment.

### 12.1.2 Inspection of Records

Investigators and institutions involved in the study will permit study-related monitoring, audits, IRB/IEC review, and regulatory inspections by providing direct access to all study records. In the event of an audit, the investigator agrees to allow the Sponsor, representatives of the Sponsor, or a regulatory agency access to all study records.

The investigator should promptly notify the Sponsor and CRO of any audits scheduled by any regulatory authorities and promptly forward copies of any audit reports received to the Sponsor.

## 12.2 Management of Protocol Amendments and Deviations

### 12.2.1 Modification of the Protocol

Any changes in the required procedures defined in this protocol, except those necessary to remove an apparent, immediate hazard to the participant, must be reviewed and approved by the Sponsor or designee. Amendments to the protocol (including emergency changes) must be submitted in writing to the investigator's IRB/IEC, along with any applicable changes to the ICF, for approval before participants can be enrolled into an amended protocol.

### 12.2.2 Protocol Deviations

A deviation from the protocol is an unintended or unanticipated departure from the procedures or processes approved by the Sponsor and the IRB/IEC and agreed to by the investigator. A significant deviation occurs when there is non-adherence to the protocol or to local regulations or

ICH GCP guidelines that may or may not result in a significant, additional risk to the participant or impacts the integrity of study data.

The investigator or designee must document and explain in the participant's source documentation any deviation from the approved protocol. The investigator may implement a deviation from or a change of the protocol to eliminate an immediate hazard/safety risk to study participants without prior IRB/IEC approval. As soon as possible, after such an occurrence, the implemented deviation or change, the reasons for it, and any proposed protocol amendments should be submitted to the IRB/IEC for review and approval and to the Sponsor for agreement and regulatory authorities, where required.

In order to keep deviations from the protocol to a minimum, the investigator and relevant site personnel will be trained in all aspects of study conduct by the Sponsor/Sponsor's representative. This training will occur either as part of the investigator meeting or site initiation. Ongoing training may also be performed throughout the study during routine site monitoring activities.

As this Phase 3 study is planned for execution during a global health emergency (COVID-19 pandemic), there may be local restrictions that prevent or modify the performance of certain study-related procedures (eg, clinic visits and blood sampling). For protocol deviations attributed to COVID-19 interruptions, relevant details leading to the protocol deviation will be captured in the source documents and EDC system in accordance with relevant guidances (for example FDA guidance (FDA 2020a) or any applicable country-specific guidance).

## 12.3 Study Termination

Although the Sponsor has every intention of completing the study, the Sponsor reserves the right to discontinue the study at any time for clinical or administrative reasons.

The EOS is defined as the date on which the last participant completes the last visit (includes Follow-up Visit).

If the study is prematurely terminated or suspended, the Sponsor or Sponsor representative shall promptly inform the investigators, IRBs/IECs, regulatory authorities, and any contract research organization(s) used in the study of the reason(s) for termination or suspension, as specified by the applicable regulatory requirements. The investigator shall promptly inform the participant and should assure appropriate participant therapy and/or follow-up.

## 12.4 Final Report

Whether the study is completed or prematurely terminated, the Sponsor will ensure that the final data are summarized and provided to the regulatory agency(ies) as required by the applicable regulatory requirement(s). The Sponsor will also ensure that any CSRs in marketing applications (as applicable) meet the standards of the ICH harmonized tripartite guideline E3: Structure and content of clinical study reports.

Where required by applicable regulatory requirements, an investigator signatory will be identified for the approval of the CSR. The investigator will be provided reasonable access to statistical tables, figures, and relevant reports and will have the opportunity to review the complete study results.

# 13 REFERENCES

**Auld 2020**

Auld SC, Caridi-Scheible M, Blum JM, et al. ICU and ventilator mortality among critically ill adults with COVID-19. medRxiv. 2020. https://doi.org/10.1101/2020.04.23.20076737.

**Bhimraj 2020**

Bhimraj A, Morgan RL, Shumaker AH, et al. Infectious Diseases Society of America Guidelines on the treatment and management of patients with COVID-19. Clin Infec Dis. 2020. https://www.ncbi.nlm.nih.gov/pmc/articles/PMC7197612/pdf/ciaa478.pdf. Accessed 06 Nov 2020.

**Burki 2020**

Burki TK. The Russian vaccine for COVID-19. 01 Lancet. 2020. https ://doi.org/10.1016/S2213-2600(20)30402-1

**CDC 2017**

Centers for Disease Control and Prevention (CDC). National Health Institute Survey. Glossary of Tobacco Use. https://www.cdc.gov/nchs/nhis/tobacco/tobacco_glossary.htm. Accessed 05 Apr 2021.

**CDC 2020b**

CDC 2020b: Centers for Disease Control and Prevention (CDC). People with Certain Medical Conditions. https://www.cdc.gov/coronavirus/2019-ncov/need-extra-precautions/people-with-medicalconditions.html?CDC_AA_refVal=https%3A%2F%2Fwww.cdc.gov%2Fcoronavirus%2F2019-ncov%2Fneed-extra-precautions%2Fgroups-at-higher-risk.html. Accessed 06 Nov 2020.

**CDC 2021**

CDC 2021 Underlying Medical Conditions Associated with High Risk for Severe COVID-19: Information for Healthcare Providers/Summary of Conditions with Evidence. 13 May 2021. https://www.cdc.gov/coronavirus/2019-ncov/hcp/clinical-care/underlyingconditions.

**ClinicalTrials.gov 2020**

ClinicalTrials.gov. US Library of Medicine. Listed clinical studies related to the coronavirus disease (COVID-19). https://clinicaltrials.gov/ct2/results?cond=COVID-19. Accessed 19 Nov 2020.

**De Alwis 2020**

de Alwis R, Gan ES, Chen S et al. A single dose of self-transcribing and replicating RNA based SARS-CoV-2 vaccine produces protective adaptive immunity in mice. bioRxiv 2020. https://doi.org/10.1101/2020.09.03.280446.

**De Lusignan 2020**

de Lusignan S, Dorward J, Correa A, et al. Risk factors for SARS-CoV-2 among patients in the Oxford Royal College of General Practitioners Research and Surveillance Centre primary care network: a cross-sectional study. Lancet Infect Dis. 2020; 20(9):1034-1042. https://doi.org/10.1016/S1473-3099(20)30371-6.

**DHHS 2007**

Department of Health and Human Services (DHHS), Food and Drug Administration, Center for Biologics Evaluation and Research (CBER) (US). Guidance for industry: Toxicity grading scale for healthy volunteers enrolled in preventative vaccine clinical trials. 2007. http://www.fda.gov/downloads/BiologicsBloodVaccines/GuidanceComplianceRegulatoryInformation/Guidances/Vaccines/ucm091977.pdf. Accessed 09 Nov 2020.

**DHHS 2020**

Department of Health and Human Services (DHHS), Food and Drug Administration, Center for Biologics Evaluation and Research (US). Guidance Document. Development and Licensure of Vaccines to Prevent COVID-19. 2020. https://www.fda.gov/regulatory-information/search-fda-guidance-documents/development-and-licensure-vaccines-prevent-covid-19. Accessed 10 Mar 2021.

**Dolgin 1994**

Dolgin M, Association NYH, Fox AC, Gorlin R, Levin RI, New York Heart Association. Criteria Committee. Nomenclature and criteria for diagnosis of diseases of the heart and great vessels. 9th ed. Boston, MA: Lippincott Williams and Wilkins;1994.

**EMA 2020**

European Medicines Agency: Comirnaty EPAR Medicine Overview. Published 23 Dec 2020. https://www.ema.europa.eu/en/medicines/human/EPAR/comirnaty. Accessed 22 Mar 2021.

**EMA 2021a**

European Medicines Agency: Vaxzevria (previously COVID-19 vaccine AstraZeneca) EPAR Medicine Overview. Published 18 Feb 2021. https://www.ema.europa.eu/en/medicines/human/EPAR/covid-19-vaccine-astrazeneca. Accessed 22 Mar 2021.

**EMA 2021b**

European Medicines Agency: COVID-19 Vaccine Janssen EPAR Medicine Overview. Published 11 Mar 2021. https://www.ema.europa.eu/en/medicines/human/EPAR/covid-19-vaccine-janssen. Accessed 22 Mar 2021.

**EMA 2021c**

European Medicines Agency: COVID19 Vaccine Moderna EPAR Medicine Overview. Published 20 January 2021. https://www.ema.europa.eu/en/medicines/human/EPAR/covid-19-vaccine-moderna. Accessed 22 Mar 2021.

**EMA 2021d**

European Medicines Agency. Committee for Human Medicinal Products. Reflection paper on the regulatory requirements for vaccines intended to provide protection against variant strain(s) of SARS-CoV-2. 23 February 2021. EMA/117973/2021.

**FDA 2020a**

Food and Drug Administration (FDA) Guidance on Conduct of Clinical Trials of Medical Products during COVID-19 Public Health Emergency, March 2020. Updated 21 Sep 2020.

**FDA 2020b**

Food and Drug Administration. Moderna COVID-19 vaccine. Published 18 December 2020. https://www.fda.gov/emergency-preparedness-and-response/coronavirus-disease-2019-covid-19/moderna-covid-19-vaccine. Accessed 22 Mar 2021.

**FDA 2020c**

Food and Drug Administration. FDA Pfizer-BioNTech COVID-19 Vaccine. Published 20 Dec 2020. https://www.fda.gov/emergency-preparedness-and-response/coronavirus-disease-2019-covid-19/pfizer-biontech-covid-19-vaccine. Accessed 22 Mar 2021.

**FDA 2020d**

U.S. Department of Health and Human Services, Food and Drug Administration, Center for Biologics Evaluation and Research. Guidance for Industry: Development and Licensure of Vaccines to Prevent COVID-19. June 2020. Accessed May 26, 2021. https://www.fda.gov/media/139638/download.

**FDA 2021a**

Food and Drug Administration. Frequently asked questions on the emergency use authorization for bamlanivimab 2020. Available at: https://www.fda.gov/media/143605/download. Accessed 20 Jan 2021.

**FDA 2021b**

Food and Drug Administration. Frequently asked questions on the emergency use authorization of casirivimab + imdevimab. 2020. Available at: https://www.fda.gov/media/143894/download. Accessed 20 Jan 2021.

**FDA 2021c**

Food and Drug Administration. FDA Issues Emergency Use Authorization for Third COVID-19 Vaccine. Published 27 Feb 2021. https://www.fda.gov/news-events/press-announcements/fda-issues-emergency-use-authorization-third-covid-19-vaccine. Accessed 22 Mar 2021.

**Global Regulatory Partners 2021**

Global Regulatory Partners: ANVISA unanimously approves emergency use of vaccines. Published 18 Jan 2021. https://globalregulatorypartners.com/anvisa-approves-unanimously-emergency-use-of-vaccines. Accessed 22 Mar 2021.

**Grasselli 2020**

Grasselli G, Zangrillo A, Zanella A, et al. Baseline Characteristics and outcomes of 1591 patients infected with SARS-CoV-2 admitted to ICUs of the Lombardy Region, Italy. JAMA. 2020;323(16):1574-1581. https://doi.org/10.1001/jama.2020.5394.

**James 2014**

James PA, Oparil S, Carter BL, et al. 2014 evidence-based guideline for the management of high blood pressure in adults: report from the panel members appointed to the eighth Joint National Committee (JNC 8). JAMA. 2014 :311(5)507-20. https://doi.org/10.1001/jama.2013.284427.

**Kamphuis 2006**

Kamphuis E, Junt T, Waibler Z, et al. Type I interferons directly regulate lymphocyte recirculation and cause transient blood lymphopenia. Blood. 2006;108(10):3253-61. https://doi.org/10.1182/blood-2006-06-027599.

**Li 2020**

Li B, Yang J, Zhao F, et al. Prevalence and impact of cardiovascular metabolic diseases on COVID-19 in China. Clin Res Cardiol. 2020;109(5):531-538. https://doi.org/10.1007/s00392-020-01626-9.

**Liang 2017**

Liang F, Lindgren G, Lin A, et al. Efficient targeting and activation of antigen-presenting cells in vivo after modified mRNA vaccine administration in rhesus macaques. Mol Ther. 2017;25(12):2635-2647. https://doi.org/10.1016/j.ymthe.2017.08.006.

**Lindgren 2017**

Lindgren G, Ols S, Liang F, et al. Induction of robust B cell responses after influenza mRNA vaccination is accompanied by circulating hemagglutinin-specific icOs+ PD-1+ cXcr3+ T follicular helper cells. Front Immunol. 2017;8:1539. https://doi.org/10.3389/fimmu.2017.01539.

**Lindsay 2019**

Lindsay KE, Bhosle SM, Zurla C, et al. Visualization of early events in mRNA vaccine delivery in non-human primates via PET–CT and near-infrared imaging. Nat Biomed Eng. 2019;3(5): 371-380. https://doi.org/10.1038/s41551-019-0378-3.

**Miettinen 1985**

Miettinen OS, Nurminen M., "Comparative analysis of two rates." Statistics in Medicine 4:213-226 (1985)

**Mulligan 2020**

Mulligan MJ, Lyke KE, Kitchin N, et al. Phase I/II study of COVID-19 RNA vaccine BNT162b1 in adults. Nature. 2020;586(7830):589-593. https://doi. Org/10.1038/s41586-020-2639-4.

**NIH 2020**

National Institutes of Health (NIH). Coronavirus Disease 2019 (COVID-19) Treatment Guidelines. https://files.covid19treatmentguidelines.nih.gov/guidelines/covid19treatmentguidelines.pdf. Accessed 06 Nov 2020.

**NIH 2021**

National Institutes of Health. Coronavirus Disease 2019 (COVID-19) Treatment Guidelines. https://files.covid19treatmentguidelines.nih.gov/guidelines/covid19treatmentguidelines.pdf. Accessed 22 Mar 2021.

**Pardi 2018**

Pardi N, Hogan MJ, Naradikian MS, et al. Nucleoside-modified mRNA vaccines induce potent T follicular helper and germinal center B cell responses. J Exp Med. 2018;215(6):1571–1588. Doi: 10.1084/jem.20171450. https://doi.org/10.1084/jem.20171450.

**Pfizer 2021**

Pfizer and BioNTech provide update on omicron variant. Press Release: 08 Dec 2021.

**Phua 2020**

Phua J, Weng L, Ling L, et al. Intensive care management of coronavirus disease 2019 (COVID-19): challenges and recommendations. Lancet Respir Med. 2020;8(5):506-517. https://doi.org/10.1016/S2213-2600(20)30161-2.

**Reuters 2021a**

Reuters: Russia approves its third COVID-19 vaccine, CoviVac. Published 19 Feb 2021: https://www.reuters.com/article/us-health-coronavirus-russia-vaccine-idUSKBN2AK07H. Accessed 22 Mar 2021.

**Reuters 2021b**

Reuters: China approves two more domestic COVID-19 vaccines for public use. Published 25 Feb 2021. https://www.reuters.com/article/us-health-coronavirus-china-vaccine-idUSKBN2AP1MW. Accessed 22 Mar 2021.

**Richardson 2020**

Richardson S, Hirsch JS, Narasimhan M, et al. Presenting Characteristics, comorbidities, and outcomes among 5700 patients hospitalized with COVID-19 in the New York city area. JAMA. 2020:323(20):2052-2059. https://doi.org/10.1001/jama.2020.6775.

**Rupp 2015**

Rupp JC, Sokoloski KJ, Gebhart NN, Hardy RW. Alphavirus RNA synthesis and non-structural protein functions. J Gen Virol. 2015;96(9):2483-2500. https://doi.org/10.1099/jgv.0.000249.

**Sah 2021**

Sah P, Vilches TN, Shoukat A, et al: Quantifying the potential for dominant spread of SARS-CoV-2 variant B.1.351 in the United States. medRxiv. 2021 May 12; doi: 10.1101/2021.05.10.21256996. Preprint.

**Sahin 2020**

Sahin U, Muik A, Derhovanessian E, et al. Concurrent human antibody and TH1 type T-cell responses elicited by a COVID-19 RNA vaccine. medRxiv 2020. https://doi.org/10.1101/2020.07.17.20140533.

**Tortorici 2019**

Tortorici MA and Vessler D. Structural insights into coronavirus entry. Adv Virus Res. 2019;105:93-116. https://doi.org/10.1016/bs.aivir.2019.08.002.

**Turkmenistan Today 2021**

Turkmenistan Today: Turkmenistan registers vaccines for the prevention of infectious diseases. Published 29 Jan 2021. https://tdh.gov.tm/en/post/25911/turkmenistan-registers-vaccines-prevention-infectious-diseases. Accessed 22 Mar 2021.

**VRBPAC 2020**

Vaccines and Related Biological Products Advisory Committee Meeting December 10, 2020. FDA Briefing Document. Pfizer-BioNTech COVID-19 Vaccine. Accessed February 10, 2021. https://www.fda.gov/media/144245/download.

**WashingtonPost 2020**

WashingtonPost: China approves Sinopharm coronavirus vaccine, the country's first for general use. Published 30 Dec 2020. https://www.washingtonpost.com/world/asia_pacific/coronavirus-

vaccine-china-sinopharm/2020/12/30/1cc4be9e-4a62-11eb-97b6-4eb9f72ff46b_story.html. Accessed 22 Mar 2021.

**WHO 2020**

World Health Organization: A minimal common outcome measure set for COVID-19 clinical research. WHO Working Group on the Clinical Characterisation and Management of COVID-19 infection. Lancet Infect Dis. 2020 Aug;20(8):e192-e197. https://doi.org/10.1016/S1473-3099(20)30483-7.

**WHO 2021**

World Health Organisation: Coronavirus disease (COVID-19) Weekly Epidemiological Update and Weekly Operational Update. https://covid19.who.int/. Accessed 22 Mar 2021.

**Williamson 2020**

Williamson E, Walker AJ, et al. OpenSAFELY: factors associated with COVID-19-related hospital death in the linked electronic health records of 17 million adult NHS patients. medRxiv 2020. https://doi.org/10.1101/2020.05.06.20092999.

**Wu 2021**

Wu K, Werner AP, Moliva JI, et al. mRNA-1273 vaccine induces neutralizing antibodies against spike mutants from global SARS-CoV-2 variants. BioRxiv 2021. Doi: https://doi.org/10.1101/2021.01.25.427948.

**Yang 2020**

Yang J, Zheng Y, Gou X, et al. Prevalence of comorbidities and its effects in coronavirus disease 2019 patients: a systematic review and meta-analysis. Int J Infect Dis. 2020;94:91–95. https://doi.org/10.1016/j.ijid.2020.03.017.

**Zaim 2020**

Zaim S, Chongb JH, Sankaranarayanan V, et al. COVID-19 and multi-organ response. Curr Probl Cardiol. 2020;45(8):100618. https://doi.org/10.1016/j.cpcardiol.2020.100618.

**Zhang 2011**

Zhang N, Bevan MJ. CD8(+) T Cells: foot soldiers of the immune system. Immunity. 2011;35(2):161-168. https://doi.org/10.1016/j.immuni.2011.07.010.

**Zhang 2015**

Zhang L, Wang W, Wang S. Effect of vaccine administration modality on immunogenicity and efficacy. Expert Rev Vaccines. 2015;14(11):1509–1523. https://doi.org/10.1586/14760584.2015.1081067.

**Zhang 2020**

Zhang W, Zhaoa Y, Zhanga F, et al. The use of anti-inflammatory drugs in the treatment of people with severe coronavirus disease 2019 (COVID-19): the perspectives of clinical immunologists from China. Immunology. 2020;214:108393. https://doi.org/10.1016/j.clim.2020.108393.

**Zhou 2020**

Zhou F, Yu T, Du R, et al. Clinical course and risk factors for mortality of adult inpatients with COVID-19 in Wuhan, China: a retrospective cohort study. Lancet. 2020;395(10229):1054-1062. https://doi.org/10.1016/S0140-6736(20)30566-3.

**Zhou 2021**

Zhou D, Dejnirattisai W, Supasa P, et al. Evidence of escape of SARS-CoV-2 variant B.1.351 from natural and vaccine-induced sera. Cell. 2021 Apr 29;184(9):2348-2361.e6. doi: 10.1016/j.cell.2021.02.037. Epub 2021 Feb 23. PMID: 33730597; PMCID: PMC7901269.

**Zou 2004**

Zou G. A modified Poisson regression approach to prospective studies with binary data. Am J Epidemiol. 2004 ;159(7) :702-6. https://doi.org/10.1093/aje/kwh090.

**Zou 2021**

Zou J, Xie, X, Fontes-Garfias CR, et al. The effect of SARS-CoV-2 D614G mutation on BNT162b2 vaccine-elicited neutralization. Npj Vaccines 6, 44 (2021). https://doi.org/10.1038/s41541-021-00313-8.

# 14 APPENDICES

# APPENDIX 1: SCHEDULE OF ASSESSMENTS

## INITIAL VACCINATIONS AT DAY 1 AND DAY 29 FOR PHASE 1 PARTICIPANTS

| Visit Name | Screening | Dose 1 | Follow-up Visit | Weekly Study Call | Dose 2 | Follow-up Visit | Weekly Study Call | Follow-up Visit | Weekly Study Call | Unscheduled Visit[b] | ET Visit |
|---|---|---|---|---|---|---|---|---|---|---|---|
| Visit Type[a] | Clinic | Clinic | Clinic | TC | Clinic | Clinic | TC | Clinic | TC | Clinic | Clinic |
| Study Day | -10 to 1 (pre-dose)[c] | 1 | 8 | 15, 22 | 29 | 36 | 43, 50 | 57 | 64, 71, 78, 85, | N/A | N/A |
| Visit Window (days): | 0 | 0 | +2 | +2 | +2 | +2 | ±2 | +2 | ±2 | N/A | N/A |
| Informed consent | X | | | | | | | | | | |
| Weight/height | X | | | | | | | | | | |
| Physical examination[d] | X | X[c] | X | | X | X | | | | X | X |
| Vital signs and body temperature[e] | X | X[c] | X | | X | X | | X | | X | X |
| Pulse oximetry | | | | | | | | | | X | |
| Pregnancy test[f] | X | X[c] | | | X | | | | | | |
| Blood sampling[g] | X | X | X | | X | X | | X | | | X |
| Nasal swab for RT-PCR SARS-CoV-2[h] | X | X | | | | | | | | X | |
| Review of Medical history | X | X[c] | X | | X | X | | X | | X | X |
| Review of inclusion/exclusion criteria | X | | | | | | | | | | |
| Study vaccine administration[i] | | X | | | X | | | | | | |
| Post-vaccination observation[j] | | X | | | X | | | | | | |
| Review for COVID-19 symptoms and risks[k] | X | X[c] | X | X | X | X | X | X | X | X | X |
| Adverse events[l] | X | X[c] | X | X | X | X | X | X | X | X | X |
| Concomitant medication/vaccines collection | X | X[c] | X | X | X | X | X | X | X | X | X |
| Collect paper diary [m] | | | X | | | X | | | | | |

Abbreviations: AE=adverse event; COVID-19=coronavirus disease 2019; ET=early termination; MAAE=medically attended adverse event; N/A=not applicable; RT-PCR=reverse transcription polymerase chain reaction; SAE=serious adverse event; SARS-CoV-2=severe acute respiratory syndrome coronavirus 2; TC=telephone call

a Clinic Visits will be performed in person unless the participant is otherwise directed to remain at home or be seen at a hospital.

b Unscheduled visits include visits for specific safety issues and/or evaluation of possible COVID-19. These visits may be performed in the clinic, by home visit, by hospital visit (if allowed by local policy), or via telemedicine/telephone visit. Required procedures at these visits include AE and concomitant medication collection. For participants evaluated for COVID-19, samples should be collected for SARS-CoV-2 testing. Should the visit occur in person, the visit should also include evaluation of vital signs, body temperature, pulse oximetry, and symptom-directed physical examination.

c Screening procedures may be performed on Day 1, if feasible. All Screening procedures must be performed prior to determination of eligibility and enrollment of the participant in the trial. If all Screening procedures are completed on Day 1, the following Screening assessments do not need to be repeated prior to dosing: physical examination, vital signs, body temperature, pregnancy testing, review of COVID-19 symptoms and risks, and collection of AEs and concomitant medications.

d Complete physical examination will be performed at Screening, Days 1, 8, 29, 36, 92 and 120 (Switchover/Further Study Vaccine vaccinations), and Final Visit (or ET); symptom-directed examination (if any symptoms) may be performed at other timepoints as indicated to assess changes from Screening.

e Blood pressure, heart rate, respiratory rate, and body temperature will be measured.

f Pregnancy testing will be performed on women who are not surgically sterile or postmenopausal. Pregnancy testing by urine dipstick is acceptable. Pregnancy testing will be performed and evaluated prior to each study vaccine administration.

g Screening blood sampling may be performed if warranted to confirm pregnancy (if urine dipstick not permitted), and menopausal status (as clinically relevant). Collection of blood is outlined in Section 4.5.5.3. Phase 1 participants will have additional blood collected for screening and safety laboratory assessments at Screening, Day 8, 29, 36 and 57. These blood samples will be sent to the central laboratory. Screening samples may be processed at the site or at the central laboratory if the test is not available.

h Collection of nasal samples is outlined in Section 4.5.5.3. Use of provided nasal swab test kits (including 2 collection containers) to be sent to the central laboratory is strongly preferred. Approved alternate measures of virological confirmation of SARS-CoV-2 may be performed if use of provided kits is not feasible.

i Study vaccine will be administered by intramuscular injection into the lateral aspect of the deltoid muscle. Injection into the nondominant arm is recommended. Study vaccine will be administered in an observer-blind fashion at Day 1 and Day 29. At Switchover/Further Study Vaccine (if the participant meets eligibility criteria for Switchover/Further Study Vaccine), vaccine will be administered at Day 92 and Day 120.

j Vaccinated participants in Phase 1 will be observed at the site for at least 3 hours following vaccine administration (if performed) and until clinically stable. During the post-vaccination observation period, the participant will undergo assessment of vital signs, body temperature, AEs, and concomitant medications prior to discharge from the clinic

k Review of COVID-19 symptoms and risks will include a scripted interview of the participant for risks of recent exposure to COVID-19 and for symptoms of COVID-19 (Appendix 2). If symptoms or risk of COVID-19 is confirmed, testing for SARS-CoV-2 should be performed. If this is identified during the TC with the participant, an Unscheduled visit should be arranged.

l Review of AEs will include surveillance for Solicited AEs within 7 days of each vaccination for the initial series (collected by eDiary or paper diary). Unsolicited AEs will be collected for 28 days after each vaccination in all participants. SAEs, MAAEs, and AEs leading to discontinuation/withdrawal will be collected throughout study from signing of informed consent in all participants.

m For participants who do not use the eDiary, collect paper diary at Day 8 and 36 Visit. The paper diary must be retained as a source document.

## INITIAL VACCINATIONS AT DAY 1 AND DAY 29 FOR PHASE 2, PHASE 3A AND 3B PARTICIPANTS

| Visit Name | Screening | Dose 1 | Weekly Study Call | Dose 2 | Weekly Study Call | Follow-up Visit | Weekly Study Call | Unscheduled Visit[b] | ET Visit |
|---|---|---|---|---|---|---|---|---|---|
| Visit Type[a] | Clinic | Clinic | TC | Clinic | TC | Clinic | TC | Clinic | Clinic |
| Study Day | -10 to 1 (pre-dose)[c] | 1 | 8, 15, 22 | 29 | 36, 43, 50 | 57 | 64, 71, 78, 85 | N/A | N/A |
| Visit Window (days): | 0 | 0 | +2 | +2 | +2 | +2 | ±2 | N/A | N/A |
| Informed consent | X | | | | | | | | |
| Weight/height | X | | | | | | | | |
| Physical examination[d] | X | X[c] | | X | | | | X | X |
| Vital signs and body temperature[e] | X | X[c] | | X | | X | | X | X |
| Pulse oximetry | | | | | | | | X | |
| Pregnancy test[f] | X | X[c] | | X | | | | | |
| Blood sampling[g] | X | X | | X | | X | | | X |
| Nasal swab for RT-PCR SARS-CoV-2[h] | | X | | | | | | X | |
| Review of Medical history | X | X[c] | | X | | X | | X | X |
| Review of inclusion/exclusion criteria | X | | | | | | | | |
| Determination of "at risk" status[i] | | X | | | | | | | |
| Study vaccine administration[j] | | X | | X | | | | | |
| Post-vaccination observation[k] | | X | | X | | | | | |
| Review for COVID-19 symptoms and risks[l] | X | X[c] | X | X | X | X | X | X | X |
| Adverse events[m] | X | X[c] | X | X | X | X | X | X | X |
| Concomitant medication/vaccines collection | X | X[c] | X | X | X | X | X | X | X |
| Collect paper diary [n] | | | | X | | X | | | |

Abbreviations: AE=adverse event; COVID-19=coronavirus disease 2019; ET=early termination; MAAE=medically attended adverse event; N/A=not applicable; RT-PCR=reverse transcription polymerase chain reaction; SAE=serious adverse event; SARS-CoV-2=severe acute respiratory syndrome coronavirus 2; TC=telephone call

a Clinic Visits will be performed in person unless the participant is otherwise directed to remain at home or be seen at a hospital.

b Unscheduled visits include visits for specific safety issues and/or evaluation of possible COVID-19. These visits may be performed in the clinic, by home visit, by hospital visit (if allowed by local policy), or via telemedicine/telephone visit. Required procedures at these visits include AE and concomitant medication collection. For participants evaluated for COVID-19, samples should be collected for SARS-CoV-2 testing. Should the visit occur in person, the visit should also include evaluation of vital signs, body temperature, pulse oximetry, and symptom-directed physical examination.

c Screening procedures may be performed on Day 1, if feasible. All Screening procedures must be performed prior to determination of eligibility and enrollment of the participant in the trial. If all Screening procedures are completed on Day 1, the following Screening assessments do not need to be repeated prior to dosing: physical examination, vital signs, body temperature, pregnancy testing, review of COVID-19 symptoms and risks, and collection of AEs and concomitant medications.

d Complete physical examination will be performed at Screening, Days 1 and 29, 92 and 120 (Switchover/Further Study Vaccine vaccinations), and Final Visit (or ET); symptom-directed examination (if any symptoms) may be performed at other timepoints as indicated to assess changes from Screening.

e Blood pressure, heart rate, respiratory rate, and body temperature will be measured.

f Pregnancy testing will be performed on women who are not surgically sterile or postmenopausal. Pregnancy testing by urine dipstick is acceptable. Pregnancy testing will be performed and evaluated prior to each study vaccine administration.

g Screening blood sampling may be performed if warranted to confirm pregnancy (if urine dipstick not permitted), and menopausal status (as clinically relevant). Collection of blood is outlined in Section 4.5.5.3. Phase 1, Phase 2 and Phase 3a participants (the Immunogenicity Subgroup) will have blood collected more frequently for assessment of antibody responses to the SARS-CoV-2 spike protein. These blood samples will be sent to the central laboratory. Screening samples may be processed at the site or at the central laboratory if the test is not available.

h Collection of nasal samples is outlined in Section 4.5.5.3. Use of provided nasal swab test kits (including 2 collection containers) to be sent to the central laboratory is strongly preferred. Approved alternate measures of virological confirmation of SARS-CoV-2 may be performed if use of provided kits is not feasible (Section 7.1.1).

i Determination of a participant's health status will be performed prior to randomization only (Day 1, prior to study vaccine administration).

j Study vaccine will be administered by intramuscular injection into the lateral aspect of the deltoid muscle. Injection into the nondominant arm is recommended. Study vaccine will be administered in an observer-blind fashion at Day 1 and Day 29. At Switchover/Further Study Vaccine (if the participant meets eligibility criteria for Switchover/Further Study Vaccine), vaccine will be administered at Day 92 and Day 120.

k Vaccinated participants in Phases 2, 3a and 3b will be observed at the site for at least 30 minutes following vaccine administration (if performed) and until clinically stable. During the post-vaccination observation period, the participant will undergo assessment of vital signs, body temperature, AEs, and concomitant medications prior to discharge from the clinic

l Review of COVID-19 symptoms and risks will include a scripted interview of the participant for risks of recent exposure to COVID-19 and for symptoms of COVID-19 (Appendix 2). If symptoms or risk of COVID-19 is confirmed, testing for SARS-CoV-2 should be performed. If this is identified during the TC with the participant, an Unscheduled visit must be arranged unless clinically unstable.

m Review of AEs will include surveillance for Solicited AEs within 7 days of each vaccination for the initial series (collected by eDiary or paper diary). Unsolicited AEs will be collected for 28 days after each vaccination in all participants. SAEs, MAAEs, and AEs leading to discontinuation/withdrawal will be collected throughout study from signing of informed consent to Final Visit in all participants.

n For Phase 2, 3a and 3b participants who do not use the eDiary, collect paper diary at Day 29 and 57 Visit. The paper diary must be retained as a source document.

# INITIAL VACCINATIONS AT DAY 1 AND DAY 29 FOR PHASE 3C PARTICIPANTS

| Visit Name | Screening | Dose 1 | Weekly Study Call | Dose 2 | Weekly Study Call | Weekly Study Call | Follow-up Visit | Weekly Study Call | Unscheduled Visit[b] | ET Visit |
|---|---|---|---|---|---|---|---|---|---|---|
| Visit Type[a] | Clinic | Clinic | TC | Clinic | TC | TC | Clinic | TC | Clinic | Clinic |
| Study Day | -10 to 1 (pre-dose)[c] | 1 | 8, 15, 22 | 29 | 36 | 50 | 57 | 64, 71, 78, 85 | N/A | N/A |
| Visit Window (days): | 0 | 0 | +2 | +2 | +2 | +2 | +2 | ±2 | N/A | N/A |
| Informed consent | X | | | | | | | | | |
| Weight/height | X | | | | | | | | | |
| Physical examination[d] | X | X[c] | | X | | | | | X | X |
| Vital signs and body temperature[e] | X | X[c] | | X | | | X | | X | X |
| Pulse oximetry | | | | | | | | | X | |
| Pregnancy test[f] | X | X[c] | | X | | | | | | |
| Blood sampling[g] | X | X | | X | | | X | | | X |
| Nasal swab for RT-PCR SARS-CoV-2[h] | | X | | | | | | | X | |
| Review of Medical history | X | X[c] | | X | | | X | | X | X |
| Review of inclusion/exclusion criteria | X | | | | | | | | | |
| Determination of "at risk" status[i] | | X | | | | | | | | |
| Study vaccine administration[j] | | X | | X | | | | | | |
| Post-vaccination observation[k] | | X | | X | | | | | | |
| Review for COVID-19 symptoms and risks[l] | X | X[c] | X | X | X | X | X | X | X | X |
| Adverse events[m] | X | X[c] | X | X | X | X | X | X | X | X |
| Concomitant medication/vaccines collection | X | X[c] | X | X | X | X | X | X | X | X |
| Collect paper diary [n] | | | | X | | | X | | | |

Abbreviations: AE=adverse event; COVID-19=coronavirus disease 2019; ET=early termination; MAAE=medically attended adverse event; N/A=not applicable; RT-PCR=reverse transcription polymerase chain reaction; SAE=serious adverse event; SARS-CoV-2=severe acute respiratory syndrome coronavirus 2; TC=telephone call

a Clinic Visits will be performed in person unless the participant is otherwise directed to remain at home or be seen at a hospital.

b Unscheduled visits include visits for specific safety issues and/or evaluation of possible COVID-19. These visits may be performed in the clinic, by home visit, by hospital visit (if allowed by local policy), or via telemedicine/telephone visit. Required procedures at these visits include AE and concomitant medication collection. For participants evaluated for COVID-19, samples should be collected for SARS-CoV-2 testing. Should the visit occur in person, the visit should also include evaluation of vital signs, body temperature, pulse oximetry, and symptom-directed physical examination.

c Screening procedures may be performed on Day 1, if feasible. All Screening procedures must be performed prior to determination of eligibility and enrollment of the participant in the trial. If all Screening procedures are completed on Day 1, the following Screening assessments do not need to be repeated prior to dosing: physical examination, vital signs, body temperature, pregnancy testing, review of COVID-19 symptoms and risks, and collection of AEs and concomitant medications.

d Complete physical examination will be performed at Screening, Days 1 and 29, and Final Visit (or ET); symptom-directed examination (if any symptoms) may be performed at other timepoints as indicated to assess changes from Screening.

e Blood pressure, heart rate, respiratory rate, and body temperature will be measured.

f Pregnancy testing will be performed on women who are not surgically sterile or postmenopausal. Pregnancy testing by urine dipstick is acceptable. Pregnancy testing will be performed and evaluated prior to each study vaccine administration.

g Screening blood sampling may be performed if warranted to confirm pregnancy (if urine dipstick not permitted), and menopausal status (as clinically relevant). Collection of blood is outlined in Section 4.5.5.3. Collection of blood samples is outlined in Section 4.5.5.3. The first 1,500 participants in Phase 3c (the Immunogenicity Noninferiority Subgroup, Phase 3c-1) will have additional blood collected at Day 211 for assessment of antibody responses to the SARS-CoV-2 spike protein. These blood samples will be sent to the central laboratory. The last ~900 participants enrolled (Phase 3c-2) will have phone visits Day 211.

h Collection of nasal samples is outlined in Section 4.5.5.3. Use of provided nasal swab test kits (including 2 collection containers) to be sent to the central laboratory is strongly preferred. Approved alternate measures of virological confirmation of SARS-CoV-2 may be performed if use of provided kits is not feasible (Section 7.1.1).

i Determination of a participant's health status will be performed prior to randomization only (Day 1, prior to study vaccine administration).

j Study vaccine will be administered by intramuscular injection into the lateral aspect of the deltoid muscle. Injection into the nondominant arm is recommended. Study vaccine will be administered in an observer-blind fashion at Day 1 and Day 29.

k Vaccinated participants in Phases 3c will be observed at the site for at least 30 minutes following vaccine administration (if performed) and until clinically stable. During the post-vaccination observation period, the participant will undergo assessment of vital signs, body temperature, AEs, and concomitant medications prior to discharge from the clinic

l Review of COVID-19 symptoms and risks will include a scripted interview of the participant for risks of recent exposure to COVID-19 and for symptoms of COVID-19 (Appendix 2). If symptoms or risk of COVID-19 is confirmed, testing for SARS-CoV-2 should be performed. If this is identified during the TC with the participant, an Unscheduled visit must be arranged unless clinically unstable.

m Review of AEs will include surveillance for Solicited AEs within 7 days of each vaccination for the initial series (collected by eDiary or paper diary). Unsolicited AEs will be collected for 28 days after each vaccination in all participants. SAEs, MAAEs, and AEs leading to discontinuation/withdrawal will be collected throughout study from signing of informed consent to Final Visit in all participants.

n For Phase 3c participants who do not use the eDiary, collect paper diary at Day 29 and 57 Visit.. The paper diary must be retained as a source document.

## DAY 92 TO END OF STUDY VISIT FOR PHASE 1, PHASE 2, PHASE 3A AND PHASE 3B PARTICIPANTS

| Visit Name | Further Dose 1[n] | Weekly Study Call[o] | Further Dose 2[o] | Weekly Study Call[o] | Monthly Study Call | Final Visit | Unscheduled Visit[b] | ET Visit |
|---|---|---|---|---|---|---|---|---|
| Visit Type[a] | Clinic | TC | Clinic | TC | TC | Clinic | Clinic | Clinic |
| Study Day: | 92 | 99. 106, 113 | 120[o] | 127, 134, 141, 148, 155, 162, 169, 176 | 210, 240, 270, 300, 330 and 360 | 394 | N/A | N/A |
| Visit Window (days): | +30 or +14[n] | +2 | +2 | +2 | ± 2 | +14 | N/A | N/A |
| Physical examination[c] | X | | X | | | X | X | X |
| Vital signs and body temperature[d] | X | | X | | | X | X | X |
| Pulse oximetry | | | | | | | X | |
| Pregnancy test[e] | X | | X | | | | | |
| Blood sampling[f] | X | | X | | | X | | X |
| Nasal swab sample for RT-PCR SARS-CoV-2[g] | | | | | | | X | |
| Review of Medical History | X | | X | | | X | X | X |
| Review of eligibility criteria for Switchover/Further Study Vaccine[h, i] | X | | | | | | | |
| Study vaccine administration[i] | X | | X | | | | | |
| Post-vaccination observation[j] | X | | X | | | | | |
| Review for COVID-19 symptoms and risks[k] | X | X | X | X | X | X | X | X |
| Adverse events[l] | X | X | X | X | X | X | X | X |
| Concomitant medication/vaccines collection | X | X | X | X | X | X | X | X |
| Collect paper diary[m] | | | X | | | | | |

Abbreviations: AE=adverse event; COVID-19=coronavirus disease 2019; ET=early termination; MAAE=medically attended adverse event; N/A=not applicable; RT-PCR=reverse transcription polymerase chain reaction; SAE=serious adverse event; SARS-CoV-2=severe acute respiratory syndrome coronavirus 2; TC=telephone call

a Visits will be performed in person unless the participant is otherwise directed to remain at home or be seen at a hospital.

b Unscheduled visits include visits for specific safety issues and/or evaluation of possible COVID-19. These visits may be performed in the clinic, by home visit, by hospital visit (if allowed by local policy), or via telemedicine/telephone visit. Required procedures at these visits include AE and concomitant medication collection. For participants evaluated for COVID-19, samples should be collected for SARS-CoV-2 testing. Should the visit occur in person, the visit should also include evaluation of vital signs, body temperature, pulse oximetry, and symptom-directed physical examination.

c Complete physical examination will be performed at Switchover/Further Study Vaccine vaccinations on Day 92 and Day 120, and Final Visit (or ET); symptom-directed examination (if any symptoms) may be performed at other timepoints as indicated to assess changes from Screening.

d Blood pressure, heart rate, respiratory rate, and body temperature will be measured.

e Pregnancy testing will be performed on women who are not surgically sterile or postmenopausal. Pregnancy testing by urine dipstick is acceptable. Pregnancy testing will be performed and evaluated prior to each study vaccine administration.

f Collection of blood samples is outlined in Section 4.5.5.3. Phase 1, Phase 2 and Phase 3a participants (the Immunogenicity Subgroup) will have blood collected more frequently for assessment of antibody responses to the SARS-CoV-2 spike protein. These blood samples will be sent to the central laboratory.

g Collection of nasal swab samples is outlined in Section 4.5.5.3. Use of provided nasal swab test kits (including 2 collection containers) to be sent to the central laboratory is strongly preferred. Approved alternate measures of virological confirmation of SARS-CoV-2 may be performed if use of provided kits is not feasible.

h Confirm Switchover/Further Study Vaccine eligibility criteria (Section 5.1.5) prior to Switchover/Further Study Vaccine vaccine administration.

i Study vaccine will be administered by intramuscular injection into the lateral aspect of the deltoid muscle of the nondominant arm. Study vaccine will be administered in an observer-blind fashion at Day 92 and Day 120 (if the participant meets eligibility criteria for Switchover/Further Study Vaccine). For participants who do not meet eligibility criteria for Switchover/Further Study Vaccine, study vaccination will not be performed.

j For Switchover vaccinations, participants in Phase 1 and Phase 3b will be observed for at least 15 minutes and until clinically stable. Participants in Phase 2 and 3a will be observed for at least 30 minutes and until clinically stable for vaccinations 1, 2, and 3 (Day 92) and for at least 15 minutes and until clinically stable for vaccination 4 (Day 120).

k Review of COVID-19 symptoms and risks will include a scripted interview of the participant for risks of recent exposure to COVID-19 and for symptoms of COVID-19 (Appendix 2). If symptoms or risk of COVID-19 is confirmed, testing for SARS-CoV-2 should be performed. If this is identified during the TC with the participant, an Unscheduled visit should be arranged.

l Review of AEs will include surveillance for solicited (only after Day 92 vaccination and only for Phase 2 and 3a) and unsolicited AEs, SAEs, MAAEs, and AEs leading to discontinuation/withdrawal; Solicited AE data will be gathered by Diary for Day 1 through Day 7 for all participants after each of the initial vaccination series only; Unsolicited AE data will be gathered by interview at clinic visits and telephone contacts through Screening and 28 days after each vaccination. SAEs, MAAEs, and AEs leading to discontinuation/withdrawal will be collected at all timepoints after signing of the informed consent.

m Only participants in Phase 2 and 3a complete a diary after vaccination on Day 92; no diary is completed after vaccination on Day 120. Participants in Phase 1 and 3b do not complete a diary after vaccination on either Day 92 or Day 120.

n For participants in Phase 1 and Phase 3b, the visit window at Day 92 is +30 days. For participants in Phase 2 and Phase 3a, the visit window at Day 92 is +14 days.

- Participants ineligible for additional doses (Section 5.1.5) do not have a clinic visit at Day 120 and will not have weekly telephone visits on Day 99 to Day 113 and Days 127 to Day 176 but will have monthly telephone visits instead on Days 120, 150, and 180.

## DAY 92 TO END OF STUDY VISIT FOR PHASE 3C PARTICIPANTS

| Visit Name | Weekly Study Call | Follow-up Visit | Weekly Study Call | Monthly Study Call | Final Visit | Unscheduled Visit [b] | ET Visit |
|---|---|---|---|---|---|---|---|
| Visit Type[a] | TC | Clinic | TC | TC | Clinic | Clinic | Clinic |
| Study Day: | 92, 99, 106, 113, 120, 127, 134, 141, 148, 155, 162, 169, 176, 183, 190, 197, 204 | 211 | 218, 225, 232, 239, 246, 253, 260 | 290, 320, 350 | 394 | N/A | N/A |
| Visit Window (days): | ± 2 | +30 | ± 2 | ± 2 | +14 | N/A | N/A |
| Physical examination[c] | | X | | | X | X | X |
| Vital signs and body temperature[d] | | X | | | X | X | X |
| Pulse oximetry | | | | | | X | |
| Blood sampling [e] | | X | | | X | | X |
| Nasal swab sample for RT-PCR SARS-CoV-2 [f] | | | | | | X | |
| Review of Medical History | | X | | | X | X | X |
| Review for COVID-19 symptoms and risks [g] | X | X | X | X | X | X | X |
| Adverse events [h] | X | X | X | X | X | X | X |
| Concomitant medication/vaccines collection | X | X | X | X | X | X | X |

Abbreviations: AE=adverse event; COVID-19=coronavirus disease 2019; ET=early termination; MAAE=medically attended adverse event; N/A=not applicable; RT-PCR=reverse transcription polymerase chain reaction; SAE=serious adverse event; SARS-CoV-2=severe acute respiratory syndrome coronavirus 2; TC=telephone call

a Visits will be performed in person unless the participant is otherwise directed to remain at home or be seen at a hospital.

b Unscheduled visits include visits for specific safety issues and/or evaluation of possible COVID-19. These visits may be performed in the clinic, by home visit, by hospital visit (if allowed by local policy), or via telemedicine/telephone visit. Required procedures at these visits include AE and concomitant medication collection. For participants evaluated for COVID-19, samples should be collected for SARS-CoV-2 testing. Should the visit occur in person, the visit should also include evaluation of vital signs, body temperature, pulse oximetry, and symptom-directed physical examination.

c Complete physical examination will be performed at the Final Visit (or ET); symptom-directed examination (if any symptoms) may be performed at other timepoints as indicated to assess changes from Screening.

d Blood pressure, heart rate, respiratory rate, and body temperature will be measured.

e Collection of blood samples is outlined in Section 4.5.5.3. The first 1,500 participants in Phase 3c (the Immunogenicity Noninferiority Subgroup, Phase 3c-1) will also have blood collected at Day 211 for assessment of antibody responses to the SARS-CoV-2 spike protein. These blood samples will be sent to the central laboratory. The last ~900 participants enrolled (Phase 3c-2) will have a phone visit on Day 211.

f Collection of nasal swab samples is outlined in Section 4.5.5.3. Use of provided nasal swab test kits (including 2 collection containers) to be sent to the central laboratory is strongly preferred. Approved alternate measures of virological confirmation of SARS-CoV-2 may be performed if use of provided kits is not feasible.

g Review of COVID-19 symptoms and risks will include a scripted interview of the participant for risks of recent exposure to COVID-19 and for symptoms of COVID-19 (Appendix 2). If symptoms or risk of COVID-19 is confirmed, testing for SARS-CoV-2 should be performed. If this is identified during the TC with the participant, an Unscheduled visit should be arranged.

h Review of AEs will include surveillance for unsolicited AEs, SAEs, MAAEs, and AEs leading to discontinuation/withdrawal; Solicited AE data will be gathered by Diary for Day 1 through Day 7 for all participants after each of the initial vaccination series only; Unsolicited AE data will be gathered by interview at clinic visits and telephone contacts through Screening and 28 days after each vaccination. SAEs, MAAEs, and AEs leading to discontinuation/withdrawal will be collected at all timepoints after signing of the informed consent.

# APPENDIX 2: CASE DEFINITIONS OF COVID-19

## Primary Case Definition (Based on FDA recommendation (DHHS 2020))

| Case Definition | Laboratory Finding* | Clinical Status | |
|---|---|---|---|
| | | **Symptoms** | **Other Clinical Parameters** |
| Uninfected | No positive SARS-CoV-2 test | None | None relevant |
| Asymptomatic SARS-CoV-2 Infection | Positive SARS-CoV-2 test | None | None relevant |
| Protocol-defined COVID-19 | Positive SARS-CoV-2 test | At least one of the following that is a NEW or WORSENING finding:<br>• Fever or chills<br>• Cough<br>• Shortness of breath or difficulty breathing<br>• Fatigue<br>• Muscle or body aches<br>• Headache<br>• New loss of taste or smell<br>• Sore throat<br>• Congestion or runny nose<br>• Nausea or vomiting<br>• Diarrhea | None relevant |
| Atypical COVID-19 | Positive SARS-CoV-2 test | Clinical findings suggestive of COVID-19 but not included in the row above | None relevant |
| Severe COVID-19 | Positive SARS-CoV-2 test | As above for protocol-defined COVID-19 | Any of the following:<br>• Clinical signs at rest indicative of severe systemic illness:<br>- Respiratory rate ≥30 per minute, |

| **Case Definition** | **Laboratory Finding*** | **Clinical Status** | |
|---|---|---|---|
| | | **Symptoms** | **Other Clinical Parameters** |
| | | | - Heart rate ≥125 per minute,<br>- $SpO_2$ ≤93% on room air at sea level or $PO_2/FiO_2$ <300 mm Hg<br>• Respiratory failure (defined as needing high flow oxygen, noninvasive ventilation, mechanical ventilation or ECMO)<br>• Evidence of shock:<br>- SBP <90 mm Hg, or<br>- DBP <60 mm Hg,<br>- or requiring vasopressors<br>• Significant acute renal, hepatic, or neurologic dysfunction<br>• Admission to an ICU<br>• Death |

Abbreviations: COVID-19=coronavirus disease 2019; DBP=diastolic blood pressure; ECMO=extracorporeal membrane oxygenation; $FiO_2$=fraction of inspired oxygen; ICU=intensive care unit; $pO_2$=partial pressure of oxygen; SARS-CoV-2=severe acute respiratory syndrome coronavirus 2; SBP=systolic blood pressure; $SpO_2$=oxygen saturation

**Alternate Case Definition for Grading of Severity of SARS-CoV-2 Infection/COVID-19 (Based on World Health Organization WHO 2020)**

| Case Definition | Lab Finding* | Clinical Status | Other Clinical Parameters | Score |
|---|---|---|---|---|
| Uninfected | No positive SARS-CoV-2 test | N/A | N/A | 0 |
| Mild Disease | Positive SARS-CoV-2 test | Ambulatory | Asymptomatic | 1 |
| | | | Symptomatic; independent | 2 |
| | | | Symptomatic; assistance needed | 3 |
| Moderate Disease | Positive SARS-CoV-2 test | Hospitalized | No oxygen therapy<br>NOTE: If hospitalized for isolation only, record status as for ambulatory patient | 4 |
| | | | Oxygen by mask or nasal prongs | 5 |
| Severe Disease | Positive SARS-CoV-2 test | Hospitalized | Oxygen by NIV or high flow | 6 |
| | | | Intubation and mechanical ventilation, $pO_2/FiO_2 \geq 150$ or $SpO_2/FiO_2 \geq 200$ | 7 |
| | | | Mechanical ventilation, $pO_2/FiO_2 < 150$ or $SpO_2/FiO_2 < 200$ or vasopressors | 8 |
| | | | Mechanical ventilation, $pO_2/FiO_2 < 150$ and vasopressors, dialysis or ECMO | 9 |
| Dead | Positive SARS-CoV-2 test | Dead | N/A | 10 |

Abbreviations: ECMO=extracorporeal membrane oxygenation; $FiO_2$=fraction of inspired oxygen; N/A=not applicable; NIV=noninvasive ventilation; $pO_2$=partial pressure of oxygen; SARS-CoV-2=severe acute respiratory syndrome coronavirus 2; $SpO_2$=oxygen saturation

***Laboratory Confirmation of SARS-CoV-2 Infection**

SARS-CoV-2 viral infection as measured by either reverse transcriptase-polymerase chain reaction (RT-PCR) test, other equivalent nucleic acid amplification–based test (ie, NAAT), or rapid antigen test. Results from the central laboratory (preferred) or from local laboratory will be used; however, if local tests are used, the lab test will be considered acceptable if it was obtained using

- A Food and Drug Administration (FDA)-cleared assay
- OR an assay performed in a laboratory that is currently Clinical Laboratory Improvement Amendment (CLIA-certified)
- OR an assay performed by a laboratory accredited according to the International Organization for Standardization (ISO) 15189 standard
- OR an assay accredited by a national or regional authority in the country concerned

# APPENDIX 3: CONTRACEPTIVE GUIDANCE

***Male Participant Reproductive Inclusion Criteria***

Male participants are eligible to participate if they agree to the following requirements during the intervention period and for at least 60 days after administration of study vaccine, which corresponds to the time needed to eliminate reproductive safety risk of the study intervention(s):

- Refrain from donating sperm.

PLUS, either:

- Be abstinent from heterosexual intercourse with a female of childbearing potential as their preferred and usual lifestyle (abstinent on a long-term and persistent basis) and agree to remain abstinent.

OR

- Must agree to use a male condom when engaging in any activity that allows for passage of ejaculate to another person.
- In addition to male condom use, a highly effective method of contraception may be considered in women of childbearing potential (WOCBP) partners of male participants (refer to the list of highly effective methods below).

***Female Participant Reproductive Inclusion Criteria***

A female participant is eligible to participate if she is not pregnant or breastfeeding, and at least 1 of the following conditions applies:

- Is not a WOCBP (see definitions below)

OR

- Is a WOCBP and using an acceptable contraceptive method as described below during the intervention period (for a minimum of 60 days after administration of study vaccine). The investigator should evaluate the effectiveness of the contraceptive method in relationship to the first dose of study intervention

The investigator is responsible for review of medical history, menstrual history, and recent sexual activity to decrease the risk for inclusion of a woman with an early undetected pregnancy.

***Woman of Childbearing Potential***

A woman is considered fertile following menarche and until becoming postmenopausal unless permanently sterile (see below).

If fertility is unclear (eg, amenorrhea in adolescents or athletes) and a menstrual cycle cannot be confirmed before the first dose of study intervention, additional evaluation should be considered, including follicle-stimulating hormone (FSH) test.

***Women in the following categories are not considered WOCBP***

- Premenopausal female with 1 of the following:

- Documented hysterectomy
- Documented bilateral salpingectomy
- Documented bilateral oophorectomy

For individuals with permanent infertility due to an alternate medical cause other than the above, (eg, Mullerian agenesis, androgen insensitivity), investigator discretion should be applied to determining study entry.

Note: Documentation for any of the above categories can come from the site personnel's review of the participant's medical records, medical examination, or medical history interview. The method of documentation should be recorded in the participant's medical record for the study.

- Postmenopausal defined as follows:
  - A postmenopausal state is defined as no menses for 12 months without an alternative medical cause.
  - A high FSH level in the postmenopausal range must be used to confirm a postmenopausal state in women under 60 years of age and not using hormonal contraception or hormone replacement therapy (HRT).
  - A female participant on HRT and whose menopausal status is in doubt will be required to use one of the nonestrogen hormonal highly effective contraception methods if they wish to continue their HRT during the study. Otherwise, they must discontinue HRT to allow confirmation of postmenopausal status before study enrollment.

***Contraception Methods Inclusion Criteria***

Contraceptive use by men or women should be consistent with local availability/regulations regarding the use of contraceptive methods for those participating in clinical trials.

Acceptable contraception methods include the following:

- Implantable progestogen-only hormone contraception associated with inhibition of ovulation
- Intrauterine device
- Intrauterine hormone-releasing system
- Bilateral tubal occlusion
- Vasectomized partner:
  - Vasectomized partner is a highly effective contraceptive method provided that the partner is the sole sexual partner of the woman of childbearing potential and the absence of sperm has been confirmed. If not, an additional highly effective method of contraception should be used. The spermatogenesis cycle is approximately 90 days.

- Combined (estrogen- and progestogen-containing) hormonal contraception associated with inhibition of ovulation:

  Oral
    - Intravaginal
    - Transdermal
    - Injectable
- Progestogen-only hormone contraception associated with inhibition of ovulation:
    - Oral
    - Injectable
- A combination of male condom with either cervical cap, diaphragm, or sponge with spermicide (double-barrier methods).
- Sexual abstinence:
    - Sexual abstinence is considered a highly effective method only if defined as refraining from heterosexual intercourse during the entire period of risk associated with the study intervention. The reliability of sexual abstinence needs to be evaluated in relation to the duration of the study and the preferred and usual lifestyle of the participant.

The following methods of birth control are <u>not regarded as highly reliable methods and are therefore discouraged as a single method</u> only for contraception:

- Progestogen-only oral hormonal contraception where inhibition of ovulation is not the primary mode of action
- Male or female condom with or without spermicide
- Cervical cap, diaphragm, or sponge with spermicide

# APPENDIX 4: TOXICITY GRADING SCALES

### Table 19 Toxicity Grading for Solicited Adverse Events

| **Solicited Local Adverse Events** | **Grade 0** | **Grade 1 (Mild)** | **Grade 2 (Moderate)** | **Grade 3 (Severe)** | **Grade 4 (Potentially Life Threatening)** |
|---|---|---|---|---|---|
| Injection Site Erythema * | <2.5 cm | 2.5-5 cm | 5.1-10 cm | >10 cm | Emergency Room (ER) visit, hospitalization or necrosis or exfoliative dermatitis |
| Injection Site Induration/Swelling ** | <2.5 cm | 2.5-5 cm | 5.1-10 cm | >10 cm | ER visit or hospitalization or necrosis |
| Injection Site Pain | None | Present but does not interfere with daily activity | Interferes with daily activity or leads to use of non-narcotic pain reliever for >24 hours | Prevents daily activity or leads to use of narcotic pain reliever | ER visit or hospitalization |
| Injection Site Tenderness | None | Mild discomfort to touch | Discomfort with movement | Significant discomfort at rest | ER visit or hospitalization |
| **Solicited Systemic Adverse Events** | | | | | |
| Fever (°C) ***<br>(°F) *** | <38.0<br><100.4 | 38.0-38.4<br>100.4-101.1 | 38.5-38.9<br>101.2-102.0 | 39.0-40<br>102.1-104 | >40<br>>104 |
| Nausea/vomiting | None | 1-2 episodes/24 hours | >2 episodes/24 hours without seeking medical attention | Requires outpatient intravenous hydration | ER visit or hospitalization for hypotensive shock |
| Diarrhea | <2 loose stools/24 hours | 2-3 loose stools/24 hours | 4-5 stools/24 hours | 6 or more loose or watery stools/24 hours or requires outpatient intravenous hydration | ER visit or hospitalization |

**Table 19 Toxicity Grading for Solicited Adverse Events**

| **Solicited Local Adverse Events** | **Grade 0** | **Grade 1 (Mild)** | **Grade 2 (Moderate)** | **Grade 3 (Severe)** | **Grade 4 (Potentially Life Threatening)** |
|---|---|---|---|---|---|
| Headache | None | Present but without interference with daily activity | Interferes with daily activity or leads to use of non-narcotic pain reliever for >24 hours | Prevents daily activity or leads to use of narcotic pain reliever | ER visit or hospitalization |
| Fatigue | None | Present but without interference with daily activity | Interferes with daily activity | Prevents daily activity | ER visit or hospitalization |
| Myalgia | None | Present but without interference with daily activity | Interferes with daily activity | Prevents daily activity | ER visit or hospitalization |
| Arthralgia, chills, and dizziness | None | Present but without interference with daily activity | Interferes with daily activity | Prevents daily activity | ER visit or hospitalization |

Abbreviation: ER=emergency room
* In addition to grading the measured local reaction at the greatest single diameter, the measurement should be recorded as a continuous variable.
** Induration/Swelling should be evaluated and graded using the functional scale as well as the actual measurement.
*** If temperature taken orally, no recent hot or cold beverages or smoking.
Adapted from Source: DHHS 2007

# APPENDIX 5: AT-RISK MEDICAL CONDITIONS

Ongoing surveillance of the spread and complications of severe acute respiratory syndrome coronavirus 2 infection has identified a set of conditions that are associated with increased risk of severe, life-threatening coronavirus disease 2019 (COVID-19).

COVID-19 has demonstrated both high attacks and elevated mortality rates in older individuals. As such, individuals older than 60 years are currently prioritized for immunization in several countries with COVID-19 vaccines available for distribution.

Each of the medical conditions described below has been named as probably or possibly associated with increased risk of severe complications of COVID-19 (CDC 2021).

For the purposes of determining which participants will be managed as "at risk," some relevant clinical definitions for each of these findings is provided. The information shared below should be evaluated against the participant's medical records for confirmation of risk.

- Cancer: Includes any individual with a history of malignancy EXCEPT
  - Malignancy with low potential risk for recurrence after curative treatment (eg, history of childhood leukemia) or metastasis in the opinion of the investigator
  - Adequately treated nonmelanoma skin cancer or lentigo maligna without evidence of disease
  - Adequately treated uterine cervical carcinoma in situ without evidence of disease
  - Localized prostate cancer
- Chronic kidney disease: Stage 3 (eg, estimated glomerular filtration rate $<60$ mL/min/1.73 $m^2$) or worse within the past year
- Chronic obstructive pulmonary disease such as chronic bronchitis or emphysema
- Cystic fibrosis
- Pulmonary fibrosis
- Down syndrome
- Cardiovascular conditions: Includes any participant with any of the following:
  - Hypertension that is regarded as uncontrolled for age according to the Eighth Joint National Committee guidelines (James 2014)
  - Congestive heart failure by New York Heart Association classification $\geq$2 (Dolgin 1994)
  - Recent (within 6 months prior to first study vaccination) exacerbation of coronary artery disease as manifested by cardiac intervention, addition of new cardiac medications for control of symptoms, or unstable angina
  - Cardiomyopathy
  - Pulmonary hypertension

- Obesity (body mass index of 30 $kg/m^2$ or higher measured at Screening)
- Sickle cell disease or other hemoglobinopathies such as thalassemia
- Current or former smoker defined as someone who has smoked more than 100 cigarettes in a lifetime (CDC 2017)
- Type 1 or type 2 diabetes mellitus
- Asthma (moderate-to-severe)
- Cerebrovascular disease (eg, stroke, transient ischemic attack, aneurysms, and vascular malformations)
- Dementia or Alzheimer’s
- Current or recent substances abuse disorder (alcohol, opioid, cocaine)
- Liver disease such as follows:
    - alcohol-related liver disease
    - nonalcoholic fatty liver disease
    - cirrhosis

# APPENDIX 6: PROTOCOL AMENDMENTS

## Protocol Amendment 1

The purpose of this amendment is to address the study design recommendations from the Vietnam Ministry of Health. The Phase 3a/b study was revised to a Phase 1/2/3 study design, with SRC review of safety data from one phase prior to enrollment of the next. In addition to the changes listed below, this amendment includes administrative changes for consistency of language and changes to the Synopsis for consistency with the changes to the main body outlined below, as well as correction of minor typographical and referencing errors.

**Protocol Amendment 1 (Version 2.0, 27 July 2021): Summary of Major Changes**

| Section Number | Description of Change | Brief Rationale |
|---|---|---|
| Section 1.1 | Study schematic updated to the current design | To align with the new study design and accurately reflect the Phases and timepoints |
| Section 3.1 and subsections | Objectives and Endpoints revised | To align with the new study design |
| Section 4.1 | Updated participant enrollment numbers | To align with the new study design |
| Section 4.4 | Revised text to reflect the new study design, including Phase 1, 2, and 3a/b, with safety reviews between phases. The immunogenicity subgroup is described as Phase 2 and 3a participants, and immunogenicity data at Day 57 will be pooled for the primary analysis. | To align with the new study design |
| Section 4.5.3.3 | Deleted reference to only collecting blood in Phase 3a | Blood being collected in all Phases of study |
| Section 4.5.3.5 | Corrected blood sample to nasal swab | Correction of an error in original protocol |
| Section 4.5.5.2 | Removed description of blood volumes to be collected. These will be described in the Lab Manual | Blood volumes will be described in the Lab Manual |
| Section 8 | Added language to describe how data integrity will be maintained | To align with the new study design |
| Section 8.1, | Added language to include the additional study phases | To align with the new study design |

## Protocol Amendment 1 (Version 2.0, 27 July 2021): Summary of Major Changes

| Section Number | Description of Change | Brief Rationale |
|---|---|---|
| Section 8.3.1 | Added language to include the additional study phases and the Immunogenicity Subgroup | To align with the new study design |
| Section 8.3.2 | Clarified where decision rules for sample size evaluation will be described | Clarification |
| Section 8.6.3 | Additional detail added concerning the analysis population | Consistency with stated objectives and endpoints |
| Section 8.9.1 | Added additional detail concerning time points for SRC review of safety data | To align with the new study design |
| Section 8.9.3 | Timing of constitution of DSMB updated | To align with the new study design |
| Section 14, Appendix 1 | Title updated to reflect that the Schedule of Assessments applies to all (Phases 1, 2 and 3) participants.<br><br>Footnote updated to clarify and emphasize the Immunogenicity Subgroup (Phases 2 and 3a) will have more frequent blood collection. | To align with the new study design<br><br>To align with the new study design |

## Protocol Amendment 2

The purpose of this amendment is to address the study design recommendations from the Vietnam Ethics Committee. The size of the Phase 1 part was increased and participants in Phase 1 have been incorporated into the Immunogenicity Subgroup. Assessment of safety laboratory testing had also been added for Phase 2 participants. In addition to the changes listed below, this amendment includes administrative changes for consistency of language and changes to the Synopsis for consistency with the changes to the main body outlined below, as well as correction of minor typographical and referencing errors.

**Protocol Amendment 2 (Version 3.0, 01 August 2021): Summary of Major Changes**

| Section Number | Description of Change | Brief Rationale |
|---|---|---|
| Title of Study | Reactogenicity removed from title | Request by Ethics Committee. |
| 3.1 | Updated description of Immunogenicity Subgroup and clarified the participants that each type of neutralizing antibody test will be performed in | To align with new study design. |
| 4.1 | Updated enrollment numbers for Phase 1 and Phase 2 | To align with new study design. |
| 4.4 | Revised text for Phase 1 to be 3:1 randomization, no stratification for Phase 1, and added Phase 1 to immunogenicity subgroup. Added that Phase 1 participants must be <60 years of age | To align with new study design. |
| 4.4 | Specified that tolerability criteria will be agreed with | Request by Ethics Committee. |
| 4.5.2 | Updated enrollment to be 3:1 (ARCT-154:placebo) for Phase 1. Clarified that no stratification will occur for Phase 1 participants | To align with new study design. |
| 4.5.3 | Updated number of visits for Phase 1 participants to have additional visits on Day 8 and 36. | To align with new study design. |
| 4.5.3.1 | Added nasal swab and blood sampling for screening and safety laboratories at screening for Phase 1. | To align with new study design. |
| 4.5.3.2 | For 3a and 3b, added collection of paper diaries, if applicable, at Day 29.<br><br>Added observation for at least 3 hours for Phase 1 initial series of vaccinations. | To correct an omission from previous version.<br><br>Longer observation in Phase 1 for the initial series of vaccine administrations. |
| 4.5.3.3 | Added Follow-up Visits on Day 8 and 36, including blood sampling for safety laboratories | To align with new study design. |

## Protocol Amendment 2 (Version 3.0, 01 August 2021): Summary of Major Changes

| Section Number | Description of Change | Brief Rationale |
|---|---|---|
| 4.5.3.4 | For Phase 3a and 3b, added collection of paper diaries, if applicable, at Day 57. | To correct an omission from previous version. |
| 4.5.5.2 | Added screening and safety laboratories at Screening for Phase 1.<br><br>Added safety laboratories at Day 8, 29, 36 and 57 for Phase 1 participants.<br><br>Added Phase 1 to Immunogenicity subgroup.<br><br>Added PRNT50 laboratory test at Day 29 and 57. | To align with new study design**.** |
| 5.1.2 | For Phase 1, added exclusions for clinically significant medical conditions and abnormal screening laboratory values.<br><br>Added exclusion of ≥ 60 years of age. | To align with new study design. |
| 5.1.3 | Clarified that all Phases except Phase 1 will be stratified for at-risk participants. | To align with new study design. |
| 5.2.1 | Revised language to clarify that participants must be rescreened if delay in vaccination is > 28 days after signing the informed consent. | Corrected language to align delay in study vaccination with Screening period. |
| 6.1 | Clarified that all Phases except Phase 1 will be stratified for at-risk participants. | To align with new study design. |
| 6.2 | New section describing contract organizations responsible for manufacturing ARCT-154. Following Section 6 subsections renumbered accordingly | Request by Ethics Committee. |
| 7.3 | Added safety blood sampling for Phase 1 to safety assessments. | To align with new study design. |
| 7.3.1.2 | Revised language from ongoing AE to ongoing disease/condition and clarified that conditions from signing informed consent and during screening period will be reported as medical history. | Clarified process for eliciting and documenting AEs. |
| 7.3.1.3.4 | Added new Subsection for laboratory test abnormalities | To align with new study design, which included adding safety laboratories for Phase 1 at Screening, Day 29 and Day 57. |
| 8.1 | Updated description of Immunogenicity Subgroup. Clarified in Table 9 that immunogenicity analysis target population | To align with new study design. |

## Protocol Amendment 2 (Version 3.0, 01 August 2021): Summary of Major Changes

| Section Number | Description of Change | Brief Rationale |
|---|---|---|
| | is only Phase 1, 2 and 3a participants and also clarified which neutralization tests will be performed as primary and secondary | |
| 8.3.1 | Amended the number of ARCT-154 participants in Phase 3a analysis of safety and clarified that the primary Nab test will be a pseudovirus assay | To align with new study design. |
| 8.4 | Clarified that Immunogenicity Analysis Set comprises participants in Phases 1, 2 and 3a | Clarification to align with new study design. |
| 8.6.2 | In Table 13 clarified which neutralization tests are primary and secondary | Clarification to align with new study design. |
| 8.6.3 | Clarified that Phase 3a immunogenicity analysis will be conducted in participants in Phases 1, 2 and 3a and that the primary Nab test will be a pseudovirus assay | Clarification to align with new study design. |
| 8.9.1 | Updated the enrollment number for Phase 1 to 100 participants. | To align with new study design. |
| Section 14, Appendix 1 | Added Phase 1 Schedule of Assessments Table, with additional visits on Days 8 and 36.<br><br>For Phase 2, 3a and 3b, added collection of paper diaries at Day 29 and 57.<br><br>Added Phase 1 to Immunogenicity Subgroup blood collection schedule. | To align with new study design<br><br>To correct omission from previous version.<br><br>To align with new study design. |

## Protocol Amendment 3

The primary purpose of this amendment is to address inconsistencies and errors in Protocol Version 3.0. In addition to the changes listed below, this amendment includes administrative changes for consistency of language and changes to the Synopsis for consistency with the changes to the main body outlined below, as well as correction of minor typographical and referencing errors.

**Protocol Amendment 3 (Version 4.0, 09 August 2021): Summary of Major Changes**

| Section Number | Description of Change | Brief Rationale |
|---|---|---|
| Title | Removed Phase 1/2/3 | Request from Ethics Committee |
| Section 2.6 | Revised to reflect ARCT-021 safety data as of Interim Analysis #2. | Provides a more accurate representation of available safety data. |
| Section 3.1.1 | Corrected the NAb assay from a pseudovirus assay to a surrogate virus neutralization test | Correct an error |
| Section 3.1.2 | Corrected the NAb assay from a pseudovirus assay to a surrogate virus neutralization test. Made PRNT50 a separate secondary endpoint | Correct errors |
| Section 4.1 | Clarified that the number of participants in Phase 1 will be 75 ARCT-154 and 25 placebo-treated. | Provides clarification. |
| Section 4.5.3 | Revised telephone calls to Day 216, 223, 230, 244, 251, 258, 265. | Corrects a miscalculation of study calls occurring at 7-day intervals. |
| Section 4.5.3.2 | Added language that the second study vaccination must be at least 28 days after the first vaccination, and the Switchover must be at least 180 days after the second vaccination of the initial series. | Provides clarification for the minimum interval between vaccinations. |
| Section 4.5.3.3 | Added language to clarify that visits on Day 8 and 36 must be at least 7 days after prior vaccination. | Ensures minimum time interval for safety evaluations. |
| Section 4.5.3.4 | Added clarification that Day 57 visit must occur at least 28 days after second vaccination. | Ensures minimum time interval for safety evaluations. |
| Section 4.5.3.5 | Added clarification that timing of the weekly phone calls is based on the prior study vaccination, and for Day 8 and 36, must be at least 7 days after vaccination. | Ensures minimum time interval for safety evaluations. |
| Section 4.5.3.7 | Added clarification that the Final Visit should be Day 394 for all participants.<br><br>Removed that Final Visit and ET Visit could occur by telemedicine. | Provides clarification for intent of protocol design.<br><br>Ensures that in person visit will occur to collect necessary safety assessments. |

## Protocol Amendment 3 (Version 4.0, 09 August 2021): Summary of Major Changes

| Section Number | Description of Change | Brief Rationale |
|---|---|---|
| Section 4.5.5.2 | Added for Day 29 that all participants will have nucleocapsid Ab testing performed. | Provides necessary lab testing for proper analysis of the primary endpoint, to identify participants who have had prior COVID-19. |
| Section 5.1.1 | Added link to Appendix 3 to Inclusion 4<br><br>Added to Inclusion 6 "in the opinion of the Investigator" | Provides a link to the contraceptive guidance.<br><br>Provides guidance to Investigators to use judgement in determining this. |
| Section 7.2 | Added laboratory requirements for immunogenicity laboratory tests | Ensures laboratory assessments are completed properly and ensures integrity of efficacy data. |
| Section 8.1.1 and 8.6.2 | Corrected the NAb assay from a pseudovirus assay to a surrogate virus neutralization test in Table 9 and Table 13 | Correct an error |
| Section 8.6.3.1 | Corrected the NAb assay from a pseudovirus assay to a surrogate virus neutralization test | Correct an error |
| Section 11.6 | Revised to "according to local reporting requirements." | Updated because IRB reporting requirements may not be annual. |
| Appendix 1 | Revised windows for study calls to +2 at Day 8, 15, 22, 43 and 50, and ±2 for all other study calls.<br><br>Revised windows for clinic visits to +2 days at Day 29, 36, and 57.<br><br>Revised footnote l to clarify Unscheduled visit must be arranged unless participant is clinically unstable.<br><br>Revised table name to 'Day 209 to End of Study Visit for All Participants'<br><br>Revised study calls to Day 216, 223, 230, 244, 251, 258 and 265. | Ensures a minimum safety follow-up of at least 7 days for key timepoints, while simplifying the study design.<br><br>Ensures a minimum safety follow-up for key timepoints while simplifying the study design.<br><br>Ensures appropriate data is collected by conducting Unscheduled Visits.<br><br>More accurately reflects table because not all participants will have Switchover vaccinations.<br><br>Corrects a miscalculation of study calls occurring at 7-day intervals. |
| Summary of Changes for Protocol Amendment 2 | Clarified that PRNT50 performed for Days 1, 29 and 57 by deleting erroneous reference to Days 209 and 394 | Correction of an error |

## Protocol Amendment 4

The primary purpose of this amendment is to change to allow Phase 3a to be initiated at the same time as Phase 2 and to change the randomization ratio for Phases 2 and 3a to 3:1 ARCT-154 to placebo. In addition to the changes listed below, this amendment includes administrative changes for consistency of language and changes to the Synopsis for consistency with the changes to the main body outlined below, as well as correction of minor typographical and referencing errors.

**Protocol Amendment 4 (Version 5.0, 24 August 2021): Summary of Major Changes**

| Section Number | Description of Change | Brief Rationale |
|---|---|---|
| Figure 1 | Study schematic updated | Updated to reflect the current study design in this amendment. |
| Section 3.1 and subsections and tables<br>Section 8.1.1<br>Section 8.2.1<br>Section 8.3.1<br>Section 8.6.1.1<br>Section 8.6.3 and subsections<br>Section 8.8 | Revised titles/text to reflect that objectives and endpoints relate to Phases 1, 2 and 3a | Provides a more accurate description. |
| Sections 4.4 and 8.9.1 | Revised text that Phases 2 and 3a will start simultaneously after review of safety data from Phase 1 through Day 36. | Due to the rapidly increasing rate of COVID cases in Vietnam, more rapid transition through the early phases is desirable in order to get to Phase 3 initiation as soon as possible. |
| Section 4.1 | Randomization revised to 3:1 for Phases 2 and 3a. | Updated to align with study plan. |
| Section 4.2 | The number of sites revised to approximately 20. | Updated to align with study plan. |
| Section 4.4 | Revised text that the SRC will review safety data for Phase 1 participants instead of referring to them as a sentinel cohort. | Language revised to be more accurate since all of Phase 1 participants will be enrolled and safety data reviewed by the SRC. |
| Section 4.4 | Revised the text such that Phases 2 and 3a start simultaneously after review of Phase 1 safety data through Day 56.<br><br>Updated text to be consistent with 3:1 randomization for Phases 2 and 3a. | Updated to align with study plan. |
| Section 4.5.2 | Updated text to be consistent with 3:1 randomization for Phases 2 and 3a. | Updated to align with study plan. |
| Section 4.5.3.1 | Updated text to reflect nasal swabs for SARS-CoV-2 RT-PCR will be performed in | Updated to align with study plan. Rapid results observed in Phase 1 |

## Protocol Amendment 4 (Version 5.0, 24 August 2021): Summary of Major Changes

| Section Number | Description of Change | Brief Rationale |
|---|---|---|
| | all participants at Screening, not only Phase 1 participants. | will permit testing of all participants in later Phases. |
| Section 4.5.3.2 | Revised to include Phase 2 participants who did not use eDiary to provide paper diaries at Day 29 Visit. | Correction to address omission. |
| Section 4.5.3.4 | Revised to include Phase 2 participants who did not use eDiary to provide paper diaries at Day 57 Visit. | Correction to address omission. |
| Section 5.1.2 | Revised exclusion criteria #3 to exclude participants with a positive nasal swab SARS-CoV-2 RT-PCR test at Screening. The exclusion will now apply to all participants, not only Phase 1 participants. | Due to the availability of rapid RT-PCR testing available to study sites, and the quick identification of these participants in Phase 1, rapid screening and exclusion of these participants has been expanded to Phase 2, 3a and 3b. |
| Section 8.3.1 | Updated sample size of Phase 1/2/3a ARCT-154-treated participants to 750 | Updated to align with study plan. |
| Section 8.9.3 | Revised to say that DSMB will be constituted prior to initiation of Phase 3b (previously Phase 3a and Phase 3b) | Initiation of Phase 3a now moved up to be in parallel with Phase 2 rather than in parallel with Phase 3b |
| Appendix 5 | Revised to create several sub-bullets.<br>Removed HIV. | Correction of formatting errors.<br>HIV is exclusionary, so no longer relevant as an At-Risk condition. |

## Protocol Amendment 5

The primary purpose of this amendment to add an additional Cohort (Phase 3c) in which participants are randomized to either ARCT-154 or the Astra Zeneca COVID-19 vaccine ChAdOx1 nCoV-19. This cohort will compare the two active vaccines for noninferiority based on immunogenicity endpoints. This amendment also reduces the period of observation prior to Switchover from 6 months to 2 months after the second dose of study vaccine (due to the aggressive government roll out of authorized vaccines it has become unethical to keep participants on placebo for a prolonged period) and moves the efficacy endpoints for Phase 3b from primary to exploratory (the shortened follow-up period prior to Switchover coupled with falling COVID-19 rates in Vietnam mean that it is unlikely that sufficient COVID-19 events will accrue for statistical analysis of efficacy). In addition to the changes listed below, reference to Cohort 3c has been added to multiple sections and this amendment includes administrative changes for consistency of language and changes to the Synopsis for consistency with the changes to the main body outlined below, as well as correction of minor typographical and referencing errors.

**Protocol Amendment 5 (Version 6.0, 28 October 2021): Summary of Major Changes**

| Section Number | Description of Change | Brief Rationale |
|---|---|---|
| Title pages | Protocol version and dates updated and sponsor name changed from Vinbiocare to Vinbiotech | Sponsor company changed its name |
| Figure 1 | Study schematic updated | Updated to reflect the current study design in this amendment. |
| Section 3 and subsections | Objectives and endpoints changed to reflect the new study design/objectives and Phase 3c objectives and endpoints added. A combined exploratory safety endpoint added that pools data from all phases of study | Updated to reflect study design changes. |
| Section 4.1 | Number of participants in Phase 3b amended and Phase 3c added | Updated to reflect the current study design in this amendment. |
| Section 4.4 | Changes made throughout this section to reflect new study design | Updated to reflect the current study design in this amendment. |
| Section 4.5.2 | Phase 3c added | Updated to reflect the current study design in this amendment |
| Section 4.5.3 | Visit schedule amended to move Switchover visits to Day 92 and Day 120. Telephone visits monthly after Switchover for Phase 1/2/3a/3b. Phase 3c schedule added | Updated to reflect the current study design in this amendment. |
| Section 4.5.3.2 | Amended Switchover visit days. Added clarifying text on training regarding AEs | Updated to reflect the current study design in this amendment |
| Section 4.5.3.4 | Added Day 43 visit for Cohort 3c | Updated to reflect the current study design in this amendment |

## Protocol Amendment 5 (Version 6.0, 28 October 2021): Summary of Major Changes

| Section Number | Description of Change | Brief Rationale |
|---|---|---|
| Section 4.5.3.5 | Clarified that Phase 3c paper diaries may be collected at Day 57 visit | Updated to reflect the current study design in this amendment |
| Section 4.5.3.6 | Added Day 211 visit for Cohort 3c | Updated to reflect the current study design in this amendment |
| Section 4.5.3.7 | Updated to reflect revised telephone call schedule | Updated to reflect the current study design in this amendment |
| Section 4.5.3.8 | Clarified the instructions for COVID-19 testing to include language from Protocol Clarification Memo #6 | Clarification of protocol procedures |
| Section 4.5.5.2 | Added clarification text for nasal swab sampling based on Protocol Clarification Memo #2<br>Added Phase 3c visits<br>Amended timing of Switchover Visits | Clarification of protocol procedures and updates to reflect the current study design in this amendment |
| Section 4.5.5.4 | Clarified the schedule of full physical exams for each cohort | Clarification of protocol procedures |
| Section 5.1.2 | Added an exclusion criterion specific to participants in Phase 3c | Updated to reflect the current study design in this amendment |
| Section 5.2.1 | Added instruction from Protocol Clarification Memo # 4for delay in second vaccination in the event of an intercurrent SARS-CoV-2 infection | Clarification of protocol procedures |
| Sections 6.2, 6.3 and 6.4, 6.4.3, 6.5 | Updated to include Astra Zeneca vaccine | Updated to reflect the current study design in this amendment |
| Section 6.5 | Added additional instruction for overdose | Clarification of protocol procedures |
| Section 7.2 | Clarified which assays used for antibody testing | Updated to reflect the current study design in this amendment |
| Section 8 and Subsections | Major revisions to endpoints and Estimand descriptions made to align with new study design | Updated to reflect the current study design in this amendment |

## Protocol Amendment 5

The primary purpose of this amendment to add an additional Cohort (Phase 3c) in which participants are randomized to either ARCT-154 or the Astra Zeneca COVID-19 vaccine ChAdOx1 nCoV-19. This cohort will compare the two active vaccines for noninferiority based on immunogenicity endpoints. This amendment also reduces the period of observation prior to Switchover from 6 months to 2 months after the second dose of study vaccine (due to the aggressive government rollout of authorized vaccines, it has become unethical to keep participants on placebo for a prolonged period) and moves the efficacy endpoints for Phase 3b from primary to exploratory (the shortened follow-up period prior to Switchover coupled with falling COVID-19 rates in Vietnam mean that it is unlikely that sufficient COVID-19 events will accrue for statistical analysis of efficacy). In addition to the changes listed below, reference to Cohort 3c has been added to multiple sections and this amendment includes administrative changes for consistency of language and changes to the Synopsis for consistency with the changes to the main body outlined below, as well as correction of minor typographical and referencing errors.

**Protocol Amendment 5 (Version 6.0, 28 October 2021): Summary of Major Changes**

| Section Number | Description of Change | Brief Rationale |
|---|---|---|
| Title pages | Protocol version and dates updated and sponsor name changed from Vinbiocare to Vinbiotech | Sponsor company changed its name |
| Figure 1 | Study schematic updated | Updated to reflect the current study design in this amendment. |
| Section 3 and subsections | Objectives and endpoints changed to reflect the new study design/objectives and Phase 3c objectives and endpoints added. A combined exploratory safety endpoint added that pools data from all phases of study | Updated to reflect study design changes. |
| Section 4.1 | Number of participants in Phase 3b amended and Phase 3c added | Updated to reflect the current study design in this amendment. |
| Section 4.4 | Changes made throughout this section to reflect new study design | Updated to reflect the current study design in this amendment. |
| Section 4.5.2 | Phase 3c added | Updated to reflect the current study design in this amendment |
| Section 4.5.3 | Visit schedule amended to move Switchover visits to Day 92 and Day 120. Telephone visits monthly after Switchover for Phase 1/2/3a/3b. Phase 3c schedule added | Updated to reflect the current study design in this amendment. |
| Section 4.5.3.2 | Amended Switchover visit days. Added clarifying text on training regarding AEs | Updated to reflect the current study design in this amendment |
| Section 4.5.3.4 | Added Day 43 visit for Cohort 3c | Updated to reflect the current study design in this amendment |

## Protocol Amendment 5 (Version 6.0, 28 October 2021): Summary of Major Changes

| Section Number | Description of Change | Brief Rationale |
|---|---|---|
| Section 4.5.3.5 | Clarified that Phase 3c paper diaries may be collected at Day 57 visit | Updated to reflect the current study design in this amendment |
| Section 4.5.3.6 | Added Day 211 visit for Cohort 3c | Updated to reflect the current study design in this amendment |
| Section 4.5.3.7 | Updated to reflect revised telephone call schedule | Updated to reflect the current study design in this amendment |
| Section 4.5.3.8 | Clarified the instructions for COVID-19 testing to include language from Protocol Clarification Memo #6 | Clarification of protocol procedures |
| Section 4.5.5.2 | Added clarification text for nasal swab sampling based on Protocol Clarification Memo #2<br>Added Phase 3c visits<br>Amended timing of Switchover Visits | Clarification of protocol procedures and updates to reflect the current study design in this amendment |
| Section 4.5.5.4 | Clarified the schedule of full physical exams for each cohort | Clarification of protocol procedures |
| Section 5.1.2 | Added an exclusion criterion specific to participants in Phase 3c | Updated to reflect the current study design in this amendment |
| Section 5.2.1 | Added instruction from Protocol Clarification Memo # 4for delay in second vaccination in the event of an intercurrent SARS-CoV-2 infection | Clarification of protocol procedures |
| Sections 6.2, 6.3 and 6.4, 6.4.3, 6.5 | Updated to include Astra Zeneca vaccine | Updated to reflect the current study design in this amendment |
| Section 6.5 | Added additional instruction for overdose | Clarification of protocol procedures |
| Section 7.2 | Clarified which assays used for antibody testing | Updated to reflect the current study design in this amendment |
| Section 8 and Subsections | Major revisions to endpoints and Estimand descriptions made to align with new study design | Updated to reflect the current study design in this amendment |

## Protocol Amendment 6

The primary purpose of this amendment to correct the size of Cohorts Phase 3b and Phase 3c. The sample size agreed with the Vietnam Ministry of Health for Phase 3c was 4,000, with 1,500 to be included for immunogenicity noninferiority endpoints, Protocol Version 6.0 incorrectly specified the size of this cohort as 1,500 participants. This error has been corrected by increasing the size of Phase 3c and reducing the size of Phase 3b accordingly. The other major change was returning the Phase 3b efficacy endpoint from Exploratory to Secondary, which was requested by the Ethics Committee (in Protocol Version 6.0 it had been moved from primary to exploratory). In addition to the changes listed below, the revised sample sizes for Phase 3b and Phase 3c are reflected in multiple sections and this amendment includes administrative changes for consistency of language and changes to the Synopsis for consistency with the changes to the main body outlined below, as well as correction of minor typographical and referencing errors.

**Protocol Amendment 6 (Version 7.0, 28 October 2021): Summary of Major Changes**

| Section Number | Description of Change | Brief Rationale |
|---|---|---|
| Title | Remove "Placebo" in title | The study control groups are no longer limited to placebo and include ChAdOx1 vaccine in Phase 3c. |
| Synopsis: Study Design and Protocol Section 4.4 | Clarification of primary purpose of each phase of study, addition of text plaining the study procedures overview. | To explain the rationale for each of the different phases of study |
| Synopsis: Study Objectives and Protocol Sections 3.1.3, 3.2.2, and 3.2.2 | Text added to indicate that the exploratory objective analyzing COVID-19 convalescent sera. The immunogenicity assessment(s) will be performed on the same assay(s) as that is/are used for assessing study vaccine immunogenicity. | To clarify specifications for the analysis of the COVID-19 convalescent sera. |
| | Text added to indicate that COVID-19 cases will be analyzed in Phase 1, 2, 3a participants as an exploratory objective | To clarify the handling of COVID-19 cases as a study objective in these phases of the study. |
| | An exploratory immunogenicity objective evaluating variants of concern was added | If assays are available to test immune responses against variants of concern, testing of vaccinated participants may be performed. |
| Synopsis: Statistical Considerations | Sample size calculations for participants in Phase 3c-1 and 3c-2 added | To elaborate on the study's power to evaluate safety data in Phase 3c. |
| Figure 1 | Figure updated to add asterisk | This change maps out the difference between Phase 3c-1 and Phase 3c-2 participants for immunogenicity assessments. |
| Section 2.8 | Addition of benefits and risks of ChAdOx1 vaccine added | This information is relevant to participants enrolling into Phase 3c, where the Astra Zeneca vaccine will be used as a comparator vaccine. |

## Protocol Amendment 6 (Version 7.0, 28 October 2021): Summary of Major Changes

| Section Number | Description of Change | Brief Rationale |
|---|---|---|
| Section 3.1.3 | Evaluation of sera for antibody responses to variants of concern added as an exploratory endpoint | This is required per sponsor conversations with Vietnam Ministry of Health |
| Section 3.2,2 | Vaccine efficacy moved from exploratory (Section 3.2) to a secondary endpoint | Request by Ethics Committee |
| Section 4.5.3 | Study visits numbers and types updated for each Phase | Improved protocol comprehension |
| Section 4.5.3.8 and Section 4.5.5.2 | Clarification of wording to allow for home testing for COVID-19 if clinically appropriate | Improved protocol compliance |
| Section 8.0 | Clarification text added | Clarification |
| Section 8.1 subsections | Clarification text added to tables | Clarification |
| Section 8.2.2 | Statistic hypothesis for vaccine efficacy added | Vaccine efficacy moved from exploratory to secondary endpoint |
| Section 8.3.3 | Text describing sensitivity of sample size for detection of adverse events in Phase 3c added | Safety is a key endpoint of Phase 3c |
| Section 8.6.3 | Formulae corrected and additional formula for GMFR added. Additional clarification added to text | Corrections and clarifications |
| Section 8.6.5 | Title corrected | Error correction |
| Section 8.6.6 | Section added to describe analysis of Phase 3b exploratory efficacy endpoints. Subsequent sections renumbered accordingly | Additional description of endpoints |
| Section 8.8 | Timing of interim analyses further clarified | Clarification |
| Section 11.4 | Text referring to the Code of Federal Regulations removed | These regulations cannot be applied outside of the US |

## Protocol Amendment 7

The primary purpose of this amendment is to add to the design that participants in Phase 2 and 3a who received ARCT-154 in the initial vaccination series will be further randomized to receive a 3rd vaccination of either ARCT-154 or placebo at Day 92 followed by placebo at Day 120 in order to compare immunogenicity after 3 injections of ARCT-154 with that after 2 injections. Administration of a 3rd dose of ARCT-154 to participants in Phase 2/3a allows the opportunity to evaluate the neutralizing antibody of activity of a 3rd (booster) dose of ARCT-154 against variants of concern, and in particular the newly identified omicron variant. Early data indicates that neutralizing antibody activity against omicron following two doses of other RNA vaccines may be substantially less than against other variant strains, but that activity following a third dose is considerably improved.

In addition, the principal analysis of Efficacy was changed to be performed as a secondary analysis in the pooled participant populations from Phase 1/2/3a/3b in order to increase statistical power of this analysis. Additionally, enrollment of Phase 3c was terminated at request of Vietnam Ministry of Health after enrollment of ~2,400 of the originally planned 4,000 since 4,000 participants are not needed for assessment of the principal immunogenicity endpoints. Therefore, this amendment updates the number of participants in Phase 3c and clarifies that Phase 3c immunogenicity assessments will be performed in 800 participants in subgroup Phase 3c-1. This amendment also includes administrative changes for consistency of language and changes to the Synopsis for consistency with the changes to the main body outlined below, as well as correction of minor typographical and referencing errors.

**Protocol Amendment 7 (Version 8.0, 14 December 2021): Summary of Major Changes**

| Section Number | Description of Change | Brief Rationale |
|---|---|---|
| Cover page | Name of sponsor company updated | Company name change |
| Section 1.1 | Study schematic (Figure 1) updated to reflect new study design | Updated to reflect the current study design in this amendment |
| Section 2.7 | Added rationale for the 3rd dose for cohorts 2/3a | Updated to reflect the current study design in this amendment |
| Section 3.0 and subsections | Updated objectives and endpoints to reflect the new study design<br>Wording of other objectives and endpoints amended for greater specificity/clarity | Updated to reflect the current study design in this amendment<br>Clarification changes |
| Section 3.3.2 | Deleted visit at Day 43. Made processing of samples at Day 394 optional for Phase 3c-1 secondary endpoint #6 | The principal noninferiority time point is Day 57 and inclusion of a visit at Day 43 as well adds unnecessary burden to participants. The Day 394 time point is not critical for the noninferiority comparison or characterization of long-term immunogenicity as the later time point is being evaluated in Phase 1/2/3a |

## Protocol Amendment 7 (Version 8.0, 14 December 2021): Summary of Major Changes

| Section Number | Description of Change | Brief Rationale |
|---|---|---|
| Section 3.4 | Section deleted | Redundant with the other changes made to the objectives and endpoints |
| Section 4.1 | Updated to reflect number of participants enrolled in Phase 3c at time of termination of enrollment. Added additional information on constitution of 3c-1 and 3c-2. Clarified that immunogenicity assessments will be performed on 800 participants in 3c-1. | Updated to reflect the current study design in this amendment<br>Clarification changes |
| Section 4.4 | Updated to include the revised study design elements and to include some additional clarifications. Clarified that immunogenicity assessments will be performed on 800 participants in 3c-1. | Updated to reflect the current study design in this amendment<br>Clarification changes |
| Section 4.5.2 | Updated to include the revised study design elements and to include some additional clarifications | Updated to reflect the current study design in this amendment<br>Clarification changes |
| Section 4.5.3 and subsections | Updated to include the revised study design elements and to include some additional clarifications | Updated to reflect the current study design in this amendment<br>Clarification changes |
| Section 4.5.3.2 | Reduced the period of post vaccine observation at Day 92 and Day 120 from 30 to 15 minutes to be consistent with observation period following other mRNA vaccines | Sufficient safety information has been accrued on study to reduce this observation period |
| Section 4.5.3.4 | Deleted this section as was for Day 43 visit which has been removed for cohort 3c-1 | |
| Section 4.5.5.1 and 4.5.5.2 | Updated to include the revised study design elements. Included some of the original Section 4.5.5.1 language under a new section 4.5.5.2 for clarity. Renumbering of subsequent sections | Updated to reflect the current study design in this amendment<br>Clarification changes |
| Section 4.5.5.3 | Deleted Day 43 visit for cohort 3c-1 | Updated to reflect the current study design in this amendment |
| Section 5.1.3 | Section header and section text amended for clarity | Clarification changes |
| Section 5.1.5 | Section header and section text amended to align with new study design elements | Updated to reflect the current study design in this amendment |
| Section 5.2.3 | Removed 'study vaccine not administered' as a screen fail criterion | Correction of error |
| Section 6.1 | Amended to reflect new study design elements | Updated to reflect the current study design in this amendment |
| Section 6.8.1 | Amended to reflect new study design elements | Updated to reflect the current study design in this amendment |

## Protocol Amendment 7 (Version 8.0, 14 December 2021): Summary of Major Changes

| Section Number | Description of Change | Brief Rationale |
|---|---|---|
| Section 7.2 | Additional details added on immunogenicity assays to be used in each stage of study | Additional detail for clarification |
| Section 7.2.1 | Amended level 3 heading of 7.2.1 to a level 2 heading of 7.3 and subsequent sections renumbered | Correction of error |
| Section 8.0 | Text revised to reflect changes in sequence of unblinding implemented with this amendment | Updated to reflect the current study design in this amendment |
| Section 8.1 and 8.4 | Section 8.4 moved up to become section 8.1. Provided more detailed description of the analysis sets | To place description of the analysis sets at the beginning of stats section for context, and to add further clarifications to this section |
| Section 8.2 | Amended to reflect new study design elements | Updated to reflect the current study design in this amendment |
| Section 8.2.1 | Added seropositivity neutralizing antibody (as assessed by sVNT for Phase 1/2/3a/3b and MNT for Phase 3c) to the criteria defining early infection<br>Additional intercurrent event (IcEv5) of 'Confounding on-study Medications' added<br>Text in tables amended for better clarity. Estimand handling corrected in Table 15 | Further clarification of IcEVs<br>Clarification changes and correction of errors |
| Section 8.3.2 | Removed the requirement for at least 50 events before statistical testing of vaccine efficacy. Moved vaccine efficacy in Phase 3b to be an exploratory endpoint. | Due to reduced opportunity to collect COVID events brought about by changes included in protocol version 6.0, the principal analysis of efficacy will now be performed in an analysis set that pools data from Phase 1,2,3a and 3b to maximize the number of events included in the analysis. |
| Section 8.3.3 | Corrected equation for the null hypothesis for the primary endpoint and more clearly specified the secondary endpoints to be tested in a hierarchical fashion. Changed the terminology used for the NIM from decimal (-0.1) to percentage (-10.0%) | Correction of error<br>Additional information |
| Section 8.3.4 | Section added to describe statistical hypothesis for the pooled analyses | Additional information added to reflect new study design |
| Section 8.4 and subsections | Additional text added for clarifications | Clarification |
| Section 8.4.4 | Section added to describe sample size considerations for the pooled safety analysis | Updated to include additional information relevant to study endpoints |

## Protocol Amendment 7 (Version 8.0, 14 December 2021): Summary of Major Changes

| Section Number | Description of Change | Brief Rationale |
|---|---|---|
| Section 8.5 | Amended Day 15 to Day 29 as no antibody testing being performed on Day 15<br>Clarification of language | Correction of errors and clarification changes |
| Section 8.6.1 and subsections | Additional clarifying text added | Align with revisions to the statistical analyses included in this amendment |
| Section 8.6.2 | Clarifying text added to Table 16 | Align with revisions to the statistical analyses included in this amendment |
| Section 8.6.3 and subsections | Clarifying text added. Text that was repetitive of other sections deleted and those sections cross-referenced instead to minimize chances of inconsistencies within the protocol<br>Error in formula for GMFR corrected<br>LOD corrected to LLOQ | Align with revisions to the statistical analyses included in this amendment<br>Correction of errors |
| Sections 8.6.4.1, 8.6.5 and 8.6.6 | Clarifying text added. Text that was repetitive of other sections deleted and those sections cross-referenced instead to minimize chances of inconsistencies within the protocol | Align with revisions to the statistical analyses included in this amendment |
| Section 8.6.7 | Section added to describe to pooled assessments | Align with revisions to the statistical analyses included in this amendment |
| Section 8.6.10 | Section header and section body text amended to align with revisions to statistical analysis included in this amendment | Align with revisions to the statistical analyses included in this amendment |
| Section 8.8 | Section header and section content amended, including addition of level 3 subheadings, to reflect both primary and interim analysis time points | Due to the complexities of analysis time points across all of the cohorts, having a section where all analysis time points are described aids interpretation of the study design |
| Appendix 1 | Schedule of assessments amended to reflect study design changes and to correct minor errors | Additional information added to reflect new study design<br>Correction of errors |

## Protocol Amendment 8

The primary purpose of this amendment is to declare the Phase 3b Efficacy and Safety analyses to be the overall primary endpoints for this study.

In the original version of the protocol (Version 1.0), vaccine efficacy in Phase 3b was the primary endpoint of the study. However, with protocol version 6.0, the period of placebo-controlled observation for collection of COVID events was shortened from 6 months to 2 months after last vaccination due to ethical concerns about maintaining participants on placebo for a prolonged period. This change coincided with a sharp reduction in the incidence of COVID in the Vietnamese population due to lockdown measures introduced by the Vietnamese government. Because of the combined impact of reduced placebo-controlled observation period together with reduced incidence of COVID in Vietnam, Sponsor had significant concerns about the ability to collect a sufficient number of COVID events to evaluate vaccine efficacy in a hypothesis testing fashion. Therefore, with protocol version 6, vaccine efficacy was demoted from being the primary endpoint. However, since that time, the incidence of COVID has increased substantially in Vietnam and at the time of writing of this amendment, there are over 400 PCR-confirmed COVID cases undergoing evaluation by the blinded event adjudication committee, which is sufficient to exclude 30% vaccine efficacy at lower bound of the 95% confidence interval if true vaccine efficacy is ≥50%. Sponsor therefore believes that it is appropriate to test vaccine efficacy as the primary endpoint.

This amendment also includes administrative changes for consistency of language and changes to the Synopsis for consistency with the changes to the main body outlined below, as well as correction of minor typographical and referencing errors.

**Protocol Amendment 8 (Version 9.0, 07 February 2022): Summary of Major Changes**

| Section Number | Description of Change | Brief Rationale |
|---|---|---|
| Title pages and headers | Updated version number and date | Align with new version number and date |
| Throughout the protocol | Amended ‘Switchover’ to ‘Switchover/Further Study Vaccine’ | This is a correction and aligns with the design changes introduced in protocol version 8.0 |
| Section 3 | Text added to clarify that the Phase 3b primary endpoints are the overall primary endpoints for the study, that Phase 1/2/3a and Phase 3c are substudies and that no adjustment of type 1 error is made as a consequence of endpoints evaluated in the Phase 1/2/3a or Phase 3c substudies. | Clarification of endpoints |
| Section 3.1 | Made it clear in the title that Phase 1/2/3a is a substudy | Clarification |
| Section 3.2 and subsections | The first Phase 3b secondary efficacy endpoint was moved to be the Phase 3b efficacy primary endpoint.<br>Phase 3b exploratory endpoints promoted to secondary endpoints as follows: | Phase 3b primary analyses are declared as the overall primary efficacy and safety analyses for the study, so original Phase 3b efficacy secondary efficacy endpoint |

## Protocol Amendment 8 (Version 9.0, 07 February 2022): Summary of Major Changes

| Section Number | Description of Change | Brief Rationale |
|---|---|---|
| | • 2nd and 3rd Phase 3b exploratory efficacy endpoints moved to be 1st and 3rd Phase 3b secondary endpoints<br>• 4th Phase 3b exploratory endpoint moved to be 4th Phase 3b secondary endpoint<br>The optional analyses of participants that have received any dose of study vaccine in the first vaccination series that were contained in the Phase 3b exploratory efficacy endpoints 1, 2, 3 and 4 remain as Phase 3b secondary endpoint 2, and Phase 3b exploratory endpoints 1, 2 and 3<br>Additional exploratory analyses 4 and 5 added to evaluate efficacy by specific strain, to explore the effect of emerging variant strains on efficacy. Original exploratory endpoint 6 deleted as now replaced by strain specific exploratory endpoints 4 and 5<br>Original exploratory endpoint 5 becomes exploratory endpoint 6 | promoted to a primary efficacy endpoint., The Phase 3b exploratory efficacy endpoints were also promoted to secondary to reflect the fact that the Phase3b mITT is now the principal cohort for efficacy analyses.<br>Explore the effect of emerging variant strains on efficacy |
| Section 3.4 and subsections | Clarified that Pooled analyses are conducted as sensitivity analyses.<br>The first pooled secondary efficacy endpoint was moved to be the Pooled efficacy primary endpoint.<br>Pooled exploratory endpoints promoted to secondary endpoints as follows:<br>• 2nd and 3rd Pooled exploratory efficacy endpoints moved to be 1st and 3rd Phase 3b secondary endpoints<br>• 1st Pooled exploratory endpoint moved to be 2nd pooled secondary endpoint<br>• 4th Pooled exploratory endpoint moved to be 4th Pooled secondary endpoint<br>The optional analyses of participants that have received any dose of study vaccine in the first vaccination series that were contained in the Pooled exploratory efficacy endpoints 2, 3 and 4 remain as Pooled exploratory endpoints 1, 2 and 3<br>Additional exploratory analyses 4 and 5 added to evaluate efficacy by specific strain, | Changes made to align with the Phase 3b efficacy endpoints as the pooled efficacy endpoints will be evaluated as sensitivity analyses to the Phase 3b efficacy analyses |

## Protocol Amendment 8 (Version 9.0, 07 February 2022): Summary of Major Changes

| Section Number | Description of Change | Brief Rationale |
|---|---|---|
| | to explore the effect of emerging variant strains on efficacy | |
| Section 5.1.5 | Amended title to 'Switchover/Further Study Vaccine Eligibility Criteria' | More correctly reflects what the section applies to |
| Section 8.1 | New section added to describe the endpoint testing strategy.<br>Subsequent sections renumbered accordingly | Clarification of endpoint testing strategy |
| Section 8.2 | Pooled SAS and RAS amended to only pool data from Phases 1, 2, 3a and 3b (ie, 3c removed) | This prevents unblinding of 3c participants prior to the final analysis of Phase 3c endpoints |
| Section 8.3 | Removed language relating to the risk of not having sufficient COVID events for analysis as at time of this amendment there are over 372 cases of symptomatic, PCR positive COVID in the EDC.<br>Additional clarifications relating to evaluations added<br>Pooled analyses amended to only pool data from Phases 1, 2, 3a and 3b (ie, 3c removed) | Correction<br>Align with vaccine efficacy being evaluated as primary endpoint<br>Prevents unblinding of 3c participants prior to the final analysis of Phase 3c endpoints |
| Section 8.3.1 | Correction of IcEv 3 in Table 14<br>Clarified in tables that Phase 1/2/3a and Phase 3c are substudies<br>Removed evaluation of vaccine efficacy based on confirmation antigen tests from Table 16 as these were removed from the endpoints with Protocol Amendment 7. | Correction/clarification |
| Section 8.4.1 | Made it clear in the title that Phase 1/2/3a is a substudy | Clarification |
| Section 8.4.2 | Added more detail to the Phase 3b statistical hypotheses as these are now primary endpoints | Align with changes in endpoint primacy introduced with this amendment |
| Section 8.4.3 | Made it clear in the title that Phase 3c is a substudy | Clarification and align with changes in endpoint primacy introduced with this amendment |
| Section 8.4.4 | Removed detailed description of statistical hypothesis for the pooled analyses as these are now sensitivity analyses. | Align with changes in endpoint primacy introduced with this amendment |
| Section 8.5 | Added a description of how overall sample size for the study was determined | Clarification |
| Section 8.5.1 | Made it clear in the title that Phase 1/2/3a is a substudy | Clarification |

## Protocol Amendment 8 (Version 9.0, 07 February 2022): Summary of Major Changes

| Section Number | Description of Change | Brief Rationale |
|---|---|---|
| 8.5.2 | Added more detailed description of sample size evaluation for Phase 3b | Align with changes in endpoint primacy introduced with this amendment |
| Section 8.5.3 | Made it clear in the title that Phase 3c is a substudy | Clarification and align with changes in endpoint primacy introduced with this amendment |
| Section 8.5.4 | This section on sample size considerations for the pooled analyses was deleted | Pooled analyses are no longer the overall primary endpoints for the study |
| Section 8.7.1.1 | Made it clear in the title that Phase 1/2/3a is a substudy | Clarification |
| Section 8.7.1.2 | Added more description to the Phase 3b statistical considerations | Clarification and align with changes in endpoint primacy introduced with this amendment |
| Section 8.7.1.3 | Made it clear in the title that Phase3c is a substudy | Clarification and align with changes in endpoint primacy introduced with this amendment |
| Section 8.7.2 | Added geometric mean antibody concentrations to the analysis method in Table 18.<br>Clarified that primary efficacy analysis will be conducted in Phase 3b mITT<br>Efficacy analyses based on antigen test removed from Table 18 as these no longer being performed as of Protocol Version 8.0 | Correction of erroneous omission<br>Correction of error<br>Align with changes in endpoint primacy introduced with this amendment |
| Section 8.7.3.1 | Made clear in title that Phase 1/2/3a is a substudy.<br>Correction of error in GMFR equation | Clarification<br>Correction of error |
| Section 8.7.3.4 | Added section on Phase 3b primary efficacy endpoint | Align with changes in endpoint primacy introduced with this amendment |
| Section 8.7.4.1 | Made it clear that Phase 1/2/3a and Phase 3c are substudies | Clarification |
| Section 8.7.4.2 | Section added | Clarification |
| Section 8.7.5 | Added additional details on pooled efficacy and safety analysis | Clarification |
| Section 8.7.6 | Deleted original Section 8.6.6 as redundant with the new section 8.7.4.2 | Deletion of duplication |
| Section 8.7.7 | Clarified that efficacy data after Day 92 will only be collected for Phase 3c participants | Clarification |

## Protocol Amendment 8 (Version 9.0, 07 February 2022): Summary of Major Changes

| Section Number | Description of Change | Brief Rationale |
|---|---|---|
| Section 8.7.8 | Clarified that efficacy data after Day 92 will only be collected for Phase 3c participants and will be presented up to Day 394. | Clarification |
| Section 8.9.1 | Made it clear in the title that Phase 1/2/3a is a substudy. Added further clarification concerning timing of the safety analysis | Clarification |
| Section 8.9.2 | Clarified timing of evaluation of Phase 3b endpoints | This change aligns description with what is actually happening |
| Section 8.9.3 | Made it clear in the title that Phase 3c is a substudy | Clarification |
| Appendix 1, Table 'DAY 92 TO END OF STUDY VISIT FOR PHASE 1, PHASE 2, PHASE 3A AND PHASE 3B PARTICIPANTS' | Corrected footnote 'j' to clarify the different post-dose observation periods for the 3rd dose for Phase 1/3b and Phase 2/3a participants | Correction of error |

NCT #: NCT05012943

**Statistical Analysis Plan**

**Final Analysis**

**for**

**Protocol ARCT-154-01**

**A Randomized, Observer-Blind, Controlled Study to Assess the Safety, Immunogenicity and Efficacy of the SARS-CoV-2 Self-Amplifying RNA Vaccine ARCT-154 in Adults**

| | |
|---|---|
| **Protocol Number: (Version Date)** | Version 9.0<br>07 Feb 2022 |
| **Name of Test Drug:** | ARCT-154 |
| **Phase:** | 1/2/3 |
| **Methodology:** | Randomized, Observer-Blind, Controlled Study |
| **Sponsor:** | Vinbiocare Biotechnology Joint Stock Company<br>Techno Park building, Vinhomes Ocean Park Residential Area, Da Ton ward, Gia Lam District,<br>Hanoi, Vietnam |
| **Sponsor Representative for ex-Vietnam analyses and filings:** | [redacted]<br>Arcturus |
| **Sponsor Representative for ex-Vietnam analyses and filings:** | [redacted]<br>Arcturus |
| **Document Date:** | 20July2023 |
| **Document Version:** | Final Analysis SAP Version 1.0 |



Arcturus / Cytel Confidential: This is a controlled document and shall not be changed, saved, copied or reproduced by any unauthorized person.

SIGNATURE PAGE

| | |
|---|---|
| **Protocol Title:** | A Randomized, Observer-Blind, Controlled Study to Assess the Safety, Immunogenicity and Efficacy of the SARS-CoV-2 Self-Amplifying RNA Vaccine ARCT-154 in Adults |
| **Sponsor:** | Vinbiocare Biotechnology Joint Stock Company<br>Techno Park building, Vinhomes Ocean Park Residential Area, Da Ton ward, Gia Lam District,<br>Hanoi, Vietnam |
| **Protocol Number:** | Protocol ARCT-154-01 |
| **Document Date/Version:** | Final Analysis SAP Version 1.0 |

**Cytel, Inc. Author:**
[redacted]
Cytel, Inc.
675 Massachusetts Avenue
Cambridge, MA 02139

**Signature:** [redacted]
**Email:** [redacted]
Date: 21-Jul-2023

**Sponsor Approval**

By signing this document, I acknowledge that I have read the document and approve of the planned statistical analyses described herein. I agree that the planned statistical analyses are appropriate for this study, are in accordance with the study objectives, and are consistent with the statistical methodology described in the protocol, clinical development plan, and all applicable regulatory guidance's and guidelines.

I have discussed any questions I have regarding the contents of this document with the biostatistical author.

I also understand that any subsequent changes to the planned statistical analyses, as described herein, may have a regulatory impact and/or result in timeline adjustments. All changes to the planned analyses will be described in the clinical study report (CSR).

**Sponsor Representative for ex-Vietnam analyses and filings:**
[redacted]
Arcturus Therapeutics, Inc.
10628 Science Center Dr #250

San Diego, CA 92121

**Signature:** [redacted]
**Email:** [redacted]
Date: 24-Jul-2023



Arcturus / Cytel Confidential: This is a controlled document and shall not be changed, saved, copied or reproduced by any unauthorized person.

**Sponsor Representative for ex-Vietnam analyses and filings:**
[redacted]
Arcturus Therapeutics, Inc.
10628 Science Center Dr #250

San Diego, CA 92121

**Signature:** [redacted]

**Email:** [redacted]

Date: 23-Jul-2023



Arcturus / Cytel Confidential: This is a controlled document and shall not be changed, saved, copied or reproduced by any unauthorized person.

**MODIFICATION HISTORY**

| Current Version | Date | Amended by | Summary of Changes from previous version | Reason |
|---|---|---|---|---|
| 1.0 | 20July2023 | | Original document | |
| | | | | |



Arcturus / Cytel Confidential: This is a controlled document and shall not be changed, saved, copied or reproduced by any unauthorized person.

## Table of Contents


1 INTRODUCTION AND OBJECTIVES OF ANALYSIS .......... 10
1.1 INTRODUCTION .......... 10
1.2 OBJECTIVES .......... 10
2 STUDY DESIGN .......... 21
2.1 INTRODUCTION TO STUDY DESIGN .......... 21
2.2 RANDOMIZATION METHODOLOGY .......... 22
2.3 STOPPING RULES .......... 22
2.4 BLINDING .......... 23
2.5 INTERIM ANALYSES .......... 23
3 STUDY ENDPOINTS .......... 26
4 ANALYSIS SETS .......... 27
4.1 ANALYSIS SET DEFINITIONS .......... 27
4.2 PROTOCOL DEVIATIONS .......... 31
5 DATA HANDLING .......... 32
5.1 COMPUTING ENVIRONMENT .......... 32
5.2 DATA CONVENTIONS .......... 32
5.3 REPORTING OF DATA ACROSS STUDIES AND OUTPUT DISPLAYS .......... 32
5.3.1 Output Displays .......... 33
5.4 WITHDRAWALS, DROPOUTS, LOSS TO FOLLOW-UP .......... 34
5.5 VISIT WINDOWS .......... 34
6 STATISTICAL METHODS .......... 35
6.1 SAMPLE SIZE JUSTIFICATION .......... 35
6.2 GENERAL STATISTICAL METHODS .......... 36
6.2.1 General Methods .......... 36
6.2.2 Definition of Baseline .......... 37
6.2.3 Statistical Hypothesis .......... 37
6.2.4 Multiple Comparisons/Multiplicity .......... 38
6.2.5 Subgroups .......... 39
6.2.6 Missing, Unused, and Spurious Data .......... 40
6.2.7 Intercurrent Events .......... 40
6.3 STUDY POPULATION .......... 41
6.3.1 Participant Disposition, Analysis Sets .......... 41
6.3.2 Demographic and Baseline Characteristics .......... 42
6.3.3 Prior and Concomitant Medication .......... 42
6.3.4 Exposure and Compliance .......... 43
6.3.5 Serostatus by SARS-CoV-2 nucleocapsid antibody test .......... 43




*Arcturus / Cytel Confidential: This is a controlled document and shall not be changed, saved, copied or reproduced by any unauthorized person.*

6.4 EFFICACY EVALUATION ........ 43
6.4.1 Primary Efficacy Analysis ........ 46
6.4.2 Secondary Analysis ........ 47
6.4.3 Exploratory Analysis ........ 48
6.5 IMMUNOGENICITY EVALUATIONS ........ 50
6.5.1 Overview of Immunogenicity Analyses ........ 53
6.5.2 Neutralizing antibody (NAb) responses to ARCT-154 by surrogate virus neutralization test (sVNT) ........ 57
6.5.3 Early neutralizing antibody responses using a live virus assay: NAb responses by plaque reduction neutralization test at 50% reduction (PRNT50) ........ 58
6.5.4 IgG antibody binding the SARS-CoV-2 spike protein (binding antibody [BAb]) ........ 58
6.5.5 Neutralizing antibody (NAb) responses to ARCT-154 by pseudovirus microneutralization test (MNT) ........ 58
6.5.6 Serum IgG antibodies binding the SARS-CoV-2 spike by Mesoscale Discovery (MSD) multiplex assay ........ 59
6.5.7 Immunogenicity Analysis by subgroups ........ 59
6.6 SAFETY EVALUATIONS ........ 60
6.6.1 Adverse Events ........ 60
6.6.2 Laboratory Data ........ 67
6.6.3 Vital Signs and Physical Examinations ........ 68
7 CHANGES TO PLANNED ANALYSES ........ 69
7.1 SUMMARY OF CHANGES FROM PROTOCOL IN THIS SAP (CHANGES NOT INTRODUCED SAP PART 1 OR SAP PART 2) ........ 69
7.2 SUMMARY OF CHANGES FROM PROTOCOL DOCUMENTED IN SAP PART 1 AND SAP PART 2 APPLICABLE TO THIS SAP ........ 76
7.3 SUMMARY OF CHANGES FROM PROTOCOL DOCUMENTED IN SAP PART 1 AND SAP PART 2 NOT IMPLEMENTED IN THIS SAP ........ 83
8 REFERENCES ........ 87
9 APPENDICES ........ 88
9.1 SCHEDULE OF ASSESSMENTS ........ 88
9.2 ESTIMANDS ........ 89
9.2.1 Estimands for Phase 1/2/3a Substudy and Phase 3c Substudy Primary, Secondary and Exploratory Immunogenicity Endpoints ........ 89
9.2.2 Estimands for Phase 1/2/3a, Phase 3b, and Pooled Primary, Secondary and Exploratory Efficacy Analyses ........ 90
9.2.3 Estimands for Primary Safety Analyses for Phase 1/2/3a, Phase 3b, Phase 3c and Pooled Cohorts ........ 91



Arcturus / Cytel Confidential: This is a controlled document and shall not be changed, saved, copied or reproduced by any unauthorized person.

9.3 OVERVIEW OF ASSAY AND AVAILABLE ANALYZED SAMPLES BY PHASE ....... 95
9.4 LABORATORY CONVERSION FACTORS ....... 97
9.5 EXAMPLE SAS CODE ....... 98

## List on in-text Tables

**Page**

TABLE 1 PHASE 1/2/3A SUBSTUDY OBJECTIVES AND ENDPOINTS ....... 11
TABLE 2 PHASE 3B OBJECTIVES AND ENDPOINTS ....... 13
TABLE 3 PHASE 3C OBJECTIVES AND ENDPOINTS ....... 16
TABLE 4 POOLED OBJECTIVES AND ENDPOINTS ....... 18
TABLE 5 INTERCURRENT EVENTS ....... 40
TABLE 6 OVERVIEW OF EFFICACY ANALYSIS ....... 43
TABLE 7 OVERVIEW OF PRIMARY, SECONDARY AND EXPLORATORY IMMUNOGENICITY ANALYSES ....... 54
TABLE 8 OVERVIEW OF SAFETY ENDPOINTS ....... 60
TABLE 9 VITAL SIGNS TOXICITY GRADING SCALES ....... 68



Arcturus / Cytel Confidential: This is a controlled document and shall not be changed, saved, copied or reproduced by any unauthorized person.

## ABBREVIATIONS

| **Abbreviation** | **Definition** |
|---|---|
| ACE2 | Angiotensin-converting Enzyme 2 |
| AE | Adverse Events |
| ATC | Anatomic Therapeutic Class |
| BAb | Binding Antibody |
| BMI | Body Mass Index |
| CI | Confidence Interval |
| COVID-19 | Coronavirus Disease 2019 |
| CRF | Case Report Form |
| CRO | Clinical Research Organization |
| DSMB | Data and Safety Monitoring Board |
| ET | Early Termination |
| GMC | Geometric Mean Concentration |
| GMT | Geometric Mean Titer |
| GMFR | Geometric Mean Fold Rise |
| HR | Hazard Ratio |
| IAS | Immunogenicity Analysis Set |
| IcEv | Intercurrent Event |
| ICH | International Council on Harmonization |
| IgG | Immunoglobulin G |
| ITT | Intent-to-treat |
| LLOQ | Lower Limit of Quantification |
| MAAE | Medically Attended Adverse Event |
| MedDRA | Medical Dictionary for Regulatory Activities |
| mITT | Modified Intent-to-treat |
| MNT | Microneutralization Test |
| MSD | Mesoscale Discovery |
| Nab | Neutralizing Antibody |
| NIHE | National Institute of Hygiene and Epidemiology (Vietnam) |
| PP | Per-Protocol |
| PRNT50 | Plaque Reduction Neutralization Test at 50% Reduction |
| PT | Preferred Term |
| RAS | Reactogenicity Analysis Set |
| RS | Randomized Set |
| RT-PCR | Reverse Transcriptase-polymerase Chain Reaction |
| SAE | Serious Adverse Event |
| SAP | Statistical Analysis Plan |



*Arcturus / Cytel Confidential: This is a controlled document and shall not be changed, saved, copied or reproduced by any unauthorized person.*

| Abbreviation | Definition |
|---|---|
| SARS-CoV-2 | Severe Acute Respiratory Syndrome Coronavirus 2 |
| SAS | Safety Analysis Set |
| SOC | System/Organ/Class |
| sVNT | Surrogate Virus Neutralization Test |
| ULOQ | Upper Limit of Quantification |
| VAERD | Vaccine-associated Enhanced Respiratory Disease |
| VOC | Variant of Concern |
| VOI | Variant of Interest |
| WHO | World Health Organization |



Arcturus / Cytel Confidential: This is a controlled document and shall not be changed, saved, copied or reproduced by any unauthorized person.

# 1 INTRODUCTION AND OBJECTIVES OF ANALYSIS

## 1.1 Introduction

This statistical analysis plan (SAP) is designed to outline the methods to be used in the final analysis of study ARCT-154-01. The analysis is intended to provide results for the study objectives for Phase 1/2/3a, Phase 3b, and Phase 3c at the end of study that are relevant for the clinical study report (CSR).

This SAP is based on the ARCT-154-01 Protocol Version 9, dated 7 February 2022. There are 3 previous SAPs for this study, all were based on the Protocol Version 9.

1. SAP Part 1 focusing on Phase 1/2/3a data, dated 31 March 2022
2. SAP Part 2 focusing on Phase 3b data, dated 6 April 2022
3. The 6-months analysis SAP, Version 2.0 dated 10 March 2023

SAP Part 1 and Part 2 were finalized prior to the second of two interim analyses performed by Vinbiocare in April 2022 (full details on authoring of these SAPs and interim analyses timings can be found in Section 2.5). The 6 months analysis SAP documented the re-analysis using CDISC-compliant approach that replicated the analyses previously conducted by Vinbiocare, including additional safety and immunogenicity data, for use in ex-Vietnam regulatory submissions.

This final analysis SAP will outline any differences between the planned study objectives in the Protocol Version 9 and planned implementation of these objectives in this SAP. Relevant changes introduced for these objectives within SAP Part 1, SAP Part 2, or 6-month analysis SAP are also documented in this final analysis SAP and described in Section 7, changes to planned analyses. The final analysis will use a CDISC-compliant approach.

## 1.2 Objectives

The objectives and endpoints being assessed as part of the final analysis are described below in Table 1 for Phase 1/2/3a, Table 2 for Phase 3b, Table 3 for Phase 3c, and Table 4 for Pooled Analysis across all Phases. Protocol specified study objectives are available in Section 3 of the protocol; clarifications/revisions/corrections to the Phase 1/2/3a/3b protocol objectives/endpoints made within SAP Part 1 Section 4, SAP Part 2 Section 3, and 6-month analysis SAP Section 1.2 are also reflected in Table 1 and Table 2 below where applicable. Other protocol objectives are not assessed in the final analysis because the data are not available. In interpreting the objectives and endpoints for this study, the following consideration will apply:

- The overall primary efficacy objective of the study is the primary objective in the Phase 3b part of the study.



Arcturus / Cytel Confidential: This is a controlled document and shall not be changed, saved, copied or reproduced by any unauthorized person.

## Table 1 Phase 1/2/3a Substudy Objectives and Endpoints

| **Phase 1/2/3a Primary Objectives** | **Endpoint Description** |
|---|---|
| 1. To assess the safety and reactogenicity of ARCT154 compared to placebo | Safety:<br>• Any unsolicited adverse events (AE) starting within 28 days after each study vaccine administration, summarized by severity and relationship to study vaccine.<br>• Any medically attended adverse event (MAAE), serious adverse event (SAE), or AE leading to discontinuation/withdrawal through Final Visit/Early Termination (ET) summarized by relationship to study vaccine.<br>Reactogenicity following each vaccination:<br>• Any solicited local or systemic AE starting within 7 days following each study vaccine administration (collected on Day 1 and Day 29 for Phase 1 and on Day 1, Day 29 and Day 92 for Phase 2 and Phase 3a) by toxicity grade. |
| 2. To assess the neutralizing antibody (NAb) responses to ARCT-154 by surrogate virus neutralization test (sVNT) at Day 57 | NAb responses by sVNT evaluated at Day 1 (baseline) and Day 57 for assessment of seroconversion.<br>The endpoint is defined as the proportion of participants in each study vaccine group that demonstrate seroconversion (defined as: 4-fold increase in antibody concentration from baseline). |
| **Phase 1/2/3a Secondary Objectives** | **Endpoint Description** |
| 1. To assess the neutralizing and Immunoglobulin G (IgG) binding antibody responses to ARCT-154 over time | NAb by sVNT and IgG antibody binding the full-length SARS-CoV-2 spike protein (binding antibody [BAb]) responses; assessments for both BAb and NAb will include:<br>• Antibody level: Geometric mean concentration (GMC) (BAb and NAb)<br>• Increase in antibody levels from baseline (Geometric mean fold rise [GMFR])<br>• Proportion of participants in each study vaccine group with seroconversion at each timepoint. |
| 2. To assess early neutralizing antibody responses using a live virus assay | NAb responses by plaque reduction neutralization test at 50% reduction (PRNT50) will be evaluated in the subset of the phase 1/2 IAS evaluated at Day 1 (baseline), Day 29 and Day 57.<br>The following endpoints will be assessed:<br>• GMC<br>• Increase in antibody levels from baseline (GMFR)<br>• Proportion of participants in each study vaccine group with seroconversion. |



*Arcturus / Cytel Confidential: This is a controlled document and shall not be changed, saved, copied or reproduced by any unauthorized person.*

| Phase 1/2/3a Exploratory Objectives | Endpoint Description |
|---|---|
| *1. To compare the humoral immune responses to ARCT-154 with those following COVID-19* | *Day 57 GMT/GMC results following vaccination with ARCT-154 may be compared with the comparable test results for convalescent sera from COVID-19 patients measured on the same assay for the subset of the Phase 1/2/3a IAS comprising all participants enrolled in Phase 1 and the first 50 evaluable participants in Phase 2. The following assays will be used:*<br>• *PRNT50*<br>• *MNT* |
| 2. To evaluate the efficacy of ARCT154 compared to placebo for the prevention of virologically confirmed COVID-19 | Efficacy evaluated in participants in the Phase 1/2/3a mITT. The endpoint is as follows:<br>• The first occurrence of confirmed, protocol-defined COVID-19 with onset between Day 36 and Day 92 inclusive.<br>Efficacy evaluated in ITT participants that have received any dose of study vaccine in the first vaccination series, with no evidence of infection prior to vaccination (defined as no positive RT-PCR or other COVID-19 test or seropositivity for anti-nucleocapsid antibody prior to Day 1). The endpoint is as follows:<br>• The first occurrence of confirmed, protocol-defined COVID19 with onset at any time after the first study vaccination and up to Day 92. |
| 3. To assess the neutralizing antibody immune responses to ARCT-154 to SARS-CoV-2 variants of concern (VOC)/variants of interest (VOI) following 2 and 3 vaccinations with ARCT-154 | Blood samples from post-vaccination timepoints evaluated for immune responses to SARS-CoV-2 VOC/VOI.<br><br>The following assays will be used:<br>• ACE2/sVNT (Phase 1/2 IAS at Day 1 and Day 57)<br>• MNT (Phase 1/2/3a IAS at Day 1, Day 57 and Day 92; Phase 2/3a at Day 1, Day 57, Day 92 and Day 120) |
| 4. To assess neutralizing antibody responses using a pseudovirus microneutralization test (MNT) following 2 (Day 57) and 3 (Day 120) vaccinations with ARCT-154 | NAb responses by MNT evaluated for the following groups/timepoints:<br>• Phase 1/2/3a IAS at Day 1, Day 57, Day 92<br>• Phase 2/3a IAS at Day 1, Day 57, Day 92 and Day 120<br>The following endpoints will be assessed:<br>• GMC<br>• Increase in antibody levels from baseline (GMFR)<br>• Proportion of participants in each study vaccine group with seroconversion |
| 5. To assess neutralizing antibody responses by surrogate virus neutralization test to ARCT-154 following 2 | NAb responses by sVNT evaluated at Day 1 (baseline), Day 57, and Day 120 for assessment of seroconversion in Phase 2/3a IAS that received ARCT-154 in the initial vaccination series |



Arcturus / Cytel Confidential: This is a controlled document and shall not be changed, saved, copied or reproduced by any unauthorized person.

| | |
|---|---|
| (Day 57) and 3 (Day 120) vaccinations with ARCT-154 in Phase 2/3a participants | • The endpoint is defined as the proportion of participants that demonstrate seroconversion (defined as: 4-fold increase in antibody concentration from baseline).<br><br>Data will be summarized according to use of international reference standards, if available. |
| 6. To assess the IgG binding antibody responses to ARCT-154 following 2 (Day 57) and 3 (Day 120) vaccinations with ARCT-154 in Phase 2/3a participants | IgG antibody binding the full-length SARS-CoV-2 spike protein (binding antibody [BAb]) responses evaluated at Day 1 (baseline), Day 57 and Day 120 in all participants in the Phase 2/3a IAS that received three doses of study vaccine. The following endpoints will be assessed:<br>• GMC<br>• Increase in antibody levels from baseline (GMFR)<br>• Proportion of participants in each study vaccine group with seroconversion |

Objectives/Endpoints in italicized are not analyzed and more details are provided in Section 7.

## Table 2 Phase 3b Objectives and Endpoints

| **Phase 3b Primary Objectives** | **Endpoint Description** |
|---|---|
| 1. To assess the safety and reactogenicity of ARCT-154 compared to placebo | Safety evaluated as:<br>• Any unsolicited AE starting within 28 days after each study vaccine administration, summarized by severity and relationship to study vaccine.<br>• Any MAAE, SAE, or AE leading to discontinuation/withdrawal through Final Visit/Early Termination (ET)summarized by relationship to study vaccine.<br>Reactogenicity will be summarized following vaccinations on Day 1 and Day 29:<br>• Any solicited local or systemic AE starting within 7 days after each study vaccine administration by toxicity grade. |
| 2. To evaluate the efficacy of ARCT-154 compared to placebo for the prevention of virologically confirmed COVID-19 | Efficacy evaluated in all participants in the Phase 3b mITT. The endpoint to be evaluated is as follows:<br>• The first occurrence of confirmed, protocol-defined COVID19 with onset between Day 36 and Day 92 inclusive. |
| **Phase 3b Secondary Objectives** | **Endpoint Description** |
| 1. To evaluate the efficacy of ARCT-154 compared to placebo for the prevention of | Efficacy evaluated in all participants in the Phase 3b mITT. The endpoint to be evaluated is as follows: |



*Arcturus / Cytel Confidential: This is a controlled document and shall not be changed, saved, copied or reproduced by any unauthorized person.*

| | |
|---|---|
| virologically confirmed severe COVID-19 | • The first occurrence of confirmed, protocol-defined severe COVID-19 with onset between Day 36 and Day 92 inclusive. |
| 2. To evaluate the efficacy of ARCT-154 compared to placebo for the prevention of virologically confirmed COVID-19 at any time after first vaccination | Efficacy evaluated in all participants in the Phase 3b ITT who have received any dose of study vaccine in the first vaccination series, with no evidence of infection prior to vaccination (defined as no positive RT-PCR or other COVID-19 test or seropositivity for anti-nucleocapsid antibody prior to Day 1). The endpoint is as follows:<br>• The first occurrence of confirmed, protocol-defined COVID-19 with onset at any time after the first study vaccination and up to Day 92 inclusive. |
| 3. To evaluate the efficacy of ARCT-154 compared to placebo for the prevention of death due to COVID-19 | Efficacy evaluated in all participants in the Phase 3b mITT. The endpoint is as follows:<br>• The occurrence of death attributed to COVID-19 occurring between Day 36 and Day 92 inclusive. |
| 4. To evaluate the efficacy of ARCT-154 compared to placebo for the prevention of virologically confirmed COVID-19 regardless of baseline status for evidence of prior SARS-CoV-2 infection | Efficacy evaluated in all participants in the Phase 3b who received both protocol-required doses of study vaccine with no SARS-CoV-2 infection between Day 1 and Day 35, but including those who are seropositive at baseline (Day 1). The endpoint to be evaluated is as follows:<br>• The first occurrence of confirmed, protocol-defined COVID-19 with onset between Day 36 and Day 92 inclusive. |
| **Phase 3b Exploratory Objectives** | **Endpoint Description** |
| 1. To evaluate the efficacy of ARCT154 compared to placebo for the prevention of virologically confirmed severe COVID-19 at any time after first vaccination | Efficacy evaluated in all participants in the Phase 3b ITT who have received any dose of study vaccine in the first vaccination series, with no evidence of infection prior to vaccination (defined as no positive RT-PCR or other COVID-19 test or seropositivity for anti-nucleocapsid antibody prior to Day 1). The endpoint is as follows:<br>• The first occurrence of confirmed, protocol-defined severe COVID-19 with onset at any time after the first study vaccination and up to Day 92. |
| 2. To evaluate the efficacy of ARCT-154 compared to placebo for the prevention of death due to COVID-19 at any time after first vaccination | Efficacy evaluated in all participants in the Phase 3b ITT that have received any dose of study vaccine in the first vaccination series, with no evidence of infection prior to vaccination (defined as no positive RT-PCR or other COVID-19 test or seropositivity for anti-nucleocapsid antibody prior to Day 1). The endpoint is as follows:<br>• The occurrence of death attributed to COVID-19 occurring at any time after the first study vaccination and up to Day 92. |



*Arcturus / Cytel Confidential: This is a controlled document and shall not be changed, saved, copied or reproduced by any unauthorized person.*

| | |
|---|---|
| 3. To evaluate the efficacy of ARCT-154 compared to placebo for the prevention of virologically confirmed COVID-19 at any time after first vaccination regardless of baseline status for evidence of prior SARS-CoV-2 infection | Efficacy evaluated in all participants in the Phase 3b ITT that have received any dose of study vaccine in the first vaccination series, regardless of baseline status for evidence of prior SARS-CoV-2 infection. The endpoints are as follows:<br>• The first occurrence of confirmed, protocol-defined COVID-19 with onset at any time after the first study vaccination and up to Day 92. |
| 4. To evaluate the efficacy of ARCT-154 compared to placebo for the prevention of virologically confirmed SARS-CoV-2 by specific variant | 4a) Efficacy will be evaluated in participants in Phase 3b mITT. The endpoint to be evaluated is as follows:<br>• The first occurrence of confirmed, protocol-defined COVID-19 for all participants infected with the same variant of SARS-CoV-2, with onset between Day 36 and Day 92 inclusive<br>4b) Efficacy will also be evaluated in participants in the Phase 3b ITT that have received any dose of study vaccine in the first vaccination series, with no evidence of infection prior to vaccination (defined as no positive RT-PCR or other COVID-19 test or seropositivity for anti-nucleocapsid antibody prior to Day 1). This endpoint to be evaluated is as follows:<br>• The first occurrence of confirmed, protocol-defined COVID-19 for all participants infected with the same variant of SARS-CoV-2, with onset at any time after the first study vaccination and up to Day 92 |
| 5. To evaluate the efficacy of ARCT-154 compared to placebo for the prevention of virologically confirmed severe SARS-CoV-2 by specific variant | 5a) Efficacy will be evaluated in participants in Phase 3b ITT that have received any dose of study vaccine in the first vaccination series, with no evidence of infection prior to vaccination (defined as no positive RT-PCR or other COVID-19 test or seropositivity for anti-nucleocapsid antibody prior to Day 1).<br>The endpoint to be evaluated is as follows:<br>• The first occurrence of confirmed, protocol-defined severe COVID-19 for all participants infected with the same variant of SARS-CoV-2, with onset at any time after the first study vaccination and up to Day 92.<br>5b) Efficacy will also be evaluated in participants in Phase 3b mITT. The endpoint to be evaluated is as follows:<br>• The first occurrence of confirmed, protocol-defined severe COVID-19 for all participants infected with the same variant of SARS-CoV-2, with onset between Day 36 and Day 92 inclusive |
| *6. To evaluate an immune correlate associated with reduced risk of COVID-19* | *Blood samples drawn at Baseline and Day 57 in all participants will be held for potential future testing for evaluation of a correlate of protection.* |



*Arcturus / Cytel Confidential: This is a controlled document and shall not be changed, saved, copied or reproduced by any unauthorized person.*

## Table 3 Phase 3c Objectives and Endpoints

| Phase 3c Primary Objectives | Endpoint Description |
|---|---|
| 1. To assess the safety and reactogenicity of ARCT-154 and ChAdOx1 | Safety:<br>• Any unsolicited adverse events (AE) starting within 28 days after each study vaccine administration, summarized by severity and relationship to study vaccine.<br>• Any medically attended adverse event (MAAE), serious adverse event (SAE), or AE leading to discontinuation/withdrawal through Final Visit/Early Termination (ET) summarized by relationship to study vaccine.<br>Reactogenicity following vaccinations on Day 1 and Day 29 only:<br>• Any solicited local or systemic AE starting within 7 days after each study vaccine administration by toxicity grade. |
| *2. To evaluate noninferiority of neutralizing antibody (NAb) geometric mean concentration for ARCT-154 versus ChAdOx1 at Day 57* | *NAb responses by pseudovirus microneutralization test (MNT) evaluated at Day 57 for assessment of geometric mean concentration.*<br>• *The endpoint is the geometric mean ratio of the surrogate virus neutralization test for ARCT-154 and ChAdOx1 at Day 57* |
| **Phase 3c Secondary Objectives** | **Endpoint Description** |
| *1. To evaluate noninferiority of IgG binding antibody geometric mean concentration for ARCT-154 versus ChAdOx1 at Day 57* | *IgG antibody binding the full-length SARS-CoV-2 spike protein (binding antibody [BAb]) responses evaluated at Day 57 for assessment of geometric mean concentration.*<br>• *The endpoint is the geometric mean ratio of BAb for ARCT-154 and ChAdOx1 at Day 57* |
| *2. To evaluate noninferiority of neutralizing antibody (NAb) seroconversion rate for ARCT-154 versus ChAdOx1 at Day 57* | *NAb responses by pseudovirus microneutralization test (MNT) evaluated at Day 1 (baseline) and Day 57 for assessment of seroconversion.*<br>• *The endpoint is seroconversion rate (defined as: 4-fold increase in titer from baseline) for ARCT-154 and ChAdOx1 at Day 57* |
| *3. To evaluate noninferiority of IgG binding antibody seroconversion rate for ARCT-154 versus ChAdOx1 at Day 57* | *IgG antibody binding the full-length SARS-CoV-2 spike protein (BAb) responses evaluated at Day 1 (baseline) and Day 57 for assessment of seroconversion.*<br>• *The endpoint is seroconversion rate (defined as: 4-fold increase in titer from baseline) for ARCT-154 and ChAdOx1 at Day 57* |



*Arcturus / Cytel Confidential: This is a controlled document and shall not be changed, saved, copied or reproduced by any unauthorized person.*

| | |
|---|---|
| *4. To evaluate superiority of neutralizing antibody (NAb) geometric mean concentration for ARCT-154 versus ChAdOx1 at Day 57* | *NAb responses by pseudovirus microneutralization test (MNT) evaluated at Day 57 for assessment of geometric mean concentration.*<br>• *The endpoint is the geometric mean ratio of the MNT for ARCT-154 and ChAdOx1 at Day 57* |
| *5. To evaluate superiority of IgG binding antibody geometric mean concentration for ARCT-154 versus ChAdOx1 at Day 57* | *IgG antibody binding the full-length SARS-CoV-2 spike protein (Bab) responses evaluated at Day 57 for assessment of geometric mean concentration.*<br>• *The endpoint is the geometric mean ratio of BAb for ARCT-154 and ChAdOx1 at Day 57* |
| *6. To assess the neutralizing and IgG binding antibody immune responses for ARCT-154 and ChAdOx1 over time* | *NAb by pseudovirus microneutralization test (MNT) and IgG antibody binding the full-length SARS-CoV-2 spike protein (Bab) responses evaluated at Day 1 (baseline), Day 29, Day 57 and Day 211. Assessments for both BAb and NAb will include:*<br>• *Antibody level: GMC*<br>• *Increase in antibody levels from baseline (GMFR or other measure, as appropriate for the assay concerned)*<br>• *Geometric mean ratio (GMR)*<br>• *Proportion of participants in each study vaccine group with seroconversion at each timepoint.*<br>*Samples also collected at Day 394 and may be evaluated in the same assays* |
| **Phase 3c Exploratory Objectives** | **Endpoint Description** |
| *1. To evaluate the efficacy of ARCT154 and ChAdOx1 nCov-1 for the prevention of virologically confirmed COVID-19* | *Efficacy evaluated in participants in the Phase 3c mITT. The endpoint is as follows:*<br>• *The first occurrence of confirmed, protocol-defined COVID-19 with onset after Day 35.*<br>*Efficacy may also be evaluated in participants that have received any dose of study vaccine in the first vaccination series, with no evidence of infection prior to vaccination. The endpoint is as follows:*<br>• *The first occurrence of confirmed, protocol-defined COVID-19 with onset at any time after the first study vaccination.* |
| *2. To evaluate the efficacy of ARCT154 and ChAdOx1 nCov-1 for the prevention of virologically confirmed severe COVID-19* | *Efficacy evaluated in participants in the Phase 3c mITT. The endpoint is as follows:*<br>• *The first occurrence of confirmed, protocol-defined severe COVID-19 with onset after Day 35.*<br>*Efficacy may also be evaluated in participants that have received any dose of study vaccine in the first vaccination series, with no evidence of infection prior to vaccination. The endpoint is as follows:* |



Arcturus / Cytel Confidential: This is a controlled document and shall not be changed, saved, copied or reproduced by any unauthorized person.

| | |
|---|---|
| | • *The first occurrence of confirmed, protocol-defined severe COVID-19 with onset at any time after the first study vaccination.* |
| *3. To evaluate the efficacy of ARCT154 and ChAdOx1 nCov-1 for the prevention of death due to COVID-19* | *Efficacy evaluated in participants in the Phase 3c mITT. The endpoint is as follows:*<br>• *The first occurrence of death attributed to COVID-19 disease occurring after Day 35.*<br>*Efficacy may also be evaluated in participants that have received any dose of study vaccine in the first vaccination series, with no evidence of infection prior to vaccination. The endpoint is as follows:*<br>*The first occurrence of death attributed to COVID-19 occurring at any time after the first study vaccination.* |
| *4. To evaluate the efficacy of ARCT154 and ChAdOx1 for the prevention of virologically confirmed COVID-19 regardless of baseline status for evidence of prior SARS-CoV-2 infection* | *Efficacy evaluated in participants in the Phase 3c mITT regardless of baseline status for evidence of prior SARS-CoV-2 infection. The endpoint is as follows:*<br>• *The first occurrence of confirmed, protocol-defined COVID-19 with onset after Day 35.*<br>*Efficacy may also be evaluated in participants that have received any dose of study vaccine in the first vaccination series. The endpoint is as follows:*<br>• *The first occurrence of confirmed, protocol-defined COVID-19 with onset at any time after the first study vaccination.* |
| *5. To evaluate neutralizing antibody responses of ARCT154 and ChAdOx1 using a surrogate virus neutralization test* | *Neutralizing antibody (Nab) responses by surrogate virus neutralization test (SVNT) evaluated at individual timepoints.*<br>*The following endpoints may be assessed:*<br>• *GMC*<br>• *Increase in antibody levels from baseline (GMFR)*<br>• *Proportion of participants in each study vaccine group with seroconversion.* |
| *6. To evaluate the neutralizing antibody responses of ARCT-154 and ChAdOx1 to SARS-CoV-2 variants of concern/variants of interest* | *Neutralizing antibody (Nab) responses to SARS-CoV-2 variants of concern may be evaluated at individual time points in participants in the Phase 3c-1 IAS.* |

Objectives/Endpoints in italicized are not analyzed and more details are provided in Section 7.

## Table 4 Pooled Objectives and Endpoints

| Pooled Primary Objectives | Endpoint Description |
|---|---|



Arcturus / Cytel Confidential: This is a controlled document and shall not be changed, saved, copied or reproduced by any unauthorized person.

| | |
|---|---|
| 1. To assess the safety and reactogenicity of ARCT-154 and comparator vaccines (placebo, ChAdOx1 nCov-1) | Safety of Phase 1/2/3a/3b up to Day 92:<br>• Any unsolicited adverse events (AE) starting within 28 days after each study vaccine administration, summarized by severity and relationship to study vaccine.<br>• Any medically attended adverse event (MAAE), serious adverse event (SAE), or AE leading to discontinuation/withdrawal through Final Visit/Early Termination (ET) summarized by relationship to study vaccine.<br>Reactogenicity in Phase 1/2/3a/3b following vaccinations on Day 1 and Day 29 only:<br>• Any solicited local or systemic AE starting within 7 days after each study vaccine administration by toxicity grade. |
| 2. To evaluate the efficacy of ARCT154 compared to placebo for the prevention of virologically confirmed COVID-19 | Efficacy evaluated in all participants in the Pooled mITT. The endpoint to be evaluated is as follows:<br>• The first occurrence of confirmed, protocol-defined COVID-19 with onset between Day 36 and Day 92 inclusive. |
| **Pooled Secondary Objectives** | **Endpoint Description** |
| 1. To evaluate the efficacy of ARCT-154 compared to placebo for the prevention of virologically confirmed severe COVID-19 | Efficacy evaluated in all participants in the Pooled mITT. The endpoint to be evaluated is as follows:<br>• The first occurrence of confirmed, protocol-defined severe COVID-19 with onset between Day 36 and Day 92 inclusive. |
| 2. To evaluate the efficacy of ARCT-154 compared to placebo for the prevention of virologically confirmed COVID-19 at any time after first vaccination | Efficacy evaluated in participants in the Pooled ITT who have received any dose of study vaccine in the first vaccination series, with no evidence of infection prior to vaccination (defined as no positive RT-PCR or other COVID-19 test or seropositivity for anti-nucleocapsid antibody prior to Day 1). The endpoint is as follows:<br>• The first occurrence of confirmed, protocol-defined COVID-19 with onset at any time after the first study vaccination and up to Day 92 inclusive. |
| 3. To evaluate the efficacy of ARCT-154 compared to placebo for the prevention of death due to COVID-19 | Efficacy evaluated in all participants in the Pooled mITT. The endpoint is as follows:<br>• The occurrence of death attributed to COVID-19 occurring between Day 36 and Day 92 inclusive. |
| 4. To evaluate the efficacy of ARCT-154 compared to placebo for the prevention of virologically confirmed COVID-19 regardless of baseline status for evidence of prior SARS-CoV-2 infection | Efficacy evaluated in all participants in the Pooled population who received both protocol-required doses of study vaccine with no SARS-CoV-2 infection between Day 1 and Day 35, but including those who are seropositive at baseline (Day 1). The endpoint to be evaluated is as follows:<br>• The first occurrence of confirmed, protocol-defined COVID-19 with onset between Day 36 and Day 92 inclusive. |



Arcturus / Cytel Confidential: This is a controlled document and shall not be changed, saved, copied or reproduced by any unauthorized person.

| Pooled Exploratory Objectives | Endpoint Description |
|---|---|
| 1. To evaluate the efficacy of ARCT154 compared to placebo for the prevention of virologically confirmed severe COVID-19 at any time after first vaccination | Efficacy evaluated in all participants in the Pooled ITT who have received any dose of study vaccine in the first vaccination series, with no evidence of infection prior to vaccination (defined as no positive RT-PCR or other COVID-19 test or seropositivity for anti-nucleocapsid antibody prior to Day 1). The endpoint is as follows:<br>• The first occurrence of confirmed, protocol-defined severe COVID-19 with onset at any time after the first study vaccination and up to Day 92. |
| 2. To evaluate the efficacy of ARCT-154 compared to placebo for the prevention of death due to COVID-19 at any time after first vaccination | Efficacy evaluated in all participants in the Pooled ITT that have received any dose of study vaccine in the first vaccination series, with no evidence of infection prior to vaccination (defined as no positive RT-PCR or other COVID-19 test or seropositivity for anti-nucleocapsid antibody prior to Day 1). The endpoint is as follows:<br>• The occurrence of death attributed to COVID-19 occurring at any time after the first study vaccination and up to Day 92. |
| 3. To evaluate the efficacy of ARCT-154 compared to placebo for the prevention of virologically confirmed COVID-19 at any time after first vaccination regardless of baseline status for evidence of prior SARS-CoV-2 infection | Efficacy evaluated in all participants in the Pooled ITT that have received any dose of study vaccine in the first vaccination series, regardless of baseline status for evidence of prior SARS-CoV-2 infection. The endpoints are as follows:<br>• The first occurrence of confirmed, protocol-defined COVID-19 with onset at any time after the first study vaccination and up to Day 92. |
| 4. To evaluate the efficacy of ARCT-154 compared to placebo for the prevention of virologically confirmed SARS-CoV-2 by specific variant | Efficacy will be evaluated in participants in the Pooled mITT. The endpoint to be evaluated is as follows:<br>• The first occurrence of confirmed, protocol-defined COVID-19 for all participants infected with the same variant of SARS-CoV-2, with onset between Day 36 and Day 92 inclusive. |
| 5. To evaluate the efficacy of ARCT-154 compared to placebo for the prevention of virologically confirmed severe SARS-CoV-2 by specific variant | Efficacy will be evaluated in participants in Pooled mITT. The endpoint to be evaluated is as follows:<br>• The first occurrence of confirmed, protocol-defined severe COVID-19 for all participants infected with the same variant of SARS-CoV-2, with onset between Day 36 and Day 92 inclusive. |
| 6. To evaluate for an immune correlate associated with reduced risk of COVID-19 | Blood samples drawn at Baseline and Day 57 in all participants will be held for potential future testing for evaluation of a correlate of protection. |



*Arcturus / Cytel Confidential: This is a controlled document and shall not be changed, saved, copied or reproduced by any unauthorized person.*

# 2 STUDY DESIGN

## 2.1 Introduction to Study Design

This is a Phase 1/2/3, randomized, placebo- and active-controlled, observer-blind study designed to evaluate the safety, immunogenicity and efficacy of ARCT-154 in adult participants to be enrolled in Vietnam. The full details of the study design are available in protocol Section 4.4 and illustrated in protocol's Figure 1.

The study design included 5 different enrollment phases:

- Phase 1: healthy participants ≥18 to <60 years of age, enrolled as a sentinel cohort evaluating the safety and immunogenicity of ARCT-154 versus placebo (3:1 randomization)
- Phase 2 and 3a: healthy and "at-risk" participants ≥18 years of age, respectively, evaluating safety and immunogenicity of ARCT-154 versus placebo (3:1 randomization)
- Phase 3b: healthy and "at-risk" participants ≥18 years of age, respectively, evaluating safety and efficacy of ARCT-154 versus placebo (1:1 randomization)
- Phase 3c: healthy and "at-risk" participants ≥18 years of age, respectively, evaluating safety and immunogenicity of ARCT-154 versus authorized AstraZeneca COVID-19 vaccine (1:1 randomization)

An important element of the study design is that there is a "Switchover" or "Re-randomization" stage at Day 92 in Phase 1, 2, 3a, and 3b. At that milestone, participants who were originally assigned to receive placebo in Phases 1, 2, 3a and 3b will receive two subsequent doses of ARCT-154 on Day 92 and Day 120. Participants in Phase 1 and Phase 3b who were originally assigned to receive ARCT-154 will receive two subsequent doses of placebo on Day 92 and Day 120. Participants in Phase 2 and Phase 3a who were originally assigned to ARCT-154 will be re-randomized at 3:1 ratio either to receive third dose of ARCT-154 on Day 92 and dose of placebo on Day 120 or to receive two subsequent doses of placebo on Day 92 and Day 120. Phase 3c participants will not switchover treatments.

In all phases of this study, reactogenicity (solicited AEs) is captured by diary card; unsolicited AEs are described by verbal report, individuals in Phase 1/2/3a/3b with symptoms of COVID-19 that present prior to Day 92 are tested by reverse transcriptase polymerase chain reaction (RT-PCR) to determine if SARS-CoV-2 infection has occurred, and blood samples are drawn in all participants in all phases of study for on-study or future exploratory immunogenicity testing. For Phase 3c, nasal swabs should continue to be collected until the last study visit (early termination of Day 394).

The final analysis will be performed using a final and locked database following the approval of this SAP. Efficacy, safety, immunogenicity analyses methods will be consistent with analyses pre-specified in SAPs Part 1, Part 2 prior to unblinding for Phase 1/2/3a/3b and also with methods described in 6-month SAP with any changes and clarifications documented in this



Arcturus / Cytel Confidential: This is a controlled document and shall not be changed, saved, copied or reproduced by any unauthorized person.

final analysis SAP Section 7. Same methods will be used for Phase 3c analyses where applicable.

## 2.2 Randomization Methodology

Phase 1, 2 and 3a participants were randomly assigned 3:1 (ARCT-154:placebo), while Phase 3b participants were randomly assigned 1:1 (ARCT-154:placebo).

- Participants in Phase 1 and 3b received two doses of one type of study vaccine on Day 1 and 29 (ARCT 154 or placebo) and then two doses of the opposite vaccine (placebo or ARCT-154) on Day 92 and 120 (referred to as ‘Switchover’).
- Participants in Phase 2/3a who received ARCT-154 in the initial two-dose vaccination series were further randomized via IVRS to receive either ARCT-154 or placebo (in a 3:1 ratio) at Day 92 followed by placebo at Day 120 (referred to as “Re-randomization”). Participants that received placebo in the initial vaccination series underwent Switchover to receive ARCT-154 at Day 92 and Day 120 (referred to as “Switchover”).

Phase 3c participants were randomized 1:1 to receive ARCT-154 or ChAdOx1; no Switchover or re-randomization occurred for Phase 3c participants as all participants in this cohort receive active vaccine.

For Phases 2, 3a, 3b and 3c, prior to randomization, participants were stratified by the following 3 stratification factors:

- Age < 60 at high risk of severe COVID-19
- Age < 60 not at high risk of severe COVID-19
- Age ≥ 60 years of age (considered at risk of severe COVID-19 by default)

Participants were defined as “at risk” if they are 60 years of age or older OR have medical history described as putting an individual at risk or possibly at risk of severe coronavirus disease 2019 (COVID 19).

As the eligibility criteria for Phase 1 exclude participants ≥ 60 years of age or otherwise at risk for severe COVID-19, stratification did not occur for Phase 1.

## 2.3 Stopping Rules

An independent Data and Safety Monitoring Board (DSMB), conducted ongoing review of blinded and unblinded data, including safety and confirmed cases of COVID-19 at data review meetings.

At each meeting, the DSMB reviewed the available data and made a recommendation to the Sponsor to continue, modify, or discontinue study enrollment (while the study was enrolling). In addition to the ongoing review of safety data, the DSMB also reviewed available severe



Arcturus / Cytel Confidential: This is a controlled document and shall not be changed, saved, copied or reproduced by any unauthorized person.

COVID-19 case data to determine the risk of vaccine-associated enhanced respiratory disease (VAERD).

## 2.4 Blinding

The study vaccines were administered in an observer-blind fashion.

In order to maintain an observer-blind design, investigators, site staff, participants, and CRO staff with oversight of study conduct remained blinded to vaccine assignments for the study duration. The Sponsor and Arcturus' representatives with direct safety oversight of the study remained blinded to individual participant vaccine assignments until the time that the study is fully unblinded. The Switchover/Further Study Vaccine occurred in a blinded fashion and participants and blinded site and Sponsor personnel remained blinded to treatment assignment until End of Study (EOS).

Additional members of the Sponsor team that were not involved in direct oversight of the study may have received unblinded study data but did not share any unblinded information with the Sponsor team overseeing the study. Sponsor staff and other persons that became unblinded at the various data analysis time points were specified in a written unblinding plan prior to unblinding occurring.

Specific considerations for sponsor personnel unblinding at the time of SAP writing are described in Section 2.5 on interim analyses (IA).

A small team of individuals at Arcturus involved in the authorship of the 6-month and final SAPs was included in the list of individuals who had access to the unblinded data at the participant level through Ministry of Health (MOH) analyses (2 analyses). The first analysis was performed on the safety and immunogenicity data from participants in the Phase 1/2/3a study. The second analysis was performed on the safety, immunogenicity, and efficacy data from participants in the Phase 1/2/3a/3b portions of the study.

This was not thought to influence the development of the 6-month and final SAP which is largely streamlining the original analyses and stating specifics for the final analysis of data (more details described on Section 2.5).

No analysis of Phase 3c data was performed in the IAs, and this study part is blinded in this final analysis SAP development.

## 2.5 Interim Analyses

Per Vietnam MOH request, unblinded safety analysis including data through Day 92 and immunogenicity analysis including data through Day 57 in Phase 1/2/3a participants were performed. The immunogenicity analysis included all data to support the evaluation of the immunogenicity objectives in Phase 1/2/3a. This first analysis was used to support the emergency use authorization (EUA) in Vietnam.



Arcturus / Cytel Confidential: This is a controlled document and shall not be changed, saved, copied or reproduced by any unauthorized person.

Per Vietnam MOH request, the unblinded Phase 3b safety and efficacy analyses were performed when all participants in Phase 3b that were evaluable for the primary efficacy endpoint had reached Day 92 and all potential COVID-19 events in these participants up to Day 92 had been adjudicated by the blinded Independent Adjudication Committee. This second analysis was also used to support the EUA in Vietnam.

An additional 6-month safety analysis was performed when all participants in Phase 1/2/3a/3b had at minimum of 6-month safety follow-up. The 6-month safety analysis also included all analyses of efficacy and immunogenicity previously conducted per Vietnam MOH requests, as well as analyses of results generated by using additional immunogenicity assays. In addition, the 6-month analysis was the subject of the interim CSR and provided updated safety (Phase 1/2/3a/3b) and immunogenicity (Phase 1/2/3a) data. The 6-month analysis is a CDISC-compliant implementation of previous MOH analyses with updated safety data including up to 6-month follow-up and additional immunogenicity analysis to support the ex-Vietnam regulatory submissions. Version 1.0 of the SAP for this analysis was finalized and signed on 02 December 2022. In order to clarify analysis and align with recommendations provided during an European Medicines Agency pre-submission interaction discussion on 31 January 2023, the SAP was revised on 10 March 2023 after extraction of the data for the analyses, which occurred on 12 January 2023. The data cut point for immunogenicity and safety for this analysis was 12 January 2023 when all participants in Phases 1, 2, 3a, and 3b had been followed up for at least 6 months after the 2nd vaccination (or had terminated early). The data cut point for VE remained the same (i.e., Day 92 visit) as that for the earlier analysis, ie, 30 March 2022. Immunogenicity analyses were presented up to Day 120, which was the last timepoint at which immunogenicity is assessed prior to the EOS (Day 394). Safety data were presented up to 6 months after the 2nd dose.

Since not all associated data were fully cleaned, these analyses are still considered as interim. However no multiplicity adjustments will be made for these and subsequent final analyses, as the final analyses are reflective of the MOH and 6-month analyses using the same set of endpoint data.

<u>Phase 1/2/3a</u>

The primary immunogenicity analysis for Phase 1/2/3a was performed after all participants in this cohort reached the Day 57 timepoint.

The primary Phase 1/2/3a analysis of safety was performed when all participants in this cohort reached Day 92.

First interim analysis of safety data through at least Day 57 were performed for Phase 1/2/3a at the time of analysis of the primary immunogenicity endpoint of Phase 1/2/3a to support application for EUA in Vietnam.

Safety data before Day 57 and efficacy data before Day 92 were entered into the EDC, verified and validated where possible prior to the data extractions for the interim analyses for EUA.



*<u>Arcturus / Cytel</u> Confidential: This is a controlled document and shall not be changed, saved, copied or reproduced by any unauthorized person.*

Randomization was opened for the subgroup of participants who were further randomized to receive ARCT-154 or placebo at Day 92, in order to conduct Day 120 analyses to evaluate safety and immunogenicity following the third dose. Sponsor and CRO staff responsible for day-to-day conduct of the study will remain blinded until the final unblinding of data at the end of study occurs in order that safety assessments can continue in an unbiased fashion.

Phase 3b

The primary Phase 3b analysis of safety and efficacy were performed when all participants in Phase 3b that were evaluable for the primary efficacy analysis had reached Day 92 and all COVID-19 events in these participants up to Day 92 had been adjudicated by the blinded Independent Adjudication Committee.

Subsequent analyses of safety would be exploratory. However, Sponsor and CRO staff responsible for day-to-day conduct of the study would remain blinded until the final unblinding of data at the end of study occurs in order that safety assessments can continue in an unbiased fashion. SAP Part 1 and Part 2 were finalized prior to the second of two interim analyses performed by Vinbiocare in April 2022 although the SAPs were finalized and the analyses were intended for execution by another vendor, Biophics. The plan was partially implemented by Vinbiocare due to the severe COVID-19 outbreak in Vietnam and the interest in a faster and focused review of the data.

The analyses that were therefore performed by Vinbiocare included the following features:

- They were requested by the Vietnam MOH as part of the ongoing review of the ARCT-154 program for consideration of an Emergency Use Authorization.
- The first analysis performed by Vinbiocare focused on the safety and immunogenicity data from participants in the Phase 1/2/3a study.
- A small team of individuals at Vinbiocare and Vietstar were unblinded for this analysis. These individuals did not contribute to the finalization of both SAP Part 1 and Part 2.
- The second analysis performed by Vinbiocare focused on the safety, immunogenicity, and efficacy data from participants in the Phase 1/2/3a/3b portions of the study. This analysis was also performed in collaboration with Vietstar. The analysis was aligned with, but did not follow, all elements of SAP Part 2.



Arcturus / Cytel Confidential: This is a controlled document and shall not be changed, saved, copied or reproduced by any unauthorized person.

# 3 STUDY ENDPOINTS

Study endpoints corresponding to protocol objectives assessed as part of the final analysis are briefly described in Table 1, Table 2, Table 3, and Table 4 and are covered in more details within analysis methods in Table 6, Table 7, and Table 8.



Arcturus / Cytel Confidential: This is a controlled document and shall not be changed, saved, copied or reproduced by any unauthorized person.

# 4 ANALYSIS SETS

## 4.1 Analysis Set Definitions

- **All Screened Participants:** Includes all participants who signed informed consent form. This analysis sets are defined for each Phase of the study as follows:
  - Phase 1/2/3a: this pools data from Phase 1, 2 and 3a participants
  - Phase 3b
  - Phase 3c
  - Pooled: this pools data from Phase 1, 2, 3a and 3b participants
- **Randomized Set (RS):** Includes all participants who are randomly assigned in the study regardless of the participants' vaccination status in the study. Participants will be analyzed according to the vaccine to which the participant was randomly assigned. RS are defined for each Phase of the study as follows:
  - Phase 1/2/3a RS: this pools data from Phase 1, 2 and 3a participants
  - Phase 1 RS
  - Phase 2/3a RS
  - Phase 3b RS
  - Phase 3c RS
  - Pooled RS: this pools data from Phase 1, 2, 3a and 3b participants

  Participants who are entered into the unblinded part of IVRS will receive a random ID (randomID). Participants who received a randomID will be entered into the blinded part of the IVRS to receive the treatment assignment. Participants with an assigned randomID but without assigned treatment in the IVRS system will not be considered as randomized.
- **Intent-to-Treat (ITT) Analysis Set:** Includes all participants who receive any dose of study vaccine (ARCT-154 or placebo or ChAdOx1). Participants will be analyzed according to the vaccine to which the participant was randomly assigned. ITT are defined for each Phase of the study as follows:
  - Phase 3b ITT
  - Phase 3c ITT
  - Phase 1/2/3a ITT: this pools data from Phase 1, 2 and 3a participants and will include evaluations up to Day 92 only
  - Pooled ITT: this pools data from Phases 1/2/3a/3b and will include evaluations up to Day 92 only


Arcturus / Cytel Confidential: This is a controlled document and shall not be changed, saved, copied or reproduced by any unauthorized person.

- **Modified Intent-to-Treat (mITT) Analysis Set:** Includes all participants who received all protocol-required doses of study vaccine (ARCT-154 or placebo or ChAdOx1) up to the evaluation timepoint concerned, and who have no evidence of SARS-CoV-2 infection (no positive RT-PCR or other COVID-19 test or seropositivity for anti-nucleocapsid antibody) on Day 1 or up to 7 days after the 2nd study vaccination. The mITT analysis set will be analyzed according to vaccine assigned. The mITT analysis sets are defined for:
    - Phase 3b mITT
    - Phase 1/2/3a mITT
    - Phase 3c mITT
    - Pooled mITT: this pools data from Phases 1/2/3a/3b and will include evaluations up to Day 92 only
- **Per-Protocol (PP) analysis Set:** Includes all eligible randomized participants who received all protocol-required doses of study vaccine (ARCT-154 or placebo) up to the evaluation timepoint concerned and within the protocol predefined window, and who have no major protocol deviations expected to affect efficacy (PP efficacy), immunogenicity (PP immunogenicity), safety or reactogenicity assessments as determined by the Sponsor Medical Monitor or designee in a blinded manner. The PP analysis set will be analyzed according to which study vaccine was assigned, in the event there is a discrepancy. PP sets will be further derived for efficacy and immunogenicity as follows:
    - **Efficacy**
        - Phase 3b PP efficacy set: includes per protocol Phase 1 participants and excludes any participant with evidence of SARS-CoV-infection on Day 1 up to 7 days after the 2nd study vaccination (IcEv3) or that receives an non-study COVID-19 vaccine prior to Day 92 (IcEv2).
        - Pooled PP efficacy set: includes per protocol Phase 1, 2, 3a and Phase 3b participants and excludes any participant with evidence of SARS-CoV-infection on Day 1 up to 7 days after the 2nd study vaccination (IcEv3) or that receives an non-study COVID-19 vaccine prior to Day 92 (IcEv2).
    - **Immunogenicity**
        - Phase 1 PP immunogenicity set: includes per protocol Phase 1 participants and excludes any participant with evidence of SARS-CoV-2 infection on Day 1 up to Day 57 (IcEv3) or who receives a non-study COVID-19 vaccine prior to the analysis time point concerned (IcEv2).
        - Phase 2/3a PP immunogenicity set: includes per protocol Phase 2 and Phase 3a participants and excludes any participant with evidence of SARS-CoV-2 infection on Day 1 up to Day 57 (IcEv3) or who receives a



Arcturus / Cytel Confidential: This is a controlled document and shall not be changed, saved, copied or reproduced by any unauthorized person.

non-study COVID-19 vaccine prior to the analysis time point concerned (IcEv2).

- Phase 1/2/3a PP immunogenicity set (or Phase 1/2 PP immunogenicity set): includes per protocol participants from Phase 1, 2 and Phase 3a participants and excludes any participant with evidence of SARS-CoV-2 infection on Day 1 up to Day 57 (IcEv3) or who receives an non-study COVID-19 vaccine prior to the analysis time point concerned (IcEv2). This set will include evaluations of immunogenicity up to Day 92 only. Of note, this analysis set will be restricted to Phase 1/2 participants for analysis of parameters not collected in phase 3a participants and will be referred to as Phase 1/2 PP immunogenicity set)

PP immunogenicity sets will be derived up to the analysis time point concerned based on the availability of each assay. Three time points are considered:

- Up to Day 57
- Up to Day 92
- Up to Day 120

The following major protocol deviations are expected to affect efficacy and will lead to exclusion of a participant from the PP efficacy set:

- Received wrong study vaccine up to Day 92
- Did not receive study vaccine
- Received non-study COVID-19 vaccine (IcEv2) up to Day 92
- COVID-19 infection (positive RT-PCR or other COVID-19 test) up to Day 36
- Result of SARS-CoV-2 anti-nucleocapsid antibodies test is Positive/Undetermined up to Day 36

The following major protocol deviations are expected to affect immunogenicity and will lead to exclusion of a participant from the PP immunogenicity sets:

- Received wrong study vaccine up to the time point concerned
- Did not receive study vaccine
- Received non-study COVID-19 vaccine (IcEv2) up to the time point concerned
- Received confounding medication (IcEv5) up to the time point concerned
- COVID-19 infection (positive RT-PCR or other COVID-19 test) up to Day 57
- Result of SARS-CoV-2 anti-nucleocapsid antibodies test is Positive/Undetermined up to Day 57

All major protocol deviations listed above are conditions leading to exclusion from the PP set specified in the protocol.



*Arcturus / Cytel Confidential: This is a controlled document and shall not be changed, saved, copied or reproduced by any unauthorized person.*

- **Immunogenicity Analysis Set (IAS):** Includes all participants who received all protocol-required doses of study vaccine (ARCT-154 or placebo) up to the evaluation timepoint concerned, who have no evidence of prior SARS-CoV-2 infection (no positive RT-PCR or other COVID-19 test or seropositivity for anti-nucleocapsid antibody) up to Day 57 and who have at least 1 valid post-vaccination immunogenicity assay result.

All participants in the IAS will be analyzed according to which study vaccine was assigned.

IAS are defined for the following Phases of the study:

  - Phase 1 IAS: this corresponds to data from participants in Phase 1
  - Phase 2/3a IAS: this pools data from participants in Phase 2 and 3a
  - Phase 1/2/3a IAS (or phase 1/2 IAS): this pools data from Phase 1, 2 and 3a participants and will include evaluations of immunogenicity up to Day 92 only. Of note, this analysis set may be restricted to Phase 1 and Phase 2 participants for analyses on parameters not collected for phase 3a participants.

- **Safety Analysis Set (SAS):** Includes all participants who receive any dose of study vaccine (ARCT-154 or placebo or ChAdOx1). Participants will be analyzed according to the study vaccine received. SAS analysis sets are defined for each Phase of the study as follows:
  - Phase 1/2/3a SAS: this pools data from Phase 1, Phase 2 and Phase 3a participants
  - Phase 3b SAS
  - Phase 3c SAS
  - Pooled SAS: this pools data from Phase 1/2/3a/3b for ARCT-154 and placebo, respectively. It will include evaluations up to Day 92 only (execept for unsolicited events)

- **Reactogenicity Analysis Set (RAS):** Includes all participants who receive any dose of study vaccine (ARCT-154 or placebo or ChAdOx1) and provide at least 1 reactogenicity diary report. Participants will be analyzed according to the vaccine received. Reactogenicity analysis sets are defined for each Phase of the study as follows:
  - Phase 1/2/3a RAS: this pools data from Phase 1, Phase 2 and Phase 3a participants
  - Phase 2/3a RAS
  - Phase 3b RAS
  - Phase 3c RAS
  - Pooled RAS: this pools data from Phase 1/2/3a/3b for ARCT-154 and placebo, respectively.



Arcturus / Cytel Confidential: This is a controlled document and shall not be changed, saved, copied or reproduced by any unauthorized person.

For safety analysis sets, SAS and RAS, where participants will be analyzed according to the study vaccine received, in case a participant receives incorrect study vaccine, only data up to the receipt of the incorrect study vaccine (exclusive) will be used in the summaries. If the first dose of vaccine the participant received was the incorrectly assigned one, then only data up to the second dose (exclusive) will be used in the summaries. Outputs for “any dose” that analyze data after any ARCT-154 dose or after any Placebo or ChAdOx1 dose, will include all data after each dose even if participant received an incorrect study vaccine.

Separate listings of safety and immunogenicity data, that are currently planned in this SAP, will be produced for participants receiving at least one incorrect dose of study vaccine. Of note, participants with missing dosing will not be considered as receiving incorrect dosing.

## 4.2 Protocol Deviations

A deviation from the protocol is an unintended or unanticipated departure from the procedures or processes approved by the Sponsor and the IRB/IEC and agreed to by the investigator. A significant deviation occurs when there is non adherence to the protocol or to local regulations or ICH GCP guidelines that may or may not result in a significant, additional risk to the participant or impacts the integrity of study data.

The process for determining which deviations would result in participants being removed from the PP analysis set for immunogenicity is described in Section 4.1. Derivations for exclusion from PP analysis set will be re-programmed for the final analysis using the final locked data from EDC.

A summary of major protocol deviations, and the reasons for exclusion from efficacy and immunogenicity analyses will be presented by treatment group in the Randomized Set, and detailed data will be provided in the listing.



Arcturus / Cytel Confidential: This is a controlled document and shall not be changed, saved, copied or reproduced by any unauthorized person.

# 5 DATA HANDLING

## 5.1 Computing Environment

All statistical analyses will be performed using SAS statistical software (Version 9.4), unless otherwise noted. Medical History and adverse events will be coded using Medical Dictionary for Regulatory Activities (MedDRA) version 24.0. Concomitant medications will be coded using World Health Organization (WHO) Drug Dictionary version March 2022.

## 5.2 Data Conventions

- **Day 1:** The date of first dose of study vaccine administration
- **Day 92:** refers to Day 92 visit unless otherwise specified in efficacy Section 6.4
- **Unscheduled or repeated visits:** Unscheduled visits results will be listed, but not included in tables or graphs.
- 1 year = 365.25 days
- **Values below the lower limit of quantification (LLOQ):** Any immunogenicity parameters which are below LLOQ will be listed, and LLOQ/2 will be used for summaries. Additional analyses of seroconversion with a different imputation of LLOQ value will be performed as previously requested by US FDA.
- **Values above the upper limit of quantification (ULOQ):** Any immunogenicity parameters which are above ULOQ will be listed, for summaries ULOQ will be used. For Neutralizing antibody (NAb) responses by surrogate virus neutralization test (sVNT) as performed by National Institute of Hygiene and Epidemiology, Department of Virology, Vietnam (NIHE), ULOQ will be 6000 IU/mL.
- (Absolute) Change from baseline = Value at the time point – Baseline value, or specified otherwise.

## 5.3 Reporting of data across studies and Output Displays

The data collected in phase 1, phase 3b and phase 3c will be reported separately as applicable. In addition, the following pooling will be performed as applicable:

- Phase 2/3a; Phase 2 and 3a will be pooled together, where data are available for each of the phases
- Phase 1/2/3a: Phase 1, 2 and 3a will be pooled together, where data are available for each of the phases
- Pooled: Phase 1, 2, 3a, 3b will be pooled together, where data are available for each of the phases



Arcturus / Cytel Confidential: This is a controlled document and shall not be changed, saved, copied or reproduced by any unauthorized person.

### 5.3.1 Output Displays

**Phase 1 and Phase 3b:**

- Baseline and disposition outputs:

| ARCT-154 (Initial) | Placebo (Initial) | Total |
|---|---|---|

- Analysis of data up to the Switchover/Further study vaccine or after any dose:

| ARCT-154 (Initial) | Placebo (Initial) |
|---|---|

- Analysis of data after Switchover/Further study vaccine

| ARCT-154 (Initial)/<br>Placebo (Dose 3 and 4) | Placebo (Initial)/<br>ARCT-154 (Dose 3 and 4) |
|---|---|

**Phase 1/2/3a, Phase 2/3a, Pooled:**

Since participants underwent Switchover at Day 92 or were re-randomized (participants initially randomized to ARCT-154 in Phase 2 and 3a) at Day 92 and then received Placebo at Day 120, headers by dose will be presented in the following way:

- Baseline and disposition outputs:

| ARCT-154 (Initial) | Placebo (Initial) | Total |
|---|---|---|

- Analysis of data up to the Switchover/Further study vaccine:

| ARCT-154 (Initial) | Placebo (Initial) |
|---|---|

- Analysis of data after Switchover/Further study vaccine

| ARCT-154 (Initial) | | Placebo (Initial) |
|---|---|---|
| ARCT-154 (Dose 3)/<br>Placebo (Dose 4) | Placebo<br>(Dose 3 and 4) | ARCT-154<br>(Dose 3 and 4) |

- Analysis of combined data before and after Switchover/Further study vaccine:

| ARCT-154 (Initial) | | | Placebo (Initial) |
|---|---|---|---|
| ARCT-154 (Dose 3)/ | Placebo | Not Re-randomized | ARCT-154 |



Arcturus / Cytel Confidential: This is a controlled document and shall not be changed, saved, copied or reproduced by any unauthorized person.

| | | | |
|---|---|---|---|
| Placebo (Dose 4) | (Dose 3 and 4) | | (Dose 3 and 4) |

- Analysis of data over multiple doses

ARCT-154 vaccine at any time; no ARCT-154 vaccine:

| | |
|---|---|
| ARCT-154 | Placebo |

- Analysis of data from the first dose of ARCT-154 vaccine administration:

| |
|---|
| ARCT-154 |

**Phase 3c**

- Baseline and disposition outputs:

| | | |
|---|---|---|
| ARCT-154 | ChAdOx1 | Total |

- Other outputs:

| | |
|---|---|
| ARCT-154 | ChAdOx1 |

## 5.4 Withdrawals, Dropouts, Loss to Follow-up

Participants who discontinue from the study vaccine after randomization will not be replaced.

## 5.5 Visit Windows

Visit window from the target day is applied to the following visits (Study day) for analyses in the PP set:

- A window of ± 30 days: Study Day 92 (Switchover/Further study vaccine). As per protocol, for participants in Phase 1 and Phase 3b, the visit window at Day 92 is +30 days while for participants in Phase 2 and Phase 3a, the visit window at Day 92 is +14 days." For consistency across studies, the same time window will be applied for all studies.
- A window of ± 14 days: Study Day 394 (Final visit)
- A window of ± 7 days for other scheduled visits

Only assessments that fit within the protocol defined visit windows will be used in the analysis of efficacy and immunogenicity for PP analysis set.



Arcturus / Cytel Confidential: This is a controlled document and shall not be changed, saved, copied or reproduced by any unauthorized person.

# 6 STATISTICAL METHODS

## 6.1 Sample Size Justification

The sample size for the study overall is driven by the sum of the sample size assumptions for the individual phases of the study, as outlined in the subsections below.

**Sample Size for Phase 1/2/3a Substudy**

Data from Phases 1, 2 and 3a will be pooled up to Day 92 for safety analyses, which will be performed in the Phase 1/2/3a SAS and Phase 1/2/3a RAS. The total sample size for Phase 1/2/3a is primarily driven by the size of safety database required at the time of potential EUA application. With approximately 750 participants randomized in Phases 1, 2 and 3a to receive ARCT-154 for the Phase 1/2/3a primary safety analysis, if the incidence rate of an adverse event is 1.0%, the probability to detect one event in 750 vaccinated participants is >99%, based upon the following formula:

$$p = 1 - (1 - R)^N$$

where R = incidence rate and N = sample size

Data from Phases 1, 2 and 3a will be pooled up to Day 92 for immunogenicity analyses, which will be performed in the Phase 1/2/3a IAS. The primary immunogenicity endpoint is defined as the proportion of participants in each study vaccine group that demonstrate seroconversion (defined as: 4-fold increase in titer from baseline) by surrogate virus NAb assay at Day 57.

The null hypothesis is $H0^{Immunogenicity}$: lower bound 95% CI for $SC^{154} \leq SC^{Placebo}$. A sample size of 750 and 250 participants in the ARCT-154 and placebo groups respectively will provide greater than 90% power to exclude the null hypothesis with a 1-sided type 1 error rate of 0.025 assuming that 10% of participants are excluded from the analysis (for example due to baseline seropositivity), and that $SC^{154}$ >50% and $SC^{Placebo}$ ≤10%. However, a more conservative hypothesis testing has been added (refer to section 6.2.3 for more details).

**Sample Size for Phase 3b**

The primary safety endpoints will be evaluated in the Phase 3b SAS and the Phase 3b RAS. With a sample size of ~16,000 participants randomized and with approximately 8,000 participants randomized in the study to receive ARCT-154 for the primary safety analysis, if the incidence rate of an adverse event is 0.1%, the probability to detect one event in 8,000 vaccinated participants is >99.9%, based upon the following formula:

$$p = 1 - (1 - R)^N$$

where R = incidence rate and N = sample size

For the overall primary efficacy objective of the study, the null hypothesis is that the vaccine efficacy (VE) of ARCT-154 to prevent first occurrence of polymerase chain reaction (PCR)-confirmed COVID-19 is ≤30% (ie, $H0^{efficacy}$: VE ≤0.3).



Arcturus / Cytel Confidential: This is a controlled document and shall not be changed, saved, copied or reproduced by any unauthorized person.

The primary efficacy objective will be met if the lower limit of the 95% CI for VE exceeds 30%.

VE is defined as the percent reduction in the hazard of the primary endpoint (ARCT-154 versus placebo). Equivalently, the null hypothesis is as follows:

- $H0^{efficacy}$: HR >0.7 (equivalently, proportional hazards VE ≤0.3)

A Cox proportional hazard model will be used to assess the magnitude of the study group difference (ie, HR) between ARCT-154 and placebo at a 1-sided 0.025 significance level.

Under the assumption of proportional hazards over time and with 1:1 randomization of ARCT-154 and placebo, a total of 372 COVID-19 cases will provide approximately 90% power to detect a 50% reduction in hazard rate (50% VE), rejecting the null hypothesis with a 1-sided false positive error rate of 0.025.

The study will be considered positive at the primary analysis if the one-sided p-value for rejecting HR ≥ 0.7 is less than or equal to 0.025.

**Sample Size for Phase 3c Substudy**

The primary safety endpoints of Phase 3c will be conducted in the Phase 3c Safety Analysis Set and Phase 3c Reactogenicity Analysis Set. With a sample size of approximately 2,400 participants randomized and with approximately 1,200 participants randomized in the study to receive ARCT-154 or ChAdOx1 for the safety analysis, based upon the following formula, if the incidence rate of an adverse event is 0.1%, the probability to detect one event in 1,200 vaccinated participants is 69.9% and if the incidence rate is 1.0% the probability is more than 99%:

$$p = 1 - (1 - R)^N$$

where R = incidence rate and N = sample size

## 6.2 General Statistical Methods

### 6.2.1 General Methods

All outputs will be incorporated into RTF files, sorted and labeled according to the International Conference on Harmonization (ICH) recommendations, and formatted to the appropriate page size(s).

Tabulations will be produced for appropriate demographic, baseline, efficacy, immunogenicity and safety parameters. For categorical variables, summary tabulations of the number and percentage within each category (with a category for missing data) of the parameter will be presented. For continuous variables, the mean, median, standard deviation, 1st and 3rd quartiles, minimum and maximum values will be presented. All percentages will be presented to one decimal place. Two-sided 95% confidence intervals will be produced where applicable, unless otherwise stated.



Arcturus / Cytel Confidential: This is a controlled document and shall not be changed, saved, copied or reproduced by any unauthorized person.

### 6.2.2 Definition of Baseline

Baseline is defined as the last non-missing assessment prior to the first dose of study vaccine administration. If an assessment is collected on the day of first dose of study vaccine administration and time of the dose or assessment is not available, such assessment will be considered for the derivation of baseline. Assessments labeled as 30 minutes post-dose and 3 hours post-dose will not be considered for the baseline derivation, even if the time is not available.

### 6.2.3 Statistical Hypothesis

**Phase 1/2/3a Substudy Statistical Hypothesis**

As per protocol, the primary immunogenicity objective (assessment of ARCT NAb responses by surrogate virus neutralization test (sVNT) at Day 57 versus Placebo) was to be assessed by means of the following Hypotheses:

- Null hypothesis ($H0^{Immunogenicity}$): lower bound of the 95% CI of ARCT-154 seronverstion rate at Day 57 ($SC^{154}$) ≤ Placebo seroconversion rate at Day 57 ($SC^{Placebo}$); i.e., $H0^{Immunogenicity}$: lower bound 95% CI for $SC^{154} \leq SC^{Placebo}$ versus,
- Alternative hypothesis ($H1^{Immunogenicity}$): lower bound of the 95% CI of $SC^{154} > SC^{Placebo}$

Phase 1/2/3a part of the study was to be considered to meet its primary immunogenicity objective if the lower bound of the 95%CI for $SC^{154} > SC^{Placebo}$. Following regulatory interactions, a more conservative methodology has been added:

- $H0^{Immunogenicity}$: lower bound of the 95% CI of ($SC^{154}$-$SC^{Placebo}$) ≤ 0% , versus
- $H1^{Immunogenicity:}$ lower bound of the 95% CI of ($SC^{154}$-$SC^{Placebo}$) > 0%

The 95% CI for the difference of seroconversion rates at Day 57 between the two groups (ARCT-Placebo) will be calculated using the Miettinen and Nurminen method. Two-sided $\alpha$= 0.05 will be used for all tests

There is no hypothesis testing for safety data analysis. Where statistical methods are applied, the emphasis will be on estimation with 95% CIs.

**Phase 3b Statistical Hypothesis**

The overall primary endpoints for the study are evaluated in the Phase 3b population as follows:

- The overall primary efficacy endpoint for the study is vaccine efficacy as evaluated in the Phase 3b Modified Intent to Treat (mITT) population
- The overall primary safety endpoints are those evaluated in the Phase 3b Safety Analysis Set (SAS) and Phase 3b Reactogenicity Analysis Set (RAS)

There is no hypothesis testing for safety data analysis. Where statistical methods are applied, the emphasis will be on estimation with 95% CIs.



Arcturus / Cytel Confidential: This is a controlled document and shall not be changed, saved, copied or reproduced by any unauthorized person.

The primary statistical analyses for VE will be performed using virologically confirmed COVID-19 cases as adjudicated in a blinded fashion by the Independent Adjudication Committee.

The null hypothesis ($H0^{Efficacy}$) as follows: $H0^{Efficacy}$: VE ≤ 30%

VE is estimated as 1-HR, where HR is the hazard ratio, and will be presented together with 95% CIs. The primary efficacy objective will be met if the lower limit of the 95% CI for VE exceeds 30% in the Phase 3b mITT population.

Under the assumptions of proportional hazards over time and a 50% reduction in hazard rate (50% VE) and with 1:1 randomization of ARCT-154 and placebo, a total of 372 confirmed COVID-19 cases will provide approximately 90% power to reject the null hypothesis (H0: VE ≤ 30%), using a log-rank test statistic with a 1-sided false positive error rate of 0.025.

The secondary efficacy objectives (and second primary objective for regulatory filings outside of Vietnam) will be met if the lower limit of the 95% CI for VE exceeds 0%.

**Phase 3c Substudy Statistical Hypotheses**

There is no hypothesis testing for primary Phase 3c safety objective.

### 6.2.4 Multiple Comparisons/Multiplicity

The overall primary efficacy objective for the study is determination of VE as evaluated in the Phase 3b mITT population. Hence the study will be regarded to have met the primary objective if the null hypothesis is rejected. Hence the primary efficacy objective will be met, and the study overall declared to be positive, if the lower limit of the 95% confidence interval (CI) for VE exceeds 30%. VE is defined as the percent reduction in the hazard of the primary endpoint (ARCT-154 versus placebo).

The overall key secondary objectives for the study are Phase 3b Secondary Endpoints 1 and 2. These endpoints will be evaluated in a hierarchical fashion such that Secondary Endpoint 1 will only be evaluated in a hypothesis testing fashion if the overall primary objective of the study has been met and Secondary Endpoint 2 will only be evaluated in a hypothesis testing fashion if the null hypothesis for Secondary Endpoint 1 has been rejected.

Phase 3b secondary endpoints 3 and 4 will be evaluated in a hypothesis generating fashion with any statistical significance being nominal.

For the pooled analyses, endpoints are nominally declared as primary/secondary/exploratory, but all endpoints are evaluated as sensitivity analyses to the equivalent endpoints being assessed in the Phase 3b populations.

Phase 1/2/3a and subgroups thereof constitute a distinct substudy with its own distinct objectives and endpoints. There is no testing procedure for controlling type 1 error for Phase 1/2/3a substudy primary or secondary endpoints. As this is a substudy, and there is no evaluation of



Arcturus / Cytel Confidential: This is a controlled document and shall not be changed, saved, copied or reproduced by any unauthorized person.

efficacy for this substudy, no adjustment of type 1 error is applied to the overall efficacy primary endpoint.

### 6.2.5 Subgroups

The following subgroups will be assessed for some analyses as presented below:

- Age group (≥ 18 to < 60 years, ≥ 60 years) (based on data collected in CRF)
- Risk group (≥ 18 to < 60 years and “Healthy”, ≥ 18 to < 60 years and “At risk”, ≥ 60 years) (based on data collected in CRF)
- Sex (Male, Female)
- Region (North HMU, North VMMU, South PasteurHCM)
- SARS-Cov2 variants: Delta, Omicron, Not determined (refer to section 6.4.3.4)
- Study site

| Site No. | Site ID* | Site Name | Region |
|---|---|---|---|
| 1 | 101, 201 | HMU | North HMU |
| 2 | 202, 302, 402 | PMC Yen Phong | North HMU |
| 3 | 203, 303, 403, 503 | PMC Vinh Long | South PasteurHCM |
| 4 | 404, 504 | PMC Long Ho | South PasteurHCM |
| 5 | 405, 505 | PMC Cai Be | South PasteurHCM |
| 6 | 406, 506 | PMC Cho Gao | South PasteurHCM |
| 7 | 407, 507 | PMC Cao Lanh | South PasteurHCM |
| 8 | 408, 508 | PMC Lap Vo | South PasteurHCM |
| 9 | 409 | PMC Van Lam | North VMMU |
| 10 | 410 | PMC Van Giang | North VMMU |
| 11 | 411 | PMC Phu Cu | North VMMU |
| 12 | 412 | PMC Tien Lu | North VMMU |
| 13 | 413 | PMC Kim Dong | North VMMU |



Arcturus / Cytel Confidential: This is a controlled document and shall not be changed, saved, copied or reproduced by any unauthorized person.

| Site No. | Site ID* | Site Name | Region |
|---|---|---|---|
| 14 | 414 | PMC An Thi | North VMMU |
| 15 | 415 | PMC Khoai Chau | North VMMU |
| 16 | 416 | PMC Hung Yen | North VMMU |
| 17 | 517 | PMC Quang Xuong | North VMMU |

*The first digit of the site ID refers to phases of the study: 1=Phase 1, 2=Phase 2, 3=Phase 3a, 4=Phase 3b, and 5=Phase 3c

Selected disposition, demography, exposure, reactogenicity (refer to section 6.6.1.1 for further details), unsolicited AEs (refer to section 6.6.1.2 for further details) and SAEs outputs (refer to section 6.6.1.3 for further details) will be produced by age group, risk group, sex and study site subgroups.

Primary and secondary efficacy endpoints will be assessed by risk group, sex, region and study site subgroups. Please refer to Section 6.4.1.1.

Selected Immunogenicity analyses will be produced by age group, risk group and sex (refer to section 6.5.6 for further details)

### 6.2.6 Missing, Unused, and Spurious Data

Missing data will not be imputed and will be analyzed as if they were missing completely at random unless otherwise specified. Missing demographic data will be presented as missing and will not be included in the denominator for calculation of proportion. Methods to handle missing data for specific analysis will be described in safety, efficacy and immunogenicity sections.

For handling of missing and partial AE dates and medication dates, please refer to Section 6.6.1 and Section 6.3.3, respectively.

### 6.2.7 Intercurrent Events

**Table 5 Intercurrent Events**

| Label | Intercurrent Event (IcEv) Type |
|---|---|
| IcEv1 (Death not due to COVID-19) | Death due to any cause other than COVID-19 associated. |



*Arcturus / Cytel Confidential: This is a controlled document and shall not be changed, saved, copied or reproduced by any unauthorized person.*

| | |
|---|---|
| IcEv2 (Non-study COVID-19 vaccine) | Use of non-study COVID-19 vaccine. |
| IcEv3 (Early infection) | Positive reverse transcriptase-polymerase chain reaction (RT-PCR) test or other COVID-19 positive test or seropositivity for anti-nucleocapsid antibody indicating exposure to SARS-CoV-2 prior to vaccination or SARS-CoV-2 infection prior to 7 days (inclusive) after the second study vaccination dose (Day 35). |
| IcEv4 (Study infection) | Develops virologically confirmed SARS-CoV-2 infection, with or without COVID-19 symptoms (Positive RT-PCR or other COVID-19 positive test or seropositivity for anti-nucleocapsid antibody indicating exposure to SARS-CoV-2) more than 7 days after second study vaccine dose (≥Day 36). Additional details on how to define primary efficacy endpoint is presented in Section 6.4. |
| IcEv5 (Confounding On-study Medications) | Receives any of the following concomitant medications/vaccines prior to the time point for which the analysis is being performed:<br>• Live vaccines within 28 days before or after any study vaccination<br>• Systemic (oral, intravenous, intramuscular or subcutaneous) corticosteroids given at any study time point prior to the timepoint at which the analysis is being performed<br>• Blood products or immunoglobulins given at any study time point prior to the timepoint at which the analysis is being performed<br>• Immunosuppressive medications, including cytotoxic medication for cancer or autoimmune disease, given at any study time point prior to the timepoint at which the analysis is being performed |

Abbreviations: COVID-19=coronavirus disease 2019; IcEv=intercurrent event; SARS-CoV-2=severe acute respiratory syndrome coronavirus 2.

## 6.3 Study Population

### 6.3.1 Participant Disposition, Analysis Sets

Summaries of participant disposition will be prepared for all participants for randomized set, including the number and percent screened, randomized, and administered study vaccine at each dose. The reasons for screen failures and the number that withdrew from study treatment and from the study along with the reasons will be summarized and listed. Participant disposition will also be presented separately for Day 1 to Day 91 and Day 92 to EOS.

The reasons for exclusion from different analysis sets, and termination from study will be summarized and listed.



Arcturus / Cytel Confidential: This is a controlled document and shall not be changed, saved, copied or reproduced by any unauthorized person.

The number of participants in each analysis set will be summarized in the randomized set.

The summaries will be presented by vaccine group and overall. The denominator for all percentages will be the total number of participants in each vaccine group.

The following by-participant listings will be presented.

- Study completion information, including the reason for premature study withdrawal.
- Inclusion/exclusion criteria
- Inclusion in study analysis sets
- Protocol deviations
- Reasons for exclusion from the analysis set and if they completed the study.

Disposition table for Phase 3b SAS will be also produced by subgroups as specified in Section 6.2.5.

### 6.3.2 Demographic and Baseline Characteristics

Baseline demographics and characteristics, including age, height, weight, sex, race, ethnicity, region of study sites, age group, risk group, and body mass index (BMI) will be summarized. These will be summarized for the mITT, SAS, RS and IAS by vaccine group and total of all participants. If the number of participants included in the PP analysis set or RAS analysis set is ≥10% different from mITT or the SAS, it will also be presented.

Demography table for Phase 3b SAS will be also produced by subgroups as specified in Section 6.2.5.

A listing of individual demographics will be provided.

Medical history data will be presented by System/Organ/Class (SOC) and Preferred Term (PT) for SAS.

### 6.3.3 Prior and Concomitant Medication

Prior and Concomitant medications will be coded using the WHO Drug dictionary.

Prior medications will be defined as any medication with a start date before the date of first dose of study vaccine administration. If a medication date is missing, or partially missing, and it cannot be determined whether it was taken prior or concomitant to treatment, it will be considered a prior and concomitant medication.

Medications with a start date on or after the date of first dose of study vaccine administration and not after the last dose of study vaccine administration will be considered concomitant.

Medications taken prior to the first dose of study vaccine administration and continuing after the first dose of study vaccine administration will be considered both prior and concomitant.



Arcturus / Cytel Confidential: This is a controlled document and shall not be changed, saved, copied or reproduced by any unauthorized person.

Prior and Concomitant medications results will be tabulated by ATC and preferred term.

The use of Prior and Concomitant medications will be included in by-participant data listing.

### 6.3.4 Exposure and Compliance

Vaccine exposure and compliance are considered in analysis set definitions. The reasons a vaccine dose was delayed will be summarized.

Vaccine exposure table for Phase 3b SAS will be also produced by subgroups as specified in Section 6.2.5.

Participants receiving incorrect/unplanned treatment will be presented in a listing for planned versus actual treatment.

### 6.3.5 Serostatus by SARS-CoV-2 nucleocapsid antibody test

Seropositivity status by SARS-CoV-2 nucleocapsid antibody test will be listed for the randomized set.

## 6.4 Efficacy Evaluation

Each efficacy evaluation will be performed for Phase 3b, Phase 1/2/3a, and Pooled (data from Phase 1/2/3a/3b) separately. Analysis will be performed on the mITT analysis set unless stated otherwise. Efficacy estimands are described in Section 9.2.2.

**Table 6 Overview of Efficacy Analysis**

| Reference to Protocol Endpoints (a) | Endpoint/Event Description* | Analysis Set | Time at Risk** | Subgroups |
|---|---|---|---|---|
| Primary<br>Exploratory 2 (Phase 1/2/3a) | First occurrence of confirmed, protocol-defined COVID-19 with onset between Day 36 and Day 92 | mITT (3b & 1/2/3a & Pooled)<br>PP (3b & Pooled) | [Last date at risk – (Date received 2nd dose + 7 days)]+1 | Yes, for mITT (3b) |
| Secondary 1 | Severe COVID-19 with onset between Day 36 and Day 92 inclusive | mITT (3b & 1/2/3a Pooled)<br>PP (3b & Pooled) | [Last date at risk – (Date received 2nd dose + 7 days)]+1 | Yes, for mITT (3b) |



Arcturus / Cytel Confidential: This is a controlled document and shall not be changed, saved, copied or reproduced by any unauthorized person.

| Reference to Protocol Endpoints (a) | Endpoint/Event Description* | Analysis Set | Time at Risk** | Subgroups |
|---|---|---|---|---|
| Secondary 2 | COVID-19 with onset at any time after the first dose of study vaccine administration and up to Day 92 | ITT + no prior infection<br>(3b & 1/2/3a & Pooled) | [Last date at risk – Date received 1st dose]+1 | No |
| Secondary 3 | Death attributed to COVID-19 occurring between Day 36 and Day 92 | mITT (3b & 1/2/3a & Pooled)<br>PP (3b & Pooled) | [Last date at risk – (Date received 2nd dose + 7 days)]+1 | Yes, for mITT (3b) |
| Secondary 4 | COVID-19 with onset between Day 36 and Day 92 | Participants with both doses of study vaccine and no infection between Day 1 and Day 35, but including those who are seropositive at baseline (Day 1) (3b & 1/2/3a & Pooled) | [Last date at risk – (Date received 2nd dose + 7 days)]+1 | Yes, for 3b |
| Exploratory 1 | Severe COVID-19 with onset at any time after the first dose of study vaccine administration and up to Day 92 | ITT + no prior infection (3b & 1/2/3a & Pooled) | [Last date at risk – Date received 1st dose]+1 | No |
| Exploratory 2 | Death attributed to COVID-19 occurring at any time after the first dose of study vaccine administration and up to Day 92 | ITT + no prior infection (3b & 1/2/3a & Pooled) | [Last date at risk – Date received 1st dose]+1 | No |
| Exploratory 3 | COVID-19 with onset at any time after the first dose of study vaccine administration and up to Day 92 | ITT (3b & 1/2/3a & Pooled) | [Last date at risk – Date received 1st dose]+1 | No |



Arcturus / Cytel Confidential: This is a controlled document and shall not be changed, saved, copied or reproduced by any unauthorized person.

| Reference to Protocol Endpoints (a) | Endpoint/Event Description* | Analysis Set | Time at Risk** | Subgroups |
|---|---|---|---|---|
| Exploratory 4a | COVID-19 with onset between Day 36 and Day 92 by specific variant | mITT (3b & Pooled) | [Last date at risk – Date received 1st dose]+1 | No |
| Exploratory 4b | COVID-19 with onset at any time after the first dose of study vaccine administration and up to Day 92 | ITT + no prior infection<br>(3b & Pooled) | [Last date at risk – Date received 1st dose]+1 | No |
| Exploratory 5a | Severe COVID-19 with onset between Day 36 and Day 92 by specific variant | mITT (3b & Pooled) | [Last date at risk – Date received 1st dose]+1 | No |
| Exploratory 5b | Severe COVID-19 with onset at any time after the first dose of study vaccine administration and up to Day 92 | ITT + no prior infection<br>(3b & Pooled) | [Last date at risk – Date received 1st dose]+1 | No |

*COVID-19 events will be confirmed, protocol-defined cases. ** Where the last date at risk will be defined as COVID-19-event date or censor date, whichever comes first.
(a) Unless specified otherwise, reference to the protocol endpoints is a reference to the Phase 3b endpoints.

Confirmed, protocol-defined COVID-19 cases will be determined by case report form (CRF) data, as confirmed by the blinded Independent Adjudication Committee and adjudicated for severity, with the date of case onset as date of symptom onset and date of positive COVID test date, whichever occurs first. Both protocol-defined and (protocol-defined) severe cases flagged in the CRF will be included in the primary endpoint.

For participants with no covid-event during the time interval the censor date for the last date at risk is whichever of the following comes first:

- Date of completion of Day 92 visit;
- Date of death unrelated to COVID-19;
- Date of receipt of Switchover or receipt of non-study COVID-19 vaccine
- Lost to Follow-up (last known alive date or date of Day 1+91 days, which is earlier).

Section 9.2.2 presents the endpoints and analyses in terms of estimands for primary, secondary and exploratory efficacy analyses.



*Arcturus / Cytel Confidential: This is a controlled document and shall not be changed, saved, copied or reproduced by any unauthorized person.*

As a sensitivity analysis of the primary endpoint (Phase 3b only), severe cases and deaths due to COVID-19 will be repeated using all COVID-19 cases from CRF, including those cases that are not confirmed by the blinded Independent Adjudication Committee and with severe or death severity as reported in the CRF.

Day 36 is defined as second dose+7 days (29+7=36 days), and Day 92 is defined as second dose+63 days (29+63=92 days). Another sensitivity analysis of the primary endpoint (Phase 3b only) will be performed using Day 92 visit instead of second dose+63 days.

COVID-19 cases confirmed by the blinded Independent Adjudication Committee will be presented as frequency table by time intervals of Day 1-Day 35 and Day 36-Day 92 for RS, ITT, mITT and PP. Severe COVID-19 and death due to COVID-19 by the blinded Independent Adjudication Committee will also be presented.

Number of suspected COVID-19 cases reported from Day 1 to Day 92, those tested with any COVID testing within 14 days after symptoms onset, and Proportion of participants who had COVID testing performed within 14 days after symptoms onset from Day 1 to Day 92 will be presented the Randomized Set.

### 6.4.1 Primary Efficacy Analysis

The analysis of the first primary endpoint will consider the first occurrence of confirmed, protocol-defined COVID-19 with onset between Day 36 and Day 92 inclusive.

Time at risk = [Last date at risk – (Date received 2nd dose + 7 days)]+1; where the last date at risk will be defined as COVID-19-event date or censor date, whichever comes first.

Summaries of vaccine efficacy will display the number of participants included in the analysis, number of protocol-defined COVID-19 cases (Protocol Appendix 2) and surveillance time in person-year will be summarized in each vaccine group. Surveillance time refers to the total person-time at risk (years) for the given endpoint.

The vaccine efficacy (VE) is defined as the percent reduction in the hazard rate of the COVID-19 events (ARCT-154 versus placebo). The vaccine efficacy is calculated as:

$$VE = 1 - \frac{h_{ARCT-154}}{h_{placebo}} = 1 - HR$$

where $h_{\text{ARCT-154}}$ and $h_{\text{placebo}}$ are hazard rates of the confirmed, protocol-defined COVID-19/Severe COVID-19/death among those in ARCT-154 group and placebo group, respectively.

Vaccine efficacy will be displayed together with 95% confidence interval (CI). Vaccine efficacy is calculated from 1-hazard ratio, where, the hazard ratio (HR) and 95% CI are measured by adjusted Cox proportional hazard regression, with the assumptions of proportional hazards over time. The p-value will be calculated from Cox proportional hazard regression.



Arcturus / Cytel Confidential: This is a controlled document and shall not be changed, saved, copied or reproduced by any unauthorized person.

Efficacy summaries will also include the proportion of participants with COVID-19 cases in each vaccine group will be displayed together with the Clopper-Pearson 95% CI and a comparison between ARCT-154 and Placebo with 95% CIs. A Log-Rank test p-value will be calculated (in addition to VE% Cox model p-value).

Factors to be used as covariates in Cox proportional hazard regression include:

- Risk group: A randomization stratification factor for age and health risk for severe disease; ≥18 to <60 years and "healthy", ≥18 and <60 years and "at risk" and ≥60 years.Age and health risk at randomization collected in the CRF will be used.
- Study site region: Study site is a randomization stratification factor. However, the sites are grouped into study site region for adjustment factor. Site collected in the CRF will be used.
    - Risk group or study site region will be removed from the model when performing subgroup analysis on that variable.

A Kaplan-Meier plot will be provided for the primary endpoint.

#### 6.4.1.1 Sensitivity and Subgroup Analysis

The Phase 3b and Pooled primary, 1st secondary (severe) and the 3rd secondary (deaths) endpoints' analyses will be repeated for the following analysis:

- PP Analysis Set
- All COVID-19 cases from CRF using mITT

As indicated in Table 6, Primary and secondary endpoints assessments of different phases (3b or 1/2/3a or Pooled) will be repeated for below subgroups. A forest plot of VE% will be provided for each subgroup analysis

- Risk group
- Sex
- Region
- Study site

### 6.4.2 Secondary Analysis

The analysis of secondary endpoints will follow the same methods of primary analysis. Subgroup analysis will be performed for each endpoint and phase (3b, 1/2/3a and 1/2/3/3b) as indicated in Table 6. Kaplan-Meier plots will be provided for each endpoint.

#### 6.4.2.1 Severe COVID-19

The first occurrence of confirmed, protocol-defined severe COVID-19 with onset between Day 36 and Day 92 inclusive. This endpoint includes confirmed, protocol-defined severe COVID-19



Arcturus / Cytel Confidential: This is a controlled document and shall not be changed, saved, copied or reproduced by any unauthorized person.

and death due to confirmed, protocol-defined COVID-19, as confirmed by the blinded Independent Adjudication Committee and adjudicated for severity.

Time at risk = [Last date at risk – (Date received 2nd dose + 7 days)]+1; where the last date at risk will be defined as COVID-19-event date or censor date, whichever comes first.

The analysis will follow the same methods as for the first primary endpoint.

#### 6.4.2.2 COVID-19 Any Time After Vaccination (Without Prior COVID-19)

The first occurrence of confirmed, protocol-defined COVID-19 with onset at any time after the first dose of study vaccine administration and up to Day 92 inclusive; ITT who have received any dose of study vaccine in the first vaccination series, with no evidence of infection (i.e. positive RT-PCR test or seropositivity for anti-nucleocapsid antibody) prior to vaccination on Day 1.

Time at risk = [Last date at risk – Date received 1st dose]+1; where the last date at risk will be defined as COVID-19-event date or censor date, whichever comes first.

#### 6.4.2.3 Death Due to COVID-19

The occurrence of death attributed to COVID-19 occurring between Day 36 and Day 92 inclusive.

Time at risk = [Last date at risk – (Date received 2nd dose + 7 days)]+1; where the last date at risk will be defined as date of death attributed to COVID-19 or censor date, whichever comes first. The corresponding onset date of COVID-19 will be between Day 36 and Day 92 inclusive.

#### 6.4.2.4 COVID-19 After Both Vaccines and Regardless of Baseline SARS-CoV-2 Serostatus

The efficacy of ARCT-154 and placebo for the prevention of virologically confirmed COVID-19 regardless of baseline serostatus for evidence of prior SARS-CoV-2 infection with onset between Day 36 and Day 92 inclusive, based on participants who received both protocol-required doses of study vaccine with no SARS-CoV-2 infection between Day 1 and Day 35, but including those who are seropositive at baseline (Day 1).

Time at risk = [Last date at risk – (Date received 2nd dose + 7 days)]+1; where the last date at risk will be defined as COVID-19-event date or censor date, whichever comes first.

### 6.4.3 Exploratory Analysis

The analysis of exploratory endpoints will follow the same methods of primary analysis and will be performed for Phase 3b and Pooled. Kaplan-Meier plots will be provided for each endpoint.



Arcturus / Cytel Confidential: This is a controlled document and shall not be changed, saved, copied or reproduced by any unauthorized person.

#### 6.4.3.1 Severe COVID-19 After First Vaccination

The first occurrence of confirmed, protocol-defined severe COVID-19 with onset at any time after the first dose of study vaccine administration and up to Day 92; ITT that have received any dose of study vaccine in the first vaccination series, with no evidence of infection (i.e. positive RT-PCR test or seropositivity for anti-nucleocapsid antibody) prior to vaccination.

Time at risk = [Last date at risk – Date received 1st dose]+1; where the last date at risk will be defined as COVID-19-event date or censor date, whichever comes first.

#### 6.4.3.2 COVID-19 Death After First Vaccination

The occurrence of death attributed to COVID-19 occurring at any time after the first dose of study vaccine administration and up to Day 92; ITT that have received any dose of study vaccine in the first vaccination series, with no evidence of SARS-CoV-2 infection (i.e. positive RT-PCR test or seropositivity for anti-nucleocapsid antibody) prior to vaccination.

Time at risk = [Last date at risk – Date received 1st dose]+1; where the last date at risk will be defined as death date or censor date, whichever comes first.

#### 6.4.3.3 COVID-19 After First Vaccination

The first occurrence of confirmed, protocol-defined COVID-19 with onset at any time after the first dose of study vaccine administration and up to Day 92; ITT that have received any dose of study vaccine in the first vaccination series.

Time at risk = [Last date at risk – Date received 1st dose]+1; where the last date at risk will be defined as COVID-19-event date or censor date, whichever comes first.

#### 6.4.3.4 COVID-19 by SARS CoV-2 variants

The first occurrence of confirmed, protocol-defined COVID-19 with onset between Day 36 and Day 92 inclusive will be presented by SARS COV-2 variants as frequency table for mITT. The first occurrence of confirmed, protocol-defined COVID-19 for all participants infected with the same variant (Delta, Omicron, Not undetermined) of SARS-CoV-2, with onset between Day 36 and Day 92 inclusive, will be analyzed for mITT.

Similar analysis will be performed for the first occurrence of confirmed, protocol-defined COVID-19, with onset at any time after the first study vaccination and up to Day 92, based on ITT that have received any dose of study vaccine in the first vaccination series, with no evidence of infection prior to vaccination (defined as no positive RT-PCR or other COVID-19 test or seropositivity for anti-nucleocapsid antibody prior to Day 1 vaccination).



*Arcturus / Cytel Confidential: This is a controlled document and shall not be changed, saved, copied or reproduced by any unauthorized person.*

#### 6.4.3.5 Severe COVID-19 by SARS CoV-2 variants

The first occurrence of confirmed, protocol-defined severe COVID-19 with onset between Day 36 and Day 92 inclusive will be presented by SARS COV-2 variants as frequency table for mITT. The first occurrence of confirmed, protocol-defined severe COVID-19 for all participants infected with the same variant (Delta, Omicron, Not determined) of SARS-CoV-2, with onset between Day 36 and Day 92 inclusive, will be analyzed for mITT.

Similar analysis will be performed for the first occurrence of confirmed, protocol-defined severe COVID-19, with onset at any time after the first study vaccination and up to Day 92, based on ITT that have received any dose of study vaccine in the first vaccination series, with no evidence of infection prior to vaccination (defined as no positive RT-PCR or other COVID-19 test or seropositivity for anti-nucleocapsid antibody prior to Day 1 vaccination).

## 6.5 Immunogenicity Evaluations

The immunogenicity assays were measured at 2 different laboratories:

1. Assays performed at National Institute of Hygiene and Epidemiology (NIHE), Department of Virology, Vietnam. These are regarded as protocol defined primary endpoints for analysis of immune responses in Phase 1/2/3a.
    a. Serum NAb titer against SARS-CoV-2:
        i. Genscript SARS-CoV-2 sVNT
        ii. PRNT50
            1. Ancestral-clinical isolate
            2. Delta-clinical isolate
    b. Serum IgG antibodies binding the SARS-CoV-2 spike protein (BAb)-Siemens Advia platform
2. Validated Assays performed at PPD BioA Labs in Richmond, VA, USA. These are regarded as supportive endpoints for the analysis of immune responses in Phase 1/2/3a.
    a. Serum NAb titer against SARS-CoV-2: Pseudovirus microneutralization test (MNT)
        i. D614G VAC62 (validated)
        ii. Delta VAC120 (validated)
        iii. Omicron BA.1 VAC122 (validated)
    b. BAb by Mesoscale Discovery (MSD) multiplex: Ancestral V72 (validated)



*Arcturus / Cytel Confidential: This is a controlled document and shall not be changed, saved, copied or reproduced by any unauthorized person.*

c. Angiotensin-converting enzyme 2 (ACE2)/sVNT (exploratory): Multiplex variants VAC114

Even though the sVNT assay performed at National Institute of Hygiene and Epidemiology (NIHE) was defined on the protocol as primary, following regulatory interactions it was agreed to use a validated assay (i.e. MNT performed at PPD) for this investigation. Appendix 9.3 provides an overview of the assay measured in the study along with analysis sample timepoints available for each phase.

Immunogenicity data from participants in Phases 1, 2 and 3a will be pooled for analysis up to Day 92 and will include participants who received the first two doses of study vaccine (referred as 2 dose schedule). Participants in Phase 2 and 3a that received ARCT-154 in the initial vaccination series are further randomized to receive a 3rd vaccination of either ARCT-154 or placebo at Day 92 followed by placebo at Day 120. Therefore, after Day 92 only data from Phase 2/3a will be pooled (referred as 3 dose schedule). Additional analyses will be performed to report Phase 1 data up to day 120 separately. Phase 3c immunogenicity samples were not tested and therefore no immunogenicity analysis will be performed on the phase 3c participants (refer to section 7.1 for rationale).



Arcturus / Cytel Confidential: This is a controlled document and shall not be changed, saved, copied or reproduced by any unauthorized person.

Immunogenicity analyses will be performed on Immunogenicity analysis sets as defined in section 4.1. Sensitivity analyses will be performed on the immunogenicity PP sets as defined in section 4.1; selection of participants included in the analyses on the PP sets at various analysis timepoints will be performed as described in section 5.5.

The primary immunogenicity estimand for the Phase 1/2/3a substudy along with handling of intercurrent events is described in section 9.2.1. For analyses performed on Phase 1/2/3a sets (two dose schedule), participant's immunogenicity data after switchover/receipt of further study vaccine, non-study COVID-19 vaccine (Intercurrent event 2) or confounding on-study medications (intercurrent event 5) will be excluded from the analysis. For analysis performed on Phase 1 and Phase 2/3a (three dose schedule) sets, participant's immunogenicity data after non-study COVID-19 vaccine or confounding on-study medications will be excluded from the analysis.

Section 6.5.1 provides an overview of the immunogenicity analyses while sections 6.5.2 to 6.5.6 provides a description of analyses (including analysis sets and timepoints) that will be performed for each parameter. Section 6.5.7 described immunogenicity analyses performed by subgroup.

The following analyses and definitions will be used. No p-values will be calculated:

- Number and proportion (along with Exact Clopper-Pearson 95% CI) of participants achieving antibody seroconversion as defined below (FDA defined), by timepoint and treatment arm:
    - For participants with Day 1 (baseline) antibody concentration < LLOQ, seroconversion is defined as ≥ 4-fold increase from LLOQ.
    - For participants with Day 1 antibody concentration ≥ LLOQ, seroconversion defined as ≥ 4-fold increase from Day 1 antibody concentration

  An additional definition (protocol defined) of seroconversion will be applied as follows:
    - For participants with Day 1 (baseline) antibody concentration < LLOQ, seroconversion defined as ≥ 4-fold increase from LLOQ/2.
    - For participants with Day 1 (baseline) antibody concentration ≥ LLOQ, seroconversion defined as ≥ 4-fold increase from Day 1 antibody concentration

Seroconversion at D120 will be assessed using both Day 1 and Day 92 as reference.

- Geometric Mean Concentration (or Titer) (GMC or GMT) and corresponding 95% CI, minimum and maximum concentration, by timepoint and treatment arm
- GMC (or GMT) ratio of ARCT-154 versus placebo and corresponding 95% CI by timepoint (not applicable for three dose-schedule analysis)
- Geometric Mean Fold Rise (GMFR) and corresponding 95% CI at each timepoint, from Day 1 (and additionally from Day 92 for Day 120 where applicable) by treatment arm.



Arcturus / Cytel Confidential: This is a controlled document and shall not be changed, saved, copied or reproduced by any unauthorized person.

Reverse cumulative functions will be presented for each assay by treatment arm and post baseline timepoint. Antibody levels will be presented on x axis in log10 scale.

Geometric mean concentration (GMC) is the geometric mean of the antibody concentration and is derived as follows:

$$GMC = ln^{-1}\left(\frac{\sum_{i=1}^{n} lnX}{\mathrm{n}}\right), where\ X: antibody\ concentration$$

The GMC ratio at timepoint D is the ratio of ARTC-154 GMC obtained at timepoint D over placebo GMC obtained at timepoint D.

$$GMC\ ratio\ at\ Day\ D = \frac{ARCT - 154\ GMC\ at\ Day\ D}{Placebo\ GMC\ Day\ D}$$

GMFR is defined as the geometric mean of fold rise of post-study intervention concentrations over the Day 1 (baseline) concentration. These will be calculated as follows:

$$\mathrm{GMFR} = \ln^{-1}\left(\frac{\sum_{i=1}^{n} \ln(\frac{X2}{X1})}{n}\right),$$

where X2 is the antibody concentration at time point of evaluation and X1 is the antibody concentration at baseline (or Day 92).

Handling of immune response concentration below LLOQ or greater than ULOQ is described in section 5.2.

Conversion factors to be applied to immunogenicity parameters are summarized in section 9.4.

### 6.5.1 Overview of Immunogenicity Analyses

Table 7 presents an overview of the immunogenicity analyses that will be performed. A table summarizing the disposition of participants for immunogenicity samples will be provided for each immunogenicity assay.



Arcturus / Cytel Confidential: This is a controlled document and shall not be changed, saved, copied or reproduced by any unauthorized person.

## Table 7 Overview of Primary, Secondary and Exploratory Immunogenicity Analyses

| Laboratory/Assay | Reference to Phase 1/2/3a Protocol Endpoints | Summary Level | Main/ Supportive | Variant/antigen | Analyzed Time-points | Analysis Set (a) (b) | Subgroup |
|---|---|---|---|---|---|---|---|
| NIHE/NAb by sVNT | Primary | SC rate | Main | Ancestral | Day 57 | Phase 1/2/3a IAS (Two dose schedule) | Yes |
| NIHE/NAb by sVNT | Secondary 1 | GMC; GMFR; SC rate | Main | Ancestral | Day 1, Day 29, Day 57, Day 92 | Phase 1/2/3a IAS (Two dose schedule) | Yes |
| NIHE/IgG bAb | Secondary 1 | GMC; GMFR; SC rate | Main | Ancestral | Day 1, Day 29, Day 57, Day 92 | Phase 1/2/3a IAS (Two dose schedule) | Yes |
| PPD/IgG bAb by MSD multiplex[(c)] | | GMC; GMFR; SC rate | Supportive for Secondary enpoint 1 (Serum IgG bAb) | Ancestral | Day 1, Day 57 | Phase 1/2 IAS (Two dose schedule) | |
| NIHE/NAb by PRNT50 | Secondary 2 | GMC; GMFR; SC rate | Main | Ancestral-clinical isolate variant (ancestral variant) | Day 1, Day 29, Day 57 | Phase 1/2 IAS (Two dose schedule) | |
| NIHE/NAb by PRNT50 | Exploratory (d) | GMC; GMFR; SC rate | Main | Delta-clinical isolate variant | Day 1, Day 57 | Phase 1 IAS | |
| PPD/NAb by ACE2/sVNT | Exploratory 3 | GMC; GMFR; SC rate | Main | S, S_B.1.1.7, S_B.1.351, S_B.1.526.1, S_B.1.617, S_B.1.617.1, S_B.1.617.2, S_B.1.617.3, S_P1, S_P2 | Day 1, Day 57 | Phase 1/2 IAS (Two dose schedule) | |
| PPD/NAb by ACE2/sVNT | Exploratory (d) | GMC; GMFR; SC rate | Main | S, S_B.1.1.7, S_B.1.351, S_B.1.526.1, S_B.1.617, S_B.1.617.1, S_B.1.617.2, S_B.1.617.3, S_P1, S_P2 | Day 1, Day 57 | Phase 1 IAS | |



Arcturus / Cytel Confidential: This is a controlled document and shall not be changed, saved, copied or reproduced by any unauthorized person.

| Laboratory/Assay | Reference to Phase 1/2/3a Protocol Endpoints | Summary Level | Main/ Supportive | Variant/antigen | Analyzed Time-points | Analysis Set (a) (b) | Subgroup |
|---|---|---|---|---|---|---|---|
| PPD/NAb by MNT | Exploratory 3,4 | GMC; GMFR; SC rate | Main | D614G | Day 1, Day 57, Day 92, Day 120 | Phase 1/2/3a IAS (Two dose schedule)<br>Phase 2/3a IAS (Three dose schedule)<br>Phase 1 IAS | |
| | Exploratory 3,4 | GMC; GMFR; SC rate | | Delta | Day 1, Day 57, Day 92, Day 120 | Phase 2/3a IAS (Two dose schedule)<br>Phase 2/3a IAS (Three dose schedule) | |
| | Exploratory 3,4 | GMC; GMFR; SC rate | | Omicron BA.1 | Day 1, Day 57, Day 92, Day 120 | Phase 2/3a IAS (Two dose schedule)<br>Phase 2/3a IAS (Three dose schedule) | |
| NIHE/NAb by sVNT | Exploratory 5 | GMC; GMFR; SC rate | Main | Ancestral | Day 1, Day 29, Day 57, Day 92, D120 | Phase 2/3a IAS (Three dose schedule) | |
| NIHE/NAb by sVNT | Exploratory (d) | GMC; GMFR; SC rate | Main | Ancestral | Day 1, Day 29, Day 57, Day 92 | Phase 1 IAS | |
| NiHE/IgG bAb | Exploratory 6 | GMC; GMFR; SC rate | Main | | Day 1, Day 57, Day 92, Day 120 | Phase 2/3a IAS (Three dose schedule) | |
| NIHE/IgG bAb | Exploratory (d) | GMC; GMFR; SC rate | Main | | Day 1, Day 57, Day 92 | Phase 1 IAS | |

(a) IAS includes all participants who received all protocol-required doses of study vaccine (ARCT-154 or placebo) up to the evaluation timepoint concerned. Two Dose Schedule or Three Dose Schedule means that IAS for the analysis of the endpoint includes participants who received the first two or three doses of study vaccine, respectively.
(b) sensitivity analyses will be performed on the PP set (immunogenicity) for the same pooling as IAS.



Arcturus / Cytel Confidential: This is a controlled document and shall not be changed, saved, copied or reproduced by any unauthorized person.

(c) Assessment of serum IgG bAb by MSD multiplex assessment is not a secondary objective but is considered as a supportive analysis for the secondary objective Serum IgG bAb (by NIHE).(d) As per protocol, no immunogenicity objectives are defined so immunogenicity analyses performed on phase 1 are considered exploratory.



Arcturus / Cytel Confidential: This is a controlled document and shall not be changed, saved, copied or reproduced by any unauthorized person.

### 6.5.2 Neutralizing antibody (NAb) responses to ARCT-154 by surrogate virus neutralization test (sVNT)

Neutralizing antibody (NAb) responses by surrogate virus neutralization test (sVNT) as performed by National Institute of Hygiene and Epidemiology, Department of Virology, Vietnam (NIHE), evaluated at Day 1 (baseline) and Day 57 for assessment of seroconversion (based on seroconversion FDA definition provided in section 6.5) in all participants in the Phase 1/2/3a IAS is considered as the_Phase 1/2/3a Substudy Primary Immunogenicity Objective (Primary Immunogenicity estimand as described in appendix 9.2). The hypotheses to be tested are described in section 6.2.3.

The number and proportion (and its 95% CI) of participants achieving seroconversion at Day 57 will be provided for each treatment. The two-sided 95% CI of the seroconversion rate will be calculated using the Clopper-Pearson method. In addition, the treatmentdifference, ARCT-154-Placebo, in proportion of participants achieving seroconversion at Day 57 will be provided, along with corresponding two-sided 95% CI (calculated using the Miettinen and Nurminen method)

The following formula, which is provided in the assay kit by the manufacture, will be used for converting sVNT NAb titer (U/mL) to standard unit (IU/mL):

Result (IU/mL) = Result (NAb titer)/12.3

Neutralizing antibody (Nab) responses by sVNT as performed by NIHE will also be evaluated by GMC, GMFR (not applicable for Day 1), and seroconversion rate (not applicable for Day 1) for the following analysis sets and timepoints where applicable:

- Phase 1 IAS: Day 1, Day 29, Day 57, Day 92.
- Phase 1/2/3a IAS: Day 1, Day 29, Day 57, Day 92 (Phase 1/2/3a Substudy Secondary Immunogenicity Objective)
- Phase 2/3a IAS: Day 1, Day 29, Day 57, Day 92, Day 120 (Phase 1/2/3a Substudy Exploratory Immunogenicity Objective)

Neutralizing antibody (Nab) responses by sVNT, as performed by PPD Laboratories, USA, (Angiotensin-converting enzyme 2 (ACE2)/sVNT) will be evaluated by GMC, GMFR, and seroconversion rate for the following analysis sets and timepoints where applicable:

- Phase 1 IAS: Day 1, Day 57.
- Phase 1/2 IAS: Day 1, Day 57 (Phase 1/2/3a Substudy Exploratory Immunogenicity Objective)

Supportive analyses will be performed in the corresponding per protocol (PP) immunogenicity sets. Subgroup analyses will be performed as described in section 6.5.7.



Arcturus / Cytel Confidential: This is a controlled document and shall not be changed, saved, copied or reproduced by any unauthorized person.

### 6.5.3 Early neutralizing antibody responses using a live virus assay: NAb responses by plaque reduction neutralization test at 50% reduction (PRNT50)

NAb responses by plaque reduction neutralization test at 50% reduction (PRNT50), as performed by NIHE will be evaluated for immune responses to SARS-CoV-2 D614G variant by GMC, GMFR, and seroconversion rate for the following analysis sets and timepoints where applicable:

- Phase 1 IAS: Day 1 (baseline), Day 29 and Day 57.
- Phase 1/2 IAS: Day 1 (baseline), Day 29 and Day 57 (Phase 1/2/3a Substudy Secondary Immunogenicity Objective)

Sensitivity analyses will be performed in the per protocol (PP) immunogenicity sets.

NAb responses by plaque reduction neutralization test at 50% reduction (PRNT50), as performed by NIHE will be evaluated for immune responses to SARS-CoV-2, Delta variant by GMC, GMFR, and seroconversion rate for the following analysis sets and timepoints where applicable:

- Phase 1 IAS: Day 1 (baseline), Day 29 and Day 57.
- Phase 1/2 IAS: Day 1 (baseline), Day 29 and Day 57 (Phase 1/2/3a Substudy Exploratory Immunogenicity Objective)

Sensitivity analyses will be performed in the per protocol (PP) immunogenicity sets.

### 6.5.4 IgG antibody binding the SARS-CoV-2 spike protein (binding antibody [BAb])

IgG antibody binding the SARS-CoV-2 spike protein (binding antibody [BAb]), as performed by NIHE will be evaluated for assessment of by GMC, GMFR, and seroconversion rate for the following analysis sets and timepoints where applicable:

- Phase 1 IAS: Day 1, Day 29, Day 57, Day 92
- Phase 1/2/3a IAS: Day 1, Day 29, Day 57, Day 92 (Phase 1/2/3a Substudy Secondary Immunogenicity Objective)
- Phase 2/3a IAS: Day 1, Day 29, Day 57, Day 92, Day 120 (Phase 1/2/3a Substudy Exploratory Immunogenicity Objective)

Sensitivity analyses will be performed in the per protocol (PP) immunogenicity sets.

### 6.5.5 Neutralizing antibody (NAb) responses to ARCT-154 by pseudovirus microneutralization test (MNT)

NAb responses by microneutralization test (MNT), as performed by PPD Laboratories, USA, will be evaluated for immune responses to SARS-CoV-2 variants, including D614G (VAC62), Delta (VAC120) and Omicron BA.1 (VAC122) variants by GMC, GMFR, and seroconversion rate for the following analysis sets and timepoints where applicable:



Arcturus / Cytel Confidential: This is a controlled document and shall not be changed, saved, copied or reproduced by any unauthorized person.

- D614G VAC62 (validated):
  - Phase 1 IAS: Day 1 and Day 57
  - Phase 1/2/3a IAS: Day 1 and Day 57, Day 92 (Phase 1/2/3a Substudy Exploratory Immunogenicity Objective)
  - Phase 2/3a IAS: Day 1 and Day 57, Day 92 and D120 (Phase 1/2/3a Substudy Exploratory Immunogenicity Objective)
- Delta VAC120 (validated):
  - Phase 2/3a IAS: Day 1 and Day 57, Day 92 and D120 (Phase 1/2/3a Substudy Exploratory Immunogenicity Objective)
- Omicron BA.1 VAC122 (validated):
  - Phase 2/3a IAS: Day 1 and Day 57, Day 92 and D120 (Phase 1/2/3a Substudy Exploratory Immunogenicity Objective)

Sensitivity analyses will be performed in the per protocol (PP) immunogenicity sets.

For D614G VAC62, the following formula will be used for converting results in AU/mL into standard units (IU/mL): AU/mL is 1 IU/mL = 1.275 AU/mL. Results in both units will be presented.

### 6.5.6 Serum IgG antibodies binding the SARS-CoV-2 spike by Mesoscale Discovery (MSD) multiplex assay

IgG antibody binding the SARS-CoV-2 spike protein (binding antibody [BAb]) by Mesoscale discovery (MSD) multiplex assay, as performed by PPD Laboratories, USA, will be assessed for immune response by GMC, GMFR, and seroconversion rate in the following analysis sets and timepoints where applicable:

- Phase 1 IAS: at Day 1 and Day 57
- Phase 1/2 IAS: at Day 1 and Day 57

In addition, the number and proportion (along with two-sided exact Clopper-Pearson 95% CI) of participants demonstrating seropositivity, defined as BAb by MSD multiplex Nucleocapsid (VAC72 N) > 5000 AU/mL) will be summarized by timepoint and treatment arm (as exploratory analysis).

Sensitivity analyses will be performed in the per protocol (PP) immunogenicity sets.

### 6.5.7 Immunogenicity Analysis by subgroups

To assess the consistency of immunogenicity across various subgroups in Phase 1/2/3a, the following immunogenicity analyses will be performed in the subgroups listed in section 6.2.5.

The following analysis will be repeated by subgroup:



Arcturus / Cytel Confidential: This is a controlled document and shall not be changed, saved, copied or reproduced by any unauthorized person.

- Neutralizing antibody (NAb) responses by surrogate virus neutralization test (sVNT) as performed by NIHE (seroconversion rate) in Phase 1/2/3a IAS analysis set: Day 1, Day 29, Day 57, Day 92
- IgG antibody binding the SARS-CoV-2 spike protein (binding antibody [BAb]), as performed by NIHE (seroconversion rate) in Phase 1/2/3a IAS: Day 1, Day 29, Day 57, Day 92

## 6.6 Safety Evaluations

Main estimation of primary safety endpoint for Phase 1/2/3a, 3b, and 3c are described by estimands 3 and 4. Refer to section 9.2.2 for description of those estimands. Pooled data from Phase 1/2/3a/3b will also be presented.

### 6.6.1 Adverse Events

In this study, AEs include subcategories of AEs collected by solicitation of participants as to types of signs and symptoms observed following vaccination (reactogenicity), referred to as "solicited AEs", and a general category of unsolicited AEs that includes any spontaneously reported or observed AE occurring after the signing of informed consent. Further definitions are provided in section 6.6.1.1 and 6.6.1.2.

An overview of main and supportive analyses for safety endpoints is provided in Table 8.

All adverse events that started on or after first vaccination date (Day 1) will be classified as treatment emergent adverse events (TEAEs). For events occurring on the day of first vaccination, an AE will be considered emergent if the investigator indicated on the CRF that the AE occurred after the vaccination. If there is not enough evidence to identify whether an AE occurred before or after the first injection, the AE will be considered as a TEAE. In addition, if an AE onset date or time is missing, or partially missing, and it cannot be determined whether the AE started during a specific reporting interval, it will be considered for the reporting of this interval, unless the end date indicates that the event ended before the interval started.

For participants with more than one episode of the same event, the event of greatest severity and relationship will be used in any summary, while retaining individual records of the episodes in the dataset.

Handling of solicited and unsolicited events of participants who received incorrect study vaccine is described in section 4.1.

**Table 8 Overview of Safety Endpoints**

| | **Description** | **Analysis Set and timepoint** |
|---|---|---|



Arcturus / Cytel Confidential: This is a controlled document and shall not be changed, saved, copied or reproduced by any unauthorized person.

| | | |
|---|---|---|
| Solicited AEs | Occurrence of solicited AEs within 7 days after each study vaccine administration | Phase 1/2/3a RAS: after Day 1 and Day 29 doses<br>Phase 3b RAS: after Day 1 and Day 29 doses<br>Phase 2/3a RAS: After Day 92<br>Phase 3c RAS: after Day 1 and Day 29 doses<br>Pooled RAS: after Day 1 and Day 29 doses |
| | Occurrence of solicited AEs within 30 minutes after each study vaccine administration.<br>Occurrence of solicited AEs by grade | Phase 1/2/3a RAS: after Day 1 and Day 29 doses<br>Phase 3b RAS: after Day 1 and Day 29 doses<br>Phase 2/3a RAS: After Day 92 DosePhase 3c RAS: after Day 1 and Day 29 doses<br>Pooled RAS: after Day 1 and Day 29 doses |
| Unsolicited AEs | Occurrence of unsolicited AEs up to 28 days after each study vaccine administration up to Day 92 (dose 1, dose 2) | Phase 1/2/3a SAS<br>Phase 3b SAS<br>Phase 3c SAS<br>Pooled SAS |
| | Occurrence of unsolicited AEs up to 28 days after each study vaccine administration after switchover/receipt of further study vaccine | Phase 1/2/3a SAS<br>Phase 3b SAS<br>Pooled SAS |
| SAEs, MAAEs, AEs leading to discontinuation of treatment, AEs leading to discontinuation of Study | Occurrence of AEs leading to discontinuation, MAAEs, or SAEs up to Day 92 or Switchover/Further Study Vaccine or receipt of non-study COVID-19 vaccine | Phase 1/2/3a SAS<br>Phase 3b SAS<br>Phase 3c SAS<br>Pooled SAS |



Arcturus / Cytel Confidential: This is a controlled document and shall not be changed, saved, copied or reproduced by any unauthorized person.

| | | |
|---|---|---|
| | Occurrence of AEs leading to discontinuation, MAAEs, or SAEs in other time intervals | Phase 1/2/3a SAS<br>Phase 3b SAS<br>Phase 3c SAS<br>Pooled SAS |

#### 6.6.1.1 Solicited Adverse Events (Reactogenicity)

The term “reactogenicity” refers to selected signs and symptoms (“reactions”) occurring in the hours and days following a vaccination. These signs and symptoms are collected as solicited AEs from participants during the 30 minute observation period after vaccination and by use of the Diary for 7 consecutive days following each study vaccine administrations. The following solicited AEs are included in the Diary:

- **Solicited local AEs:** injection site erythema, injection site pain, injection site induration/swelling, and injection site tenderness
- **Solicited systemic AEs:** arthralgia, chills, diarrhea, dizziness, fatigue, fever (categorized by measured body temperature), headache, myalgia, and nausea, vomiting

Solicited AEs are captured in the following way:

- Participant’s data recorded directly on the Diary (paper or eDiary, primary analysis)
- Investigator’s re-assessed data

Analysis of reactogenicity data will be performed based on the participant’s data recorded on the Diary/eDiary.

Reactogenicity will be analyzed after each vaccine dose (within 30 minutes or during first 7 days after the dose - refer to Table 8 for timepoints available). Participants will be considered for the computation of percentages in the specified time interval if they received the study vaccine and provided at least 1 reactogenicity diary report in the specified interval. Participants whose data are completely missing within the summary of event and timepoint (i.e no answer to a reaction category for the specific timepoint) will not be included in both numerator and denominator. For each time interval and reaction category the number of participants without missing data will be presented.

Severity of solicited local and systemic reactions will be graded as follows: grade 0 (none), grade 1 (mild), grade 2 (moderate), grade 3 (severe), and grade 4 (potentially life threatening). For participants with more than one episode of the same event in a reporting period, the event of greatest severity will be used in any summary.

Presence of a local/systemic reaction within the 30 minute observation period following each vaccination is defined as the reaction recorded in the CRF with a severity grade of 1 or higher on the analysis time point. Presence of any local/systemic reaction within 30 minute observation period following each vaccination is defined as participant reports at least one local or systemic reaction within 30 minute observation period.



Arcturus / Cytel Confidential: This is a controlled document and shall not be changed, saved, copied or reproduced by any unauthorized person.

Presence of each local/systemic reaction within 7 days after each dose of study vaccine is defined as participant reports the reaction as "yes" on the analysis time point in the diary. Presence of any local/systemic reaction within 7 days after each dose is defined as participant reports the reaction as "yes" in the diary for at least one local or systemic reaction.

The number and proportion of participants experiencing solicited events will be presented as follows:

- Overview of Reactions within 30 minutes after each dose:
    - at least one reaction (local or systemic), at least one local reaction, at least one systemic reaction
    - At least one reaction, by reaction
- Summary of reactions within 30 minutes after each dose by grade:
    - one reaction (local or systemic), at least one local reaction, at least one systemic reaction
    - At least one reaction, by reaction
- Overview of Reactions within 7 days after each dose:
    - at least one reaction (local or systemic), at least one local reaction, at least one systemic reaction
    - At least one reaction, by reaction
- Summary of reactions within 7 days after each dose by grade:
    - one reaction (local or systemic), at least one local reaction, at least one systemic reaction
    - At least one reaction, by reaction
- Summary of first onset of reaction within 7 days after each dose by day of occurrence:
    - at least one reaction (local or systemic), at least one local reaction, at least one systemic reaction
    - At least one reaction, by reaction
- Summary of any reactions within 7 days after each dose by day of occurrence:
    - at least one reaction (local or systemic), at least one local reaction, at least one systemic reaction
    - At least one reaction, by reaction

Each of the analysis above will be displayed in the following analysis set and timepoint:

| Analysis set | Timepoint |
|---|---|
| Phase 1/2/3a RAS | After Dose 1, after Dose 2, after Dose 1 or 2 |
| Phase 2/3a RAS | After Dose 3 |



*Arcturus / Cytel Confidential: This is a controlled document and shall not be changed, saved, copied or reproduced by any unauthorized person.*

| | |
|---|---|
| | Any dose (Dose 1, 2 and 3) |
| Phase 3b RAS | After Dose 1, after Dose 2, after Dose 1 or 2 |
| Phase 3c RAS | After Dose 1, after Dose 2, after Dose 1 or 2 |
| Pooled RAS | After Dose 1, after Dose 2, after Dose 1 or 2 |

Overview tables for Phase 3b RAS will be also produced by subgroups as specified in Section 6.2.5.

For outputs by grade, bar plots will be produced displaying percentage of participants with each grade.

Median time of onset and median duration of local and systemic reactions will be summarized for Phase 1/2/3a RAS, Phase 3b RAS, Phase 3c RAS, and Pooled RAS after Dose 1 or 2.

All solicited AEs occurring on study will be listed in participant data listings using the randomized set. In addition, solicited AEs occurring in the set of participants who received at least one incorrect dose of study vaccines will be provided.

#### 6.6.1.2 Unsolicited Adverse Events

Unsolicited AEs are defined as any spontaneously reported or discovered AE. Unsolicited AEs can also be solicited AE that leads to: A medically attended visit, an SAE, a discontinuation of study vaccine, a withdrawal from the study, or any solicited AE that persists beyond 7 days after the first study vaccine administration.

Unsolicited AEs will be coded using the MedDRA and displayed in tables using System/Organ/Class (SOC) and Preferred Term (PT). Treatment emergent AEs will be presented in tables, and all AEs will be included in listings where applicable.

In any tabulation, unsolicited AEs will be summarized by participant, therefore, a participant contributes only once to the count for a given Unsolicited AE (SOC or PT) in the tabulation. All episodes of all events will be provided in listings.

Unsolicited AEs with onset during the first 28 days after vaccination will be analyzed in the following analysis sets as defined below:

| Analysis set | Timepoint |
|---|---|
| Phase 1/2/3a SAS | • After Dose 1, after Dose 2, after Dose 1 or 2<br>• After Dose 3, after Dose 4<br>• After Any dose |
| Phase 3b SAS | • After Dose 1, after Dose 2, after Dose 1 or 2<br>• After Dose 3, after Dose 4<br>• After Any dose |
| Phase 3c SAS | • After Dose 1, after Dose 2, after Dose 1 or 2 |



Arcturus / Cytel Confidential: This is a controlled document and shall not be changed, saved, copied or reproduced by any unauthorized person.

| | |
|---|---|
| | • After Any dose |
| Pooled SAS | • After Dose 1, after Dose 2, after Dose 1 or 2<br>• After Dose 3, after Dose 4<br>• After Any dose |

Participants will be considered for the computation of percentages in the specified time interval if they received the study vaccine in the specified interval. For each time interval the number of participants without missing dose will be presented.

Only data up to receipt of non-study COVID-19 vaccine (IcEv2) will be included in summary tables. Participant's data after the receipt of non-study COVID-19 vaccine will be summarized separately in a similar manner as unsolicited AEs for the following time interval: from the receipt of non-study COVID-19 vaccine up to the Final Visit/ET.

The following summaries will be presented for unsolicited AEs:

- Overview of number and percentage of participants with at least one:
  - AE
  - Related AE
  - AE by severity
  - MAAE
  - Related MAAE
  - SAE
  - Related SAE
  - AE leading to premature discontinuation of vaccine series
  - AE leading to withdrawal from study
  - AE with death as an outcome
  - AE with covid-related death as an outcome
- Number and percentage of participants with an AE by SOC and PT
- Number and percentage of participants with an AE by PT sorted by descending frequency in the ARCT-154 group
- Number and percentage of participants with an AE related to study vaccination by SOC and PT
- Number and percentage of participants with an AE by SOC, PT and severity
- Number and percentage of participants with an AE related to study vaccination by SOC, PT and severity

Overview tables and outputs by SOC and PT after each will be also produced by subgroups as specified in Section 6.2.5 based Phase 3b SAS.



Arcturus / Cytel Confidential: This is a controlled document and shall not be changed, saved, copied or reproduced by any unauthorized person.

Non-treatment emergent AEs will be listed.

#### 6.6.1.3 Serious Adverse Events and other Events

SAEs, MAAEs, AEs leading to withdrawal of vaccine and AEs leading to study discontinuation will be summarized in the same manner as unsolicited AEs:

- Overview table
- Events by SAE criteria (only applicable for SAEs)
- Summary by SOC and PT
- Summary by PT
- Summary by SOC, PT and Severity
- Summary of related events by SOC and PT
- Summary of related events by SOC, PT and severity

SAEs with fatal outcome will be summarized by SOC and PT.

SAEs, MAAEs, AE leading to discontinuation and AEs with fatal outcome will be summarized for the following analysis sets and time intervals:

| Analysis set | Time interval |
|---|---|
| Phase 1/2/3a SAS<br>Phase 3b SAS | • From Day 1 to Day 92 (for participants without Switchover/Further study vaccine) or Switchover/Further study vaccine . Percentages will be computed based on the number of participants in SAS who received at least one dose of study vaccine during reporting period (i.e a dose at Day 1 or at Day 29).<br>• From Switchover/Further study vaccine to End of study, for participants with Switchover/Further study vaccine. Percentages will be computed based on the number of participants in SAS who received at least one dose of study vaccine during reporting period (i.e a dose at Day 92 or at Day 120).<br>• From Day 92 to End of Study, for participants without Switchover/Further study vaccine. Percentages will be computed based on the number of participants in SAS still on study at Day 92 and without Switchover or further study vaccine.<br>• From the first dose of ARCT-154 vaccine administration to End of Study. Percentages will be computed based on the number of participants in SAS who received at least one dose of ACRT154 study vaccine. |
| Phase 3c SAS | • From Day 1 to Day 92. Percentages will be computed based on the number of participants included in Phase 3c SAS. |



Arcturus / Cytel Confidential: This is a controlled document and shall not be changed, saved, copied or reproduced by any unauthorized person.

| | |
|---|---|
| | • From Day 92 to End of Study. Percentages will be computed based on the number of participants included in Phase 3c SAS. |
| Pooled SAS | From Day 1 to Day 92 (for participants without Switchover/Further study vaccine) or Switchover/Further study vaccine (main estimation). Percentages will be computed based on the number of participants in Pooled SAS who received at least one dose of study vaccine during reporting period (i.e Day 1 or Day 29). |

Overview table and output by SOC and PT for SAEs from the first dose of ARCT-154 vaccine administration to End of Study for Phase 3b SAS will be also produced by subgroups as specified in Section 6.2.5.

The following participant data listings will be produced using the randomized set:

- SAEs
- MAAEs
- AEs leading to premature discontinuation of vaccine series
- AEs leading to withdrawal from the study
- AEs with Fatal Outcome

A separate set of listings will be produced for these AEs occurring in the set of participants who received at least one incorrect dose of study vaccines.

### 6.6.2 Laboratory Data

Hematology and chemistry laboratory data were collected only for participants in Phase 1. For that reason, analysis of hematology and chemistry laboratory data will be done on Phase 1 SAS only.

The following parameters will be analyzed:

- Hematology: Hemoglobin, White blood cells, Platelets, Neutrophils, Lymphocytes
- Chemistry: Creatinine, Alanine transaminase, Aspartate transaminase, Total bilirubin, Gamma-glutamyl transferase, Alkaline phosphatase

Data will be analyzed according to the visit labels as collected. Assessments performed at unscheduled visits will be not included in summary tables but will be included in listings.

The actual value and change from Baseline to each visit where assessment was scheduled will be summarized for each hematology and chemistry parameter. Additionally, number and percentage of participants with abnormality (high/low) and with clinically significant result will be summarized at each visit.

Positive pregnancy test results will be provided in data listings.

A listing presenting the laboratory data of subjects with at least one abnormal laboratory values will be provided.



Arcturus / Cytel Confidential: This is a controlled document and shall not be changed, saved, copied or reproduced by any unauthorized person.

### 6.6.3 Vital Signs and Physical Examinations

Vital Signs will be analyzed for the following analysis sets:

- Phase 1/2/3a SAS
- Phase 3b SAS
- Phase 3c SAS
- Pooled SAS

Vital signs measured include systolic and diastolic blood pressure, heart rate, and respiratory rate.

Data will be analyzed according to the visit labels as collected. Assessments performed at unscheduled visits will be not included in summary tables but will be included in listings.

The actual value and change from Baseline to each visit (pre-dose assessment, if dosing visit) where assessment was scheduled (including ET visit) will be summarized for systolic and diastolic blood pressure. Summary of post-dose measurements and changes from pre-dose measurement will be done for each time point where pre- and post-dose measurements were performed.

Systolic and diastolic blood pressure will be graded as shown in Table 9. Shift tables from Baseline to each visit and from pre-dose to post-dose will be done.

**Table 9 Vital Signs Toxicity Grading Scales**

| | Systolic Blood Pressure (mmHg) | Diastolic Blood Pressure (mmHg) |
|---|---|---|
| Normal | ≤ 140 | ≤ 90 |
| Grade 1 | 141-150 | 91-95 |
| Grade 2 | 151-155 | 96-100 |
| Grade 3 | > 155 | > 100 |



Arcturus / Cytel Confidential: This is a controlled document and shall not be changed, saved, copied or reproduced by any unauthorized person.

# 7 CHANGES TO PLANNED ANALYSES

## 7.1 Summary of changes from protocol in this SAP (changes not introduced SAP Part 1 or SAP Part 2)

Changes and clarifications to planned analyses described in the protocol and not already introduced within SAP Part 1 or SAP Part 2 are described in the below table.

| **Protocol Section** | **Analysis method described originally in the protocol** | **Planned Analysis** | **Reasons for changes** |
|---|---|---|---|
| Section 3.1.1 Phase 1/2/3a Substudy Primary Objectives and Endpoints | Safety data from participants in Phase 2/3a will be pooled for all time points and will be displayed separately to safety data from Phase 1. | Safety analyses will mostly be performed on the Phase 1/2/3a populations and we will not be repeated separately for Ph1 and Ph2/3a as described in Table 8 of this SAP | Safety data are comprehensive, with same inclusion and exclusion criteria across Phase 1/2/3a, so not relevant to display safety from a smaller phase 1 set separately |
| Section 3.1.2 Phase 1/2/3a Substudy Secondary Objectives and Endpoints | Immunogenicity will be at the following time points.<br>- Phase 1 IAS: Day 1 (baseline), Day 29, Day 57, Day 92, Day 394.<br>- Phase 2/3a IAS: Day 1 (baseline), Day 29, Day 57, Day 92, Day 120, Day 394<br>- Phase 1/2/3a IAS: Day 1 (baseline), Day 29, Day 57, Day 92 | Samples were collected at Day 394 but were not analyzed so no Day 394 results will be presented for immunogenicity | Immunogenicity would be affected by recommended routine covid-19 vaccinations as such the immunogenicity results will not represent the persistence after the primary vaccination |
| | | Immunogenicity analyses will mostly be performed on the Phase 1/2/3a populations and we will not be repeated separately for Ph1 and Ph2/3a unless the data is only available for Ph1, Ph1/2 or Ph2/3a as described in Table 7 of this SAP | Immunogenicity data are comprehensive, with same inclusion and exclusion criteria across Phase 1/2/3a, so not relevant to display immunogenicity separately from small phase 1 dataset |
| Section 3.1.3 Phase 1/2/3a Substudy Exploratory Objectives and Endpoints | To compare the humoral immune responses to ARCT-154 with those following COVID-19 | This objective will not be assessed | No convalescent sera results available |



*Arcturus / Cytel Confidential: This is a controlled document and shall not be changed, saved, copied or reproduced by any unauthorized person.*

| | | | |
|---|---|---|---|
| Section 3.1.3 Phase 1/2/3a Substudy Exploratory Objectives and Endpoints | Blood samples from post-vaccination timepoints evaluated for immune responses to SARS-CoV-2 VOC/VOI using the following assays PRNT50, sVNT, MNT | PRNT 50 is removed from the exploratory endpoint description | It is already covered in secondary objective 2. |
| Section 3.3.1 Phase 3c Primary objective | To evaluate noninferiority of neutralizing antibody geometric mean concentration for ARCT-154 versus ChAdOx1 at Day 57 | No immunogenicity analysis will be performed for Phase 3c. | Phase 3c samples were not analyzed as this immunogenicity objective is considered not relevant. Clinical efficacy was demonstrated in the phase 3b of this study as such demonstration of immunological comparability against authorized comparator (ChAdOx1) is not required. |
| Section 3.3.2 Phase 3c Secondary Objectives | Secondary Objectives 1-6 to analyze immunogenicity | No immunogenicity analysis will be performed for Phase 3c. | |
| Section 3.3.3 Phase 3c Exploratory Objectives | Exploratory Objectives 5-6 to analyze immunogenicity | No immunogenicity analysis will be performed for Phase 3c. | |
| Section 3.3.3 Phase 3c Exploratory Objectives | Exploratory Objectives 1-4 to analyze efficacy | No efficacy analysis will be performed for Phase 3c. | Covid cases were not adjudicated for this exploratory objective. The clinical exploratory objective is considered not relevant. |
| Section 8.2 Analysis set<br><br>Immunogenicity Analysis Set (IAS) | Phase 1/2/3a IAS this pools data from Phase 1, 2 and 3a participants and will include evaluations of immunogenicity up to Day 92 only. | Phase 1/2/3a IAS (or phase 1/2 IAS): this pools data from Phase 1, 2 and 3a participants and will include evaluations of immunogenicity up to Day 92 only. Of note, this analysis set may be restricted to Phase 1 and Phase 2 participants for analysis on parameter not collected for phase 3a participants.<br><br>Phase 1/2/3a PP immunogenicity set (or Phase 1/2 PP | This was done due to some immunogenicity data being collected only for Phase 1 and 2 participants and therefore Phase 1/2/3a PP set is not applicable for such analyses. |



Arcturus / Cytel Confidential: This is a controlled document and shall not be changed, saved, copied or reproduced by any unauthorized person.

| | | | |
|---|---|---|---|
| | Phase 1/2/3a PP Immunogenicity set: this pools data from Phase 1, 2 and 3a participants and will include evaluations of immunogenicity up to Day 92 only. | immunogenicity set): this pools data from Phase 1, 2 and Phase 3a participants and excludes any participant that has evidence of SARS-CoV-2 infection at baseline or prior to the analysis time point concerned (IcEv3) or that receives a non-study COVID-19 vaccine prior to the analysis time point concerned (IcEv2). This set will include evaluations of immunogenicity up to Day 92 only. Of note, this analysis set will be restricted to Phase 1/2 participants for analysis on parameter not collected for phase 3a participants and will be referred as Phase 1/2 PP immunogenicity set) | |
| Section 8.2 Analysis Set Immunogenicity (IAS) Analysis set | Includes all participants who received all protocol-required doses of study vaccine (ARCT-154 or placebo or ChAdOx1) up to the evaluation timepoint concerned, who have no evidence of prior SARS-CoV-2 infection at Day 1 (IcEv3; Section 8.3.1) and who have at least 1 valid post-vaccination immunogenicity assay result. | Includes all participants who received all protocol-required doses of study vaccine (ARCT-154 or placebo) up to the evaluation timepoint concerned, who have no evidence of prior SARS-CoV-2 infection (no positive RT-PCR or other COVID-19 test or seropositivity for anti-nucleocapsid antibody) up to Day 57 and who have at least 1 valid post-vaccination immunogenicity assay result. | The definition was updated to clarify that participants with early COVID-19 infection (positive RT-PCR or other COVID-19 test or seropositivity for anti-nucleocapsid antibody) up to Day 57 are excluded from these analysis sets as also described in Tables 14 and 15 of the protocol. |
| Section 8.2 Analysis set | All participants in the IAS will be analyzed according to which study vaccine was received. | All participants in the IAS will be analyzed according to which study vaccine was assigned. | Align with ICH E9.<br>As per definition of IAS, participants included are those who received the per protocol required dose. Thus in case of misdosing, the participants will be excluded from the IAS. |
| Section 8.3.1 Table 14 Intercurrent Event types | IcEv3 (Early infection): Positive RT-PCR test or seropositivity for | IcEv3 (Early infection): Positive RT-PCR test or | Addition of other COVID-19 positive test for complete |



Arcturus / Cytel Confidential: This is a controlled document and shall not be changed, saved, copied or reproduced by any unauthorized person.

| | | | |
|---|---|---|---|
| | antinucleocapsid antibody or neutralizing antibody (as assessed by sVNT for Phase 1/2/3a and MNT for Phase 3c) indicating exposure to SARS-CoV-2 prior to vaccination or SARS-CoV-2 infection prior to 7 days (inclusive) after the second study vaccination dose (Day 35). | other COVID-19 positive test or seropositivity for anti-nucleocapsid antibody indicating exposure to SARS-CoV-2 prior to vaccination or SARS-CoV-2 infection prior to 7 days (inclusive) after the second study vaccination dose (Day 35).<br><br>Note: This also impacts mITT and IAS analysis sets definitions where IcEv3 is considered. | assessment of early infection |
| Section 8.3.1 Table 15 (estimand 1 – Rationale for Strategies) | IcEv4 and IcEv5 could also be potentially confounding but it is more difficult to determine the precise timing of these events, so a principal stratum strategy is used to exclude these participants from immunogenicity analyses. | IcEv4 and IcEv3 could also be potentially confounding but it is more difficult to determine the precise timing of these events, so a principal stratum strategy is used to exclude these participants from immunogenicity analyses. | Correction |
| Section 8.3.1 Table 17 (estimand 3 – unsolicited Aes - Intercurrent Event (IcEv) Strategy) | Intercurrent Event (IcEv) Strategy:<br><br>IcEv2 (Non-study vaccine): treatment policy strategy<br><br>IcEv5 (Confounding On-study Medications): composite strategy | IcEv2 (Non-study vaccine): while on treatment strategy<br><br>IcEv5 (Confounding On-study Medications): treatment policy strategy | icEv2: Correction of the name of the strategy to reflect the fact that events occurring after IcEv2 will not be included in the analysis as specified in protocol<br><br>icEv5: Correction of the name of the strategy to reflect the fact that events occurring after IcEv5 are included in the analysis as specified in the protocol |
| Section 8.3.1 Table 17 (estimand 4 – Reactogenicity - Rationale for Strategies) | Events occurring after IcEv2, IcEv3 and IcEv5 could contribute spurious reactogenicity data so a treatment policy is used for these events and events occurring after | All solicited events will be analyzed regardless of occurrence of intercurrent events. | Reactogenicity is participant reported outcome, which will be analyzed as it is without modification. |



Arcturus / Cytel Confidential: This is a controlled document and shall not be changed, saved, copied or reproduced by any unauthorized person.

| | | | |
|---|---|---|---|
| | these IcEv's will be summarized separately | | |
| Section 8.7.2 Table 18 estimand 2 (efficacy) | Imputation/Data/Censoring Rules: For deaths unrelated to COVID-19, censor at date of death. For participants who undergo Switchover/Further Study Vaccine or receive a non-study COVID-19 vaccine, censor at date of Switchover or non-study vaccination. | Participants last date at risk date will also be censored for Lost to Follow-up | Add rule of handing censoring condition for lost to follow-up scenario. |
| Section 8.7.2 Table 18 estimand 2 (efficacy) | Sensitivity: the same analysis will be performed in the Pooled mITT and Phase 3b PP analysis sets. | 2 additional sensitivity analyses to be performed.<br>• A sensitivity analysis of all Covid cases reported in CRF and not confirmed by the blinded Independent Adjudication Committee or with severity as reported in the CRF has been added for the primary endpoint, severe cases and deaths due to COVID-19.<br>• Day 36 is defined as second dose+7 days (29+7=36 days), and Day 92 is defined as second dose+63 days (29+63=92 days). Another sensitivity analysis of the primary endpoint (Phase 3b only) will be performed using Day 92 visit instead of second dose+63 days. | Add additional sensitivity analyses to assess the robustness of the primary analysis results. |
| Section 8.7.2 Table 18 estimand 3 (safety) | Supportive: Analysis will be performed for different time intervals, specifically: | As per section 6.6.1.3 of this SAP, only the following intervals will be done: | To summarize study periods that are not overlapping |



Arcturus / Cytel Confidential: This is a controlled document and shall not be changed, saved, copied or reproduced by any unauthorized person.

| | | | |
|---|---|---|---|
| | All MAAEs, AE/SAEs, AEs leading to discontinuation/ withdrawal up to Day 29, Day 57, Day 92, and Day 394 or Final Visit/Early Termination Visit/receipt of non-study COVID-19 vaccine after the first vaccination will be summarized accordingly.<br><br>AEs occurring after the Day 92 will be recorded and summarized for the interval Day 92 up to Day 394 (Phase 1/2/3a and Phase 3b only). For Phase 1/2/3a and 3b, AEs in participants that do not receive additional study vaccine at Day 92 will be recorded separately for the interval from Day 92 up to Day 394. | • From Day 1 to Day 92 (for participants without Switchover/Further study vaccine) or Switchover/Further study vaccine<br>• From Switchover/Further study vaccine to End of Study, for participants with Switchover/Further study vaccine<br>• From Day 92 to End of Study, for participants without Switchover/Further study vaccine<br>• From the first dose of ARCT-154 vaccine administration to End of Study | |
| Section 8.7.3.1 Main Estimation of Primary Immunogenicity Endpoint for Phase 1/2/3a Substudy (Estimand 1) | For SCR, chi-square or Fisher's exact test will be used to perform comparison of the two treatment arms. 95% CI of the SCR will also be presented. Pearson's Chi-square test will be used except for the case of small cell count (less than 5). | The number and proportion of participants achieving seroconversion at Day 57 will be provided for each treatment. In addition, the treatment difference in proportion of participants achieving seroconversion at Day 57 will be provided, along with corresponding 95% CI. | No p-value will be computed as the main interest for this analysis is change from baseline. |
| Section 8.6 Description of Additional Subgroups to Be Analyzed | For immunogenicity subgroup analyses, the following subgroups were listed as potential subgroups to be assessed if feasible.<br>• Seropositive or seronegative by SARS-CoV-2 nucleocapsid antibody test on or prior to Day 29<br>• Receipt of a live non-COVID-19 vaccine within 28 days before or after any study vaccination | These Subgroups will not be defined, and subgroup analyses will not be performed | These analyses will not be performed due to low frequency in one of the two subgroups. |
| Section 8.4.1 Phase 1/2/3a Substudy | For the seroconversion rate primary immunogenicity objective, the null hypothesis | Following regulatory interactions, a more | More conservative approach added for testing |



Arcturus / Cytel Confidential: This is a controlled document and shall not be changed, saved, copied or reproduced by any unauthorized person.

| | | | |
|---|---|---|---|
| Statistical Hypothesis | ($H0^{Immunogenicity}$) is that the lower bound of the 95% CI for ARCT-154 seroconversion rate ($SC^{154}$) is ≤ seroconversion rate for placebo ($SC^{Placebo}$); i.e., $H0^{Immunogenicity}$: lower bound 95% CI for $SC^{154} \leq SC^{Placebo}$ | conservative methodology has been added:<br>• $H0^{Immunogenicity}$: lower bound of the 95% CI of ($SC^{154}$ - $SC^{Placebo}$) ≤ 0% , versus<br>• $H1^{Immunogenicity}$: lower bound of the 95% CI of ($SC^{154}$ - $SC^{Placebo}$) > 0% | |



Arcturus / Cytel Confidential: This is a controlled document and shall not be changed, saved, copied or reproduced by any unauthorized person.

## 7.2 Summary of changes from protocol documented in SAP Part 1 and SAP Part 2 applicable to this SAP

SAP Part 1 Section 13 and SAP Part 2 Section 12 describe changes or clarifications from the protocol in planned analyses. The table below describes changes or clarifications from the protocol described in SAP Part 1 and/or SAP Part 2 applicable also for this SAP and any additional clarifications added in this SAP for these aspects.

| Protocol Section | Analysis method described originally in the protocol | Planned Analysis | Reasons of changes |
|---|---|---|---|
| Section 3.2.2 Phase 3b Secondary Objectives and Endpoints | Secondary endpoint #2: 'Efficacy will be evaluated in all participants in the Phase 3b mITT who have received any dose of study vaccine in the first vaccination series, with no evidence of infection prior to vaccination.' | Efficacy will be evaluated in all participants in the Phase 3b ITT who have received any dose of study vaccine in the first vaccination series, with no evidence of infection prior to vaccination.'<br><br>This change also applies for the endpoint analysis for Phase 1/2/3a and Pooled as described in Table 6 of this SAP. | Analysis population corrected to ITT since the mITT states that participants must have received both vaccinations in the initial series so is not compatible with this analysis |
| Section 3.2.2 Phase 3b Secondary Objectives and Endpoints | Secondary endpoint #4: 'Efficacy will be evaluated in all participants in the Phase 3b mITT' | Efficacy will be evaluated in all participants in the Phase 3b ITT that have received both vaccinations in the first vaccination series and who had no evidence of onset of SARS-CoV-2 infection (i.e. positive RT-PCR test) between Day 1 and Day 35 inclusive.<br><br>This change also applies for the endpoint analysis for Phase 1/2/3a and Pooled as described in Table 6 of this SAP.<br><br>In the current SAP, the analysis population is now described as ""who received both protocol-required doses of study vaccine with no SARS-CoV-2 infection between Day 1 and Day 35, but including those who are seropositive at baseline (Day 1)" as further clarification. | Analysis population corrected to mITT since the mITT states that participants must have received both vaccinations in the initial series. Additionally clarified that for this endpoint participants must have no evidence of onset of SARS-CoV-2 infection between Day 1 and Day 35 inclusive |



Arcturus / Cytel Confidential: This is a controlled document and shall not be changed, saved, copied or reproduced by any unauthorized person.

| Protocol Section | Analysis method described originally in the protocol | Planned Analysis | Reasons of changes |
|---|---|---|---|
| Section 3.2.3 Phase 3b Exploratory Objectives and Endpoints | Exploratory endpoint #2: 'Efficacy may be evaluated in all participants in the Phase 3b mITT'<br><br>This also applies to exploratory endpoint #1. | Efficacy may be evaluated in all participants in the Phase 3b ITT that have received any dose of study vaccine in the first vaccination series, with no evidence of SARS-CoV-2 infection (i.e. positive RT-PCR test) prior to vaccination.<br><br>This change also applies for the endpoint analysis for Phase 1/2/3a and Pooled as described in Table 6 of this SAP.<br><br>In the current SAP, further clarification is added for infection as "infection (i.e. positive RT-PCR test or seropositivity for anti-nucleocapsid antibody)". | Analysis population corrected to ITT since the mITT states that participants must have received both vaccinations in the initial series so is not compatible with this analysis. Additionally clarified that for this endpoint evidence of SARS-CoV-2 infection is a positive RT-PCR test |
| Section 3.2.3 Phase 3b Exploratory Objectives and Endpoints | Exploratory endpoint #3: 'Efficacy may be evaluated in all participants in the Phase 3b mITT that have received any dose of study vaccine in the first vaccination series, regardless of baseline status for evidence of prior SARS-CoV-2 infection. | Efficacy may be evaluated in all participants in the Phase 3b ITT that have received any dose of study vaccine in the first vaccination series, regardless of baseline status for evidence of prior SARS-CoV-2 infection.<br><br>This change also applies for the endpoint analysis for Phase 1/2/3a and Pooled as described in Table 6 of this SAP. | Analysis population corrected to ITT since the mITT states that participants must have received both vaccinations in the initial series so is not compatible with this analysis. |
| Section 3.2.3 Phase 3b Exploratory Objectives and Endpoints | Exploratory endpoint #4: 'Efficacy will be evaluated in participants in Phase 3b mITT. The endpoint to be evaluated is as follows:<br><br>The first occurrence of confirmed, protocol-defined COVID-19 for all participants infected with the same strain of SARS-CoV-2, with onset between Day 36 and Day 92 inclusive' | 4a) Efficacy will be evaluated in participants in Phase 3b mITT. The endpoint to be evaluated is as follows:<br><br>The first occurrence of confirmed, protocol-defined COVID-19 for all participants infected with the same variant of SARS-CoV-2, with onset between Day 36 and Day 92 inclusive | An additional analysis added for this objective for completeness of evaluations |



Arcturus / Cytel Confidential: This is a controlled document and shall not be changed, saved, copied or reproduced by any unauthorized person.

| Protocol Section | Analysis method described originally in the protocol | Planned Analysis | Reasons of changes |
|---|---|---|---|
| | | 4b) Efficacy will also be evaluated in participants in the Phase 3b ITT that have received any dose of study vaccine in the first vaccination series, with no evidence of infection prior to vaccination. This endpoint to be evaluated is as follows:<br><br>The first occurrence of confirmed, protocol-defined COVID-19 for all participants infected with the same variant of SARS-CoV-2, with onset between at any time after the first study vaccination and up to Day 92<br><br>This change also applies for the endpoint analysis for Pooled as described in Table 6 of this SAP. | |
| Section 3.2.3 Phase 3b Exploratory Objectives and Endpoints | Exploratory endpoint #5 'Efficacy will be evaluated in participants in Phase 3b mITT.<br><br>The endpoint to be evaluated is as follows:<br><br>• The first occurrence of confirmed, protocol-defined severe COVID-19 for all participants infected with the same strain of SARS-CoV-2, with onset at any time after the first study vaccination and up to Day 92.' | 5a) Efficacy will be evaluated in participants in Phase 3b ITT that have received any dose of study vaccine in the first vaccination series, with no evidence of infection prior to vaccination.<br><br>The endpoint to be evaluated is as follows:<br><br>• The first occurrence of confirmed, protocol-defined severe COVID-19 for all participants infected with the same variant of SARS-CoV-2, with onset at any time after the first study vaccination and up to Day 92.<br><br>5b) Efficacy will also be evaluated in participants in Phase 3b mITT. The endpoint to be evaluated is as follows: | An additional analysis added for this objective for completeness of evaluations. Also, the analysis population for the original endpoint (5a) corrected to ITT since the mITT states that participants must have received both vaccinations in the initial series so is not compatible with this analysis. |



Arcturus / Cytel Confidential: This is a controlled document and shall not be changed, saved, copied or reproduced by any unauthorized person.

| Protocol Section | Analysis method described originally in the protocol | Planned Analysis | Reasons of changes |
|---|---|---|---|
| | | • The first occurrence of confirmed, protocol-defined severe COVID-19 for all participants infected with the same variant of SARS-CoV-2, with onset between Day 36 and Day 92 inclusive<br><br>This change also applies for the endpoint analysis for Pooled as described in Table 6 of this SAP. | |
| Section 7.4.1.1.1.1 Solicited Adverse Events | Also solicited from participants but not categorized as an AE is the use of antipyretics and analgesics to prevent or treat solicited AEs. | This information was not solicited in the diary card | Information not collected in the diary card |
| Section 8.2 Modified Intent-to-treat (mITT) Analysis Set | Includes all participants who received all protocol-required doses of study vaccine (ARCT 154 or placebo) up to the evaluation timepoint concerned, and who have no evidence of SARS-CoV-2 infection on Day 1 up to 7 days after the 2nd study vaccination. | Includes all participants who received all protocol-required doses of study vaccine (ARCT 154 or placebo) up to the evaluation timepoint concerned, and who have no evidence of SARS-CoV-2 infection **(refer to IcEv 3, Section 6.3.10)** on Day 1 up to 7 days after the 2nd study vaccination.<br><br>In the current SAP, further clarification is added for infection instead of the reference i.e. "infection (i.e. no positive RT-PCR or other COVID-19 test or seropositivity for anti-nucleocapsid antibody)". | Added reference to definition of early infection |
| Section 8.2 Per-protocol Analysis Set | Includes all eligible randomized participants who received all protocol-required doses of study vaccine (ARCT-154 or placebo) up to the evaluation timepoint concerned and within the protocol predefined window, and who have no major protocol deviations expected | Includes all eligible randomized participants who received all protocol-required doses of study vaccine (ARCT-154 or placebo) up to the evaluation timepoint concerned and within the protocol predefined window, and who have no major protocol deviations expected | Additional clarifications added. 'Received' corrected to 'assigned'. |



Arcturus / Cytel Confidential: This is a controlled document and shall not be changed, saved, copied or reproduced by any unauthorized person.

| Protocol Section | Analysis method described originally in the protocol | Planned Analysis | Reasons of changes |
|---|---|---|---|
| | to affect efficacy, safety or reactogenicity assessments as determined by the Sponsor Medical Monitor or designee in a blinded manner. The PP analysis set will be analyzed according to which study vaccine was received, in the event there is a discrepancy. | to affect efficacy **(Efficacy PP set)**, safety **(Safety PP set)** or reactogenicity **(Reactogenicity PP set)** assessments as determined by the Sponsor Medical Monitor or designee in a blinded manner. The PP analysis set will be analyzed according to which study vaccine was **assigned**, in the event there is a discrepancy.<br><br>In the current SAP, wording for Safety PS et and Reactogenicity PP set removed since these analyses sets are not used. Further clarification is added to define PP efficacy and PP immunogenicity. | |
| Section 8.2 Per-protocol (PP) analysis set | The PP analysis set will be analyzed according to which study vaccine was received, in the event there is a discrepancy. | The PP analysis set will be analyzed according to which study vaccine was assigned, in the event there is a discrepancy. | Corrected received to assigned |
| Section 8.3.1 Table 14 Intercurrent Events | IcEv3 (Early infection): Positive RT-PCR test or seropositivity for antinucleocapsid antibody or neutralizing antibody (as assessed by sVNT for Phase 1/2/3a and MNT for Phase 3c) indicating exposure to SARS-CoV-2 prior to vaccination or SARS-CoV-2 infection prior to 7 days (inclusive) after the second study vaccination dose (Day 35).<br><br>Note: for immunogenicity endpoints, Day 57 is the watershed for early infection. | Positive RT-PCR test or seropositivity for antinucleocapsid antibody indicating exposure to SARS-CoV-2 prior to vaccination or SARS-CoV-2 infection prior to 7 days (inclusive) after the second study vaccination dose (Day 35).<br><br>Further clarification in current SAP:<br><br>Positive RT-PCR test or other COVID-19 positive test or seropositivity for anti-nucleocapsid antibody indicating exposure to SARS-CoV-2 prior to vaccination or SARS-CoV-2 infection prior to 7 days (inclusive) after the second study vaccination dose (Day 35). | Text of protocol did not specifically define this IcEv for analyses to be conducted in the Phase 3b and Pooled population so this has been added. Text referring to Phase 1/2/3a and 3c removed as not relevant to this SAP |



*Arcturus / Cytel Confidential: This is a controlled document and shall not be changed, saved, copied or reproduced by any unauthorized person.*

| Protocol Section | Analysis method described originally in the protocol | Planned Analysis | Reasons of changes |
|---|---|---|---|
| Section 8.3.1 Table 15 Estimands for Phase 1/2/3a Primary, Secondary and Exploratory Immunogenicity endpoints | IcEv1 (Death): hypothetical strategy | IcEv1 (Death): Treatment policy strategy | No imputation of missing data is performed so treatment policy is the appropriate strategy |
| Section 8.3.1 Table 16 Estimands for Primary, Secondary and Exploratory Efficacy Analyses | IcEv1 (Death not due to COVID-19): Hypothetical strategy<br><br>IcEv2 (Non-study COVID-19 vaccine):<br><br>IcEv3 (Early infection): Principal stratum strategy<br><br>IcEv4 (Study infection): Composite strategy<br><br>IcEv5 (Confounding On-study Medications): Hypothetical strategy | IcEv1 (Death not due to COVID-19): **Treatment policy strategy**<br><br>IcEv2 (Non-study COVID-19 vaccine): **Treatment policy strategy**<br><br>IcEv3 (Early infection): **Principal stratum strategy for analyses where events only counted from 7 days after second vaccination**<br><br>**Composite strategy where events counted at any time after first vaccination**<br><br>IcEv4 (Study infection): Composite strategy<br><br>IcEv5 (Confounding On-study Medications): **Treatment policy strategy** | Correction of errors |
| Section Section 8.3.1 Table 16 (estimand 2 – Estimand Description) | The proportion of participants (and 95% confidence interval) with virologically confirmed COVID-19 (Appendix 2) detected after 7 days post the second study vaccine dose.<br><br>Proportion of participants (and 95% confidence interval) with virologically confirmed COVID-19 (Appendix 2) detected at any time after first vaccine dose | VE estimated as 1-HR, where HR is the hazard ratio from the adjusted Cox Proportional Hazard model for virologically confirmed COVID-19 detected after 7 days post the second study vaccine dose.<br><br>VE estimated as 1-HR, where HR is the hazard ratio from the adjusted Cox Proportional Hazard model for virologically confirmed COVID-19 detected at any time after first vaccine dose | Correction made in the SAP to align with planned analysis method |



Arcturus / Cytel Confidential: This is a controlled document and shall not be changed, saved, copied or reproduced by any unauthorized person.

| Protocol Section | Analysis method described originally in the protocol | Planned Analysis | Reasons of changes |
|---|---|---|---|
| Section 8.3.1 - Table 17 (estimand 3 - rationale for strategies) | Events occurring after IcEv2 could contribute spurious safety data so a treatment policy is used for these events and events occurring after this IcEv's will be summarized separately. Sensitivity analyses will be conducted in the ITT population where these events will be included (composite strategy). | No Sensitivity analysis of unsolicited AEs for ITT analysis set will be produced | Statistical analysis of safety data will be based on the Phase 1/2/3a SAS and Phase 2/3a SAS and reactogenicity analysis set (RAS). |
| 8.3.1 Table 17 Estimands for Primary Safety Analyses | Estimand 4, IcEv1 (Death): Composite strategy | Amended to Treatment policy strategy | Correction of error |
| Section 8.3.1 Table 17 (estimand 4 - Reactogenicity) | Events occurring after non-study vaccine administration or early COVID-19 infection could contribute spurious reactogenicity data, so only the events occurring prior the non-study vaccine administration or early COVID-19 infection will be included in the analysis. Events occurring after these will be summarized separately. | In SAP part 2, it was specified that no censoring of events after receipt of non-study COVID-19 vaccine would be performed. All events were to be presented, irrespective of whether non-study vaccine was received.<br>This rule will be applied in this final SAP i.e. a Treatment policy strategy will be applied for icEv2. | Randomization should ensure no imbalance in the number of participants receiving non-study COVID-19 vaccine. |
| Section 8.4.2 Phase 3b Primary hypothesis | VE is estimated as 1-HR, where HR is the hazard ratio from the adjusted Cox Proportional Hazard model, and will be presented together with 95%CIs. The primary efficacy objective will be met if the lower limit of the 95% CI for VE exceeds 30%. | VE is estimated as 1-HR, where HR is the hazard ratio from the adjusted Cox Proportional Hazard model, and will be presented together with 95%CIs. The primary efficacy objective will be met if the lower limit of the 95% CI for VE exceeds 30%. The secondary efficacy objectives will be met if the lower limit of the 95% CI for VE exceeds 0%. | Add the hypothesis testing for the secondary efficacy objectives. |
| Section 8.6 Subgroups | Subgroups for which immunogenicity, safety and efficacy may be evaluated:<br>-Sex<br>-Age group<br>-Risk group | Subgroups for which immunogenicity, safety and efficacy may be evaluated:<br>-Sex<br>-Age group<br>-Risk group | Exclude "Race/ethnicity" from the subgroup analysis, as the majority of participants (>90%) belong to one ethnicity (Kihn). |



*Arcturus / Cytel Confidential: This is a controlled document and shall not be changed, saved, copied or reproduced by any unauthorized person.*

| Protocol Section | Analysis method described originally in the protocol | Planned Analysis | Reasons of changes |
|---|---|---|---|
| | -Race/ethnicity | -Region of study sites | Add “Region of study sites” in the subgroup analysis due to the heterogeneity of background risk among different sites. |
| Section 8.7.1.1 General Considerations for Phase 1/2/3a | The Clopper-Pearson 95% CIs will be presented for safety evaluation, including solicited and unsolicited AEs. | The Clopper-Pearson 95% CIs will be presented at SOC and overall summary level<br><br>Further change made in this SAP:<br>Clopper Pearson CIs will also not be presented at SOC and overall summary level and only incidence rates will be presented.<br>This applies to all Phases analysed. | To avoid crowded table with unnecessary 95%CI for each event term.<br><br>Not presenting confidence interval for incidence rates of adverse events is a standard approach in analyzing clinical trial data, except when specific hypothesis is tested. This is due to multiplicity issues, potentially invalidating the interpretation of the confidence intervals. |
| Section 9.1 Demographic and Other Baseline Characteristics | Baseline demographics and characteristics, including age, height, weight, sex, ethnicity, BMI, age group, and risk group will be summarized for modified intention-to-treat (mITT), Safety (SAS), and immunogenicity (IAS) populations by treatment group (…). | If the number of participants included in the Per Protocol (PP) and Reactogenicity Analysis Set (RAS) are ≥10% different from mITT and SAS, the analysis for PP and RAS population will be performed. | To clarify the analysis sets performed in the baseline demographics and characteristics analyses. |

## 7.3 Summary of changes from protocol documented in SAP Part 1 and SAP Part 2 not implemented in this SAP

The below table describes changes introduced in SAP Part 1 or SAP Part 2 that are not implemented in this SAP.



*Arcturus / Cytel Confidential: This is a controlled document and shall not be changed, saved, copied or reproduced by any unauthorized person.*

| Protocol Section | Analysis method described originally in the protocol | Planned Analysis | Reasons of changes |
|---|---|---|---|
| Section 3.1 Phase 3b Primary Objectives and Endpoints | Single efficacy primary endpoint | In SAP Part 2, it was specified that the first secondary endpoint for Phase 3b in the protocol (first occurrence of severe COVID-19) will be analyzed as a second primary endpoint.<br>In this SAP, first occurrence of severe COVID-19 will be analyzed as the first secondary endpoint for Phase 3b as specified in the protocol | Regulatory feedback. |
| Section 3.4 Reactogenicity Analysis Set (RAS) | Protocol: Includes all participants who receive a dose of study vaccine (ARCT-154 or placebo) prior to the specific window of observation (e.g., dose 1, dose 2) and who has returned at least a partially completed diary card reflecting that window of observation.<br><br>SAP Part1/2: the RAS includes all participants who receive a dose of study vaccine (ARCT-154 or placebo) prior to the specific window of observation (e.g., dose 1, dose 2) and who has returned at least a partially completed diary card reflecting that window of observation or have at least one reactogenicity report entered in the EDC for the 30 minute observation period after vaccination. | In this SAP, The RAS includes all participants who receive any dose of study vaccine (ARCT-154 or placebo or ChAdOx1) and provide at least 1 reactogenicity diary report. | The condition to receive a dose of study vaccine prior to the specific window of observation will not be implemented in the analysis set definition. Instead participants who did not receive a dose of study vaccine prior to the specific window of observation will not be included in the computation of the percentages in the specific window. |
| Section 8.3.1 Table 17 (estimand 3) Unsolicited Adverse Events | In the protocol, it was specified that events occurring after non-study vaccine administration or early COVID-19 infection could contribute spurious safety data, so only the events occurring prior the non-study vaccine administration or early | Events occurring after non-study vaccine administration or early COVID-19 infection could contribute spurious safety data, so only the events occurring prior the non- | Reverting back to protocol definition. |



Arcturus / Cytel Confidential: This is a controlled document and shall not be changed, saved, copied or reproduced by any unauthorized person.

| Protocol Section | Analysis method described originally in the protocol | Planned Analysis | Reasons of changes |
|---|---|---|---|
| | COVID-19 infection will be included in the analysis. Events occurring after these will be summarized separately.<br><br>In SAP part 2 section 8.1.2, it was specified that no censoring of events after receipt of non-study COVID-19 vaccine would be performed. All events would be presented, irrespective of whether non-study vaccine was received. The rationale provided for the change was the following:<br><br>Randomization should ensure no imbalance in the number of participants receiving non-study COVID-19 vaccine. | study vaccine administration or early COVID-19 infection will be included in the analysis. | |
| Section 8.2 Safety Analysis Set (SAS) | Includes all participants who receive any dose of study vaccine (ARCT-154 or placebo). Participants will be analyzed according to the study vaccine received. | In SAP Part 1/2, the definition was modified to "Includes all participants who receive any dose of study vaccine (ARCT-154 or placebo). **Participants with no safety data after receipt of study vaccine will not be included in the SAS.** Participants will be analyzed according to the study vaccine received."<br><br>This change in not implemented int eh final SAP. | Reverting back to protocol definition. |
| Section 8.6 Subgroups | To determine consistency of immunogenicity across various subgroups in Phase 1/2/3a and Phase 3c, the immunogenicity and the 95% CIs may be estimated using similar methods to the primary analyses for groups stratified by the following classification variables, if feasible. | In SAP part 1, section 10, subgroup analyses were described as "The Cochran-Mantel-Hanenszel Statistics will be performed for the stratified analysis of seroconversion. Breslow-Day test for homogeneity p-value will be calculate to determine the homogeneity of the stratum specific effect."<br><br>As per this SAP, these analyses will not be | Comparison of immunogenicity results of an active vaccine to placebo for statistical significance provides no clinical interpretation. |



Arcturus / Cytel Confidential: This is a controlled document and shall not be changed, saved, copied or reproduced by any unauthorized person.

| Protocol Section | Analysis method described originally in the protocol | Planned Analysis | Reasons of changes |
|---|---|---|---|
| | | performed and no -values will be computed. | |



Arcturus / Cytel Confidential: This is a controlled document and shall not be changed, saved, copied or reproduced by any unauthorized person.

# 8 REFERENCES

Please see the protocol Section 13 for relevant study references.



Arcturus / Cytel Confidential: This is a controlled document and shall not be changed, saved, copied or reproduced by any unauthorized person.

# 9 APPENDICES

## 9.1 Schedule of Assessments

Protocol Appendix 1 contains the study's schedule of assessments.



Arcturus / Cytel Confidential: This is a controlled document and shall not be changed, saved, copied or reproduced by any unauthorized person.

## 9.2 Estimands

The following sections provide full description of the protocol estimands. Updates made to these are described in section 7 .

### 9.2.1 Estimands for Phase 1/2/3a Substudy and Phase 3c Substudy Primary, Secondary and Exploratory Immunogenicity Endpoints

| **Estimand Label** | **Estimand 1 (immunogenicity)** |
|---|---|
| Estimand Description | NAb by surrogate virus neutralization test (primary) and NAb by PRNT50 and BAb (secondary) responses at defined time points following vaccination (as defined in Section 1.2) with ARCT-154 or placebo. The primary endpoint will be measured based on responses at Day 57. |
| Target Population | Vaccinated participants (as defined by eligibility criteria) in Phases 1, 2 and 3a. The population includes participants without evidence of COVID-19, SARS-CoV-2 infection, or nucleocapsid antibody positivity on or prior to Day 57. |
| Endpoint | NAb and BAb responses as defined in Section 1.2 |
| Treatment conditions | ARCT-154 dose 5 µg or placebo administered on Day 1 and Day 29. |
| Population-level Summary | • Geometric mean concentrations for NAb and BAb<br>• The geometric mean ratio (ARCT-154 /Placebo) for NAb and BAb concentrations<br>• Proportion of participants in each study vaccine group that demonstrate seroconversion (as defined by 4-fold increase in antibody concentration [based on international units, if available] from baseline).<br>• Geometric mean fold rise (GMFR) for NAb and BAb |
| Intercurrent events (IcEv) Strategy | |
| IcEv1 (Death) | Treatment policy strategy |
| IcEv2 (Non-Study vaccine) | While on treatment strategy |
| IcEv3 (Early infection) | Principal stratum strategy |
| IcEv4 (Study infection) | Principal stratum strategy |
| IcEv5 (confounding on-Study Medications) | While on treatment strategy |
| Rationale for strategies | IcEv2 and IcEv5 could be potentially confounding to the analysis of the endpoint and result in censoring of the participant for all endpoint assessments after the time of the IcEv (while on treatment). IcEv4 and IcEv3 could also be potentially confounding but it is more difficult to determine the |



Arcturus / Cytel Confidential: This is a controlled document and shall not be changed, saved, copied or reproduced by any unauthorized person.

| | |
|---|---|
| | precise timing of these events, so a principal stratum strategy is used to exclude these participants from immunogenicity analyses. |

### 9.2.2 Estimands for Phase 1/2/3a, Phase 3b, and Pooled Primary, Secondary and Exploratory Efficacy Analyses

| **Estimand Label** | **Protocol Estimand 2 (Vaccine Efficacy)** |
|---|---|
| Estimand Description | • VE estimated as 1-HR, where HR is the hazard ratio from the adjusted Cox Proportional Hazard model for virologically confirmed COVID-19 detected after 7 days post the second study vaccine dose.<br>• VE estimated as 1-HR, where HR is the hazard ratio from the adjusted Cox Proportional Hazard model for virologically confirmed COVID-19 detected at any time after first vaccine dose |
| Target Population | Eligible participants who have received both doses of study vaccine in the initial series. The population includes participants without evidence of COVID-19, SARS-CoV-2 infection, or nucleocapsid antibody positivity on or prior to Day 35. |
| Endpoint | VE for COVID-19/severe COVID 19/death due to COVID-19 (dependent on the endpoint concerned) detected after 7 days post the second study vaccine dose and based upon virologically confirmed COVID-19, estimated as 1-HR, where HR is the hazard ratio from the adjusted Cox Proportional Hazard model<br>Additional endpoints will include VE for virologically confirmed COVID-19 detected at any time after first vaccine dose estimated as 1-HR, where HR is the hazard ratio from the adjusted Cox Proportional Hazard model. |
| Treatment Conditions | ARCT-154 dose 5 µg or placebo administered on Day 1 and Day 29. |
| Population-Level Summary | See 'Endpoint' above |
| Intercurrent Event (IcEv) Strategy | |



Arcturus / Cytel Confidential: This is a controlled document and shall not be changed, saved, copied or reproduced by any unauthorized person.

| | |
|---|---|
| • IcEv1 (Death not due to COVID-19) | Treatment policy strategy |
| • IcEv2 (Non-study COVID-19 vaccine) | Treatment policy strategy |
| • IcEv3 (Early infection) | Principal stratum strategy for analyses where events only counted from 7 days after second vaccination<br>Composite strategy where events counted at any time after first vaccination |
| • IcEv4 (Study infection) | Composite strategy |
| • IcEv5 (Confounding On-study Medications) | Treatment policy strategy |
| Rationale for Strategies | Early infection (IcEv3) results in complete exclusion from analyses from the primary endpoint because prior to Day 36, the vaccine may not have achieved full efficacy. Additional secondary and exploratory analyses are performed where participants with early infection occurring after Day 1 but before Day 36 are included. |

### 9.2.3 Estimands for Primary Safety Analyses for Phase 1/2/3a, Phase 3b, Phase 3c and Pooled Cohorts

| **Estimand Label** | **Estimand 3 (Safety – Unsolicited AEs/AEs leading to discontinuation or withdrawal/MAAE/SAE)** |
|---|---|
| Estimand Description | Count and percentage of vaccinated participants who would develop unsolicited AEs, AEs leading to discontinuation/withdrawal, MAAEs, and SAEs.<br>These will be evaluated with each dose of study vaccine, ARCT-154 or placebo. |
| Target Population | The population who are enrolled in the study and receive any dose of study vaccine |



Arcturus / Cytel Confidential: This is a controlled document and shall not be changed, saved, copied or reproduced by any unauthorized person.

| | |
|---|---|
| Endpoint | Occurrence of unsolicited AEs will be analyzed up to 28 days after each study vaccine administration.<br><br>Occurrence of AEs leading to discontinuation/withdrawal, MAAEs, or SAEs will be analyzed up to Day of Switchover/Further Study Vaccine, receipt of non-study vaccine, early withdrawal, or last on-study assessment visit, whichever comes first.<br><br>Occurrence of AEs leading to discontinuation/withdrawal, MAAEs and SAEs after the Switchover/Further Study Vaccine will be recorded and summarized for the interval from the switchover/Further Study Vaccine up to Day 394 (Phase 1/2/3a and Phase 3b only).<br><br>For Phase 1/2/3a and 3b, AEs in participants who do not undergo Switchover/Further Study Vaccine will be recorded for the interval from Day 92 up to Day 394 |
| Treatment Conditions | Phase 1/2/3a and 3b: ARCT-154 dose 5 µg or placebo administered on Day 1, Day 29, or at Switchover/Further Study Vaccine (Day 92 and Day 120)<br><br>Phase 3c: ARCT-154 dose 5 µg or ChAdOx1 administered on Day 1 and Day 29. |
| Population-Level Summary | Percentage of vaccinated participants who develop each type of AE |
| Intercurrent Event (IcEv) Strategy | |
| IcEv1 (Death not due to COVID-19) | Composite strategy |
| IcEv2 (Non-study vaccine) | While on treatment strategy |
| IcEv3 (Early infection) | Composite strategy |



Arcturus / Cytel Confidential: This is a controlled document and shall not be changed, saved, copied or reproduced by any unauthorized person.

| | |
|---|---|
| IcEv4 (Study infection) | Composite strategy |
| IcEv5 (Confounding On-study Medications) | treatment policy strategy |
| Rationale for Strategies | Events occurring after IcEv2 could contribute spurious safety data so a while on treatment policy is used for these events and events occurring after this IcEv's will be summarized separately. |
| **Estimand Label** | **Estimand 4 (Reactogenicity – Solicited AEs)** |
| Estimand Description | Count and percentage of vaccinated participants who would develop solicited AEs.<br>These will be evaluated after vaccination on Day 1 and Day 29, ARCT-154 or placebo. |
| Target Population | Vaccinated adults providing at least 1 Diary assessment, including reported absence of solicited events. |
| Endpoint | Occurrence of solicited AEs:<br>Solicited AEs within 7 days after study vaccination on Day 1 and Day 29, by Toxicity Grade |
| Treatment Conditions | Placebo or ARCT-154 at dose 5 µg administered on Day 1 and Day 29. |
| Population-Level Summary | Percentage of vaccinated participants who provide at least 1 safety assessment. |
| Intercurrent Event (IcEv) Strategy | |
| IcEv1 (Death not due to COVID-19) | Treatment policy strategy |
| IcEv2 (Non-study vaccine) | Treatment policy strategy |
| IcEv3 (Early infection) | Treatment policy strategy |



*Arcturus / Cytel Confidential: This is a controlled document and shall not be changed, saved, copied or reproduced by any unauthorized person.*

| | |
|---|---|
| IcEv4 (Study infection) | Composite strategy |
| IcEv5 (Confounding Concomitant Medications) | Treatment policy strategy |
| Rationale for Strategies | A treatment policy strategy is used for assessing safety irrespective of occurrence of IcEv2, IcEv3, IcEv5 |



Arcturus / Cytel Confidential: This is a controlled document and shall not be changed, saved, copied or reproduced by any unauthorized person.

## 9.3 Overview of assay and available analyzed samples by phase

| Lab. | Assay | | Timepoints |
|---|---|---|---|
| NIHE | Serum NAb titer against SARS-CoV-2 | Genscript SARS-CoV-2 surrogate virus neutralization test (sVNT) | • Phase 1: at Day 1, Day 29, Day 57, Day 92<br>• Phase 2: at Day 1, Day 29, Day 57, Day 92, D120<br>• Phase 3a: at Day 1, Day 29, Day 57, Day 92, D120 |
| | | plaque reduction neutralization test (PRNT50) (live virus assay) :<br>- Ancestral-clinical isolate variant<br>- Delta-clinical isolate variant | • Phase 1: at Day 1, day 29, Day 57<br>• Phase 2: at Day 1, day 29, Day 57<br>• Phase 3a: no data available |
| | Serum IgG antibodies binding (bAB) the SARS-CoV-2 spike protein-Siemens Advia platform | | • Phase 1: at Day 1, Day 29, Day 57, Day 92<br>• Phase 2: at Day 1, Day 29, Day 57, Day 92, D120<br>• Phase 3a: at Day 1, Day 29, Day 57, Day 92, D120 |
| PPD | Serum NAb titer against SARS-CoV-2: Pseudovirus microneutralization test (MNT) | Ancestral-Hu-1 [D614G variant (VAC62 validated) | • Phase 1: Day 1 and Day 57<br>• Phase 2: Day 1 and Day 57, Day 92 and D120<br>• Phase 3a: Day 1 and Day 57, Day 92 and D120 |
| | | Delta variant (VAC120 )<br>○ | • Phase 1: no data available<br>• Phase 2: Day 1 and Day 57, Day 92 and D120<br>• Phase 3a: Day 1 and Day 57, Day 92 and D120 |
| | | Omicron BA.1 variant (VAC122 validated) | • Phase 1: no data available<br>• Phase 2: Day 1, Day 57, Day 92, Day 120<br>• Phase 3a: Day 1, Day 57, Day 92, Day 120 |
| | | - S | • Phase 1: at Day 1 and Day 57 |



Arcturus / Cytel Confidential: This is a controlled document and shall not be changed, saved, copied or reproduced by any unauthorized person.

| | | | |
|---|---|---|---|
| | ACE2 receptor binding inhibition sVNT assay: multiplex neutralization assay (VAC114) | - S_B.1.1.7<br>- S_B.1.351<br>- S_B.1.526.1<br>- S_B.1.617<br>- S_B.1.617.1<br>- S_B.1.617.2<br>- S_B.1.617.3<br>- S_P1<br>- S_P2 | • Phase 2: at Day 1 and Day 57<br>• Phase 3a: no data available |
| | Serum IgG antibodies binding the SARS-CoV-2 spike : Mesoscale Discovery (MSD) multiplex assay | - Spike (S)<br>- Receptor binding domain (RBD)<br>- Nucleocapsid (N)<br><br>(antigens of the Ancestral variant SARS-CoV-2) | • Phase 1: at Day 1 and Day 57<br>• Phase 2: at Day 1 and Day 57<br>• Phase 3a: no data available |



*Arcturus / Cytel Confidential: This is a controlled document and shall not be changed, saved, copied or reproduced by any unauthorized person.*

## 9.4 Laboratory Conversion Factors

G/L original unit corresponds with $10^9$/L SI unit for white blood cells and platelets. No other conversions are needed.

The conversion factor relating IU/mL to AU/mL for NAb by MNT assay, D614G variant is 1 IU/mL = 1.275 AU/mL.

The conversion factor relating IU/mL to AU/mL for NAb by sVNT (NIHE) is 1 IU/ml=Titer (AU/ml)/12.3

The conversion factor relating BAU/mL to AU/mL for Serum IgG antibodies binding the SARS-CoV-2 spike, MSD multiplex assay, Spike antigen is 1 BAU/mL = 0.00901*(AU/mL).

The conversion factor relating BAU/mL to AU/mL for Serum IgG antibodies binding the SARS-CoV-2 spike, MSD multiplex assay, RBD antigen is 1 BAU/mL = 0.0272*(AU/mL).



Arcturus / Cytel Confidential: This is a controlled document and shall not be changed, saved, copied or reproduced by any unauthorized person.

## 9.5 Example SAS code

**Time to Event/Hazard Ratio used for VE%:**

```
proc phreg data=<input>;
        class Treatment(ref='Placebo') Risk_group site_region;
        model aval*cnsr(1) = Treatment Risk_group site_region;
        estimate 'HR' Treatment 1 /exp cl;
        ods output estimates=est;
run;
```

**Categorical 95% CIs:**

```
proc freq data=<input>;
  by treatment;
    table <response>/bin(CL=CLOPPERPEARSON);
run;
```



*Arcturus / Cytel Confidential: This is a controlled document and shall not be changed, saved, copied or reproduced by any unauthorized person.*

# Arcturus ARCT-154_Final Analysis SAP

Final Audit Report 2023-07-24

| | |
|---|---|
| Created: | 2023-07-21 |
| By: | [redacted] |
| Status: | Signed |
| Transaction ID: | CBJCHBCAABAAam00aE9Y7nforZUB0Dd_8HTt_KD6Lfw6 |

## "Arcturus ARCT-154_Final Analysis SAP" History

Document created by [redacted]
2023-07-21 - 4:55:19 PM GMT- IP address: [redacted]

Document emailed to [redacted] for signature
2023-07-21 - 4:57:09 PM GMT

Document emailed to [redacted] for signature
2023-07-21 - 4:57:09 PM GMT

Document emailed to [redacted] for signature
2023-07-21 - 4:57:09 PM GMT

[redacted] authenticated with Adobe Acrobat Sign.
2023-07-21 - 4:57:59 PM GMT

Document e-signed by [redacted]
Signing reason: Approve
Signature Date: 2023-07-21 - 4:57:59 PM GMT - Time Source: server- IP address: [redacted]

Email viewed by [redacted]
2023-07-21 - 5:21:17 PM GMT- IP address: [redacted]

[redacted] authenticated with phone by verifying one-time code sent to the phone number +X XXX-XXX-2326
Challenge: The user opened the agreement.
2023-07-23 - 5:40:12 AM GMT

Signer [redacted] entered name at signing as [redacted]
2023-07-23 - 5:41:52 AM GMT- IP address: [redacted]
