# Supplementary material for: Safety, immunogenicity and efficacy of the self-amplifying mRNA ARCT-154 COVID-19 vaccine: pooled phase 1, 2, 3a and 3b randomized, controlled trials
Source: Nat Commun. 2024 May 14;15:4081. doi: 10.1038/s41467-024-47905-1 (PMC11094049; doi:10.1038/s41467-024-47905-1)
Supplement: Supplementary file 3 — Reporting Summary [file 41467_2024_47905_MOESM3_ESM.pdf]

nature portfolio

Corresponding author(s): Xuan-Hung Nguyen

Last updated by author(s): 04/04/2024

Reporting Summary

Nature Portfolio wishes to improve the reproducibility of the work that we publish. This form provides structure for consistency and transparency in reporting. For further information on Nature Portfolio policies, see our Editorial Policies and the Editorial Policy Checklist.

Statistics

For all statistical analyses, confirm that the following items are present in the figure legend, table legend, main text, or Methods section.

n/a	Confirmed	
☐	☒	The exact sample size (*n*) for each experimental group/condition, given as a discrete number and unit of measurement
☒	☐	A statement on whether measurements were taken from distinct samples or whether the same sample was measured repeatedly
☐	☒	The statistical test(s) used AND whether they are one- or two-sided<br>*Only common tests should be described solely by name; describe more complex techniques in the Methods section.*
☐	☒	A description of all covariates tested
☐	☒	A description of any assumptions or corrections, such as tests of normality and adjustment for multiple comparisons
☐	☒	A full description of the statistical parameters including central tendency (e.g. means) or other basic estimates (e.g. regression coefficient) AND variation (e.g. standard deviation) or associated estimates of uncertainty (e.g. confidence intervals)
☐	☒	For null hypothesis testing, the test statistic (e.g. *F*, *t*, *r*) with confidence intervals, effect sizes, degrees of freedom and *P* value noted<br>*Give P values as exact values whenever suitable.*
☒	☐	For Bayesian analysis, information on the choice of priors and Markov chain Monte Carlo settings
☐	☒	For hierarchical and complex designs, identification of the appropriate level for tests and full reporting of outcomes
☒	☐	Estimates of effect sizes (e.g. Cohen's *d*, Pearson's *r*), indicating how they were calculated

Our web collection on statistics for biologists contains articles on many of the points above.

Software and code

Policy information about availability of computer code

Data collection	Electronic Data Capturing system for clinical study Case Report Forms: DFdiscover version 5.4.0.<br>Electronic Patient Report Outcome system for Patient diary: MedProve Version 1.0
Data analysis	SAS platform using v9.4 run under WIN 10.0.14393 Server

For manuscripts utilizing custom algorithms or software that are central to the research but not yet described in published literature, software must be made available to editors and reviewers. We strongly encourage code deposition in a community repository (e.g. GitHub). See the Nature Portfolio guidelines for submitting code & software for further information.

Data

Policy information about availability of data

All manuscripts must include a data availability statement. This statement should provide the following information, where applicable:

- Accession codes, unique identifiers, or web links for publicly available datasets
- A description of any restrictions on data availability
- For clinical datasets or third party data, please ensure that the statement adheres to our policy

The data for figures in this study are provided in the Source Data file. The data that support the findings of this study are available in the paper and its Supplementary Information or from the corresponding author upon request. Source data are provided with this paper.

Research involving human participants, their data, or biological material

Policy information about studies with human participants or human data. See also policy information about sex, gender (identity/presentation), and sexual orientation and race, ethnicity and racism.

Reporting on sex and gender	sex (self reporting) was collected on eCRF at the time of enrollment for each participant in the clinical trial
Reporting on race, ethnicity, or other socially relevant groupings	ethnicity information was collected at the time of enrollment . Approximately 99% of the study participants ethnicity was The Kinh (Asian)
Population characteristics	important demographic variables collected at enrollment were :<br>age: Age groups: ≥18 to <60 years and ≥60 years<br>Age and health risk for severe disease: ≥18 to <60 years and "healthy," ≥18 and <60 years and "at risk," and ≥60 years<br>Sex (female, male).<br>Analyses of Efficacy, Immunogenicity and Safety were repeated in each populations subgroup. Results are reported on this manuscript
Recruitment	Participants in all study parts underwent informed consent and screening procedures prior to enrollment. After enrollment, participants were randomized to treatment groups. Study vaccines were given in a supervised medical setting.
Ethics oversight	An Independent Ethics Committee (IEC) or Institutional Review Board (IRB) applicable for each study center approved the final clinical study protocol (CSP), including the final version of the informed consent form (ICF) and any other written information and/or materials that were provided to the study participants. The investigator ensured the distribution of these documents to the applicable IEC/IRB and to the study center staff.<br>The opinion of the IEC/IRB was provided in writing.

Note that full information on the approval of the study protocol must also be provided in the manuscript.

Field-specific reporting

Please select the one below that is the best fit for your research. If you are not sure, read the appropriate sections before making your selection.

☒ Life sciences ☐ Behavioural & social sciences ☐ Ecological, evolutionary & environmental sciences

For a reference copy of the document with all sections, see nature.com/documents/nr-reporting-summary-flat.pdf

Life sciences study design

All studies must disclose on these points even when the disclosure is negative.

Sample size	For the overall primary efficacy objective of the study, the null hypothesis is that the VE of ARCT-154 to prevent first occurrence of polymerase chain reaction (PCR)-confirmed COVID-19 is ≤30% (ie, H0efficacy: VE ≤0.3).<br>The primary efficacy objective will be met if the lower limit of the 95% CI for VE exceeds 30%.<br>VE is defined as the percent reduction in the hazard of the primary endpoint (ARCT-154 versus placebo). Equivalently, the null hypothesis is as follows:<br>• H0 efficacy: HR >0.7 (equivalently, proportional hazards VE ≤0.3)<br>A Cox proportional hazard model will be used to assess the magnitude of the study group difference (ie, HR) between ARCT-154 and placebo at a 1-sided 0.025 significance level. Data will be presented overall and by the stratification factors.<br>Under the assumption of proportional hazards over time and with 1:1 randomization of ARCT-154 and placebo, a total of 372 COVID-19 cases will provide approximately 90% power to detect a 50% reduction in hazard rate (50% VE), rejecting the null hypothesis with a 1-sided false positive error rate of 0.025.
Data exclusions	The following major protocol deviations were expected to affect efficacy and will lead to exclusion of a participant from the efficacy set:<br>• Received wrong study vaccine up to Day 92<br>• Did not receive study vaccine<br>• Received off-study COVID-19 vaccine (IcEv2) up to Day 92<br>• COVID-19 infection (positive RT-PCR or other COVID-19 test) before Day 36<br>• Result of SARS-CoV-2 anti-nucleocapsid antibodies test is Positive/Undetermined up to Day 36
Replication	NA
Randomization	Phase 1, 2 and 3a participants will be randomly assigned 3:1 (ARCT-154:placebo), while Phase 3b participants will be randomly assigned 1:1 (ARCT-154:placebo).<br>• Participants in Phase 1 and 3b will receive two doses of one type of study vaccine on Day 1 and 29 (ARCT-154 or placebo) and then two doses of the opposite vaccine (placebo or ARCT-154) on Day 92 and 120 (referred to as 'Switchover').<br>• Participants in Phase 2/3a who received ARCT-154 in the initial two-dose vaccination series will be further randomized to receive either ARCT-154 or placebo (in a 3:1 ratio) at Day 92 followed by placebo at Day 120. Participants that received placebo in the initial vaccination series will undergo Switchover to receive ARCT-154 at Day 92 and Day 120.<br>For Phases 2, 3a, 3b and 3c, prior to randomization, participants will be stratified by the following 3 stratification factors:

	• Age < 60 at high risk of severe COVID-19<br>• Age < 60 not at high risk of severe COVID-19<br>• Age ≥ 60 years of age (considered at risk of severe COVID-19 by default)<br>Participants will be defined as "at risk" if they are 60 years of age or older OR have medical history described as putting an individual at risk or possibly at risk of severe .<br>As the eligibility criteria for Phase 1 exclude participants ≥ 60 years of age or otherwise at risk for severe COVID-19, stratification will not occur for Phase 1.
Blinding	The study vaccines were administered in an observer-blind fashion.<br>Each of the study vaccines were prepared by an unblinded trained team member and in accordance with the Pharmacy Manual.<br>Unblinded personnel were assigned to vaccine accountability procedures and were prepared study vaccine for all participants. These personnel were have no study functions other than study vaccine management, documentation, accountability, preparation, and administration. They were not be involved in participant evaluations and will not reveal the identity of study vaccine to either the participant or the blinded study site personnel involved in the conduct of the study unless this information is necessary in the case of an emergency.<br>• Unblinded health care providers were administer the study vaccine. They were be involved in assessments of any study endpoints.<br>• All documents containing unblinded information must kept out of view of the blinded team members and the study participants.<br>• Unblinded site monitors not involved in other aspects of monitoring will be assigned as the study vaccine accountability monitors. They were have responsibilities to ensure that sites are following all proper study vaccine accountability, preparation, and administration procedures.<br>• An unblinded DSMB was perform the ongoing review of safety data from enrolled participants to determine if there are any unexpected risks that warrant modification or termination of the study. The DSMB was provided recommendations to the Sponsor following each data review.<br>In order to maintain an observer-blind design, investigators, site staff, participants, and CRO staff with oversight of study conduct remain blinded to vaccine assignments for the study duration. The Sponsor representatives with direct oversight of the study remain blinded to individual participant vaccine assignments until the time of study unblinding at the final analysis.

Reporting for specific materials, systems and methods

We require information from authors about some types of materials, experimental systems and methods used in many studies. Here, indicate whether each material, system or method listed is relevant to your study. If you are not sure if a list item applies to your research, read the appropriate section before selecting a response.

Materials & experimental systems

n/a	Involved in the study
☒	☐ Antibodies
☒	☐ Eukaryotic cell lines
☒	☐ Palaeontology and archaeology
☒	☐ Animals and other organisms
☐	☒ Clinical data
☒	☐ Dual use research of concern
☒	☐ Plants

Methods

n/a	Involved in the study
☒	☐ ChIP-seq
☒	☐ Flow cytometry
☒	☐ MRI-based neuroimaging

Clinical data

Policy information about clinical studies

All manuscripts should comply with the ICMJEguidelines for publication of clinical research and a completedCONSORT checklist must be included with all submissions.

Clinical trial registration	ClinicalTrial.gov identifier: NCT05012943
Study protocol	Supplied
Data collection	Included
Outcomes	Included
